# Supplementary material for: Efficient access to aliphatic esters by photocatalyzed alkoxycarbonylation of alkenes with alkyloxalyl chlorides
Source: Nat Commun. 2021 Sep 7;12:5328. doi: 10.1038/s41467-021-25628-x (PMC8423752; doi:10.1038/s41467-021-25628-x)
Supplement: Supplementary file 1 — Supplementary Information [file 41467_2021_25628_MOESM1_ESM.pdf]

Supplementary Information

Efficient access to aliphatic esters by photocatalyzed alkoxycarbonylation of alkenes with alkyloxalyl chlorides

Jian-Qiang Chen[1]*, Xiaodong Tu[1], Qi Tang[1], Ke Li[1], Liang Xu[1], Siyu Wang[1], Mingjuan Ji[1], Zhiming Li[2]* & Jie Wu[1,3,4]*

[1] *School of Pharmaceutical and Materials Engineering & Institute for Advanced Studies, Taizhou University, Taizhou 318000, China.*
[2] *Department of Chemistry, Fudan University, 2005 Songhu Road, Shanghai, 200438, China.*
[3] *State Key Laboratory of Organometallic Chemistry, Shanghai Institute of Organic Chemistry, Chinese Academy of Sciences, Shanghai 200032, China.*
[4] *School of Chemistry and Chemical Engineering, Henan Normal University, Xinxiang 453007, China.*

Supplementary Methods

General information

All glassware was thoroughly oven-dried. Chemicals and solvents were either purchased from commercial suppliers or purified by standard techniques. Thin-layer chromatography plates were visualized by exposure to ultraviolet light and/or staining with phosphomolybdic acid followed by heating on a hot plate. Flash chromatography was carried out using silica gel (200−300 mesh). ^{1}H NMR and ^{13}C NMR spectra were recorded on a Bruker AM-400 (400 MHz). The spectra were recorded in deuterochloroform ($CDCl_3$) as solvent at room temperature, ^{1}H and ^{13}C NMR chemical shifts are reported in ppm relative to the residual solvent peak. The residual solvent signals were used as references and the chemical shifts were converted to the TMS scale ($CDCl_3$: δ_H = 7.26 ppm, δ_C = 77.0 ppm). Data for ^{1}H NMR are reported as follows: chemical shift (δ ppm), multiplicity (s = singlet, d = doublet, t = triplet, m = multiplet, dd = doublet, br = broad), integration, coupling constant (Hz) and assignment. Data for ^{13}C NMR are reported as chemical shift. HRMS were performed on a Bruker Apex II mass instrument (ESI). Ultraviolet-visible spectroscopy was performed on a Perkin Elmer Lambda 950 spectrophotometer using CH_3CN as the solvent. All luminescence spectra were surveyed on a Cary Eclipse fluorescence spectrophotometer and equipped with a 1 cm quartz cell. Cyclic voltammetry (CV) studies were carried out on a CHI760E instrument.

General experimental procedure for the preparation of substrates [1]

(a) General procedure for the synthesis of substrates ***2****.*[1]

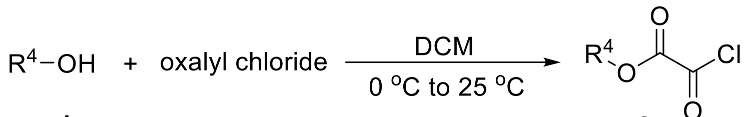

Into a 100 mL round-bottom flask equipped with a magnetic stir-bar was added solution of oxalyl chloride (20 mmol, 2 equiv) in DCM (20 mL). The mixture was stirred at 0 °C, and a solution of an appropriate alcohol **s1** (10 mmol) in dry DCM (20 ml) was added dropwisely over 30 min. After the addition was completed, the mixture was allowed to warm to 25 °C for 2 h. Excess oxalyl chloride was removed by vacuum distillation. The alkyloxyoxalyl chloride **2** was used for the next step without purification.

Ethyl chlorooxoacetate **2a** and methyl chloroglyoxylate are commercially available.

(b) General procedure for the synthesis of substrates 7.[2]

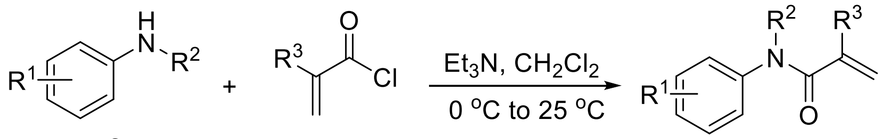

A round-bottom flask (100 mL) was charged with aniline **s2** (10 mmol) and Et_3N (20 mmol, 2 equiv) in dry dichloromethane (40 mL). Then acyl chloride **s3** (15 mmol, 1.5 equiv) was added slowly to the reaction mixture at 0 °C. After that, the ice bath was removed and the mixture was stirred vigorously for 6 hours at 25 °C. After completion of reaction, the mixture was quenched with H_2O and extracted with CH_2Cl_2 (3 × 20 mL). The combined organic phases were washed with saturated brine, dried over anhydrous Na_2SO_4, concentrated under reduced pressure. The residue was purified by column chromatography to afford compound **7**.

General procedure for alkoxycarbonylchlorination

(a) General procedure for the alkoxycarbonylchlorination of activated alkenes

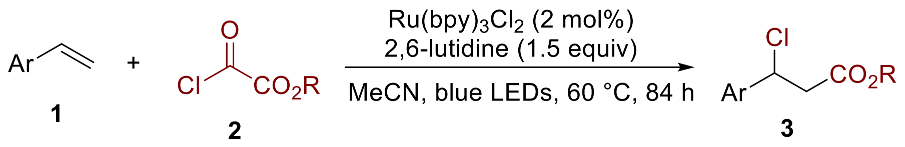

Substrate **1** (0.2 mmol), alkyloxyoxalyl chloride **2** (0.6 mmol) and 2,6-lutidine (32.1 mg, 0.3 mmol) were added to a solution of Ru(bpy)$_3$Cl$_2$ (3.0 mg, 2 mol %) in dry MeCN (4.0 mL) at 25 °C. The heterogenous mixture was degassed by three cycles of freeze-pump-thaw and then placed in the irradiation apparatus equipped with blue LEDs. The resulting mixture was stirred at 60 °C for 84 h. Upon completion of the reaction, the mixture was diluted with ethyl acetate (30 mL), washed with brine (10 x 3 mL), dried with Na_2SO_4. After evaporation of the solvent, the crude product was purified by column chromatography on silica gel to afford the desired product **3**.

(b) General procedure for the alkoxycarbonylchlorination of unactivated alkenes

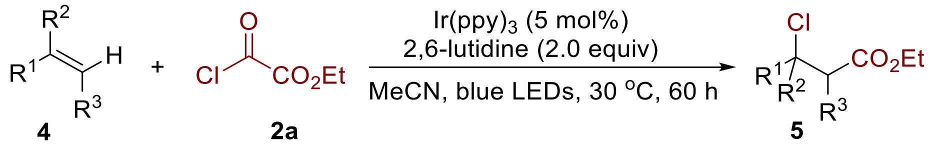

Substrate **4** (0.2 mmol), chlorooxoacetate **2a** (218.4 mg, 1.6 mmol) and 2,6-lutidine (42.8 mg, 0.4 mmol) were added to a solution of Ir(ppy)$_3$ (6.54 mg, 5 mol %) in dry MeCN (4 mL) at 25 °C. The heterogenous mixture was degassed by three cycles of freeze-pump-thaw and then placed in the irradiation apparatus equipped with blue LEDs. The resulting mixture was stirred at 30 °C for 60 h. Upon completion of the reaction, the mixture was diluted with ethyl acetate (30 mL), washed with brine (10 x 3 mL), dried with Na_2SO_4. The solvent was evaporated, and the residue was purified by column chromatography on silica gel to afford the desired product **5**.

General procedure for the synthesis of α,β-unsaturated esters

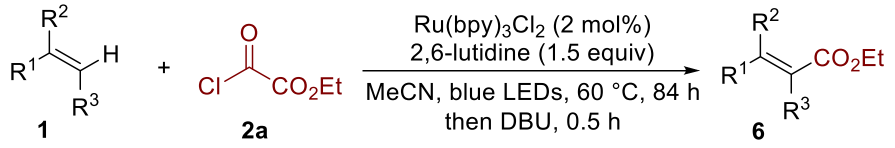

Substrate **1** (0.2 mmol), chlorooxoacetate **2a** (81.9 mg, 0.6 mmol) and 2,6-lutidine (32.1 mg, 0.3 mmol) were added to a solution of Ru(bpy)$_3$Cl$_2$ (3.0 mg, 2 mol %) in dry

MeCN (4 mL) at 25 °C. The heterogenous mixture was degassed by three cycles of freeze-pump-thaw and then placed in the irradiation apparatus equipped with blue LEDs. The resulting mixture was stirred at 60 °C for 84 h. Upon completion of the reaction, 1,8-diazabicyclo[5.4.0]undec-7-ene (152.2 mg, 1.0 mmol) was added and then stirred at 25 °C for 0.5 h. The mixture was diluted with ethyl acetate (30 mL), washed with brine (10 x 3 mL), and dried with Na_2SO_4. The solvent was evaporated, and the crude product was purified by column chromatography on silica gel to afford the desired product **6 (6a-6n**, and **6q)**.

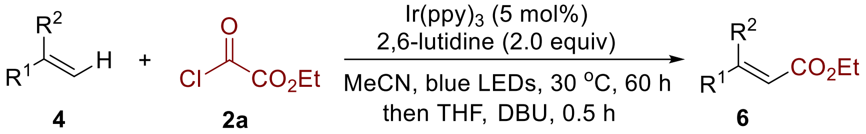

Substrate **4** (0.2 mmol), alkyloxyoxalyl chloride **2a** (218.4 mg, 1.6 mmol) and 2,6-lutidine (42.8 mg, 0.4 mmol) were added to a solution of Ir(ppy)$_3$ (6.54 mg, 5 mol %) in dry MeCN (4 mL) at 25 °C. The heterogenous mixture was degassed by three cycles of freeze-pump-thaw and then placed in the irradiation apparatus equipped with blue LEDs. The resulting mixture was stirred at 30 °C for 60 h. Upon completion of the reaction, the mixture was diluted with ethyl acetate (30 mL), washed with brine (10 x 3 mL), dried with Na_2SO_4. The solvent was evaporated, and the crude product was dissolved in THF (4 mL) After the addition of DBU (152.2 mg, 1.0 mmol), the reaction mixture was stirred at 25 °C for 0.5 h. The mixture was then diluted with ethyl acetate (30 mL), washed with brine (10 x 3 mL), dried with Na_2SO_4. The solvent was evaporated, and the residue was purified by column chromatography on silica gel to afford the desired product **6** (**6o** and **6p**).

General procedure for the synthesis of oxindole derivatives

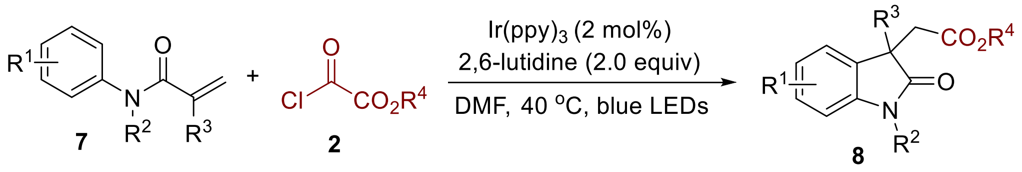

Substrate **7** (0.2 mmol), alkyloxyoxalyl chloride **2** (0.6 mmol) and 2,6-lutidine (42.8 mg, 0.4 mmol) were added to a solution of Ir(ppy)$_3$ (2.62 mg, 2 mol %) in dry DMF (4

mL) at 25 °C. The heterogenous mixture was degassed by three cycles of freeze-pump-thaw and then placed in the irradiation apparatus equipped with blue LEDs. The resulting mixture was stirred at 40 °C until the starting material was completely consumed as monitored by TLC. Upon completion of the reaction, the mixture was diluted with ethyl acetate (30 mL), washed with brine (10 x 3 mL), dried with Na_2SO_4. The solvent was then evaporated, and the crude product was purified by column chromatography on silica gel to afford the desired product **8**.

General procedure for the synthesis of furoindolines

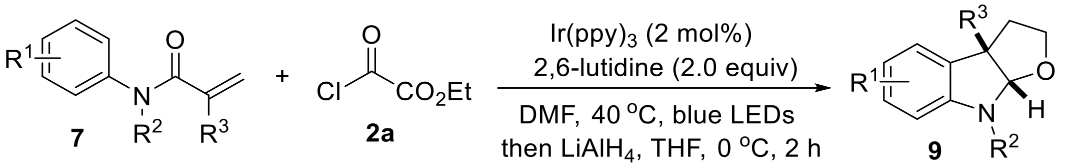

Step 1: Substrate **7** (0.2 mmol), ethyl chlorooxoacetate **2a** (81.9 mg, 0.6 mmol) and 2,6-lutidine (42.8 mg, 0.4 mmol) were added to a solution of $Ir(ppy)_3$ (2.62 mg, 2 mol%) in dry DMF (4 mL) at 25 °C. The heterogenous mixture was degassed by three cycles of freeze-pump-thaw and then placed in the irradiation apparatus equipped with blue LEDs. The resulting mixture was stirred at 40 °C until the starting material was completely consumed as monitored by TLC. Upon completion of the reaction, the mixture was diluted with ethyl acetate (30 mL), washed with brine (10 x 3 mL), dried with Na_2SO_4. The solvent was then evaporated, and the crude product was obtained and was used in the following step without further purification.

Step 2: To a solution of crude product in THF (4 mL) at 0 °C was added $LiAlH_4$ (38 mg, 1.0 mmol) in small portions under nitrogen atmosphere. The reaction mixture was stirred at 0 °C for 2 h, and then the reaction was quenched with the addition of brine (15 mL) and diluted with EtOAc (30 mL). The combined organic layers were washed with brine (3 × 10 mL), dried over Na_2SO_4, filtered and concentrated under reduced pressure. The resulting residue was purified by flash column chromatography with gradient eluents (*n*-hexane/ ethyl acetate = 20/1) to provide **9**.[3]

Initial studies and the reaction optimization

Supplementary Table 1. Initial studies for the photoinduced reaction of 4-vinyl-1,1'-biphenyl **1a** with chlorooxoacetate **2a**. [a,b]

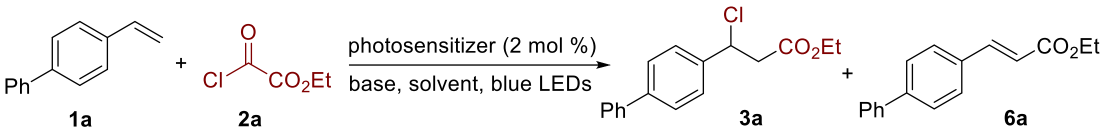

Entry	Photosensitizer	Solvent	Base (equiv)	T (°C)	Time	Yield (3a, %)	3a/6a [b]	1a
1	$Ir(ppy)_3$	MeCN	2,6-Lutidine (3)	40	24 h	30	3:1	0
2	$Ir(ppy)_2(dtbbpy)PF_6$	MeCN	2,6-Lutidine (3)	40	24 h	25	>20:1	0
3	$Ir[d(Me)ppy)_2(dtbbpy)PF_6$	MeCN	2,6-Lutidine (3)	40	24 h	25	12:1	0
4	$Ir[dF(CF_3)ppy)_2(dtbbpy)PF_6$	MeCN	2,6-Lutidine (3)	40	24 h	Complex	-	0
5	4CzIPN	MeCN	2,6-Lutidine (3)	40	24 h	Complex	-	0
6	3CzClIPN	MeCN	2,6-Lutidine (3)	40	24 h	Complex	-	0
7	5CzBN	MeCN	2,6-Lutidine (3)	40	24 h	10	-	0
8	3DPA2FBN	MeCN	2,6-Lutidine (3)	40	24 h	22	>20:1	0
9	$Ru(bpy)_3Cl_2$	MeCN	2,6-Lutidine (3)	40	6 d	42	14:1	38
10	$Ru(bpy)_3Cl_2$	MeCN	2,6-Lutidine (3)	60	72 h	66	11:1	12
11	$Ru(bpy)_3Cl_2$	DMF	2,6-Lutidine (3)	60	72 h	13	1:1.3	54
12	$Ru(bpy)_3Cl_2$	DCM	2,6-Lutidine (3)	60	72 h	19	7:1	55
13	$Ru(bpy)_3Cl_2$	$CHCl_3$	2,6-Lutidine (3)	60	72 h	20	10:1	61
14	$Ru(bpy)_3Cl_2$	THF	2,6-Lutidine (3)	60	72 h	10	-	62
15	$Ru(bpy)_3Cl_2$	Acetone	2,6-Lutidine (3)	60	72 h	18	10:1	61
16	$Ru(bpy)_3Cl_2$	MeCN	2,6-Lutidine (2)	60	84 h	66	>20:1	12
**17**	**$Ru(bpy)_3Cl_2$**	**MeCN**	**2,6-Lutidine (1.5)**	**60**	**84 h**	**85**	**>20:1**	**0**
18	$Ru(bpy)_3Cl_2$	MeCN	2,6-Lutidine (1)	60	84 h	73	>20:1	0
19	$Ru(bpy)_3Cl_2$	MeCN	2,6-Lutidine (0.5)	60	84 h	65	12:1	9
20	$Ru(bpy)_3Cl_2$	MeCN	2,6-di^{t}Bu-Py (1.5)	60	84 h	60	9:1	31
21	$Ru(bpy)_3Cl_2$	MeCN	DABCO (1.5)	60	84 h	13	-	85
22	$Ru(bpy)_3Cl_2$	MeCN	K_2HPO_4 (1.5)	60	84 h	10	-	89
23	$Ru(bpy)_3Cl_2$	MeCN	K_3PO_4 (1.5)	60	84 h	NR	-	-
24	-	MeCN	2,6-Lutidine (1.5)	60	84 h	NR	-	-
25[c]	$Ru(bpy)_3Cl_2$	MeCN	2,6-Lutidine (1.5)	60	84 h	NR	-	-

[a]Reaction conditions: *N*-methyl-*N*-phenylmethacrylamide **1a** (0.2 mmol), chlorooxoacetate **2a** (0.6 mmol), photocatalyst (0.004 mmol), base, solvent (4 mL), blue LEDs, under a N_2 atmosphere. [b]Determined by ^1H NMR analysis using 1,3,5-trimethoxybenzene as an internal standard. [c]In the dark.

Supplementary Table 2. Initial studies for the photoinduced reaction of 4-phenyl-1-butene **4a** with chlorooxoacetate **2a**.[a,b]

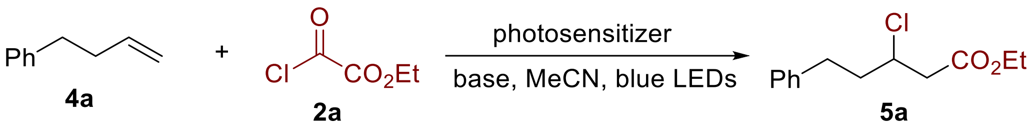

Entry	Photosensitizer (mol%)	2a (equiv)	Base (equiv)	T (ºC)	Time	Yield (%)
1	$Ru(bpy)_3Cl_2$ (2 mol%)	3.0	2,6-Lutidine (1.5)	60	84 h	0
2	$Ir(ppy)_2(dtbbpy)PF_6$ (2 mol%)	3.0	2,6-Lutidine (1.5)	60	60 h	22
3	$Ir(ppy)_3$ (2 mol%)	3.0	2,6-Lutidine (1.5)	60	60 h	39
4	3DPA2FBN (2 mol%)	3.0	2,6-Lutidine (1.5)	60	60 h	36
5	$Ir(ppy)_3$ (2 mol%)	3.0	2,6-Lutidine (1.5)	30	60 h	44
6	$Ir(ppy)_3$ (3 mol%)	3.0	2,6-Lutidine (1.5)	30	60 h	48
7	$Ir(ppy)_3$ (4 mol%)	3.0	2,6-Lutidine (1.5)	30	60 h	52
8	$Ir(ppy)_3$ (5 mol%)	3.0	2,6-Lutidine (1.5)	30	60 h	59
9	$Ir(ppy)_3$ (5 mol%)	4.0	2,6-Lutidine (1.5)	30	60 h	63
10	$Ir(ppy)_3$ (5 mol%)	5.0	2,6-Lutidine (1.5)	30	60 h	67
11	$Ir(ppy)_3$ (5 mol%)	8.0	2,6-Lutidine (1.5)	30	60 h	71
12	$Ir(ppy)_3$ (5 mol%)	8.0	-	30	60 h	20
13	$Ir(ppy)_3$ (5 mol%)	8.0	2,6-Lutidine (1.0)	30	60 h	64
**14**	**$Ir(ppy)_3$ (5 mol%)**	**8.0**	**2,6-Lutidine (2.0)**	**30**	**60 h**	**74**
15	$Ir(ppy)_3$ (5 mol%)	8.0	2,6-Lutidine (3.0)	30	60 h	64
16	$Ir(ppy)_3$ (5 mol%)	8.0	2,6-Lutidine (4.0)	30	60 h	63

[a]Reaction conditions: 4-phenyl-1-butene **4a** (0.2 mmol), chlorooxoacetate **2a**, photocatalyst (0.004 mmol), base, MeCN (4 mL), blue LEDs, under a N_2 atmosphere. [b]Determined by ^1H NMR analysis using 1,3,5-trimethoxybenzene as an internal standard.

Photosensitizer:

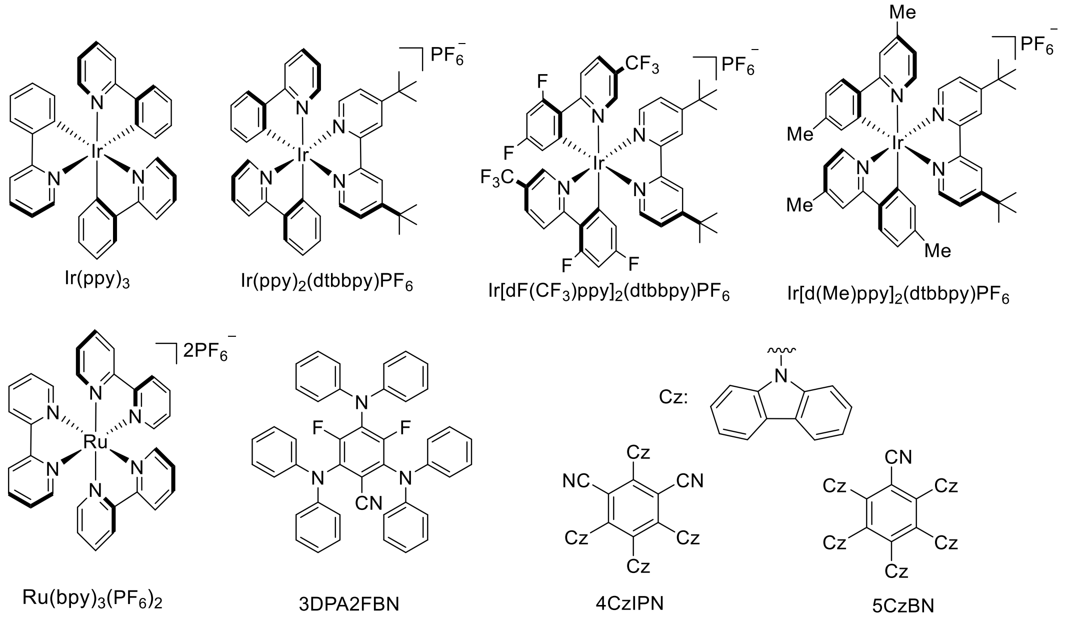

Supplementary Table 3. Initial studies for the photoinduced reaction of *N*-methyl-*N*-phenylmethacrylamide **7a** with chlorooxoacetate **2a**.[a]

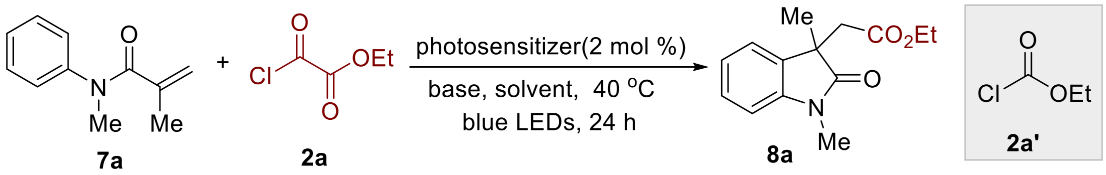

Entry	Photocatalyst	Solvent	Base	Yield (%)[b]
1	$Ir(ppy)_2(dtbbpy)PF_6$	MeCN	2,6-Lutidine	71
2	$Ir(ppy)_3$	MeCN	2,6-Lutidine	80
3	$Ir[dF(CF_3)ppy]_2(dtbbpy)PF_6$	MeCN	2,6-Lutidine	32
4	$Ru(bpy)_3(PF_6)_2$	MeCN	2,6-Lutidine	65
5	3DPA2FBN	MeCN	2,6-Lutidine	76
6	4CzIPN	MeCN	2,6-Lutidine	38
7	5CzBN	MeCN	2,6-Lutidine	29
8	$Ir(ppy)_3$	DCM	2,6-Lutidine	73
9	$Ir(ppy)_3$	THF	2,6-Lutidine	18
10	$Ir(ppy)_3$	Acetone	2,6-Lutidine	9
11	$Ir(ppy)_3$	DMF	2,6-Lutidine	89
12	$Ir(ppy)_3$	DCE	2,6-Lutidine	72
13	$Ir(ppy)_3$	EA	2,6-Lutidine	trace
14	$Ir(ppy)_3$	DMA	2,6-Lutidine	29
15	$Ir(ppy)_3$	Xylenes	2,6-Lutidine	8
16	$Ir(ppy)_3$	DMF	2,6-di^{t}Bu-Py	88
17	$Ir(ppy)_3$	DMF	2,4,6-triMePy	75
18	$Ir(ppy)_3$	DMF	K_2HPO_4	77
19	$Ir(ppy)_3$	DMF	K_2CO_3	12
20	$Ir(ppy)_3$	DMF	K_3PO_4	trace
21[c]	$Ir(ppy)_3$	DMF	2,6-Lutidine	95
22[d]	$Ir(ppy)_3$	DMF	2,6-Lutidine	95
23[c]	-	DMF	2,6-Lutidine	0
24[c,e]	$Ir(ppy)_3$	DMF	2,6-Lutidine	0
25 [c,f]	$Ir(ppy)_3$	DMF	2,6-Lutidine	0

[a] Reaction conditions: *N*-methyl-*N*-phenylmethacrylamide **7a** (0.2 mmol), chlorooxoacetate **2a** (0.4 mmol), photocatalyst (0.004 mmol), base (0.4 mmol), solvent (4 mL), blue LEDs, 40 °C, 24 h, under a N_2 atmosphere. [b] Determined by ^1H NMR analysis using 1,3,5-trimethoxybenzene as an internal standard. [c] In the presence of chlorooxoacetate **2a** (0.6 mmol). [d] **2a** (0.8 mmol), [e] In the dark. [f] Compound **2a'** was used instead of chlorooxoacetate **2a**.

Devices for the photocatalytic reactions

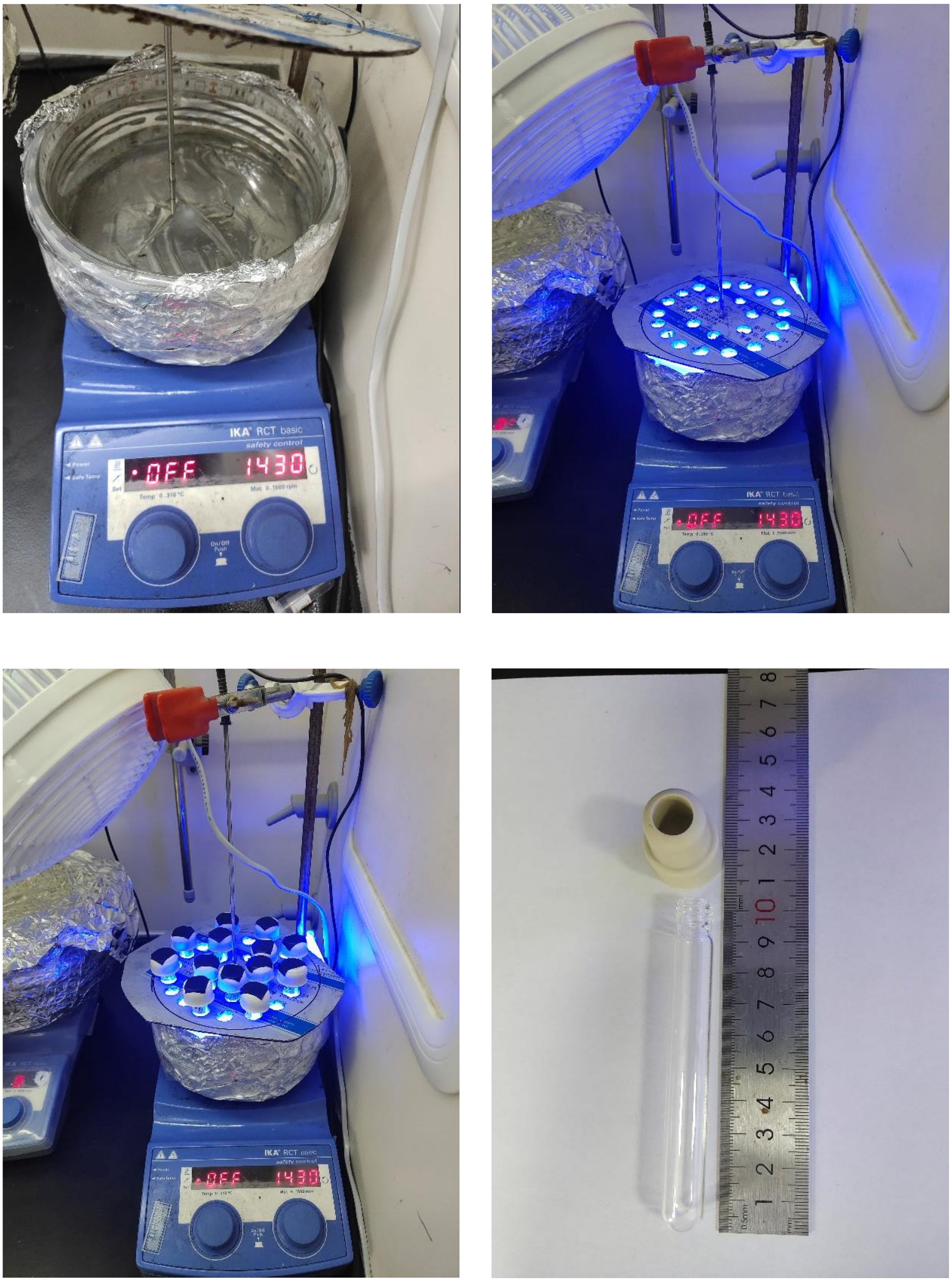

Supplementary Figure 1. Devices for the photocatalytic reactions

Preparation of compound 10i

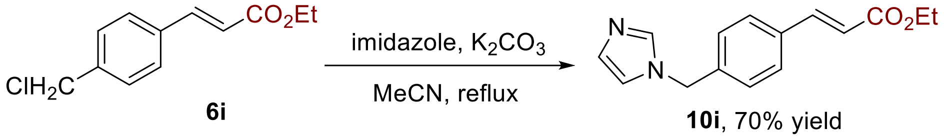

A mixture of potassium carbonate (82.9 mg, 0.6 mmol), imidazole (32.9 mmol) and compound **6i** (0.2 mmol) in dry acetonitrile (2.0 ml) was heated under reflux for 5 h. Then the solvent was evaporated under vacuum and the residue was purified by flash column chromatography (DCM/EtOH = 30/1) to give the product **10i**.[4]

Mechanistic studies

(a) TEMPO trapping experiment.

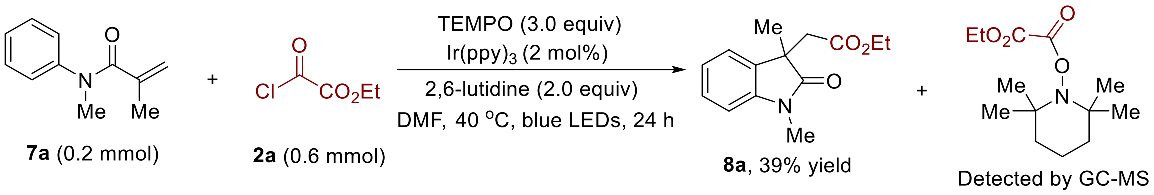

(b) NMR study of ethyl chlorooxoacetate 2a and pyridine mixtures

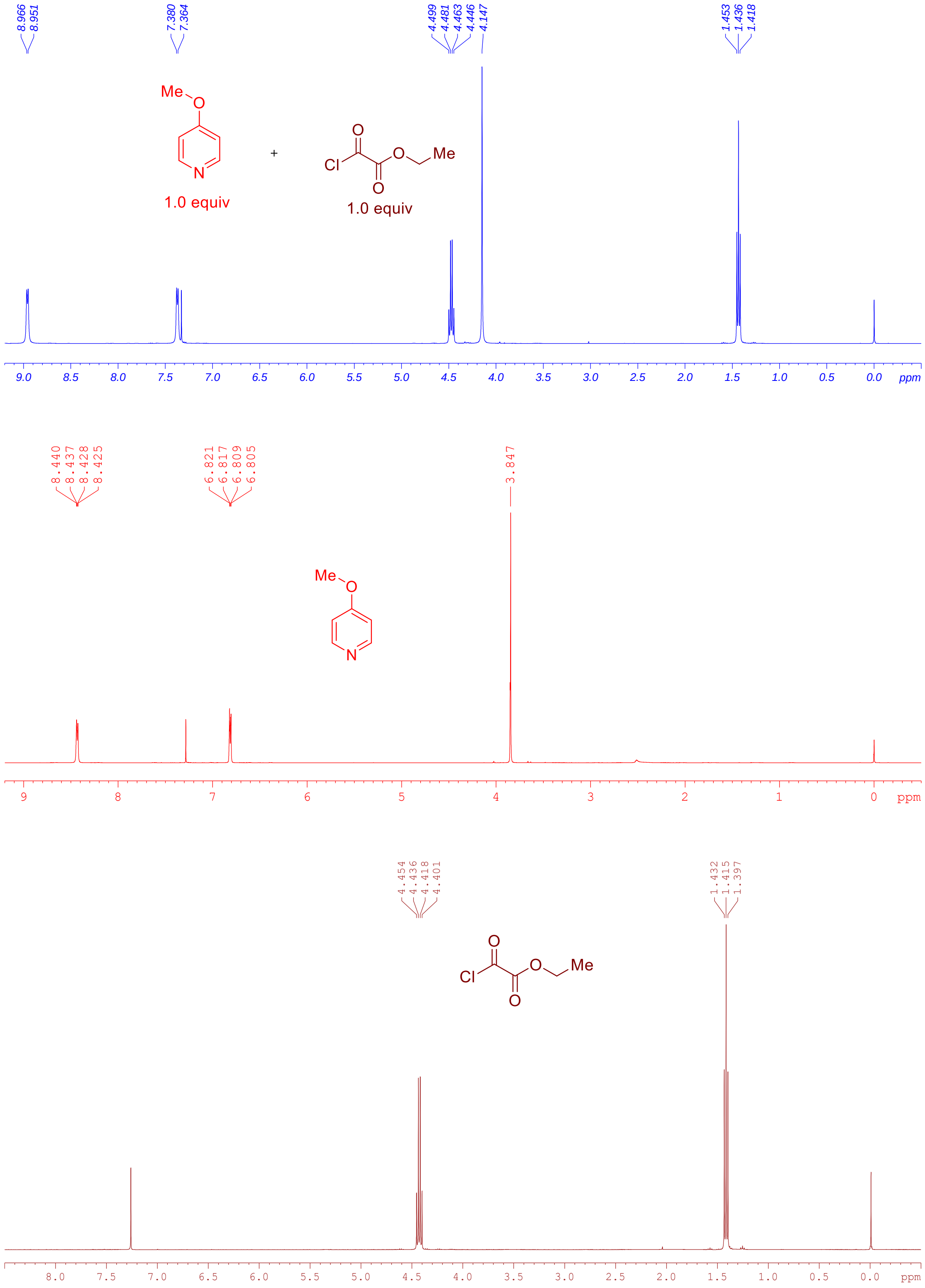

Supplementary Figure 2. ^{1}H NMR study of ethyl chlorooxoacetate **2a** and 4-methoxypyridine mixture

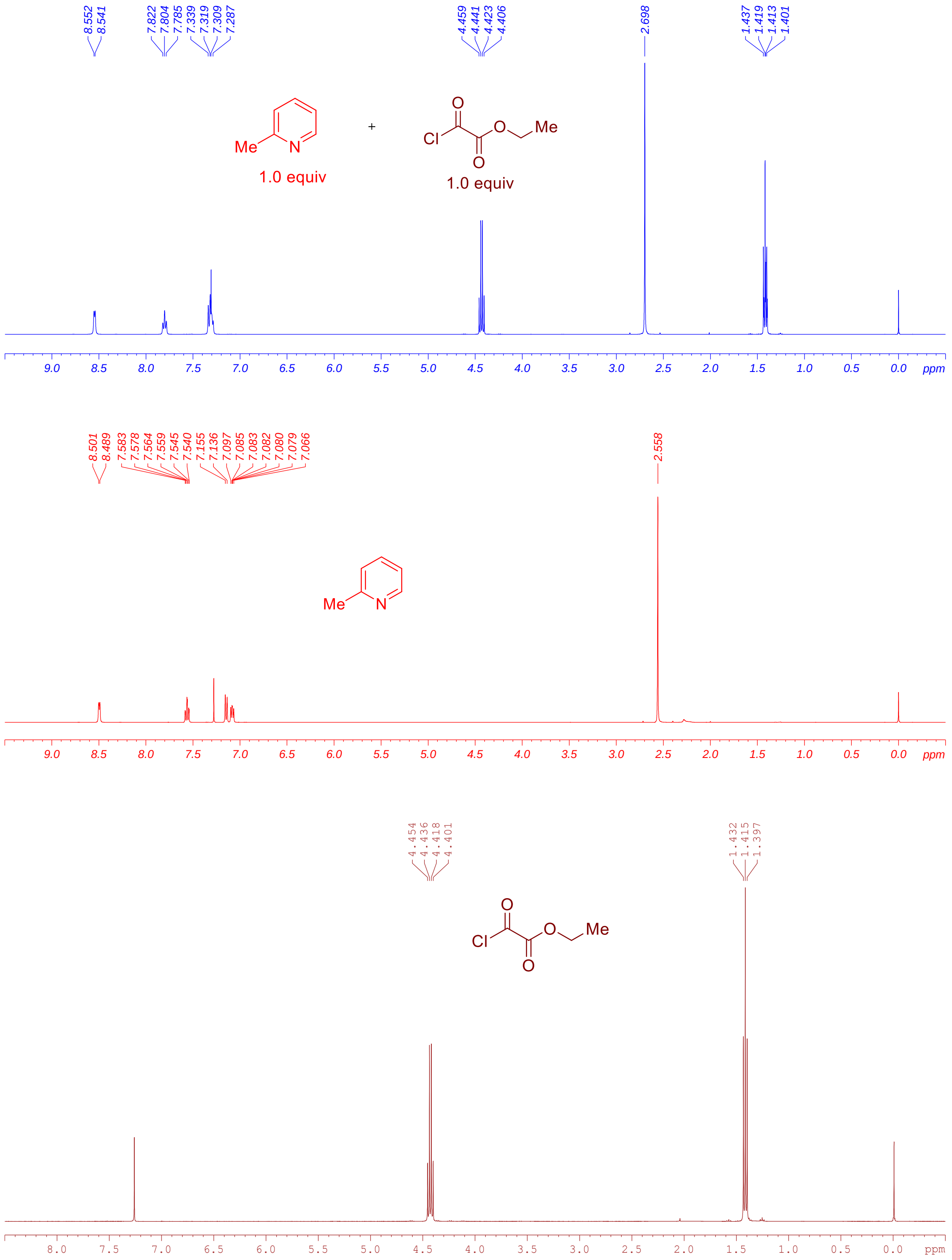

Supplementary Figure 3. ^{1}H NMR study of ethyl chlorooxoacetate **2a** and 2-methylpyridine mixture

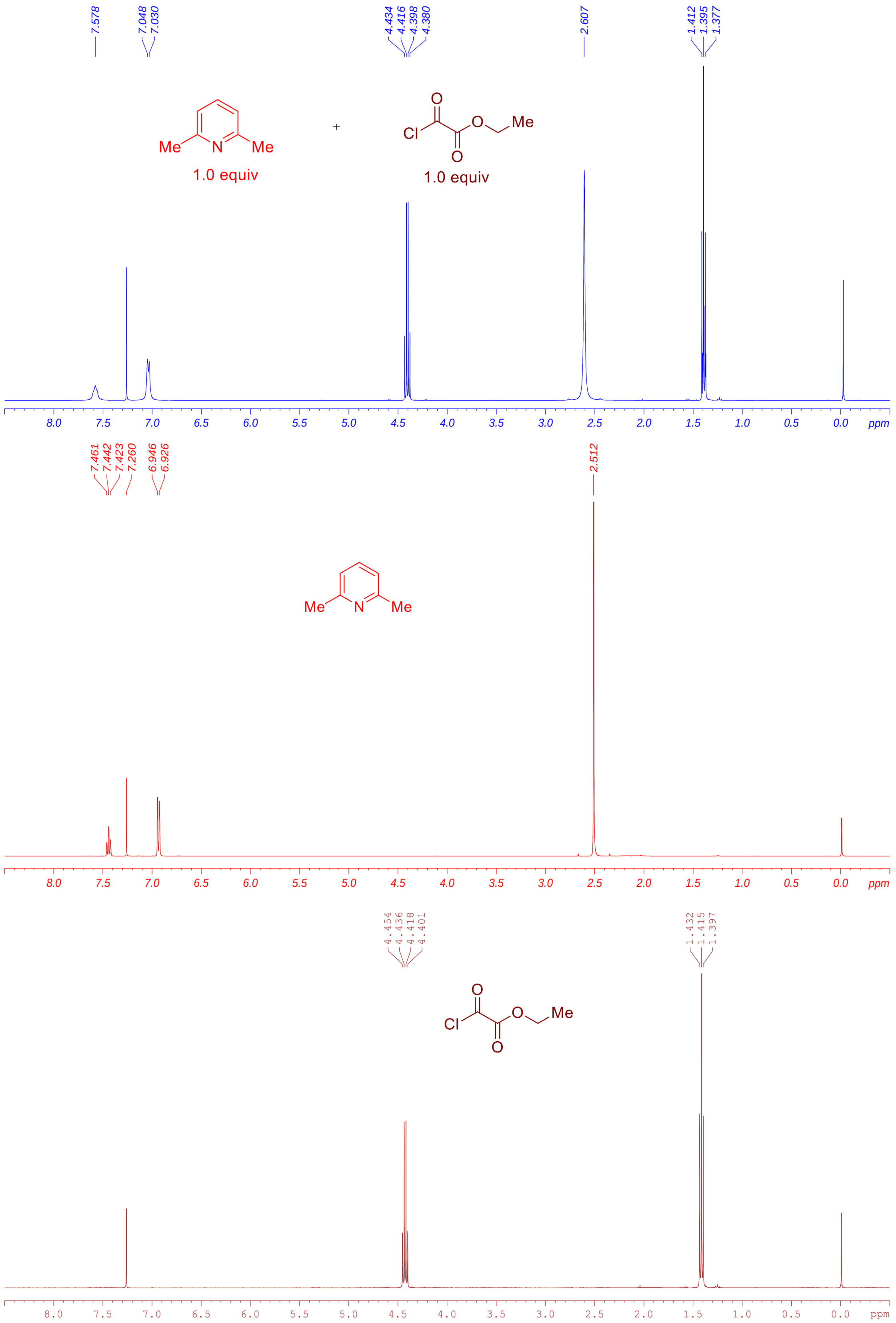

Supplementary Figure 4. ^{1}H NMR study of ethyl chlorooxoacetate **2a** and 2,6-lutidine mixture

(c) Cyclic Voltammetry Studies

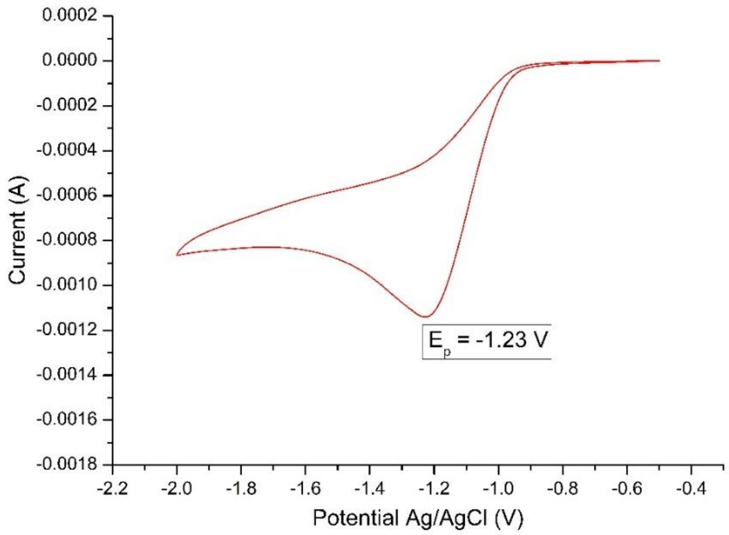

Supplementary Figure 5. Cyclic voltammogram of ethyl chlorooxoacetate **2a** [0.02 M] in [0.1 M] $TBAPF_6$ in CH_3CN. Sweep rate: 200 mV/s. Glassy carbon working electrode, Ag/AgCl (satd. KCl) reference electrode, Pt wire auxiliary electrode. Irreversible reduction. E_p = -1.23 V.

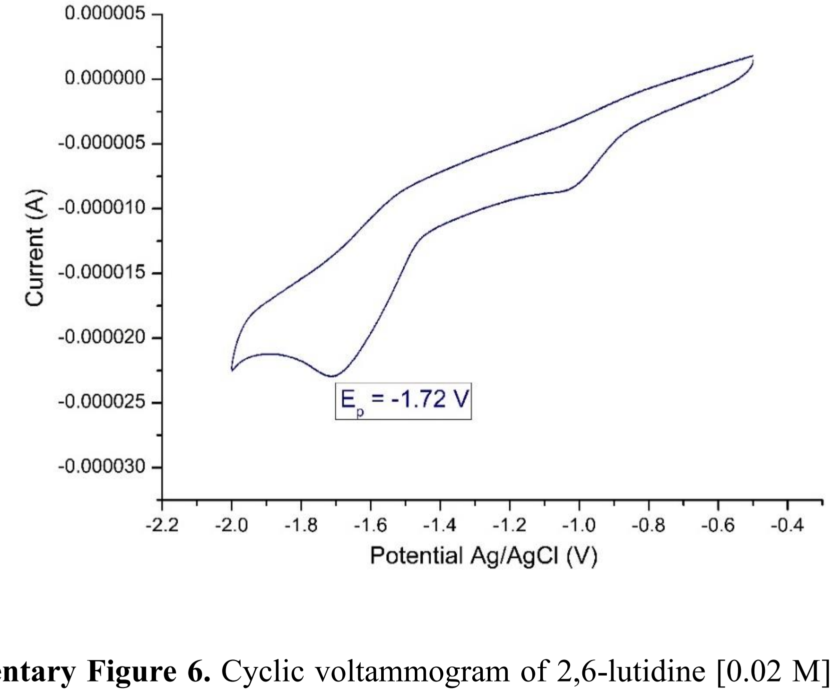

Supplementary Figure 6. Cyclic voltammogram of 2,6-lutidine [0.02 M] in [0.1 M] $TBAPF_6$ in CH_3CN. Sweep rate: 200 mV/s. Glassy carbon working electrode, Ag/AgCl (satd. KCl) reference electrode, Pt wire auxiliary electrode. Irreversible reduction. E_p = -1.72 V.

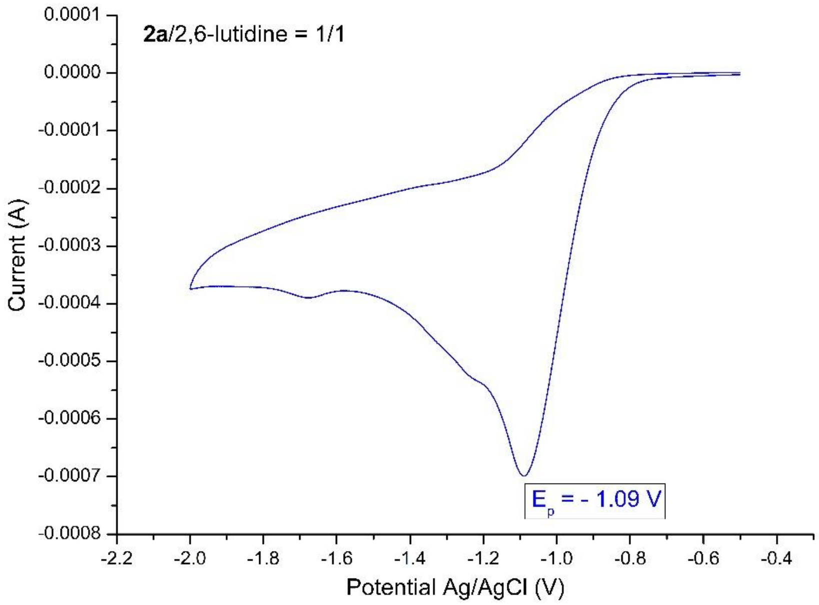

Supplementary Figure 7. Cyclic voltammogram of ethyl chlorooxoacetate **2a** [0.02 M] and 2,6-lutidine [0.02 M] in [0.1 M] $TBAPF_6$ in CH_3CN. Sweep rate: 200 mV/s. Glassy carbon working electrode, Ag/AgCl (satd. KCl) reference electrode, Pt wire auxiliary electrode. Irreversible reduction. E_p = -1.09 V;

(c) Stern-Volmer fluorescence quenching experiments

Stern-Volmer fluorescence quenching experiments were run with freshly prepared solutions of 0.1 mM Ir(ppy)$_3$ in degassed dry CH_3CN added with the appropriate amount of a quencher in a screw-top quartz cuvette at room temperature. The solutions were irradiated at 395 nm and fluorescence was measured from 450 nm to 650 nm.

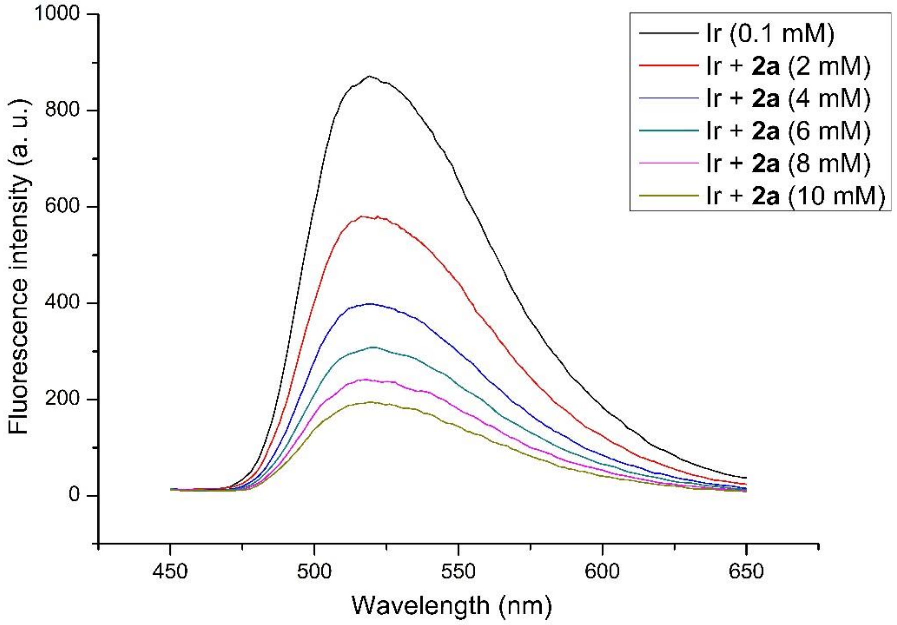

Supplementary Figure 8. Fluorescence quenching experiments of Ir(ppy)$_3$ and **2a**.

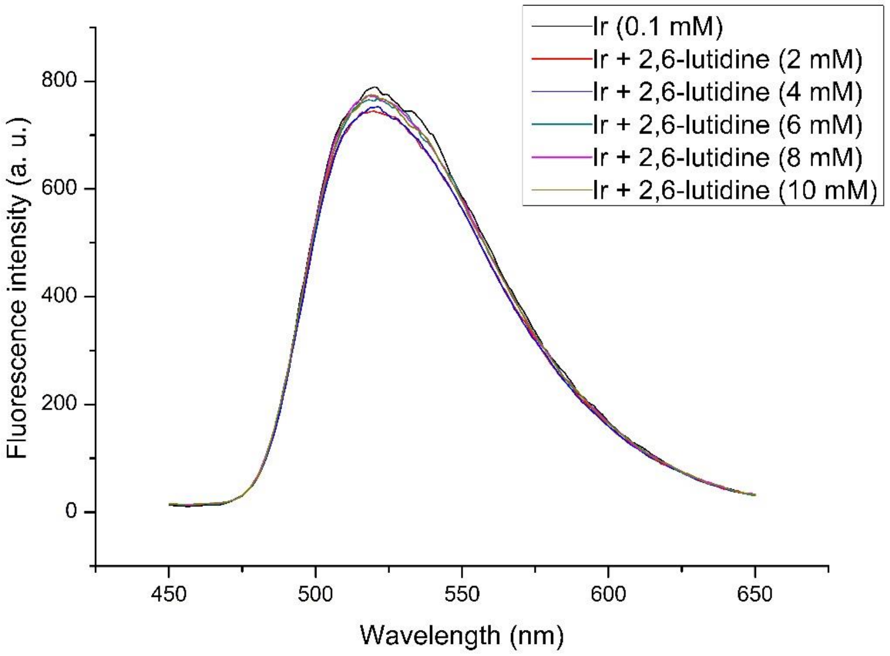

Supplementary Figure 9. Fluorescence quenching experiments of Ir(ppy)$_3$ and 2,6-lutidine.

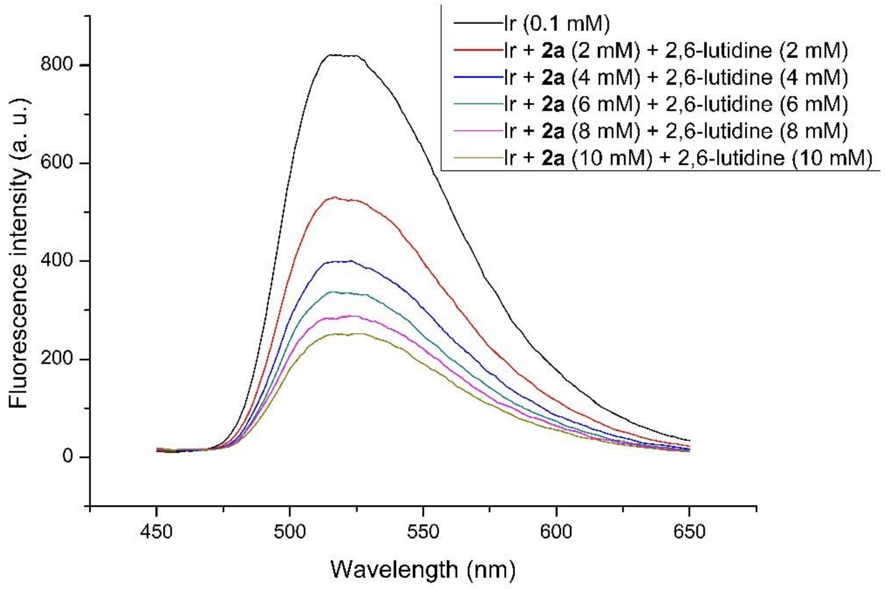

Supplementary Figure 10. Fluorescence quenching experiments of Ir(ppy)$_3$ and **2a** + 2,6-lutidine .

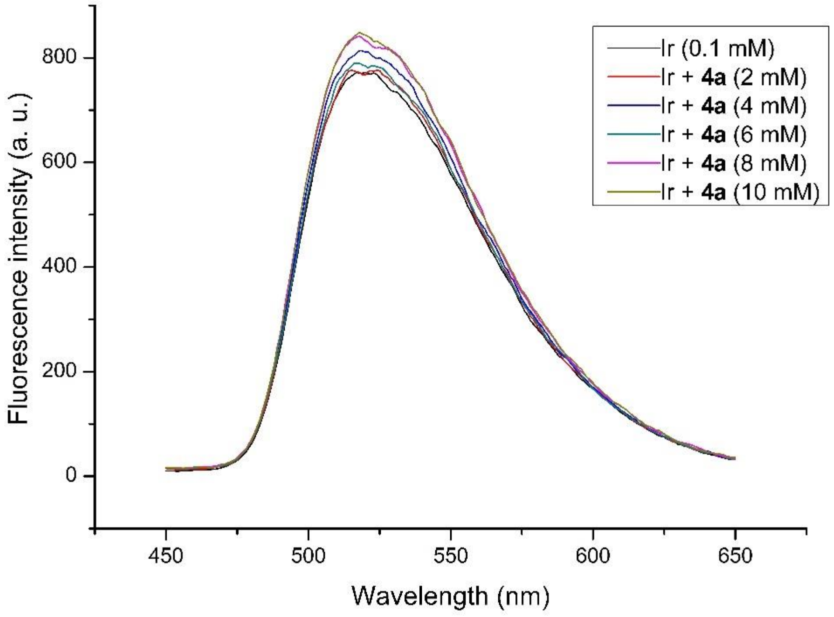

Supplementary Figure 11. Fluorescence quenching experiments of $Ir(ppy)_3$ and **4a**.

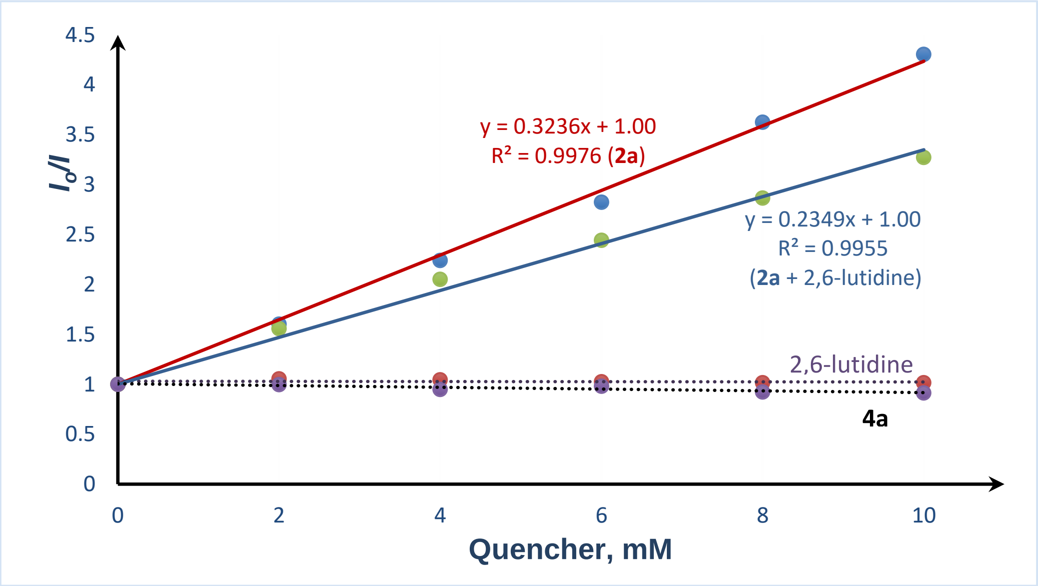

Supplementary Figure 12. Stern-Volmer plots of $Ir(ppy)_3$ with different quenchers.

Conclusion: Neither 2,6-lutidine nor but-3-en-1-ylbenzene **4a** quenched the excited $*Ir(ppy)_3$. Ethyl chlorooxoacetate **2a** quenched the excited $*Ir(ppy)_3$. Additionally, the quenching efficiency of ethyl chlorooxoacetate **2a** was weakened in the presence of 2,6-lutidine.

Stern-Volmer fluorescence quenching experiments were run with freshly prepared solutions of 0.1 mM Ru(bpy)$_3$Cl$_2$ in degassed dry CH_3CN added the appropriate amount of a quencher in a screw-top quartz cuvette at room temperature. The solutions were irradiated at 452 nm and fluorescence was measured from 500 nm to 750 nm.

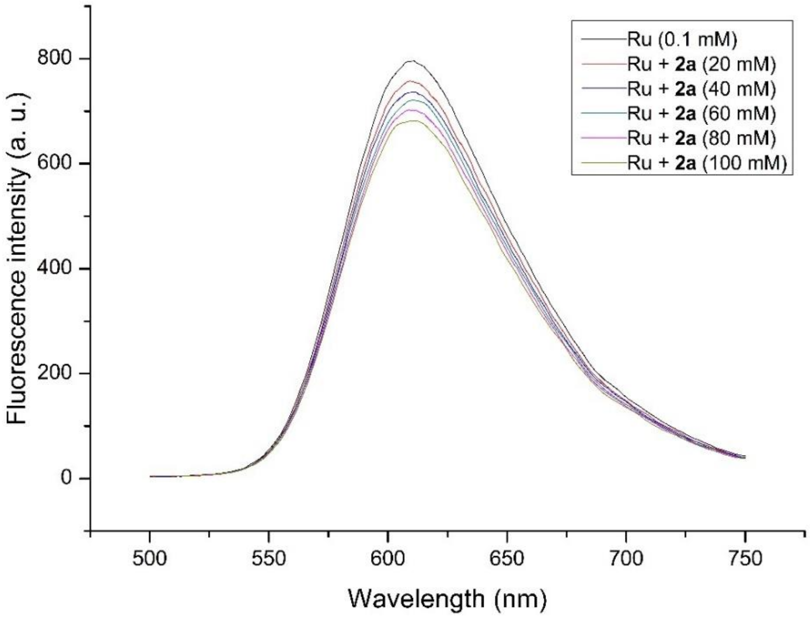

Supplementary Figure 13. Fluorescence quenching experiments of Ru(bpy)$_3$ and **2a**.

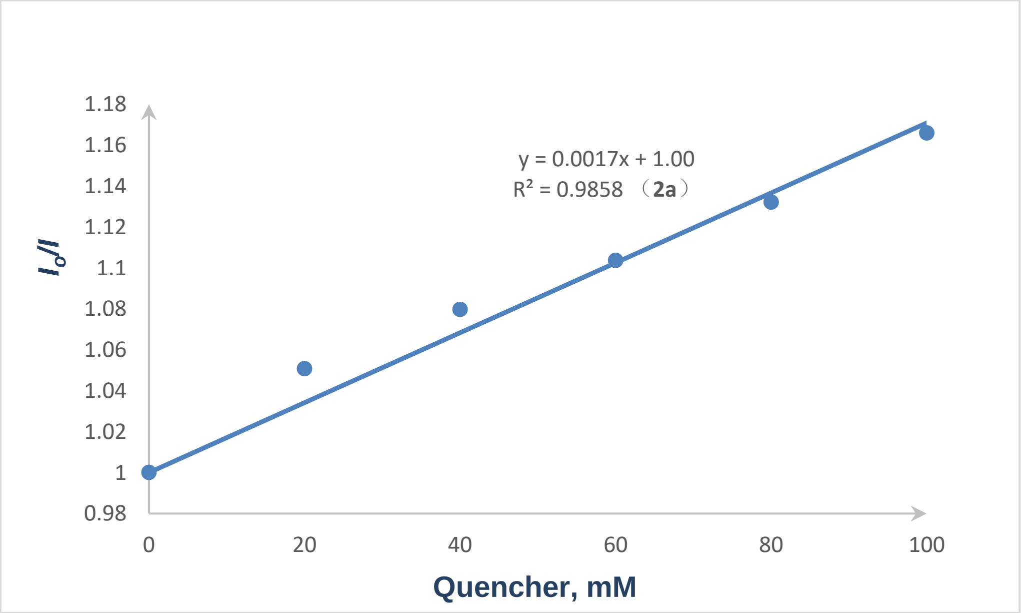

Supplementary Figure 14. Stern-Volmer plots of Ru(bpy)$_3$ with **2a**.

(d) Measurement of quantum yield

According to the procedure of Yoon, [8] the photon flux of the LED was determined by standard ferrioxalate actinometry. A 0.15 M solution of ferrioxalate was prepared by dissolving potassium ferrioxalate hydrate (2.21 g) in 30 mL of H_2SO_4 (0.05 M) solution. A buffered solution of 1,10-phenanthroline was prepared by dissolving 1,10-phenanthroline (50 mg) and sodium acetate (11.25 g) in 50 mL of H_2SO_4 (0.5 M) solution. Both solutions were stored in the dark. To determine the photon flux of the LEDs, the ferrioxalate solution (2.0 mL) was placed in a cuvette and irradiated for 90 s at λ_{max} = 420 nm. After irradiation, the phenanthroline solution (0.35 mL) was added to the cuvette, and the mixture was allowed to stir in the dark for 1 h to allow the ferrous ions to completely coordinate with phenanthroline. The absorbance of solution was measured at 510 nm. A non-irradiated sample was also prepared and the absorbance at 510 nm was measured. Conversion was calculated using eq. 1.

$$\mathrm{n}\,Fe^{2+} = \frac{V \times \Delta A}{l \times \varepsilon} \qquad (1)$$

where V is the total volume (0.00235L) of the measurement sample, ΔA is the difference in absorbance at 510 nm between the irradiated and non-irradiated solutions [ΔA = 0.477 (irradiated for 90 s at λ_{max} = 420 nm)], l is the optical path of the sample in the spectrophotometer (1 cm), and ε is the extinction coefficient of the complex $Fe^{II}(phen)_3^{2+}$ at 510 nm (11100 L mol^{-1} cm^{-1})

$$\text{photon flux} = \frac{n\,Fe^{2+}}{\Phi \times t \times f} \qquad (2)$$

where Φ is the quantum yield for the ferrioxalate actinometer (1.12 at λ_{ex} = 420 nm), t is the irradiation time (90 s), and f is the fraction of light absorbed at λ_{ex} = 420 nm by the ferrioxalate actinometer. This value is calculated using eq. 3 where A (420 nm) is the absorbance of the ferrioxalate solution at 420 nm.

$$f = 1\text{-}10^{-A\,(420nm)} = 1\text{-}10^{-1.880} = 0.9868 \qquad (3)$$

$$\mathrm{n}\,Fe^{2+} = \frac{V \times \Delta A}{l \times \varepsilon} = \frac{0.00235L \times 0.477}{1cm \times 11100L\,mol^{-1}cm^{-1}} = 1.0099 \times 10^{-7}\ \mathrm{mol}$$

$$photon\ flux = \frac{n\,Fe^{2+}}{\Phi \times t \times f} = \frac{1.0099\times10^{-7}}{1.12\times90\times0.9868} = 1.015\times10^{-9}\ \text{einstein}\cdot\text{s}^{-1}$$

Determination of the reaction quantum yield at 420 nm and quantum yield measurement was performed in an oven-dried 20 mL quartz vial with a magnetic stirring bar. Ethyl chlorooxoacetate **2a** (0.6 mmol) and 2,6-lutidine (0.3 mmol) were added to a solution of 4-vinyl-1,1'-biphenyl **1a** (0.2 mmol) and $Ru(bpy)_3Cl_2$ (2 mol %) in dry CH_3CN (4.0 mL) at room temperature. The mixture was degassed by three cycles of freeze-pump-thaw and then irradiated in Parallel Light Reactor (WP-TEC-1020) for 18000 s (5.0 h). The crude yield of product **3a** was determined by ^{1}H NMR based on a 1,3,5-trimethoxybenzene standard and the final yield was 10% (2.0 x 10^{-5} mol). The reaction quantum yield (Φ) was determined using eq. 4.

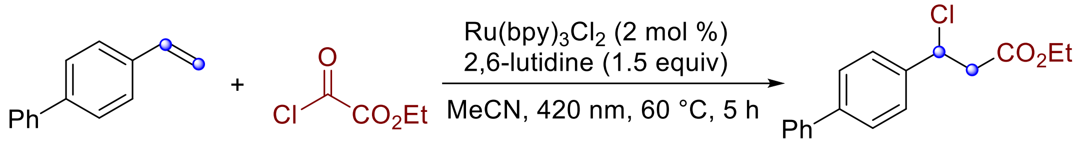

1a **2a** **3a,** 10% yield

$$\Phi\ (\mathbf{3a}) = \frac{Mol\ product}{flux*t*f} = \frac{2.0\times10^{-5}}{1.015\times10^{-9}\times18000\times0.9868} = 1.11 \qquad (4)$$

Determination of the reaction quantum yield at 420 nm and quantum yield measurement was performed in an oven-dried 20 mL quartz vial with a magnetic stirring bar. Ethyl chlorooxoacetate **2a** (1.6 mmol) and 2,6-lutidine (0.4 mmol) were added to a solution of 1-octene **4c** (0.2 mmol) and $Ir(ppy)_3$ (5 mol %) in dry CH_3CN (4.0 mL) at room temperature. The mixture was degassed by three cycles of freeze-pump-thaw and then irradiated in Parallel Light Reactor (WP-TEC-1020) for 18000 s (5.0 h). The crude yield of the product **5c** was determined by ^{1}H NMR based on a 1,3,5- trimethoxybenzene standard and the final yield was 12% (2.4 x 10^{-5} mol). The reaction quantum yield (Φ) was determined using eq. 5.

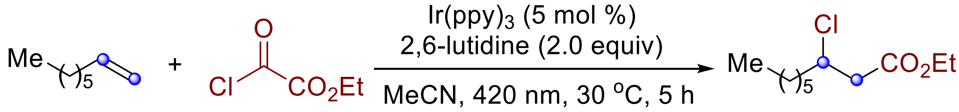

4c **2a** **5c**, 12% yield

$$\Phi\ (\mathbf{5c}) = \frac{Mol\ product}{flux*t*f} = \frac{2.4\times10^{-5}}{1.015\times10^{-9}\times18000\times0.9868} = 1.33 \qquad (5)$$

Determination of the reaction quantum yield at 420 nm and quantum yield measurement was performed in an oven-dried 20 mL quartz vial with a magnetic stirring bar. Ethyl chlorooxoacetate **2a** (0.6 mmol) and 2,6-lutidine (0.4 mmol) were added to a solution of *N*-methyl-*N*-phenylmethacrylamide **7a** (0.2 mmol) and Ir(ppy)$_3$ (2 mol%) in dry DMF (4.0 mL) at room temperature. The mixture was degassed by three cycles of freeze-pump-thaw and then irradiated in Parallel Light Reactor (WP-TEC-1020) for 7200 s (2.0 h). The crude yield of the product **8a** was determined by ^{1}H NMR based on a 1,3,5- trimethoxybenzene standard and the final yield was 36% (7.2 x 10^{-5} mol). The reaction quantum yield (Φ) was determined using eq. 6.

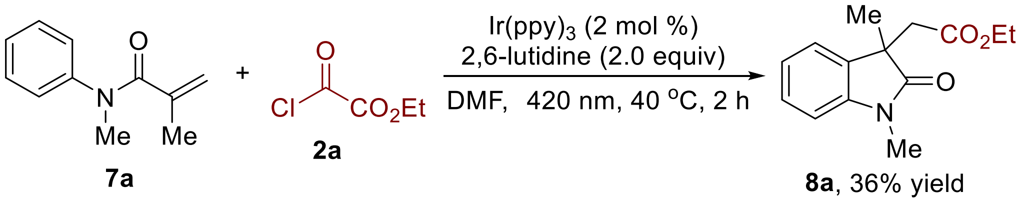

$$\Phi\,(\mathbf{8a}) = \frac{Mol\ product}{flux*t*f} = \frac{7.2\times10^{-5}}{1.015\times10^{-9}\times7200\times0.9868} = 9.98 \qquad (6)$$

(e) Computational details

To shed light on the mechanism of the title reaction, density functional theory (DFT) calculations were then carried out with the Gaussian 09 software package.[9-12] The geometry optimization and frequency calculations were carried out with M062X method and 6-31+G(d) basis set.[13] Truhlar and coworkers' SMD solvation model was employed to consider the solvent effect of acetonitrile (ε = 35.688).[14] Reaction Gibbs free energies of elementary steps involving single electron transfer (SET) were calculated from DFT reaction energies of redox half-reactions for the organic species and the experimental redox potentials of the Ir photoredox catalyst. The Gibbs free energy of the electron was taken to be -0.867 kcal/mol.[15,16]

Free-energy reaction profiles (kcal/mol) calculated with the SMD (acetonitrile) M062X/6-31+G(d) level at 303 K.

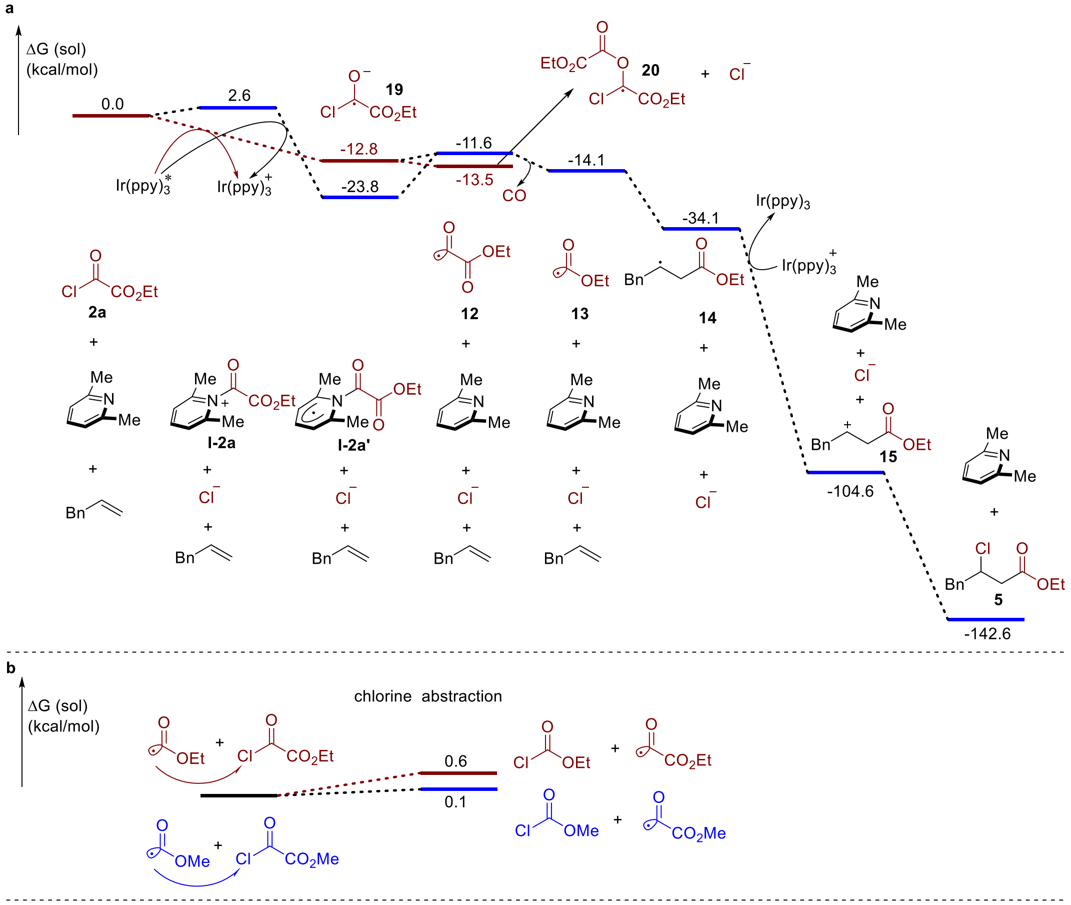

Supplementary Figure 15. Propose reaction pathway and related free energy profile

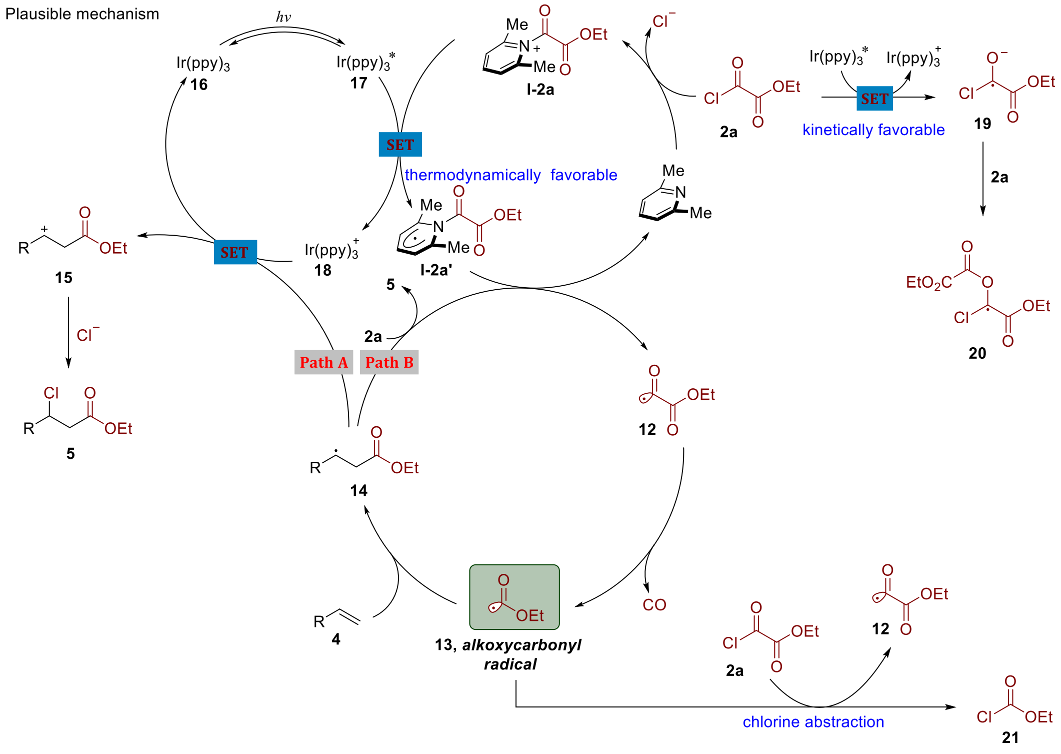

Supplementary Figure 16. Plausible mechanism

In order to shed more light on the reaction mechanism, especially the role of 2,6-lutidine, DFT calculations were carried out with the Gaussian 09 software package. The calculation details were provided in the Supporting Information. As shown in Figure 15, the whole reaction was thermodynamically favorable and the driving force for this transformation was the extrusion of carbon monoxide, the generation of stable alkoxycarbonyl radical **13** and subsequent reaction with alkene to afford alkyl radical **14**. Concerning the formation of key intermediate **12**, though the generation of acyl pyridinium salt **I-2a** was a little bit endothermic, the formation of **I-2a'** *via* reduction of **I-2a** by highly reducing species *Ir(ppy)$_3$ was more thermodynamically favored compared to the direct formation of **19** without partition of 2,6-lutidine. Additionally, anion radical **19** could react with compound **2a** to deliver the undesired alkyl radical **20**. Thus 2,6-lutidine might facilitate the transformation relative to no participation of 2,6-lutidine. Moreover, Stern-Volmer experiments have demonstrated that the step to generate anion radical **19** is kinetically favorable. Hence, raising the equivalent of 2,6-lutidine would facilitate the generation of the **I-2a'** and suppress the formation of anion radical **19**.

DFT calculations were also applied to study the side reaction of ethoxycarbonyl radical and ethyl chlorooxoacetate to generate radical **12** and ethyl carbonochloridate. The reaction free energy was 0.6 kcal/mol, while the reaction free energy of methoxycarbonyl radical and methyloxalyl chloride was only 0.1 kcal/mol. According to Arrhenius equation, the side reaction of the latter was more than the former. This might contribute to the low yield of methyloxalyl chloride compared to ethyloxalyl chloride (**3b** vs **3r**). By raising the equivalent of methyl oxalyl chloride, the yield of **3r** was increased from 38% to 54%.

Supplementary Table 4. The free energies and coordinates of the optimized structures.

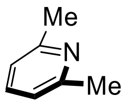

-326.681406 a.u. (zero imaginary freq.)

6	-1.201187	1.120310	-0.000004
6	-0.009054	1.831561	0.000005
6	1.196922	1.131628	0.000002
6	1.163985	-0.262974	-0.000008
7	0.006945	-0.948878	-0.000012
6	-1.152584	-0.280065	-0.000009
6	2.425026	-1.082778	0.000000
6	-2.422815	-1.087981	0.000011
1	-2.161368	1.629006	-0.000004
1	-0.014654	2.918520	0.000011
1	2.148941	1.654062	0.000005
1	3.313185	-0.446043	-0.000132
1	2.458110	-1.730182	0.883165
1	2.457986	-1.730397	-0.883011
1	-2.193426	-2.156049	-0.000193
1	-3.029452	-0.857644	0.882676
1	-3.029692	-0.857340	-0.882407

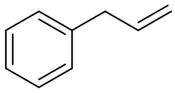

-348.683187 a.u. (zero imaginary freq.)

6	-1.463424	-0.924682	-0.266078
6	-2.376735	-0.108257	0.610682
6	-3.401178	0.618714	0.161440
6	-0.028649	-0.441668	-0.180454
6	0.322707	0.806355	-0.709885
6	1.633360	1.272752	-0.627194
6	2.614768	0.497289	-0.004568
6	2.274579	-0.744686	0.529618
6	0.959930	-1.209242	0.441287
1	-1.501623	-1.976943	0.041293
1	-1.815672	-0.868569	-1.303274
1	-2.149001	-0.119428	1.677895
1	-4.025229	1.200952	0.835049
1	-3.648416	0.652710	-0.898809
1	-0.441227	1.414102	-1.192492
1	1.890879	2.241529	-1.047621
1	3.637005	0.859827	0.061499
1	3.031386	-1.355497	1.015105
1	0.698910	-2.180682	0.856482

2a 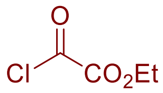

-841.081435 a.u. (zero imaginary freq.)

17	2.708981	-0.235043	0.000564
6	1.145867	0.581567	-0.000008
6	-0.050493	-0.400860	-0.000269
8	1.049188	1.762118	-0.000205
8	0.087392	-1.595796	-0.000579
8	-1.174089	0.276858	-0.000069
6	-3.554129	0.453594	0.000188
6	-2.396551	-0.513283	-0.000066
1	-4.488844	-0.115409	0.000230
1	-3.535778	1.087492	0.891394
1	-3.535987	1.087718	-0.890861
1	-2.384900	-1.146808	-0.890711
1	-2.384729	-1.147055	0.890404

I-2a 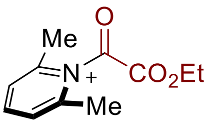

-707.403582 a.u. (zero imaginary freq.)

6	-2.830305	-1.502907	0.130714
6	-3.671188	-0.466945	0.524979
6	-3.214372	0.846932	0.510758
6	-1.921586	1.127563	0.102463
7	-1.130007	0.077354	-0.266800
6	-1.534082	-1.227169	-0.268324
6	0.246196	0.373451	-0.703946
6	1.313041	-0.012159	0.341837
8	0.458808	0.899302	-1.746941
8	1.003702	-0.360194	1.454388
8	2.512203	0.122390	-0.160497
6	4.895374	0.030679	-0.045075
6	3.622766	-0.184359	0.734314
6	-1.367142	2.516360	0.054252
6	-0.571156	-2.286031	-0.706150

1	-3.165880	-2.533676	0.122909
1	-4.687174	-0.683973	0.840248
1	-3.849907	1.671165	0.813943
1	5.747979	-0.198711	0.601027
1	4.936525	-0.627380	-0.917758
1	4.981016	1.069726	-0.375714
1	3.543960	0.481096	1.597729
1	3.504818	-1.219533	1.065072
1	-2.067032	3.190060	0.550006
1	-0.399720	2.585029	0.564150
1	-1.238192	2.848454	-0.980873
1	-0.043649	-2.008688	-1.624506
1	0.166876	-2.491236	0.077420
1	-1.122550	-3.208279	-0.896577

I-2a'

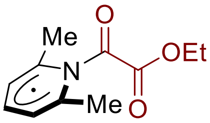

-707.546204 a.u. (zero imaginary freq.)

6	2.558727	-1.227633	-1.231203
6	3.392998	-0.117689	-1.289015
6	2.967514	1.088096	-0.743553
6	1.715357	1.175642	-0.151006
7	0.924632	0.068268	-0.109909
6	1.311980	-1.129326	-0.626955
6	-0.386618	0.174778	0.592277
6	-1.499580	0.181287	-0.315458
8	-0.366883	0.300577	1.837525
8	-1.412289	0.103026	-1.549701
8	-2.690100	0.277690	0.328532
6	-5.063605	0.363748	0.389771
6	-3.850184	0.312826	-0.511797
6	1.194313	2.452662	0.427179
6	0.389629	-2.302878	-0.522010
1	2.864974	-2.184083	-1.640661
1	4.370701	-0.193106	-1.755435
1	3.591174	1.974580	-0.776690
1	-5.972489	0.391282	-0.220081
1	-5.107104	-0.520295	1.033831
1	-5.045404	1.257445	1.021481
1	-3.798008	1.193279	-1.161529
1	-3.862889	-0.578687	-1.147019

1	1.871372	3.269074	0.171242
1	0.195893	2.673731	0.034183
1	1.112055	2.380144	1.515663
1	0.016344	-2.421949	0.499536
1	-0.470319	-2.171935	-1.187320
1	0.926348	-3.207576	-0.813434

Cl⁻

-460.355147 a.u. (zero imaginary freq.)

17	0.000000	0.000000	0.000000

12

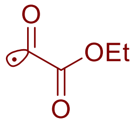

-380.845332 a.u. (zero imaginary freq.)

6	1.819835	-0.794098	0.000394
6	0.633913	0.234927	0.000607
8	2.950261	-0.471816	-0.001087
8	0.811508	1.423971	0.000087
8	-0.507679	-0.410650	0.000445
6	-2.892034	-0.530663	-0.000124
6	-1.711233	0.408010	0.000066
1	-3.813162	0.060195	-0.000402
1	-2.888611	-1.165004	-0.891157
1	-2.889037	-1.164830	0.891034
1	-1.685339	1.041205	0.890699
1	-1.684912	1.041022	-0.890685

13

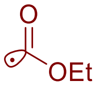

-267.56271 a.u. (zero imaginary freq.)

6	1.341967	0.300430	-0.000012
8	2.421692	-0.200043	0.000004
8	0.177546	-0.302773	0.000041
6	-2.212925	-0.323753	-0.000042
6	-0.997497	0.568462	0.000007
1	-3.110927	0.301823	-0.000056
1	-2.233602	-0.957554	-0.891409
1	-2.233651	-0.957583	0.891304
1	-0.949071	1.195277	0.893404

1	-0.949011	1.195298	-0.893372

CO

-113.286497 a.u. (zero imaginary freq.)

6	0.000000	0.000000	-0.646257
8	0.000000	0.000000	0.484693

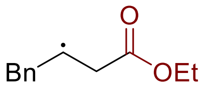

-616.277858 a.u. (zero imaginary freq.)

6	-1.194882	-1.194608	-0.453823
6	-0.033094	-0.469683	0.145801
6	1.347574	-0.835291	-0.273829
6	2.416102	0.066642	0.297640
8	2.236145	0.936351	1.123551
6	-2.511665	-0.477952	-0.248431
6	-2.692182	0.816586	-0.753226
6	-3.898441	1.491196	-0.574236
6	-4.947308	0.879833	0.117758
6	-4.777732	-0.407253	0.625973
6	-3.566962	-1.079749	0.442481
8	3.618156	-0.220209	-0.213852
6	5.974034	0.055970	-0.421321
6	4.730186	0.557161	0.275244
1	-1.014484	-1.332443	-1.530443
1	-1.272707	-2.211949	-0.033775
1	-0.168826	0.061129	1.083844
1	1.445986	-0.834364	-1.368156
1	1.606738	-1.864664	0.032892
1	-1.876909	1.296000	-1.292828
1	-4.022083	2.493375	-0.976529
1	-5.888747	1.403995	0.258521
1	-5.587040	-0.891202	1.166449
1	-3.440185	-2.083843	0.842142
1	6.840350	0.627305	-0.073576
1	6.147316	-1.001570	-0.199504
1	5.891885	0.180489	-1.505497
1	4.539587	1.612231	0.058025
1	4.792764	0.430543	1.360165

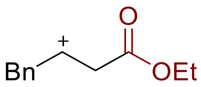

-616.097079 a.u. (zero imaginary freq.)

6	6.553856	-4.076493	-2.397779
6	5.618432	-3.358912	-1.583457
6	4.212903	-3.249533	-1.904787
6	3.522568	-2.052172	-1.274260
8	3.956353	-1.460997	-0.312413
6	7.820305	-4.569797	-1.749025
6	7.767021	-5.229806	-0.517297
6	8.937005	-5.711121	0.069291
6	10.162983	-5.540459	-0.573532
6	10.217554	-4.886567	-1.805540
6	9.049962	-4.401273	-2.391373
8	2.393642	-1.773971	-1.901857
6	0.398936	-0.510893	-2.246761
6	1.620381	-0.664810	-1.373623
1	6.064509	-4.787258	-3.075693
1	6.784054	-3.197898	-3.066701
1	5.981719	-2.840551	-0.692128
1	4.000143	-3.393878	-2.968945
1	3.805515	-4.162163	-1.406026
1	6.810290	-5.376851	-0.019371
1	8.888990	-6.221684	1.026951
1	11.073224	-5.918635	-0.116707
1	11.170051	-4.751285	-2.310194
1	9.090112	-3.889191	-3.349864
1	-0.203612	0.322343	-1.872526
1	0.682024	-0.294441	-3.281191
1	-0.214841	-1.416252	-2.226786
1	1.360881	-0.897503	-0.337326
1	2.253348	0.226425	-1.389987

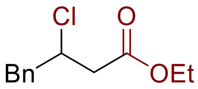

-1076.512706 a.u. (zero imaginary freq.)

6	-1.002181	1.090032	-0.836405
6	-0.066466	1.367663	0.342289
6	0.607545	0.153030	0.976984
6	1.557758	-0.563671	0.044826
8	1.243995	-1.039039	-1.027640
17	1.227180	2.535850	-0.205202

6	-2.169852	0.226763	-0.414774
6	-3.262949	0.794625	0.250305
6	-4.335277	0.003874	0.663361
6	-4.328029	-1.369563	0.413822
6	-3.243157	-1.944817	-0.249048
6	-2.170906	-1.150916	-0.658661
8	2.793819	-0.627913	0.541312
6	5.098949	-1.215203	0.465455
6	3.781946	-1.298135	-0.270167
1	-1.371459	2.053737	-1.202428
1	-0.443402	0.608621	-1.643161
1	-0.608548	1.903229	1.124605
1	1.139050	0.457957	1.880389
1	-0.177538	-0.556491	1.266685
1	-3.274870	1.866441	0.440543
1	-5.178395	0.461433	1.174176
1	-5.164892	-1.986446	0.729915
1	-3.231843	-3.012763	-0.450793
1	-1.322460	-1.600116	-1.170290
1	5.874696	-1.719327	-0.119082
1	5.399347	-0.173061	0.611496
1	5.031469	-1.703660	1.442409
1	3.462332	-2.333460	-0.421883
1	3.825809	-0.800284	-1.243189

19

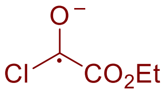

-841.202338 a.u. (zero imaginary freq.)

17	2.734467	-0.276261	-0.000382
6	1.084100	0.581632	0.000046
6	-0.006130	-0.347314	-0.000212
8	1.086877	1.815502	0.000480
8	0.052902	-1.586694	-0.000654
8	-1.206113	0.320627	0.000098
6	-3.578092	0.401651	0.000280
6	-2.368787	-0.508628	-0.000121
1	-4.492908	-0.200288	0.000130
1	-3.585964	1.040129	0.889547
1	-3.586084	1.040753	-0.888537
1	-2.359158	-1.153067	-0.886287
1	-2.359042	-1.153694	0.885588

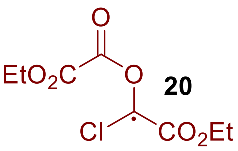

20

-1221.929811 a.u. (zero imaginary freq.)

17	3.155570	-1.491415	0.150895
6	1.779449	-0.516024	0.217153
6	1.862753	0.935269	0.059253
8	0.607483	-1.138829	0.502747
8	2.903443	1.519418	-0.166905
6	-0.354514	-1.152458	-0.464054
8	-0.211023	-0.753576	-1.580368
8	0.664118	1.501223	0.196623
6	-0.836038	3.349845	0.057722
6	0.617425	2.940968	0.036362
6	-1.612685	-1.858524	0.061457
8	-1.540718	-3.016214	0.385788
8	-2.736253	-1.173322	0.061297
6	-3.967377	0.841370	0.424705
6	-2.723813	0.262287	-0.202610
1	-0.900698	4.438280	-0.035772
1	-1.380035	2.899943	-0.778893
1	-1.314629	3.056248	0.997375
1	1.184653	3.390969	0.856100
1	1.099837	3.195890	-0.910758
1	-3.996523	1.917450	0.227268
1	-4.866151	0.388147	-0.003389
1	-3.965759	0.683640	1.507016
1	-1.821763	0.693632	0.241953
1	-2.705601	0.402362	-1.285946

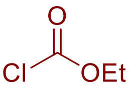

-727.797928 a.u. (zero imaginary freq.)

8	2.396396	-0.388310	-0.000036
8	0.190280	-0.203855	0.000016
6	-2.183950	-0.329631	-0.000034
6	-1.012032	0.620849	-0.000019
1	-3.108292	0.255913	-0.000053
1	-2.175318	-0.962923	-0.891585
1	-2.175351	-0.962911	0.891527
1	-0.996711	1.247156	0.894256
1	-0.996676	1.247137	-0.894304

6	1.407169	0.278764	-0.000019
17	1.532762	2.056932	-0.000022

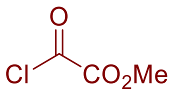

-801.807395 a.u. (zero imaginary freq.)

17	2.709444	-0.228849	-0.000247
6	1.146237	0.582930	0.000173
6	-0.046527	-0.402575	-0.000127
8	1.045265	1.763238	0.000383
8	0.091035	-1.596778	-0.000167
8	-1.173728	0.272267	-0.000077
6	-2.376771	-0.525351	-0.000106
1	-2.404121	-1.147039	-0.897025
1	-2.404130	-1.147033	0.896819
1	-3.196421	0.190163	-0.000111

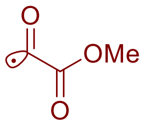

-341.571762 a.u. (zero imaginary freq.)

6	1.819349	-0.796543	0.000170
6	0.636576	0.233970	0.000065
8	2.950030	-0.475684	-0.000202
8	0.813390	1.422331	0.000072
8	-0.508818	-0.408369	0.000038
6	-1.690885	0.420500	-0.000011
1	-1.701680	1.042825	0.896911
1	-1.701632	1.042795	-0.896955
1	-2.529586	-0.272362	-0.000022

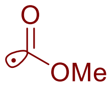

-228.288709 a.u. (zero imaginary freq.)

6	1.345247	0.295702	-0.000022
8	2.417064	-0.219383	-0.000006
8	0.172165	-0.295396	0.000046
6	-0.977863	0.586376	0.000018
1	-0.965612	1.203265	0.899598

1	-0.965570	1.203273	-0.899555
1	-1.845373	-0.071383	-0.000004

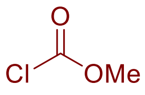

-688.524108 a.u. (zero imaginary freq.)

8	2.421865	-0.367311	-0.000034
8	0.215710	-0.203555	0.000038
6	-0.977488	0.610263	0.000007
1	-1.012875	1.223810	0.900989
1	-1.012890	1.223722	-0.901033
6	1.428846	0.292867	-0.000046
17	1.535689	2.070551	-0.000036
1	-1.795043	-0.108356	0.000048

Characterization of products

ethyl 3-([1,1'-biphenyl]-4-yl)-3-chloropropanoate (3a)

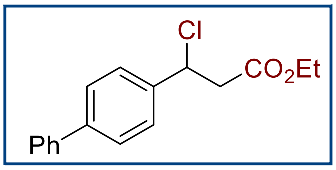

Purification by flash chromatography (PE/EA = 20/1) afforded **3a.** Colorless oil; 46.8 mg, 81% yield; **^{1}H NMR (400 MHz, $CDCl_3$)** δ (ppm) = 1.24 (t, *J* = 7.1 Hz, 3H), 3.06 (dd, *J* = 15.9, 5.8 Hz, 1H), 3.21 (dd, *J* = 15.9, 9.1 Hz, 1H), 4.11−4.23 (m, 2H), 5.40 (dd, *J* = 9.1, 5.8 Hz, 1H), 7.35 (t, *J* = 7.3 Hz, 1H), 7.41−7.49 (m, 4H), 7.56−7.60 (m, 4H); **^{13}C NMR (100 MHz, $CDCl_3$)** δ (ppm) = 14.1, 44.8, 57.9, 61.0, 127.1, 127.3, 127.5, 127.6, 128.8, 139.2, 140.3, 141.6, 169.5; HRMS (ESI) for $C_{17}H_{17}ClO_2Na$ $[M+Na]^+$ calcd. 311.0809, found 275.1046 (-HCl). Benzyl chloride moiety is unstable.[5-7]

ethyl 3-chloro-3-phenylpropanoate (3b)

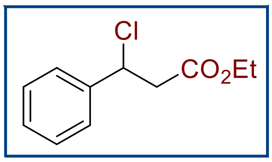

Purification by flash chromatography (PE/EA = 20/1) afforded **3b**. Colorless oil; 27.6 mg, 65% yield; **^{1}H NMR (400 MHz, $CDCl_3$)** δ (ppm) = 1.23 (t, *J* = 7.1 Hz, 3H), 3.02 (dd, *J* = 15.9, 5.8 Hz, 1H), 3.17 (dd, *J* = 15.9, 9.0 Hz, 1H), 4.10−4.22 (m, 2H), 5.34 (dd, *J* = 9.0, 5.8 Hz, 1H), 7.29−7.43 (m, 5H); **^{13}C NMR (100 MHz, $CDCl_3$)** δ (ppm) = 14.1, 44.9, 58.1, 61.0, 126.9, 128.7, 128.7, 140.3, 169.5; HRMS (ESI) for $C_{11}H_{13}ClO_2Na$ $[M+Na]^+$ calcd. 235.0496, found 231.1001 (-Cl, +OMe). All analytical data are consistent with those reported in the literature.[4]

ethyl 3-chloro-3-(*p*-tolyl)propanoate (3c)

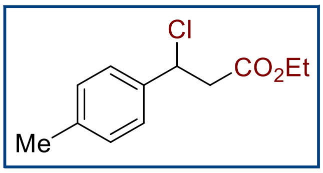

Purification by flash chromatography (PE/EA = 20/1) afforded **3c**. Colorless oil; 28.7 mg, 63% yield; **^{1}H NMR (400 MHz, $CDCl_3$)** δ (ppm) = 1.24 (t, *J* = 7.1 Hz, 3H), 2.34 (s, 3H), 3.01 (dd, *J* = 15.8, 5.8 Hz, 1H), 3.17 (dd, *J* = 15.8, 9.0 Hz, 1H), 4.11−4.19 (m, 2H), 5.33 (dd, *J* = 9.0, 5.8 Hz, 1H), 7.16 (d, *J* = 7.9 Hz, 2H), 7.30 (d, *J* = 7.9 Hz, 2H); **^{13}C NMR (100 MHz, $CDCl_3$)** δ (ppm) = 14.1, 21.1, 44.8, 58.1, 61.0, 126.8, 129.4,

137.4, 138.6, 169.6; HRMS (ESI) for $C_{12}H_{15}ClO_2Na$ $[M+Na]^+$ calcd. 249.0653, found 245.1153 (-Cl, +OMe). All analytical data are consistent with those reported in the literature.[5]

ethyl 3-(4-(tert-butyl)phenyl)-3-chloropropanoate (3d)

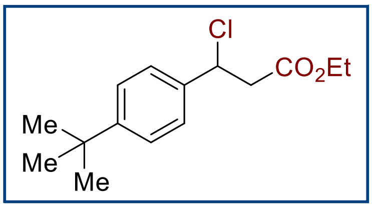

Purification by flash chromatography (PE/EA = 20/1) afforded **3d**. Colorless oil; 36.5 mg, 68% yield; **^{1}H NMR (400 MHz, CDCl$_3$)** δ (ppm) = 1.24 (t, *J* = 7.1 Hz, 3H), 1.31 (s, 9H), 3.01 (dd, *J* = 15.8, 5.7 Hz, 1H), 3.17 (dd, *J* = 15.8, 9.3 Hz, 1H), 4.13−4.20 (m, 2H), 5.34 (dd, *J* = 9.3, 5.7 Hz, 1H), 7.32−7.39 (m, 4H); **^{13}C NMR (100 MHz, CDCl$_3$)** δ (ppm) = 14.1, 31.2, 34.6, 44.8, 58.1, 61.0, 125.7, 126.6, 137.3, 151.8, 169.7; HRMS (ESI) for $C_{15}H_{21}ClO_2Na$ $[M+Na]^+$ calcd. 291.1122, found 255.1367 (-HCl), 287.1622 (-Cl, +OMe).

ethyl 3-chloro-3-(4-fluorophenyl)propanoate (3e)

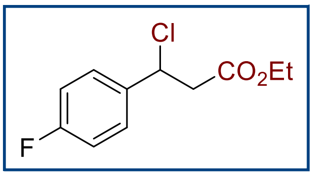

Purification by flash chromatography (PE/EA = 20/1) afforded **3e**. Colorless oil; 30.5 mg, 66% yield; **^{1}H NMR (400 MHz, CDCl$_3$)** δ (ppm) = 1.23 (t, *J* = 7.1 Hz, 3H), 3.00 (dd, *J* = 15.9, 6.3 Hz, 1H), 3.16 (dd, *J* = 15.9, 8.7 Hz, 1H), 4.09−4.21 (m, 2H), 5.33 (dd, *J* = 8.7, 6.3 Hz, 1H), 7.02−7.07 (m, 2H), 7.37−7.42 (m, 2H); **^{13}C NMR (100 MHz, CDCl$_3$)** δ (ppm) = 14.1, 45.0, 57.2, 61.1, 115.7 (d, *J* = 21.6 Hz), 128.8 (d, *J* = 8.4 Hz), 136.2 (d, *J* = 3.1 Hz), 162.6 (d, *J* = 246.6 Hz), 169.3; HRMS (ESI) for $C_{11}H_{12}FClO_2Na$ $[M+Na]^+$ calcd. 253.0402, found 249.0908 (-Cl, +OMe).

ethyl 3-chloro-3-(4-chlorophenyl)propanoate (3f)

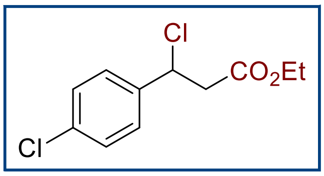

Purification by flash chromatography (PE/EA = 20/1) afforded **3f**. Colorless oil; 33.7 mg, 68% yield; **^{1}H NMR (400 MHz, CDCl$_3$)** δ (ppm) = 1.23 (t, *J* = 7.1 Hz, 3H), 3.00 (dd, *J* = 15.9, 6.3 Hz, 1H), 3.15 (dd, *J* = 15.9, 8.6 Hz, 1H), 4.09−4.21 (m, 2H), 5.31 (dd, *J* = 8.6, 6.3 Hz, 1H), 7.32−7.37 (m, 4H); **^{13}C NMR (100 MHz, CDCl$_3$)** δ (ppm) = 14.1,

44.8, 57.1, 61.1, 128.3, 129.0, 134.5, 138.8, 169.2; HRMS (ESI) for $C_{11}H_{12}Cl_2O_2Na$ $[M+Na]^+$ calcd. 269.0107, found 265.0612 (-Cl, +OMe).

ethyl 3-(4-bromophenyl)-3-chloropropanoate (3g)

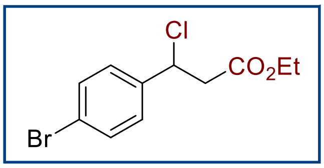

Purification by flash chromatography (PE/EA = 20/1) afforded **3g**. Colorless oil; 43.7 mg, 75% yield; **^{1}H NMR (400 MHz, $CDCl_3$)** δ (ppm) = 1.23 (t, *J* = 7.1 Hz, 3H), 2.99 (dd, *J* = 16.0, 6.3 Hz, 1H), 3.14 (dd, *J* = 16.0, 8.6 Hz, 1H), 4.09−4.21 (m, 2H), 5.29 (dd, *J* = 8.6, 6.3 Hz, 1H), 7.28−7.31 (m, 2H), 7.47−7.51 (m, 2H); **^{13}C NMR (100 MHz, $CDCl_3$)** δ (ppm) = 14.1, 44.7, 57.1, 61.1, 122.6, 128.6, 131.9, 139.3, 169.2; HRMS (ESI) for $C_{11}H_{12}ClBrO_2Na$ $[M+Na]^+$ calcd. 312.9601, found 309.0108 (-Cl, +OMe).

methyl 4-(1-chloro-3-ethoxy-3-oxopropyl)benzoate (3h)

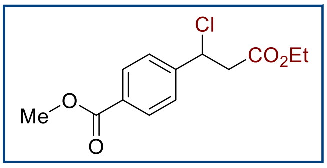

Purification by flash chromatography (PE/EA = 8/1) afforded **3h**. Colorless oil; 22.2 mg, 41% yield; **^{1}H NMR (400 MHz, $CDCl_3$)** δ (ppm) = 1.23 (t, *J* = 7.1 Hz, 3H), 3.03 (dd, *J* = 16.0, 6.2 Hz, 1H), 3.17 (dd, *J* = 16.0, 8.7 Hz, 1H), 3.92 (s, 3H), 4.10−4.21 (m, 2H), 5.37 (dd, *J* = 8.7, 6.2 Hz, 1H), 7.50 (d, *J* = 8.3 Hz, 2H), 8.03 (d, *J* = 8.3 Hz, 2H); **^{13}C NMR (100 MHz, $CDCl_3$)** δ (ppm) = 14.1, 44.6, 52.2, 57.1, 61.1, 127.0, 130.1, 130.4, 145.0, 166.4, 169.2; HRMS (ESI) for $C_{13}H_{15}ClO_4Na$ $[M+Na]^+$ calcd. 293.0551, found 293.0563.

ethyl 3-chloro-3-(4-formylphenyl)propanoate (3i)

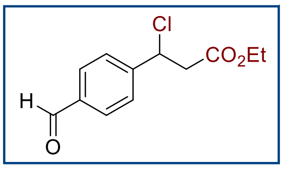

Purification by flash chromatography (PE/EA = 8/1) afforded **3i**. Colorless oil; 25.5 mg, 53% yield; **^{1}H NMR (400 MHz, $CDCl_3$)** δ (ppm) = 1.23 (t, *J* = 7.1 Hz, 3H), 3.05 (dd, *J* = 16.0, 6.3 Hz, 1H), 3.19 (dd, *J* = 16.0, 8.6 Hz, 1H), 4.10−4.22 (m, 2H), 5.38 (dd, *J* = 8.6, 6.3 Hz, 1H), 7.60 (d, *J* = 8.2 Hz, 2H), 7.89 (d, *J* = 8.3 Hz, 2H) , 10.0 (s, 1H); **^{13}C NMR (100 MHz, $CDCl_3$)** δ (ppm) = 14.1, 44.5, 57.0, 61.2, 127.7, 130.1, 136.4, 146.5, 169.1, 191.5; HRMS (ESI) for $C_{12}H_{13}ClO_3Na$ $[M+Na]^+$ calcd. 263.0445, found 259.0952 (-Cl,

+OMe).

ethyl 3-chloro-3-(4-(chloromethyl)phenyl)propanoate (3j)

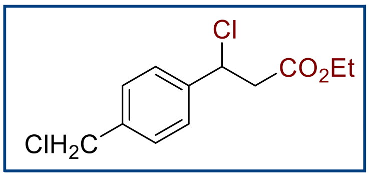

Purification by flash chromatography (PE/EA = 20/1) afforded **3j**. Colorless oil; 32.4 mg, 62% yield; **^{1}H NMR (400 MHz, CDCl$_3$)** δ (ppm) = 1.24 (t, J = 7.1 Hz, 3H), 3.01 (dd, J = 15.9, 6.0 Hz, 1H), 3.16 (dd, J = 15.9, 8.9 Hz, 1H), 4.10–4.21 (m, 2H), 4.57 (s, 2H), 5.34 (dd, J = 8.9, 6.0 Hz, 1H), 7.37–7.43 (m, 4H); **^{13}C NMR (100 MHz, CDCl$_3$)** δ (ppm) = 14.1, 44.8, 45.6, 57.5, 61.1, 127.3, 129.0, 137.9, 140.5, 169.4; HRMS (ESI) for $C_{12}H_{14}Cl_2O_2Na$ [M+Na]$^+$ calcd. 283.0263, found 283.0273.

ethyl 3-chloro-3-(naphthalen-1-yl)propanoate (3k)

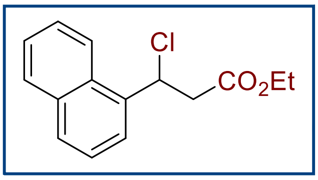

Purification by flash chromatography (PE/EA = 20/1) afforded **3k**. Colorless oil; 33.2 mg, 63% yield; **^{1}H NMR (400 MHz, CDCl$_3$)** δ (ppm) = 1.25 (t, J = 7.1 Hz, 3H), 3.25 (dd, J = 16.0, 5.0 Hz, 1H), 3.38 (dd, J = 16.0, 9.4 Hz, 1H), 4.14–4.26 (m, 2H), 6.19 (dd, J = 9.4, 5.0 Hz, 1H), 7.45–7.55 (m, 2H), 7.58–7.63 (m, 1H), 7.67 (d, J = 7.1 Hz, 1H), 7.83–7.90 (m, 2H), 8.21 (d, J = 8.5 Hz, 1H); **^{13}C NMR (100 MHz, CDCl$_3$)** δ (ppm) = 14.1, 43.8, 54.3, 61.1, 122.9, 124.3, 125.2, 126.0, 126.8, 129.0, 129.5, 130.2, 133.9, 135.4, 169.7; HRMS (ESI) for $C_{15}H_{15}ClO_2Na$ [M+Na]$^+$ calcd. 285.0653, found 281.1157 (-Cl, +OMe).

ethyl 3-chloro-3-(naphthalen-1-yl)propanoate (3l)

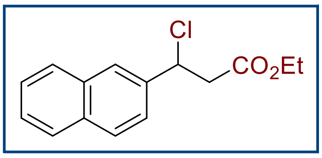

Purification by flash chromatography (PE/EA = 20/1) afforded **3l**. Colorless oil; 43.6 mg, 83% yield; **^{1}H NMR (400 MHz, CDCl$_3$)** δ (ppm) = 1.23 (t, J = 7.1 Hz, 3H), 3.12 (dd, J = 15.9, 6.0 Hz, 1H), 3.27 (dd, J = 15.9, 8.9 Hz, 1H), 4.10–4.22 (m, 2H), 5.52 (dd, J = 8.9, 6.0 Hz, 1H), 7.47–7.52 (m, 2H), 7.55 (dd, J = 8.6, 1.9 Hz, 1H), 7.80–7.87 (m, 4H); **^{13}C NMR (100 MHz, CDCl$_3$)** δ (ppm) = 14.1, 44.8, 58.4, 61.0, 124.4, 126.0, 126.5, 126.6, 127.7, 128.1, 128.9, 133.0, 133.3, 137.5, 169.5; HRMS (ESI) for $C_{15}H_{15}ClO_2Na$

[M+Na]$^+$ calcd. 285.0653, found 281.1155 (-Cl, +OMe).

ethyl 3-chloro-3-(o-tolyl)propanoate (3m)

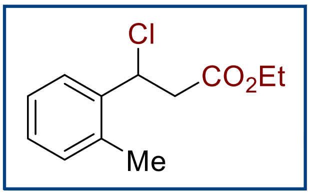

Purification by flash chromatography (PE/EA = 20/1) afforded **3m**. Colorless oil; 28.6 mg, 63% yield; **^{1}H NMR (400 MHz, CDCl$_3$)** δ (ppm) = 1.23 (t, *J* = 7.1 Hz, 3H), 2.46 (s, 3H), 3.06 (dd, *J* = 16.0, 5.7 Hz, 1H), 3.17 (dd, *J* = 16.0, 9.1 Hz, 1H), 4.11−4.22 (m, 2H), 5.61 (dd, *J* = 9.1, 5.7 Hz, 1H), 7.15−7.24 (m, 3H), 7.42−7.46 (m, 1H); **^{13}C NMR (100 MHz, CDCl$_3$)** δ (ppm) = 14.1, 19.1, 43.6, 54.3, 61.0, 126.1, 126.6, 128.6, 130.8, 135.6, 138.2, 169.7; HRMS (ESI) for $C_{12}H_{15}ClO_2Na$ [M+Na]$^+$ calcd. 249.0653, found 245.1161 (-Cl, +OMe).

ethyl 3-chloro-3-(m-tolyl)propanoate (3n)

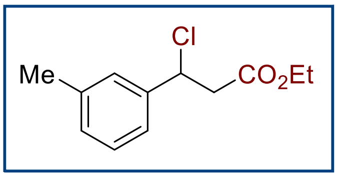

Purification by flash chromatography (PE/EA = 20/1) afforded **3n**. Colorless oil; 27.7 mg, 61% yield; **^{1}H NMR (400 MHz, CDCl$_3$)** δ (ppm) = 1.24 (t, *J* = 7.1 Hz, 3H), 2.36 (s, 3H), 3.01 (dd, *J* = 15.9, 5.7 Hz, 1H), 3.17 (dd, *J* = 15.9, 9.2 Hz, 1H), 4.11−4.22 (m, 2H), 5.31 (dd, *J* = 9.2, 5.7 Hz, 1H), 7.13 (d, *J* = 7.2 Hz, 1H), 7.19−7.27 (m, 3H); **^{13}C NMR (100 MHz, CDCl$_3$)** δ (ppm) = 14.1, 21.4, 44.9, 58.2, 61.0, 123.9, 127.6, 128.7, 129.5, 138.5, 140.2, 169.6; HRMS (ESI) for $C_{12}H_{15}ClO_2Na$ [M+Na]$^+$ calcd. 249.0653, found 245.1154 (-Cl, +OMe).

ethyl 3-chloro-3-(2-fluorophenyl)propanoate (3o)

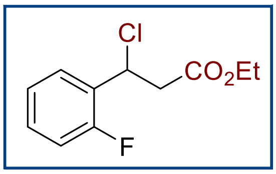

Purification by flash chromatography (PE/EA = 20/1) afforded **3o**. Colorless oil; 19.0 mg, 41% yield; **^{1}H NMR (400 MHz, CDCl$_3$)** δ (ppm) = 1.24 (t, *J* = 7.1 Hz, 3H), 3.07 (dd, *J* = 16.1, 5.8 Hz, 1H), 3.21 (dd, *J* = 16.1, 9.0 Hz, 1H), 4.11−4.23 (m, 2H), 5.64 (dd, *J* = 9.0, 5.8 Hz, 1H), 7.04−7.09 (m, 1H), 7.14−7.19 (m, 1H), 7.28−7.34 (m, 1H), 7.48 (td, *J* = 7.6, 1.6 Hz, 1H); **^{13}C NMR (100 MHz, CDCl$_3$)** δ (ppm) = 14.1, 44.6 (d, *J* = 1.1 Hz), 51.2 (d, *J* = 3.8 Hz), 61.1, 115.9 (d, *J* = 21.4 Hz), 124.5 (d, *J* = 3.6 Hz), 127.4 (d, *J* = 12.9

Hz), 128.4 (d, J = 3.2 Hz), 130.4 (d, J = 8.3 Hz), 159.6 (d, J = 247.6 Hz), 169.3; HRMS (ESI) for $C_{11}H_{12}ClFO_2Na$ $[M+Na]^+$ calcd. 253.0402, found 249.0905 (-Cl, +OMe).

ethyl 3-chloro-3-(3-fluorophenyl)propanoate (3p)

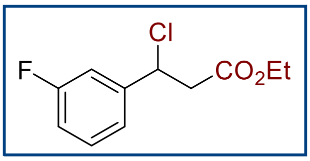

Purification by flash chromatography (PE/EA = 20/1) afforded **3p**. Colorless oil; 18.5 mg, 40% yield; **^{1}H NMR (400 MHz, $CDCl_3$)** δ (ppm) = 1.24 (t, J = 7.1 Hz, 3H), 3.01 (dd, J = 16.0, 6.1 Hz, 1H), 3.14 (dd, J = 16.0, 8.8 Hz, 1H), 4.09−4.22 (m, 2H), 5.32 (dd, J = 8.8, 6.1 Hz, 1H), 6.99−7.05 (m, 1H), 7.12−7.16 (m, 1H), 7.19 (d, J = 7.8 Hz, 1H), 7.30−7.36 (m, 1H); **^{13}C NMR (100 MHz, $CDCl_3$)** δ (ppm) = 14.1, 44.8, 57.1 (d, J = 1.7 Hz), 61.1, 114.1 (d, J = 22.3 Hz), 115.7 (d, J = 21.0 Hz), 122.6 (d, J = 2.9 Hz), 130.3 (d, J = 8.1 Hz), 142.6 (d, J = 7.2 Hz), 162.7 (d, J = 245.4 Hz), 169.2; HRMS (ESI) for $C_{11}H_{12}ClFO_2Na$ $[M+Na]^+$ calcd. 253.0402, found 249.0909 (-Cl, +OMe).

ethyl 3-chloro-3-((8R,9S,13S,14S)-13-methyl-17-oxo-7,8,9,11,12,13,14,15,16,17-decahydro-6H-cyclopenta[a]phenanthren-3-yl)propanoate (3q)

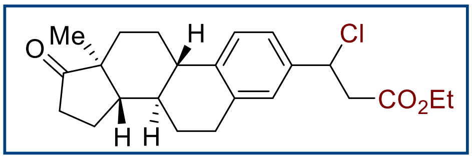

Purification by flash chromatography (PE/EA = 6/1) afforded **3q**. Colorless oil; 48.3 mg, 62% yield; **^{1}H NMR (400 MHz, $CDCl_3$)** δ (ppm) = 0.91 (s, 3H), 1.26 (t, J = 7.2 Hz, 3H), 1.45−1.55 (m, 4H), 1.58−1.67 (m, 2H), 1.93−1.98 (m, 1H), 2.01−2.20 (m, 3H), 2.26−2.32 (m, 1H), 2.40−2.45 (m, 1H), 2.51 (dd, J = 19.1, 8.9 Hz, 1H), 2.91−2.94 (m, 2H), 3.01 (dd, J = 16.0, 5.6 Hz, 1H), 3.17 (dd, J = 16.0, 9.3 Hz, 1H), 4.11−4.23 (m, 2H), 5.31 (dd, J = 9.3, 5.6 Hz, 1H), 7.15 (s, 1H), 7.19 (d, J = 8.1 Hz, 1H), 7.29 (d, J = 8.12 Hz, 1H); **^{13}C NMR (100 MHz, $CDCl_3$)** δ (ppm) = 13.8, 14.1, 21.5, 25.6, 26.3, 29.3, 31.5, 35.8, 37.9, 44.3, 44.6, 44.7, 47.9, 50.4, 58.0, 58.1, 61.0, 124.1, 124.2, 125.8, 125.8, 127.4, 127.5, 137.0, 137.7, 140.4, 169.6, 220.8; HRMS (ESI) for $C_{23}H_{29}ClO_3Na$ $[M+Na]^+$ calcd. 411.1697, found 411.1712.

methyl 3-chloro-3-phenylpropanoate (3r)

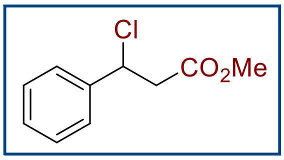

Purification by flash chromatography (PE/EA = 20/1) afforded **3r**. Colorless oil; 15.1 mg, 38% yield; **^{1}H NMR (400 MHz, $CDCl_3$)** δ (ppm) = 3.03 (dd, *J* = 16.0, 5.7 Hz, 1H), 3.19 (dd, *J* = 16.0, 9.1 Hz, 1H), 3.71 (s, 3H), 5.35 (dd, *J* = 9.1, 5.7 Hz, 1H), 7.30−7.43 (m, 5H); **^{13}C NMR (100 MHz, $CDCl_3$)** δ (ppm) = 44.6, 52.0, 58.0, 126.9, 128.7, 128.8, 140.2, 170.0; HRMS (ESI) for $C_{10}H_{11}ClO_2Na$ $[M+Na]^+$ calcd. 221.0340, found 217.0841 (-Cl, +OMe).

(R)-2-(1,3-dioxoisoindolin-2-yl)-3-phenylpropyl 3-chloro-3-phenylpropanoate (3s)

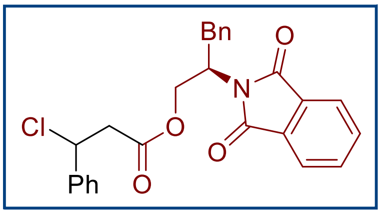

Purification by flash chromatography (PE/EA = 4/1) afforded **3s**. Colorless oil; 57.3 mg, 64% yield; **^{1}H NMR (400 MHz, $CDCl_3$)** δ (ppm) = 2.91−2.94 (m, 1H), 3.05−3.19 (m, 2H), 3.29−3.35 (m, 1H), 4.40−4.47 (m, 1H), 4.67−4.74 (m, 1H), 4.76−4.85 (m, 1H), 5.19−5.24 (m, 1H), 7.12−7.22 (m, 5H), 7.24−7.35 (m, 5H), 7.65−7.69 (m, 2H), 7.75−7.79 (m, 2H); **^{13}C NMR (100 MHz, $CDCl_3$)** δ (ppm) = 34.8, 34.8, 44.4, 44.6, 51.6, 51.6, 57.7, 57.7, 63.8, 63.9, 123.2, 126.8, 126.8, 128.6, 128.7, 128.7, 128.8, 131.5, 134.0, 136.6, 140.0, 140.0, 168.1, 168.2, 169.0, 169.1; HRMS (ESI) for $C_{26}H_{22}ClNO_4Na$ $[M+Na]^+$ calcd. 470.1130, found 470.1140.

(1R,2R,4S)-1,7,7-trimethylbicyclo[2.2.1]heptan-2-yl 3-chloro-3-phenylpropanoate (3t)

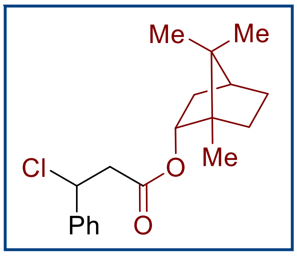

Purification by flash chromatography (PE/EA = 30/1) afforded **3t**. Colorless oil; 56.0 mg, 87% yield; **^{1}H NMR (400 MHz, $CDCl_3$)** δ (ppm) = 0.75−0.91 (m, 10H), 1.12−1.21 (m, 1H), 1.25−1.32 (m, 1H), 1.63−1.66 (m, 1H), 1.67−1.77 (m, 1H), 1.84−1.91 (m, 1H), 2.25−2.35 (m, 1H), 3.05−3.11 (m, 1H), 3.16−3.24 (m, 1H), 4.87−4.91 (m, 1H), 5.32−5.37 (m, 1H), 7.29−7.39 (m, 3H), 7.43 (d, *J* = 7.0 Hz, 2H); **^{13}C NMR (100 MHz, $CDCl_3$)** δ (ppm) = 13.3, 13.4, 18.8, 19.6, 27.1, 27.9, 36.4, 36.5, 44.7, 44.7, 45.0, 45.1, 47.8, 48.7, 58.2, 58.3, 80.7, 126.9, 126.9, 128.7, 128.8, 128.8, 140.2, 169.7, 169.8; HRMS (ESI) for $C_{19}H_{25}ClO_2Na$ $[M+Na]^+$

calcd. 343.1435, found 343.1450.

(3R,5S,8R,9S,10S,13S,14S)-10,13-dimethyl-17-oxohexadecahydro-1H-cyclopenta[a]phenanthren-3-yl 3-chloro-3-phenylpropanoate (3u)

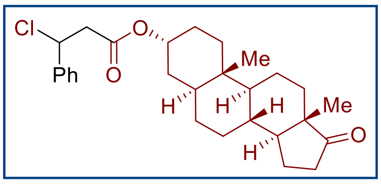

Purification by flash chromatography (PE/EA = 6/1) afforded **3u**. Colorless oil; 41.1 mg, 45% yield, dr was determined by HPLC analysis (CHIRALPAK IB-H, n-hexane/*i*PrOH, 98:2, 1.0 mL/min, 210 nm): t_r (1) = 13.3 min; t_r (2) = 16.9 min: 1:1 dr; **^{1}H NMR (400 MHz, CDCl$_3$)** δ (ppm) = 0.72 (s, 3H), 0.79 (s, 3H), 0.86−0.95 (m, 1H), 0.99−1.23 (m, 6H), 1.25−1.62 (m, 10H), 1.70−1.76 (m, 2H), 1.84−1.91 (m, 1H), 1.96−2.06 (m, 1H), 2.34−2.41 (m, 1H), 2.97−3.03 (m, 1H), 3.08−3.15 (m, 1H), 4.95−4.97 (m, 1H), 5.25−5.29 (m, 1H), 7.22−7.31 (m, 3H), 7.34−7.37 (m, 2H); **^{13}C NMR (100 MHz, CDCl$_3$)** δ (ppm) = 11.3, 13.8, 20.0, 21.7, 25.9, 25.9, 27.9, 30.7, 31.5, 32.6, 32.7, 34.9, 35.8, 40.0, 45.1, 45.1, 47.8, 51.5, 54.2, 58.3, 58.3, 70.9, 126.9, 128.7, 128.8, 140.2, 168.9, 221.3; HRMS (ESI) for $C_{28}H_{37}ClO_3Na$ $[M+Na]^+$ calcd. 479.2323, found 479.2337.

(3S,5S,8R,9S,10S,13R,14S,17R)-10,13-dimethyl-17-((R)-6-methylheptan-2-yl)hexadecahydro-1H-cyclopenta[a]phenanthren-3-yl 3-chloro-3-phenylpropanoate (3v)

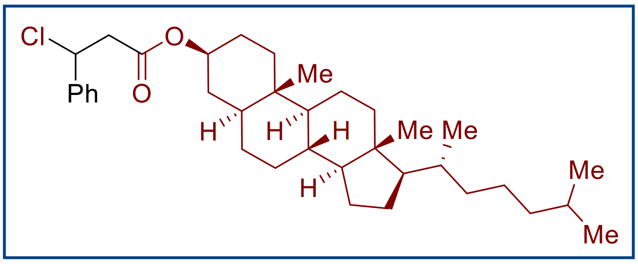

Purification by flash chromatography (PE/DCM = 4/1) afforded **3v**. White solid; 77.0 mg, 69% yield, m.p. = 125-127 °C; dr was determined by HPLC analysis (CHIRALPAK IB-H, n-hexane/*i*PrOH, 98:2, 1.0 mL/min, 210 nm): t_r (major) = 4.6 min; t_r ((minor) = 4.9 min: 2:1 dr;**^{1}H NMR (400 MHz, CDCl$_3$)** δ (ppm) = 0.59−0.64 (m, 4H), 0.81 (s, 3H), 0.85−0.90 (m, 10H), 0.94−1.03 (m, 4H), 1.06−1.16 (m, 6H), 1.21−1.30 (m, 4H), 1.29−1.36 (m, 5H), 1.44−1.56 (m, 5H), 1.63−1.84 (m, 4H), 1.94−1.97 (m, 1H), 3.00 (dd, J = 15.6, 5.9 Hz, 1H), 3.14 (dd, J = 15.6, 9.0 Hz, 1H),

4.67−4.75 (m, 1H), 5.33 (dd, J = 9.0, 5.9 Hz, 1H), 7.29−7.42 (m, 5H); **^{13}C NMR (100 MHz, CDCl$_3$)** δ (ppm) = 12.0, 12.2, 18.6, 21.2, 22.6, 22.8, 23.8, 24.2, 27.3, 27.3, 28.0, 28.2, 28.5, 31.9, 33.8, 33.8, 35.4, 35.4, 35.8, 36.1, 36.7, 39.5, 39.9, 42.5, 44.6, 44.6, 45.2, 54.1, 56.2, 56.4, 58.3, 74.6, 74.6, 126.9, 128.7, 128.7, 140.3, 169.0, 169.1; HRMS (ESI) for $C_{36}H_{55}ClO_2Na$ $[M+Na]^+$ calcd. 577.3783, found 577.3791.

(1R,2S,5R)-2-isopropyl-5-methylcyclohexyl 3-chloro-3-phenylpropanoate (3w)

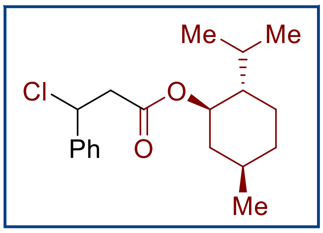

Purification by flash chromatography (PE/DCM = 4/1) afforded **3w**. Colorless oil; 43.5 mg, 68% yield; **^{1}H NMR (400 MHz, CDCl$_3$)** δ (ppm) = 0.61−0.72 (m, 3H), 0.77−0.83 (m, 2H), 0.85−0.90 (m, 5H), 0.92−0.96 (m, 1H), 0.97−1.08 (m, 1H), 1.30−1.38 (m, 1H), 1.40−1.51 (m, 1H), 1.62−1.82 (m, 3H), 1.85−1.95 (m, 1H), 3.01−3.06 (m, 1H), 3.13−3.19 (m, 1H), 4.63−4.74 (m, 1H), 5.31−5.36 (m, 1H), 7.29−7.38 (m, 3H), 7.40−7.43 (m, 2H); **^{13}C NMR (100 MHz, CDCl$_3$)** δ (ppm) = 16.1, 20.7, 20.7, 22.0, 23.2, 26.0, 26.0, 31.3, 31.3, 34.1, 40.6, 40.7, 45.1, 45.3, 46.9, 58.2, 58.2, 75.0, 126.9, 127.0, 128.7, 128.7, 128.7, 140.2, 140.2, 169.1, 169.1; HRMS (ESI) for $C_{19}H_{27}ClO_2Na$ $[M+Na]^+$ calcd. 345.1592, found 345.1598.

ethyl 3-chloro-5-phenylpentanoate (5a)

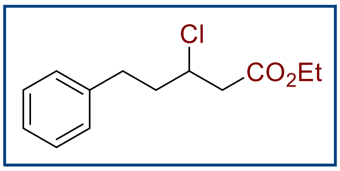

Purification by flash chromatography (PE/EA = 20/1) afforded **5a**. Colorless oil; 33.7 mg, 70% yield; **^{1}H NMR (400 MHz, CDCl$_3$)** δ (ppm) = 1.26 (t, J = 7.1 Hz, 3H), 1.98−2.14 (m, 2H), 2.70−2.82 (m, 3H), 2.87−2.94 (m, 1H), 4.11−4.22 (m, 2H), 4.24−4.31 (m, 1H), 7.19−7.22 (m, 3H), 7.28−7.32 (m, 2H); **^{13}C NMR (100 MHz, CDCl$_3$)** δ (ppm) = 14.1, 32.5, 39.6, 43.7, 57.2, 60.9, 126.2, 128.5, 128.5, 140.6, 170.0; HRMS (ESI) for $C_{13}H_{17}ClO_2Na$ $[M+Na]^+$ calcd. 263.0809, found 263.0823.

ethyl 3-chlorooctanoate (5b)

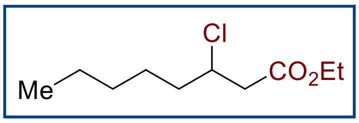

Purification by flash chromatography (PE/EA = 30/1) afforded **5b**. Colorless oil; 26.8 mg, 65% yield; **^{1}H NMR (400 MHz, $CDCl_3$)** δ (ppm) = 0.88 (t, *J* = 6.8 Hz, 3H), 1.26−1.33 (m, 7H), 1.39−1.48 (m, 1H), 1.49−1.56 (m, 1H), 1.68−1.80 (m, 2H), 2.69−2.79 (m, 2H), 4.19 (q, *J* = 7.2 Hz, 2H), 4.27−4.34 (m, 1H); **^{13}C NMR (100 MHz, $CDCl_3$)** δ (ppm) = 14.0, 14.2, 22.5, 25.9, 31.1, 38.0, 43.7, 58.0, 60.8, 170.3; HRMS (ESI) for $C_{10}H_{19}ClO_2Na$ $[M+Na]^+$ calcd. 229.0966, found 229.0970.

ethyl 3-chlorononanoate (5c)

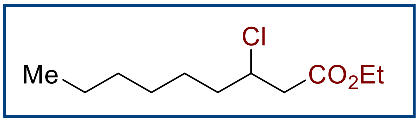

Purification by flash chromatography (PE/EA = 30/1) afforded **5c**. Colorless oil; 30.4 mg, 69% yield; **^{1}H NMR (400 MHz, $CDCl_3$)** δ (ppm) = 0.89 (t, *J* = 6.7 Hz, 3H), 1.26−1.35 (m, 9H), 1.40−1.48 (m, 1H), 1.49−1.57 (m, 1H), 1.69−1.82 (m, 2H), 2.70−2.77 (m, 2H), 4.18 (q, *J* = 7.2 Hz, 2H), 4.26−4.33 (m, 1H); **^{13}C NMR (100 MHz, $CDCl_3$)** δ (ppm) = 14.0, 14.1, 22.5, 26.2, 28.6, 31.6, 38.0, 43.7, 58.0, 60.8, 170.3; HRMS (ESI) for $C_{11}H_{21}ClO_2Na$ $[M+Na]^+$ calcd. 243.1122, found 243.1129.

ethyl 3-chlorohexadecanoate (5d)

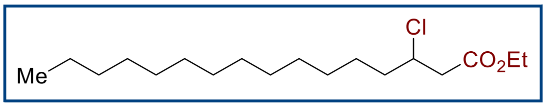

Purification by flash chromatography (PE/EA = 40/1) afforded **5d**. Colorless oil; 36.4 mg, 57% yield; **^{1}H NMR (400 MHz, $CDCl_3$)** δ (ppm) = 0.88 (t, *J* = 6.7 Hz, 3H), 1.25−1.30 (m, 23H), 1.38−1.45 (m, 1H), 1.49−1.58 (m, 1H), 1.67−1.82 (m, 2H), 2.69−2.78 (m, 2H), 4.18 (q, *J* = 7.2 Hz, 2H), 4.26−4.33 (m, 1H); **^{13}C NMR (100 MHz, $CDCl_3$)** δ (ppm) = 14.1, 14.2, 22.7, 26.3, 29.0, 29.3, 29.4, 29.5, 29.6, 29.6, 29.7, 31.9, 38.0, 43.7, 58.1, 60.8, 170.3; HRMS (ESI) for $C_{18}H_{35}ClO_2Na$ $[M+Na]^+$ calcd. 341.2218, found 341.2227.

ethyl 3-chlorooctadecanoate (5e)

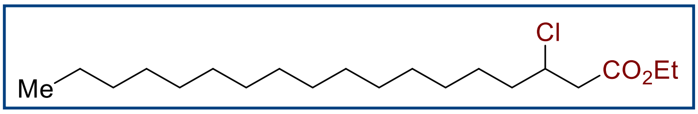

Purification by flash chromatography (PE/EA = 40/1) afforded **5e**. Colorless oil; 37.5 mg, 53% yield; **^{1}H NMR (400 MHz, $CDCl_3$)** δ (ppm) = 0.88 (t, J = 6.7 Hz, 3H), 1.25−1.30 (m, 27H), 1.38−1.45 (m, 1H), 1.49−1.55 (m, 1H), 1.67−1.82 (m, 2H), 2.68−2.78 (m, 2H), 4.18 (q, J = 7.2 Hz, 2H), 4.26−4.33 (m, 1H); **^{13}C NMR (100 MHz, $CDCl_3$)** δ (ppm) = 14.1, 14.2, 22.7, 26.3, 29.0, 29.4, 29.4, 29.5, 29.6, 29.6, 29.7, 31.9, 38.0, 43.7, 58.1, 60.8, 170.3; HRMS (ESI) for $C_{20}H_{39}ClO_2Na$ $[M+Na]^+$ calcd. 369.2531, found 369.2541.

ethyl 2-(1-chlorocyclohexyl)acetate (5f)

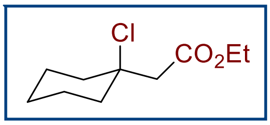

Purification by flash chromatography (PE/EA = 20/1) afforded **5f**. Colorless oil; 32.0 mg, 78% yield; **^{1}H NMR (400 MHz, $CDCl_3$)** δ (ppm) = 1.28 (t, J = 7.2 Hz, 3H), 1.54−1.81 (m, 8H), 2.05−2.09 (m, 2H), 2.80 (s, 2H), 4.17 (q, J = 7.2 Hz, 2H); **^{13}C NMR (100 MHz, $CDCl_3$)** δ (ppm) = 14.2, 22.1, 25.0, 39.5, 49.7, 60.6, 71.3, 169.5; HRMS (ESI) for $C_{10}H_{17}ClO_2Na$ $[M+Na]^+$ calcd. 227.0809, found 227.0809.

ethyl 3-chloro-4-phenylbutanoate (5g)

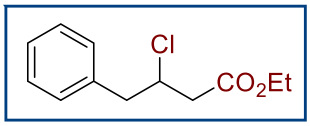

Purification by flash chromatography (PE/EA = 20/1) afforded **5g**. Colorless oil; 19.0 mg, 42% yield; **^{1}H NMR (400 MHz, $CDCl_3$)** δ (ppm) = 1.26 (t, J = 7.2 Hz, 3H), 2.67−2.79 (m, 2H), 3.05−3.15 (m, 2H), 4.16 (q, J = 7.2 Hz, 2H), 4.48−4.55 (m, 1H); 7.22−7.29 (m, 3H), 7.31−7.35 (m, 2H); **^{13}C NMR (100 MHz, $CDCl_3$)** δ (ppm) = 14.1, 42.6, 44.3, 57.7, 60.9, 127.1, 128.5, 129.4, 136.9, 170.1; HRMS (ESI) for $C_{12}H_{15}ClO_2Na$ $[M+Na]^+$ calcd. 249.0653, found 249.0659.

ethyl 3-chloro-3-cyclohexylpropanoate (5h)

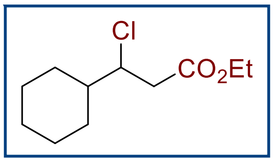

Purification by flash chromatography (PE/EA = 30/1) afforded **5h**. Colorless oil; 19.6 mg, 45% yield; **^{1}H NMR (400 MHz, $CDCl_3$)** δ (ppm) = 1.13−1.30 (m, 8H), 1.62−1.83 (m, 6H), 2.67−2.78 (m, 2H), 4.19 (q, *J* = 7.1 Hz, 2H), 4.22−4.27 (m, 1H); **^{13}C NMR (100 MHz, $CDCl_3$)** δ (ppm) = 14.2, 25.9, 26.0, 26.1, 28.1, 29.8, 41.0, 43.8, 60.9, 63.4, 170.7; HRMS (ESI) for $C_{11}H_{19}ClO_2Na$ $[M+Na]^+$ calcd. 241.0966, found 241.0963.

ethyl 3-chloro-4,4-dimethylpentanoate (5i)

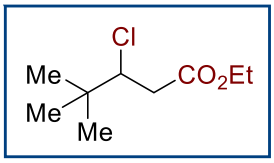

Purification by flash chromatography (PE/EA = 20/1) afforded **5i**. Colorless oil; 16.6 mg, 43% yield; **^{1}H NMR (400 MHz, $CDCl_3$)** δ (ppm) = 1.04 (s, 9H), 1.29 (t, *J* = 7.2 Hz, 3H), 2.62 (dd, *J* = 15.7, 11.1 Hz, 1H), 2.81 (dd, *J* = 15.7, 2.6 Hz, 1H), 4.17−4.23 (m, 3H); **^{13}C NMR (100 MHz, $CDCl_3$)** δ (ppm) = 14.2, 26.5, 35.6, 39.4, 60.9, 68.4, 171.1; HRMS (ESI) for $C_9H_{17}ClO_2Na$ $[M+Na]^+$ calcd. 215.0809, found 215.0814.

1-ethyl 12-methyl 3-chlorododecanedioate (5j)

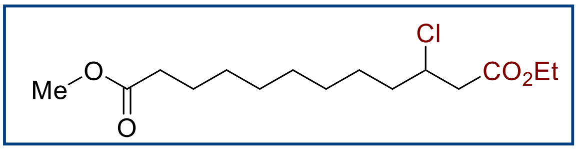

Purification by flash chromatography (PE/EA = 10/1) afforded **5j**. Colorless oil; 27.6 mg, 45% yield; **^{1}H NMR (400 MHz, $CDCl_3$)** δ (ppm) = 1.26−1.33 (m, 11H), 1.39−1.47 (m, 1H), 1.49−1.56 (m, 1H), 1.58−1.64 (m, 2H), 1.70−1.81 (m, 2H), 2.31 (t, *J* = 7.5 Hz, 2H), 2.68−2.74 (m, 2H), 3.67 (s, 3H), 4.18 (q, *J* = 7.1 Hz, 2H), 4.26−4.33 (m, 1H); **^{13}C NMR (100 MHz, $CDCl_3$)** δ (ppm) = 14.1, 24.9, 26.2, 28.8, 29.0, 29.1, 29.2, 34.0, 38.0, 43.7, 51.4, 58.0, 60.8, 170.2, 174.3; HRMS (ESI) for $C_{15}H_{27}ClO_4Na$ $[M+Na]^+$ calcd. 329.1490, found 329.1498.

3-chloro-5-ethoxy-5-oxopentyl benzoate (5k)

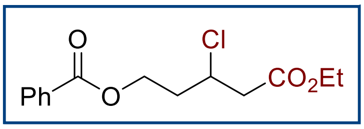

Purification by flash chromatography (PE/EA = 10/1) afforded **5k**. Colorless oil; 22.8 mg, 40% yield; **^{1}H NMR (400 MHz, $CDCl_3$)** δ (ppm) = 1.27 (t, *J* = 7.1 Hz,

3H), 2.11−2.20 (m, 1H), 2.29−2.38 (m, 1H), 2.79−2.90 (m, 2H), 4.13−4.24 (m, 2H), 4.23−4.59 (m, 3H), 7.45 (t, *J* = 7.6 Hz, 2H), 7.58 (d, *J* = 7.2 Hz, 1H), 8.05 (d, *J* = 7.6 Hz, 2H); **^{13}C NMR (100 MHz, CDCl$_3$)** δ (ppm) = 14.1, 36.8, 43.5, 54.2, 61.0, 61.5, 128.4, 129.6, 129.9, 133.1, 166.4, 169.7; HRMS (ESI) for $C_{14}H_{17}ClO_4Na$ [M+Na]$^+$ calcd. 307.0708, found 307.0717.

ethyl 3-chloro-6-oxo-6-(4-(trifluoromethoxy)phenyl)hexanoate (5l)

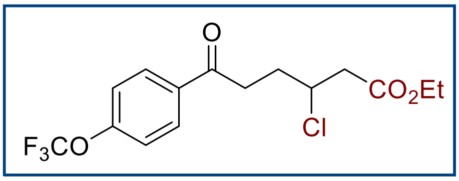

Purification by flash chromatography (PE/EA = 6/1) afforded **5l**. Colorless oil; 30.3 mg, 43% yield; **^{1}H NMR (400 MHz, CDCl$_3$)** δ (ppm) = 1.29 (t, *J* = 7.2 Hz, 3H), 2.03−2.13 (m, 1H), 2.32−2.40 (m, 1H), 2.78−2.88 (m, 2H), 3.16−3.31 (m, 2H), 4.20 (q, *J* = 7.2 Hz, 2H), 4.39−4.67 (m, 1H), 7.31 (d, *J* = 8.2 Hz, 2H), 8.05 (d, *J* = 8.2 Hz, 2H); **^{13}C NMR (100 MHz, CDCl$_3$)** δ (ppm) = 14.1, 31.9, 35.3, 43.8, 57.4, 61.0, 120.2 (q, *J* = 257.3 Hz), 120.4, 130.0, 134.8, 152.7, 169.8, 197.1; HRMS (ESI) for $C_{15}H_{16}F_3ClO_4Na$ [M+Na]$^+$ calcd. 375.0581, found 375.0594.

ethyl 3-chloro-6-cyclohexyl-6-oxohexanoate (5m)

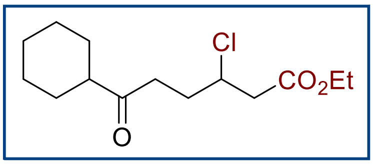

Purification by flash chromatography (PE/EA = 8/1) afforded **5m**. Colorless oil; 30.3 mg, 43% yield; **^{1}H NMR (400 MHz, CDCl$_3$)** δ (ppm) = 1.21−1.37 (m, 8H), 1.65−1.69 (m, 1H), 1.77−1.92 (m, 5H), 2.10−2.19 (m, 1H), 2.32−2.38 (m, 1H), 2.63−2.80 (m, 4H), 4.18 (q, *J* = 7.1 Hz, 2H), 4.27−4.34 (m, 1H); **^{13}C NMR (100 MHz, CDCl$_3$)** δ (ppm) = 14.1, 25.6, 25.6, 25.8, 28.4, 28.5, 31.6, 37.1, 43.8, 50.9, 57.5, 60.9, 169.8, 212.7; HRMS (ESI) for $C_{14}H_{23}ClO_3Na$ [M+Na]$^+$ calcd. 297.1228, found 297.1233.

(3S,8S,9S,10R,13R,14S,17R)-5-chloro-6-(ethoxycarbonyl)-10,13-dimethyl-17-((R)-6-methylheptan-2-yl)hexadecahydro-1H-cyclopenta[a]phenanthren-3-yl ethyl oxalate (5n)

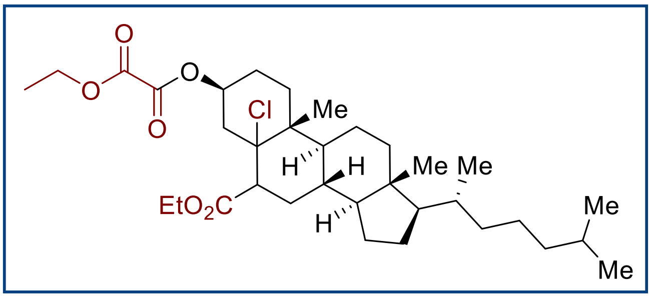

Purification by flash chromatography (PE/EA = 20/1) afforded **5n**. Colorless oil;; 25.0 mg, 21% yield; **^{1}H NMR (400 MHz, CDCl$_3$)** δ (ppm) = 0.68 (s, 3H), 0.85−0.91 (m, 9H), 0.98−1.04 (m, 4H), 1.09−1.15 (m, 6H), 1.23−1.32 (m, 7H), 1.34−1.43 (m, 6H), 1.47−1.61 (m, 5H), 1.67−1.78 (m, 2H), 183−1.92 (m, 3H), 195−2.05 (m, 3H), 2.47 (dd, J = 13.9, 3.8 Hz, 1H), 2.72 (dd, J = 13.9, 10.8 Hz, 1H), 2.92 (d, J = 3.1 Hz, 1H), 4.10−4.22 (m, 2H), 4.36 (q, J = 7.1 Hz, 2H), 5.53−5.61 (m, 1H); **^{13}C NMR (100 MHz, CDCl$_3$)** δ (ppm) = 12.2, 13.9, 14.0, 16.5, 18.6, 21.3, 22.5, 22.8, 23.8, 23.9, 25.9, 28.0, 28.2, 28.6, 31.8, 33.3, 35.7, 36.1, 39.5, 39.7, 40.3, 41.5, 42.6, 46.5, 51.5, 55.8, 56.1, 60.9, 63.1, 74.6, 84.8, 157.3, 158.0, 171.7; HRMS (ESI) for $C_{34}H_{55}ClO_6Na$ [M+Na]$^+$ calcd. 617.3579, found 617.3584.

ethyl 3-chloro-4-(indolin-1-yl)-3-methyl-4-oxobutanoate (5o)

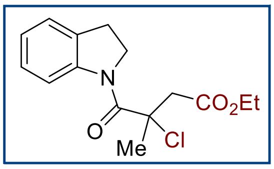

Purification by flash chromatography (PE/EA = 5/1) afforded **5o**. Colorless oil; 26.1 mg, 44% yield; **^{1}H NMR (400 MHz, CDCl$_3$)** δ (ppm) = 1.25 (t, J = 7.1 Hz, 3H), 2.06 (s, 3H), 3.14−3.27 (m, 4H), 4.17 (q, J = 7.1 Hz, 2H), 4.38−4.44 (m, 1H), 4.53−4.59 (m, 1H), 7.06 (t, J = 7.8 Hz, 1H), 7.21 (t, J = 7.8 Hz, 2H), 8.16 (d, J = 8.0 Hz, 1H); **^{13}C NMR (100 MHz, CDCl$_3$)** δ (ppm) = 14.1, 28.2, 29.3, 47.6, 50.0, 60.8, 65.8, 118.6, 124.4, 124.4, 127.4, 131.3, 143.8, 167.3, 169.2; HRMS (ESI) for $C_{15}H_{18}ClNO_3Na$ [M+Na]$^+$ calcd. 318.0867, found 318.0867.

ethyl 2-(5-methyl-4-oxo-1,2,3,4-tetrahydronaphthalen-1-yl)acetate (5a')

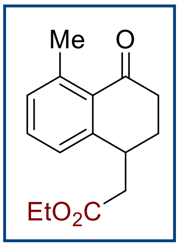

Purification by flash chromatography (PE/EA = 10/1) afforded **5a'**. Colorless oil; 21.2 mg, 43% yield; **^{1}H NMR (400 MHz, CDCl$_3$)** δ (ppm) = 1.26 (t, J = 7.1 Hz, 3H), 1.99−2.07 (m, 1H), 2.21−2.30 (m, 1H), 2.59−2.70 (m, 6H), 2.72−2.82 (m, 1H), 3.48−3.54 (m, 1H), 4.17 (q, J = 7.1 Hz, 2H), 7.12−7.17 (m, 2H), 7.34 (t, J = 7.6 Hz, 1H); **^{13}C**

NMR (100 MHz, CDCl3) δ (ppm) = 14.2, 23.2, 26.5, 35.8, 36.3, 40.0, 60.6, 125.9, 130.7, 131.1, 132.5, 141.5, 147.4, 172.0, 199.5; HRMS (ESI) for $C_{15}H_{18}O_3Na$ $[M+Na]^+$ calcd. 269.1148, found 269.1154.

ethyl 2-(4-oxo-7-phenyl-1,2,3,4-tetrahydronaphthalen-1-yl)acetate (5b')

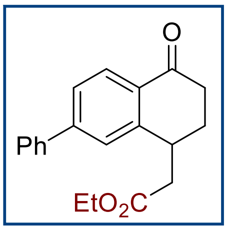

Purification by flash chromatography (PE/EA = 10/1) afforded **5b'**. Colorless oil; 24.7 mg, 40% yield; **^{1}H NMR (400 MHz, CDCl3)** δ (ppm) = 1.26 (t, *J* = 7.1 Hz, 3H), 2.08−2.15 (m, 1H), 2.31−2.39 (m, 1H), 2.64−2.85 (m, 4H), 3.58−3.64 (m, 1H), 4.19 (q, *J* = 7.1 Hz, 2H), 7.40 (t, *J* = 7.1 Hz, 1H), 7.47 (t, *J* = 7.0 Hz, 2H), 7.54−7.62 (m, 4H), 8.11 (d, *J* = 8.1 Hz, 1H); **^{13}C NMR (100 MHz, CDCl3)** δ (ppm) = 14.2, 27.4, 34.9, 35.0, 39.7, 60.8, 126.0, 126.4, 127.2, 128.2, 128.3, 128.9, 130.7, 139.8, 146.4, 146.6, 172.0, 197.3; HRMS (ESI) for $C_{20}H_{20}O_3Na$ $[M+Na]^+$ calcd. 331.1305, found 331.1312.

ethyl 2-(1-oxo-1,2,3,4-tetrahydrophenanthren-4-yl)acetate (5c')

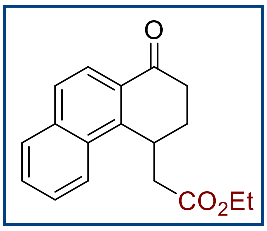

Purification by flash chromatography (PE/EA = 5/1) afforded **5c'**. Colorless oil; 28.8 mg, 51% yield; **^{1}H NMR (400 MHz, CDCl3)** δ (ppm) = 1.31 (t, *J* = 7.2 Hz, 3H), 2.27−2.44 (m, 2H), 2.71−2.94 (m, 4H), 4.21−4.28 (m, 2H), 4.30−4.35 (m, 1H), 7.60−7.66 (m, 2H), 7.79 (d, *J* = 8.7 Hz, 1H), 7.86−7.91 (m, 1H), 8.11 (d, *J* = 8.7 Hz, 1H), 8.21−8.24 (m, 1H); **^{13}C NMR (100 MHz, CDCl3)** δ (ppm) = 14.2, 25.8, 30.4, 32.9, 37.5, 60.9, 122.8, 124.6, 127.2, 127.8, 128.4, 129.1, 129.4, 130.0, 136.2, 144.7, 172.0, 197.7; HRMS (ESI) for $C_{18}H_{18}O_3Na$ $[M+Na]^+$ calcd. 305.1148, found 305.1158.

benzyl ethyl succinate (5d')

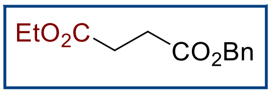

Purification by flash chromatography (DCM) afforded **5d'**. Colorless oil; 40.1 mg, 85% yield; **^{1}H NMR (400 MHz, CDCl3)** δ (ppm) = 1.24 (t, *J* = 7.1 Hz, 3H), 2.62−2.71 (m, 4H), 4.13 (q, *J* = 7.1 Hz, 2H), 5.14 (s, 2H), 7.30−7.39 (m, 5H); **^{13}C NMR (100 MHz, CDCl3)** δ (ppm) = 14.1, 29.1, 29.2, 60.7, 66.5, 128.2, 128.2, 128.5, 135.8, 172.1, 172.2; HRMS (ESI) for $C_{13}H_{16}O_4Na$

$[M+Na]^+$ calcd. 259.0941, found 259.0952.

ethyl (E)-3-([1,1'-biphenyl]-4-yl)acrylate (6a)

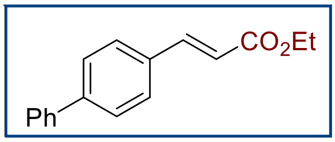

Purification by flash chromatography (PE/EA = 20/1) afforded **6a.** Colorless oil; 37.8 mg, 75% yield; **^{1}H NMR (400 MHz, $CDCl_3$)** δ (ppm) = 1.35 (t, *J* = 7.1 Hz, 3H), 4.28 (q, *J* = 7.1 Hz, 2H), 6.47 (d, *J* = 16.0 Hz, 1H), 7.34−7.39 (m, 1H), 7.43−7.47 (m, 2H), 7.58−7.63 (m, 6H), 7.73 (d, *J* = 16.0 Hz, 1H); **^{13}C NMR (100 MHz, $CDCl_3$)** δ (ppm) = 14.3, 60.5, 118.1, 127.0, 127.5, 127.8, 128.5, 128.9, 133.4, 140.1, 143.0, 144.1, 167.0; HRMS (ESI) for $C_{17}H_{16}O_2Na$ $[M+Na]^+$ calcd. 275.1043, found 275.1053.

ethyl cinnamate (6b)

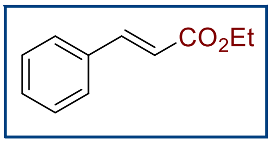

Purification by flash chromatography (PE/EA = 20/1) afforded **6b.** Colorless oil; 19.4 mg, 55% yield; **^{1}H NMR (400 MHz, $CDCl_3$)** δ (ppm) = 1.34 (t, *J* = 7.1 Hz, 3H), 4.27 (q, *J* = 7.1 Hz, 2H), 6.44 (d, *J* = 16.0 Hz, 1H), 7.37−7.39 (m, 3H), 7.51−7.54 (m, 2H), 7.69 (d, *J* = 16.0 Hz, 1H); **^{13}C NMR (100 MHz, $CDCl_3$)** δ (ppm) = 14.3, 60.5, 118.3, 128.0, 128.9, 130.2, 134.4, 144.6, 167.0; HRMS (ESI) for $C_{11}H_{13}O_2$ $[M+H]^+$ calcd. 177.0910, found 177.0921.

ethyl (*E*)-3-(p-tolyl)acrylate (6c)

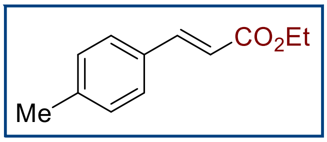

Purification by flash chromatography (PE/EA = 20/1) afforded **6c**. Colorless oil; 19.3 mg, 51% yield; **^{1}H NMR (400 MHz, $CDCl_3$)** δ (ppm) = 1.33 (t, *J* = 7.1 Hz, 3H), 2.37 (s, 3H), 4.26 (q, *J* = 7.1 Hz, 2H), 6.39 (d, *J* = 16.0 Hz, 1H), 7.19 (d, *J* = 8.0 Hz, 2H), 7.42 (d, *J* = 8.0 Hz, 2H), 7.66 (d, *J* = 16.0 Hz, 1H); **^{13}C NMR (100 MHz, $CDCl_3$)** δ (ppm) = 14.3, 21.4, 60.4, 117.1, 128.0, 129.6, 131.7, 140.6, 144.6, 167.2; HRMS (ESI) for $C_{12}H_{15}O_2$ $[M+H]^+$ calcd. 191.1067, found 191.1080.

ethyl (*E*)-3-(4-(tert-butyl)phenyl)acrylate (6d)

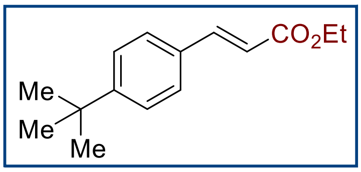

Purification by flash chromatography (PE/EA = 20/1) afforded **6d**. Colorless oil; 27.4 mg, 59% yield; **^{1}H NMR (400 MHz, CDCl$_3$)** δ (ppm) = 1.32−1.36 (m, 12H), 4.26 (q, *J* = 7.1 Hz, 2H), 6.41 (d, *J* = 16.0 Hz, 1H), 7.40 (d, *J* = 8.4 Hz, 2H), 7.47 (d, *J* = 8.4 Hz, 2H), 7.67 (d, *J* = 16.0 Hz, 1H); **^{13}C NMR (100 MHz, CDCl$_3$)** δ (ppm) = 14.3, 31.1, 34.8, 60.4, 117.3, 125.8, 127.9, 131.7, 144.5, 153.7, 167.2; HRMS (ESI) for $C_{15}H_{20}O_2Na$ [M+Na]$^+$ calcd. 255.1356, found 255.1367.

ethyl (*E*)-3-(4-methoxyphenyl)acrylate (6e)

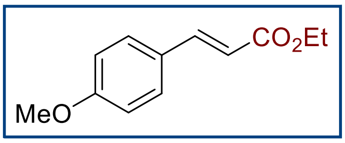

Purification by flash chromatography (PE/EA = 15/1) afforded **6e**. Colorless oil; 20.2 mg, 49% yield; **^{1}H NMR (400 MHz, CDCl$_3$)** δ (ppm) = 1.33 (t, *J* = 7.1 Hz, 3H), 3.83 (s, 3H), 4.25 (q, *J* = 7.1 Hz, 2H), 6.31 (d, *J* = 15.9 Hz, 1H), 6.88−6.92 (m, 2H), 7.45−7.49 (m, 2H), 7.64 (d, *J* = 15.9 Hz, 1H); **^{13}C NMR (100 MHz, CDCl$_3$)** δ (ppm) = 14.3, 55.3, 60.3, 114.3, 115.7, 127.2, 129.7, 144.2, 161.3, 167.3; HRMS (ESI) for $C_{12}H_{15}O_3$ [M+H]$^+$ calcd. 207.1016, found 207.1031.

ethyl (*E*)-3-(4-fluorophenyl)acrylate (6f)

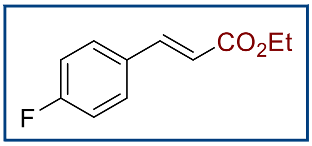

Purification by flash chromatography (PE/EA = 20/1) afforded **6f**. Colorless oil; 18.3 mg, 47% yield; **^{1}H NMR (400 MHz, CDCl$_3$)** δ (ppm) = 1.34 (t, *J* = 7.1 Hz, 3H), 4.27 (q, *J* = 7.1 Hz, 2H), 6.36 (d, *J* = 16.0 Hz, 1H), 7.05−7.10 (m, 2H), 7.49−7.53 (m, 2H), 7.65 (d, *J* = 16.0 Hz, 1H); **^{13}C NMR (100 MHz, CDCl$_3$)** δ (ppm) = 14.3, 60.5, 116.0 (d, *J* = 21.7 Hz), 118.0 (d, *J* = 2.3 Hz), 129.9 (d, *J* = 8.6 Hz), 130.7 (d, *J* = 3.2 Hz), 143.2, 163.9 (d, *J* = 249.7 Hz), 168.9; HRMS (ESI) for $C_{11}H_{12}FO_2$ [M+H]$^+$ calcd. 195.0816, found 195.0830.

ethyl (*E*)-3-(4-chlorophenyl)acrylate (6g)

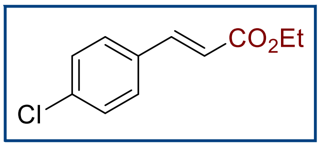

Purification by flash chromatography (PE/EA = 20/1) afforded **6g**. Colorless oil; 21.1 mg, 50% yield; **^{1}H NMR (400 MHz, CDCl$_3$)** δ (ppm) = 1.34 (t, *J* = 7.1 Hz, 3H), 4.27 (q, *J* = 7.1 Hz, 2H), 6.41 (d, *J* = 16.0 Hz, 1H), 7.36 (d, *J* = 8.5 Hz, 2H), 7.45 (d, *J* = 8.5 Hz, 2H), 7.63 (d, *J* = 16.0 Hz, 1H); **^{13}C NMR (100 MHz, CDCl$_3$)** δ (ppm) = 14.3, 60.6, 118.8, 129.1, 129.2, 132.9, 136.1, 143.1, 166.7; HRMS (ESI) for $C_{11}H_{12}ClO_2$ $[M+H]^+$ calcd. 211.0520, found 211.0530.

ethyl (*E*)-3-(4-bromophenyl)acrylate (6h)

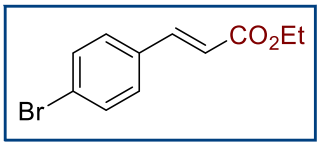

Purification by flash chromatography (PE/EA = 20/1) afforded **6h**. Colorless oil; 26.5 mg, 52% yield; **^{1}H NMR (400 MHz, CDCl$_3$)** δ (ppm) = 1.34 (t, *J* = 7.1 Hz, 3H), 4.27 (q, *J* = 7.1 Hz, 2H), 6.42 (d, *J* = 16.0 Hz, 1H), 7.37−7.40 (m, 2H), 7.50−7.53 (m, 2H), 7.61 (d, *J* = 16.0 Hz, 1H); **^{13}C NMR (100 MHz, CDCl$_3$)** δ (ppm) = 14.3, 60.6, 118.9, 124.4, 129.4, 132.1, 133.3, 143.2, 166.7; HRMS (ESI) for $C_{11}H_{12}BrO_2$ $[M+H]^+$ calcd. 255.0015, found 255.0030.

ethyl (*E*)-3-(4-(chloromethyl)phenyl)acrylate (6i)

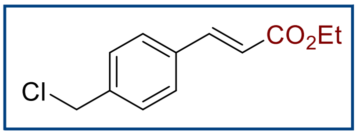

Purification by flash chromatography (PE/EA = 20/1) afforded **6i**. Colorless oil; 23.0 mg, 51% yield; **^{1}H NMR (400 MHz, CDCl$_3$)** δ (ppm) = 1.34 (t, *J* = 7.1 Hz, 3H), 4.27 (q, *J* = 7.1 Hz, 2H), 4.59 (s, 2H), 6.44 (d, *J* = 16.0 Hz, 1H), 7.40 (d, *J* = 8.2 Hz, 2H), 7.52 (d, *J* = 8.2 Hz, 2H), 7.67 (d, *J* = 16.0 Hz, 1H); **^{13}C NMR (100 MHz, CDCl$_3$)** δ (ppm) = 14.2, 45.6, 60.5, 118.8, 128.3, 129.0, 134.5, 139.4, 143.6, 166.8; HRMS (ESI) for $C_{12}H_{13}ClO_2Na$ $[M+Na]^+$ calcd. 247.0496, found 247.0494.

ethyl (*E*)-3-(naphthalen-2-yl)acrylate (6j)

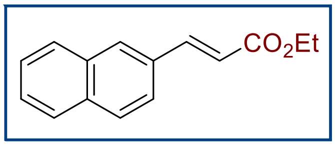

Purification by flash chromatography (PE/EA = 20/1) afforded **6j**. White solid; 25.4 mg, 56% yield, m.p. = 57-59 °C; **^{1}H NMR (400 MHz, CDCl$_3$)** δ (ppm) = 1.36 (t, *J*

= 7.1 Hz, 3H), 4.29 (q, *J* = 7.1 Hz, 2H), 6.55 (d, *J* = 16.0 Hz, 1H), 7.47−7.53 (m, 2H), 7.64−7.67 (m, 1H), 7.81−7.86 (m, 4H), 7.91 (s, 1H); **^{13}C NMR (100 MHz, CDCl$_3$)** δ (ppm) = 14.3, 60.5, 118.4, 123.4, 126.7, 127.2, 127.7, 128.5, 128.6, 129.8, 131.9, 133.2, 134.1, 144.6, 167.0; HRMS (ESI) for $C_{15}H_{14}O_2Na$ [M+Na]$^+$ calcd. 249.0886, found 249.0898.

ethyl (*E*)-3-(4-chlorophenyl)but-2-enoate (6k)

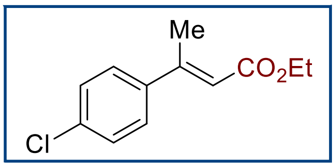

Purification by flash chromatography (PE/EA = 20/1) afforded **6k**. Colorless oil; 24.3 mg, 54% yield; **^{1}H NMR (400 MHz, CDCl$_3$)** δ (ppm) = 1.32 (t, *J* = 7.1 Hz, 3H), 2.55 (d, *J* = 1.2 Hz, 3H), 4.22 (q, *J* = 7.1 Hz, 2H), 6.11 (d, *J* = 1.2 Hz, 1H), 7.32−7.35 (m, 2H), 7.39−7.42 (m, 2H); **^{13}C NMR (100 MHz, CDCl$_3$)** δ (ppm) = 14.3, 17.8, 59.9, 117.5, 127.6, 128.7, 134.9, 140.5, 154.0, 166.6; HRMS (ESI) for $C_{12}H_{14}ClO_2$ [M+H]$^+$ calcd. 225.0677, found 225.0690.

ethyl 3,3-diphenylacrylate (6l)

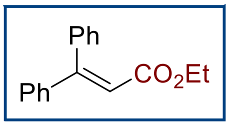

Purification by flash chromatography (PE/EA = 20/1) afforded **6l**. Colorless oil; 29.8 mg, 59% yield; **^{1}H NMR (400 MHz, CDCl$_3$)** δ (ppm) = 1.03 (t, *J* = 7.2 Hz, 3H), 3.97 (q, *J* = 7.2 Hz, 2H), 6.29 (s, 1H), 7.12−7.16 (m, 2H), 7.21−7.30 (m, 8H); **^{13}C NMR (100 MHz, CDCl$_3$)** δ (ppm) = 13.9, 60.0, 117.4, 127.8, 128.0, 128.2, 128.3, 129.1, 129.3, 138.9, 140.7, 156.4, 166.1; HRMS (ESI) for $C_{17}H_{16}O_2Na$ [M+Na]$^+$ calcd. 275.1043, found 275.1057.

ethyl (*E*)-3-(3,4,5-trimethoxyphenyl)acrylate (6m)

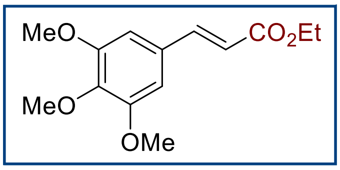

Purification by flash chromatography (PE/EA = 6/1) afforded **6m**. White solid; 26.1 mg, 49% yield, m.p. = 60-62 °C; **^{1}H NMR (400 MHz, CDCl$_3$)** δ (ppm) = 1.34 (t, *J* = 7.1 Hz, 3H), 3.88 (s, 3H), 3.891 (s, 6H), 4.27 (q, *J* = 7.1 Hz, 2H), 6.35 (d, *J* = 15.9 Hz, 1H), 6.76 (s, 2H), 7.60 (d, *J* = 15.9 Hz, 1H); **^{13}C NMR (100 MHz, CDCl$_3$)** δ (ppm) = 14.2, 56.1, 60.4, 60.9, 105.1, 117.5, 129.9, 140.0, 144.5, 153.4, 166.9; HRMS (ESI)

for $C_{14}H_{18}O_5Na$ $[M+Na]^+$ calcd. 289.1046, found 289.1059.

methyl 4-(3-ethoxy-3-oxo-1-(3,5,5,8,8-pentamethyl-5,6,7,8-tetrahydronaphthalen-2-yl)prop-1-en-1-yl)benzoate (6n)

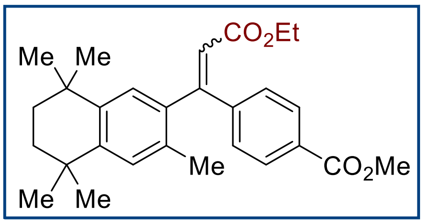

Purification by flash chromatography (PE/EA = 10/1) afforded **6n**. Colorless oil; 49.5 mg, 57% yield; **^{1}H NMR (400 MHz, CDCl$_3$)** δ (ppm) = 0.92 (t, J = 7.2 Hz, 1.8H), 1.16 (t, J = 7.1 Hz, 3H), 1.22−1.30 (m, 19.2H), 1.67 (s, 4H), 1.69 (s, 2.4H), 1.94 (s, 3H), 1.95 (s, 1.8H), 3.91−3.97 (m, 6H), 4.10 (q, J = 7.1 Hz, 2H), 6.07 (s, 1H), 6.50 (s, 0.6H), 6.95 (s, 0.6H), 7.02 (s, 1H), 7.09 (s, 0.6H), 7.11 (s, 1H), 7.30−7.32 (m, 2H), 7.36−7.40 (m, 1.2H), 7.98 (d, J = 8.2 Hz, 3.2H); **^{13}C NMR (100 MHz, CDCl$_3$)** δ (ppm) = 13.6, 13.9, 14.0, 19.3, 20.2, 31.7, 31.8, 33.8, 33.9, 34.0, 34.0, 35.0, 35.1, 35.2, 52.1, 52.1, 60.0, 60.3, 63.1, 119.9, 120.7, 126.7, 127.3, 127.6, 127.8, 128.7, 128.9, 129.2, 129.5, 129.7, 130.4, 132.1, 132.7, 135.2, 138.3, 141.8, 142.4, 144.0, 144.2, 144.4, 145.5, 156.3, 157.8, 166.1, 166.1, 166.6, 166.8; HRMS (ESI) for $C_{28}H_{34}O_4Na$ $[M+Na]^+$ calcd. 457.2349, found 457.2348.

ethyl (E)-5-phenylpent-2-enoate (6o)

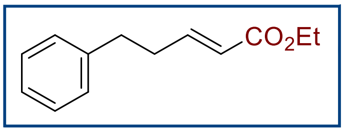

Purification by flash chromatography (PE/EA = 20/1) afforded **6o**. Colorless oil; 23.7 mg, 58% yield; **^{1}H NMR (400 MHz, CDCl$_3$)** δ (ppm) = 1.28 (t, J = 7.1 Hz, 3H), 2.48−2.55 (m, 2H), 2.77 (t, J = 7.4 Hz, 2H), 4.18 (q, J = 7.1 Hz, 2H), 5.84 (td, J = 15.6, 1.5 Hz, 1H), 7.00 (td, J = 15.6, 6.8 Hz, 1H), 7.16−7.22 (m, 3H), 7.29 (t, J = 7.3 Hz, 2H); **^{13}C NMR (100 MHz, CDCl$_3$)** δ (ppm) = 14.2, 33.8, 34.3, 60.2, 121.8, 126.1, 128.3, 128.4, 140.8, 148.0, 166.5; HRMS (ESI) for $C_{13}H_{16}O_2Na$ $[M+Na]^+$ calcd. 227.1043, found 227.1050.

1-ethyl 12-methyl (E)-dodec-2-enedioate (6p)

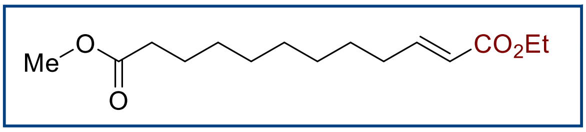

Purification by flash chromatography (PE/EA = 10/1) afforded **6p**. Colorless oil; 17.8 mg, 33% yield; **^{1}H NMR (400 MHz, CDCl$_3$)** δ (ppm) = 1.26−1.31 (m, 11H),

1.41−1.46 (m, 2H), 1.58−1.64 (m, 2H), 2.16−2.22 (m, 2H), 2.30 (t, J = 7.5 Hz, 2H), 3.67 (s, 3H), 4.18 (q, J = 7.1 Hz, 2H), 5.80 (td, J = 15.6, 1.4 Hz, 1H), 6.96 (td, J = 15.6, 7.0 Hz, 1H); **^{13}C NMR (100 MHz, CDCl$_3$)** δ (ppm) = 14.3, 24.9, 28.0, 29.0, 29.1, 29.1, 29.1, 32.1, 34.1, 51.4, 60.1, 121.2, 149.4, 166.8, 174.3; HRMS (ESI) for $C_{15}H_{26}O_4Na$ $[M+Na]^+$ calcd. 293.1723, found 293.1727.

ethyl 3,4-dihydronaphthalene-2-carboxylate (6q)

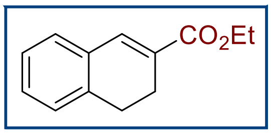

Purification by flash chromatography (PE/EA = 20/1) afforded **6q**. Colorless oil; 22.2 mg, 55% yield; **^{1}H NMR (400 MHz, CDCl$_3$)** δ (ppm) = 1.35 (t, J = 7.1 Hz, 3H), 2.61 (t, J = 8.1 Hz, 2H), 2.87 (t, J = 8.1 Hz, 2H), 4.27 (q, J = 7.1 Hz, 2H), 7.15−7.26 (m, 4H), 7.52 (s, 1H); **^{13}C NMR (100 MHz, CDCl$_3$)** δ (ppm) = 14.3, 22.2, 27.6, 60.6, 126.7, 127.6, 128.4, 129.4, 129.5, 132.6, 136.3, 136.9, 167.4; HRMS (ESI) for $C_{13}H_{15}O_2$ $[M+H]^+$ calcd. 203.1067, found 203.1080.

ethyl 2-(1,3-dimethyl-2-oxoindolin-3-yl)acetate (8a)

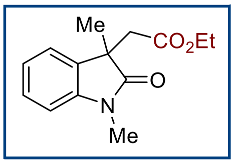

Purification by flash chromatography (*n*-hexane/ ethyl acetate = 6/1). Colorless oil; 46.2 mg, 93% yield; ^{1}H NMR (400 MHz, CDCl$_3$) δ (ppm) = 0.99 (t, J = 7.2 Hz, 3H), 1.38 (s, 3H), 2.83 (d, J = 16.1 Hz, 1H), 3.03 (d, J = 16.1 Hz, 1H), 3.25 (s, 3H), 3.80−3.95 (m, 2H), 6.85 (d, J = 7.8 Hz, 1H), 7.04 (t, J = 7.4 Hz, 1H), 7.20 (d, J = 7.0 Hz, 1H), 7.27 (td, J = 7.7, 1.0 Hz, 1H); ^{13}C NMR (100 MHz, CDCl$_3$) δ (ppm) = 13.8, 24.4, 26.3, 41.7, 45.5, 60.3, 108.0, 122.2, 122.3, 128.1, 132.9, 143.6, 169.7, 179.9; HRMS (ESI) for $C_{14}H_{17}NO_3Na$ $[M+Na]^+$ calcd. 270.1101, found 270.1108.

ethyl 2-(1,3,5-trimethyl-2-oxoindolin-3-yl)acetate (8b)

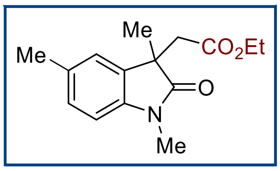

Purification by flash chromatography (*n*-hexane/ ethyl acetate = 6/1). Colorless oil; 42.0 mg, 80% yield; ^{1}H NMR (400 MHz, CDCl$_3$) δ (ppm) = 1.00 (t, J = 7.1 Hz, 3H), 1.36 (s, 3H), 2.33 (s, 3H), 2.80 (d, J = 16.1 Hz, 1H), 3.01 (d, J = 16.1 Hz, 1H), 3.23 (s, 3H), 3.81−3.96

(m, 2H), 6.74 (d, J = 7.8 Hz, 1H), 7.01 (s, 1H), 7.06 (d, J = 7.8 Hz, 1H); ^{13}C NMR (100 MHz, $CDCl_3$) δ (ppm) = 13.8, 21.1, 24.4, 26.3, 41.7, 45.6, 60.3, 107.7, 123.2, 128.3, 131.8, 132.9, 141.2, 169.8, 179.9; HRMS (ESI) for $C_{15}H_{19}NO_3Na$ $[M+Na]^+$ calcd. 284.1257, found 284.1262.

ethyl 2-(5-methoxy-1,3-dimethyl-2-oxoindolin-3-yl)acetate (8c)

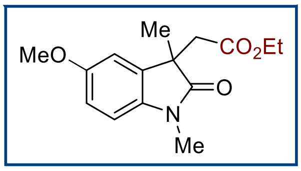

Purification by flash chromatography (*n*-hexane/ ethyl acetate = 4/1). Colorless oil; 41.0 mg, 74% yield; ^{1}H NMR (400 MHz, $CDCl_3$) δ (ppm) = 1.02 (t, J = 7.1 Hz, 3H), 1.36 (s, 3H), 2.80 (d, J = 16.3 Hz, 1H), 3.02 (d, J = 16.3 Hz, 1H), 3.23 (s, 3H), 3.79 (s, 3H), 3.83−3.97 (m, 2H), 6.75 (d, J = 8.4 Hz, 1H), 6.79 (dd, J = 8.4, 2.4Hz, 1H), 6.83 (d, J = 2.2 Hz, 1H); ^{13}C NMR (100 MHz, $CDCl_3$) δ (ppm) = 13.9, 24.4, 26.4, 41.6, 45.9, 55.8, 60.4, 108.2, 110.2, 112.0, 134.3, 137.1, 155.9, 169.7, 179.6; HRMS (ESI) for $C_{15}H_{19}NO_4Na$ $[M+Na]^+$ calcd. 300.1206, found 300.1213.

ethyl 2-(5-(tert-butyl)-1,3-dimethyl-2-oxoindolin-3-yl)acetate (8d)

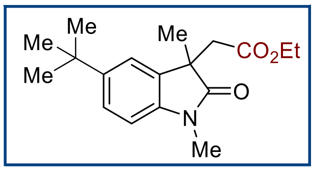

Purification by flash chromatography (*n*-hexane/ ethyl acetate = 6/1). Colorless oil; 54.2 mg, 89% yield; ^{1}H NMR (400 MHz, $CDCl_3$) δ (ppm) = 0.97 (t, J = 7.1 Hz, 3H), 1.31 (s, 9H), 1.39 (s, 3H), 2.82 (d, J = 15.8 Hz, 1H), 2.99 (d, J = 15.8 Hz, 1H), 3.23 (s, 3H), 3.81−3.94 (m, 2H), 6.77 (d, J = 8.1 Hz, 1H), 7.34 (d, J = 1.6Hz, 1H), 7.28 (dd, J = 8.2, 1.8 Hz, 1H); ^{13}C NMR (100 MHz, $CDCl_3$) δ (ppm) = 13.8, 24.3, 26.3, 31.6, 34.5, 41.8, 45.9, 60.3, 107.4, 119.6, 124.6, 132.5, 141.1, 145.6, 169.8, 180.0; HRMS (ESI) for $C_{18}H_{25}NO_3Na$ $[M+Na]^+$ calcd. 326.1727, found 326.1736.

ethyl 2-(1-methyl-2-oxo-1,2,5,6-tetrahydro-4H-pyrrolo[3,2,1-ij]quinolin-1-yl)acetate (8e)

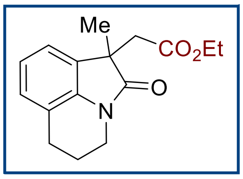

Purification by flash chromatography (*n*-hexane/ ethyl acetate = 6/1). Colorless oil; 49.6 mg, 91% yield; ^{1}H NMR (400 MHz, $CDCl_3$) δ (ppm) = 1.02 (t, *J* = 7.1 Hz, 3H), 1.39 (s, 3H), 1.98−2.08 (m, 2H), 2.77−2.84 (m, 3H), 2.98 (d, *J* = 16.1 Hz, 1H), 3.69−3.81 (m, 2H), 3.84−3.99 (m, 2H), 6.92 (t, *J* = 7.5 Hz, 1H), 7.00−7.05 (m, 2H); ^{13}C NMR (100 MHz, $CDCl_3$) δ (ppm) = 13.9, 21.1, 24.0, 24.6, 38.9, 41.5, 46.8, 60.3, 120.0, 120.2, 121.8, 126.8, 131.4, 139.3, 169.8, 178.7; HRMS (ESI) for $C_{16}H_{19}NO_3Na$ $[M+Na]^+$ calcd. 296.1257, found 296.1266.

ethyl 2-(7-methyl-6-oxo-1,2,3,4,6,7-hexahydroazepino[3,2,1-hi]indol-7-yl)acetate (8f)

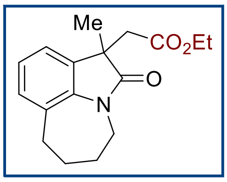

Purification by flash chromatography (*n*-hexane/ ethyl acetate = 6/1). Colorless oil; 51.8 mg, 90% yield; ^{1}H NMR (400 MHz, $CDCl_3$) δ (ppm) = 1.02 (t, *J* = 7.1 Hz, 3H), 1.35 (s, 3H), 1.96−2.08 (m, 4H), 2.80 (d, *J* = 16.2 Hz, 1H), 2.96 (t, *J* = 5.8 Hz, 2H), 3.01 (d, *J* = 16.2 Hz, 1H), 3.83−3.98 (m, 3H), 4.02−4.08 (m, 1H), 6.90 (t, *J* = 7.4 Hz, 1H), 6.97 (dd, *J* = 7.4, 0.3 Hz, 1H), 7.01 (d, *J* = 7.2 Hz, 1H); ^{13}C NMR (100 MHz, $CDCl_3$) δ (ppm) = 13.9, 24.6, 26.2, 26.5, 30.9, 41.1, 42.0, 45.5, 60.3, 120.0, 122.1, 125.2, 129.4, 133.2, 142.3, 169.8, 180.4; HRMS (ESI) for $C_{17}H_{21}NO_3Na$ $[M+Na]^+$ calcd. 310.1414, found 310.1426.

ethyl 2-(5-cyano-1,3-dimethyl-2-oxoindolin-3-yl)acetate (8g)

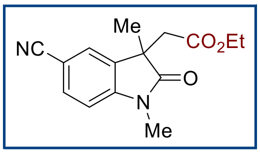

Purification by flash chromatography (*n*-hexane/ ethyl acetate = 6/1). Colorless oil; 39.7 mg, 73% yield; ^{1}H NMR (400 MHz, $CDCl_3$) δ (ppm) = 1.07 (t, *J* = 7.1 Hz, 3H), 1.38 (s, 3H), 2.87 (d, *J* = 16.8 Hz, 1H), 3.07 (d, *J* = 16.8 Hz, 1H), 3.29 (s, 3H), 3.85−3.99 (m, 2H), 6.93 (t, *J* = 8.1 Hz, 1H), 7.45 (d, *J* = 1.2 Hz, 1H), 7.62 (dd, *J* = 8.1, 1.2 Hz, 1H); ^{13}C NMR (100 MHz, $CDCl_3$) δ (ppm) = 13.9, 24.1, 26.6, 41.4, 45.2, 60.7, 105.4, 108.5, 119.2, 125.5, 133.5, 134.2, 147.7, 169.4, 179.6; HRMS (ESI) for $C_{15}H_{16}N_2O_3Na$ $[M+Na]^+$ calcd. 295.1053, found 295.1062.

ethyl 2-(1,3-dimethyl-2-oxo-5-(trifluoromethyl)indolin-3-yl)acetate (8h)

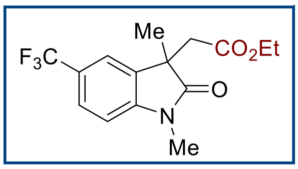

Purification by flash chromatography (*n*-hexane/ ethyl acetate = 6/1). Colorless oil; 44.8 mg, 71% yield; ^{1}H NMR (400 MHz, $CDCl_3$) δ (ppm) = 1.03 (t, *J* = 7.1 Hz, 3H), 1.40 (s, 3H), 2.88 (d, *J* = 16.5 Hz, 1H), 3.06 (d, *J* = 16.5 Hz, 1H), 3.29 (s, 3H), 3.84−3.97 (m, 2H), 6.93 (d, *J* = 8.2 Hz, 1H), 7.43 (s, 1H), 7.56 (dd, *J* = 8.2, 0.7 Hz, 1H); ^{13}C NMR (100 MHz, $CDCl_3$) δ (ppm) = 13.8, 24.2, 26.5, 41.5, 45.5, 60.6, 107.8, 119.3 (q, *J* = 3.6 Hz), 124.4 (q, *J* = 269.8 Hz), 124.5 (q, *J* = 32.4 Hz), 126.0 (q, *J* = 4.1 Hz), 133.6, 146.7, 169.5, 179.8; HRMS (ESI) for $C_{15}H_{16}F_3NO_3Na$ $[M+Na]^+$ calcd. 338.0974, found 338.0983.

ethyl 2-(5-fluoro-1,3-dimethyl-2-oxoindolin-3-yl)acetate (8i)

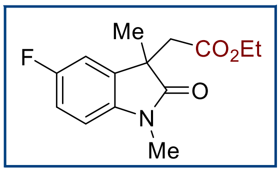

Purification by flash chromatography (*n*-hexane/ ethyl acetate = 4/1). Colorless oil; 39.2 mg, 74% yield; ^{1}H NMR (400 MHz, $CDCl_3$) δ (ppm) = 1.04 (t, *J* = 7.1 Hz, 3H), 1.37 (s, 3H), 2.81 (d, *J* = 16.4 Hz, 1H), 3.02 (d, *J* = 16.4 Hz, 1H), 3.24 (s, 3H), 3.84−3.98 (m, 2H), 6.93 (dd, *J* = 9.1, 4.1 Hz, 1H), 6.94−7.00 (m, 2H); ^{13}C NMR (100 MHz, $CDCl_3$) δ (ppm) = 13.9, 24.2, 26.5, 41.5, 45.9 (d, *J* = 1.4 Hz), 60.5, 108.4 (d, *J* = 8.1 Hz), 110.6 (d, *J* = 24.7 Hz), 114.2 (d, *J* = 23.3 Hz), 134.6 (d, *J* = 7.8 Hz), 139.5, 159.2 (d, *J* = 238.7 Hz), 169.5, 179.6; HRMS (ESI) for $C_{14}H_{16}FNO_3Na$ $[M+Na]^+$ calcd. 288.1006, found 288.1014.

ethyl 2-(5-chloro-1,3-dimethyl-2-oxoindolin-3-yl)acetate (8j)

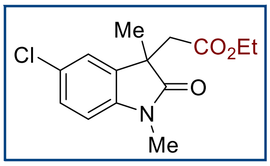

Purification by flash chromatography (*n*-hexane/ ethyl acetate = 6/1). Colorless oil; 43.4 mg, 77% yield; ^{1}H NMR (400 MHz, $CDCl_3$) δ (ppm) = 1.05 (t, *J* = 7.1 Hz, 3H), 1.36 (s, 3H), 2.82 (d, *J* = 16.5 Hz, 1H), 3.02 (d, *J* = 16.5 Hz, 1H), 3.24 (s, 3H), 3.84−3.98 (m, 2H), 6.78 (d, *J* = 8.3 Hz, 1H), 7.18 (d, *J* = 2.0 Hz, 1H), 7.25 (dd, *J* = 8.3, 2.0 Hz, 1H); ^{13}C NMR (100 MHz, $CDCl_3$) δ (ppm) = 13.9, 24.2, 26.4, 41.5, 45.7, 60.5, 108.9, 122.9, 127.6, 128.0, 134.7, 142.2, 169.5, 179.4; HRMS (ESI) for $C_{14}H_{16}ClNO_3Na$ $[M+Na]^+$ calcd. 304.0711, found 304.0720.

ethyl 2-(5-bromo-1,3-dimethyl-2-oxoindolin-3-yl)acetate (8k)

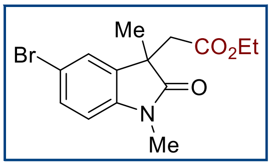

Purification by flash chromatography (*n*-hexane/ ethyl acetate = 6/1). Colorless oil; 51.9 mg, 79% yield; ^{1}H NMR (400 MHz, $CDCl_3$) δ (ppm) = 1.05 (t, *J* = 7.1 Hz, 3H), 1.36 (s, 3H), 2.81 (d, *J* = 16.5 Hz, 1H), 3.02 (d, *J* = 16.5 Hz, 1H), 3.23 (s, 3H), 3.85−3.99 (m, 2H), 6.73 (d, *J* = 8.3 Hz, 1H), 7.31 (d, *J* = 1.8 Hz, 1H), 7.40 (dd, *J* = 8.3, 1.8 Hz, 1H); ^{13}C NMR (100 MHz, $CDCl_3$) δ (ppm) = 13.9, 24.2, 26.4, 41.5, 45.6, 60.5, 109.5, 114.9, 125.6, 130.9, 135.1, 142.7, 169.5, 179.3; HRMS (ESI) for $C_{14}H_{16}BrNO_3Na$ $[M+Na]^+$ calcd. 348.0206, found 348.0216.

ethyl 2-(1,3-dimethyl-2-oxo-2,3-dihydro-1H-benzo[g]indol-3-yl)acetate (8l)

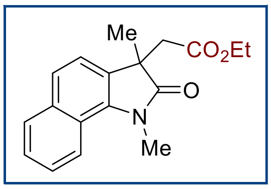

Purification by flash chromatography (*n*-hexane/ ethyl acetate = 8/1). Colorless oil; 49.5 mg, 83% yield; ^{1}H NMR (400 MHz, $CDCl_3$) δ (ppm) = 0.83 (t, *J* = 7.1 Hz, 3H), 1.58 (s, 3H), 3.07 (d, *J* = 16.7 Hz, 1H), 3.56 (s, 3H), 3.72−3.83 (m, 3H), 6.97 (d, *J* = 7.4 Hz, 1H), 7.33 (d, *J* = 7.2 Hz, 1H), 7.41−7.50 (m, 3H), 7.69 (d, *J* = 8.1 Hz, 1H); ^{13}C NMR (100 MHz, $CDCl_3$) δ (ppm) = 13.6, 29.7, 32.5, 45.0, 45.8, 60.2, 108.4, 119.4, 121.5, 122.4, 126.1, 126.4, 126.7, 133.4, 136.8, 137.7, 170.6, 173.0; HRMS (ESI) for $C_{18}H_{19}NO_3Na$ $[M+Na]^+$ calcd. 320.1257, found 320.1270.

ethyl 2-(1-ethyl-3-methyl-2-oxoindolin-3-yl)acetate (8m)

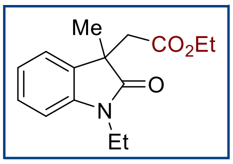

Purification by flash chromatography (*n*-hexane/ ethyl acetate = 8/1). Colorless oil; 41.2 mg, 79% yield; ^{1}H NMR (400 MHz, $CDCl_3$) δ (ppm) = 0.98 (t, *J* = 7.1 Hz, 3H), 1.29 (t, *J* = 7.2 Hz, 3H), 1.36 (s, 3H), 2.82 (d, *J* = 16.1 Hz, 1H), 3.02 (d, *J* = 16.1 Hz, 1H), 3.75−3.96 (m, 4H), 6.87 (d, *J* = 7.8 Hz, 1H), 7.02 (t, *J* = 7.5 Hz, 1H), 7.20 (d, *J* = 7.3 Hz, 1H), 7.26 (td, *J* = 7.8, 1.1 Hz, 1H); ^{13}C NMR (100 MHz, $CDCl_3$) δ (ppm) = 12.4, 13.8, 24.5, 34.7, 41.6, 45.4, 60.3, 108.1, 122.1, 122.4, 128.0, 133.1, 142.6, 169.7, 179.4; HRMS (ESI) for $C_{15}H_{19}NO_3Na$ $[M+Na]^+$ calcd. 284.1257, found 284.1268.

ethyl 2-(1-isopropyl-3-methyl-2-oxoindolin-3-yl)acetate (8n)

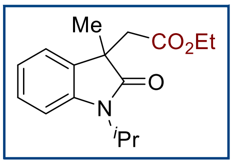

Purification by flash chromatography (*n*-hexane/ ethyl acetate = 8/1). Colorless oil; 44.0 mg, 80% yield; ^{1}H NMR (400 MHz, $CDCl_3$) δ (ppm) = 0.97 (t, *J* = 7.1 Hz, 3H), 1.35 (s, 3H), 1.51 (d, *J* = 7.1 Hz, 6H), 2.80 (d, *J* = 16.1 Hz, 1H), 3.02 (d, *J* = 16.1 Hz, 1H), 3.80−3.88 (m, 1H), 3.89−3.97 (m, 1H), 4.61−4.72 (m, 1H), 6.98−7.03 (m, 2H), 7.18−7.25 (m, 2H); ^{13}C NMR (100 MHz, $CDCl_3$) δ (ppm) = 13.8, 19.1, 19.4, 24.7, 41.7, 43.7, 45.3, 60.3, 109.8, 121.7, 122.5, 127.7, 133.5, 142.2, 169.7, 179.5; HRMS (ESI) for $C_{16}H_{21}NO_3Na$ $[M+Na]^+$ calcd. 298.1414, found 298.1421.

ethyl 2-(1-benzyl-3-methyl-2-oxoindolin-3-yl)acetate (8o)

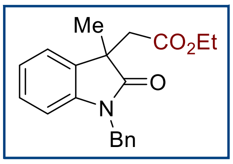

Purification by flash chromatography (*n*-hexane/ ethyl acetate = 8/1). Colorless oil; 46.5 mg, 72% yield; ^{1}H NMR (400 MHz, $CDCl_3$) δ (ppm) = 0.94 (t, *J* = 7.1 Hz, 3H), 1.43 (s, 3H), 2.88 (d, *J* = 16.2 Hz, 1H), 3.10 (d, *J* = 16.1 Hz, 1H), 3.78−3.86 (m, 1H), 3.88−3.97 (m, 1H), 4.88 (d, *J* = 15.7 Hz, 1H), 5.02 (d, *J* = 15.7 Hz, 1H), 6.72 (d, *J* = 7.8 Hz, 1H), 6.99 (td, *J* = 7.5, 0.8 Hz, 1H), 7.14 (td, *J* = 7.8, 1.2 Hz, 1H), 7.20 (dd, *J* = 7.3, 0.6 Hz, 1H), 7.23−7.27 (m, 1H), 7.29−7.37 (m, 4H); ^{13}C NMR (100 MHz, $CDCl_3$) δ (ppm) = 13.8, 24.9, 41.5, 43.9, 45.6, 60.4, 109.1, 122.3, 122.3, 127.3, 127.5, 127.9, 128.7, 132.9, 136.0, 142.6, 169.7, 179.9; HRMS (ESI) for $C_{20}H_{21}NO_3Na$ $[M+Na]^+$ calcd. 346.1414, found 346.1422.

ethyl 2-(3-benzyl-1-methyl-2-oxoindolin-3-yl)acetate (8p)

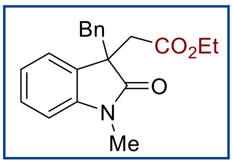

Purification by flash chromatography (*n*-hexane/ ethyl acetate = 10/1). Colorless oil; 58.8 mg, 91% yield; ^{1}H NMR (400 MHz, $CDCl_3$) δ (ppm) = 0.96 (t, *J* = 7.1 Hz, 3H), 2.93 (d, *J* = 16.2 Hz, 1H), 2.99 (s, 3H), 3.03 (s, 2H), 3.17 (d, *J* = 16.2 Hz, 1H), 3.78−3.94 (m, 2H), 6.59 (d, *J* = 7.8 Hz, 1H), 6.80 (dd, *J* = 7.8, 1.4 Hz, 2H), 6.97−7.10 (m, 5H), 7.18 (td, *J* = 7.7, 1.2 Hz, 1H); ^{13}C NMR (100 MHz, $CDCl_3$) δ (ppm) = 13.8, 25.9, 40.5, 44.0, 51.2, 60.4,

107.7, 121.8, 123.2, 126.7, 127.5, 128.2, 130.0, 130.1, 134.8, 144.1, 169.6, 178.5; HRMS (ESI) for $C_{20}H_{21}NO_3Na$ $[M+Na]^+$ calcd. 346.1414, found 346.1422.

methyl 2-(1,3-dimethyl-2-oxoindolin-3-yl)acetate (8q)

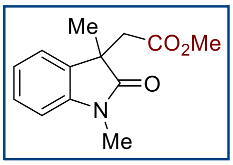

Purification by flash chromatography (*n*-hexane/ ethyl acetate = 6/1). Colorless oil; 41.2 mg, 88% yield; ^{1}H NMR (400 MHz, $CDCl_3$) δ (ppm) = 1.38 (s, 3H), 2.85 (d, J = 16.3 Hz, 1H), 3.00 (d, J = 16.3 Hz, 1H), 3.26 (s, 3H), 3.45 (s, 3H), 6.86 (d, J = 7.8 Hz, 1H), 7.04 (td, J = 7.5, 0.8 Hz, 1H), 7.19 (dd, J = 7.3, 0.6 Hz, 1H), 7.27 (td, J = 7.7, 1.2 Hz, 1H); ^{13}C NMR (100 MHz, $CDCl_3$) δ (ppm) = 24.1, 26.3, 41.3, 45.4, 51.5, 108.1, 122.2, 122.3, 128.1, 132.9, 143.5, 170.2, 179.8; HRMS (ESI) for $C_{13}H_{16}NO_3^+$ $[M+H]^+$ calcd. 234.1125, found 234.1126.

4-phenylbutyl 2-(1,3-dimethyl-2-oxoindolin-3-yl)acetate (8r)

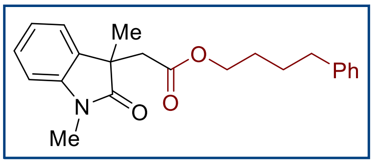

Purification by flash chromatography (*n*-hexane/ ethyl acetate = 6/1). Colorless oil; 69.5 mg, 99% yield; ^{1}H NMR (400 MHz, $CDCl_3$) δ (ppm) = 1.33−1.51 (m, 7H), 2.52 (t, J = 7.4 Hz, 2H), 2.83 (d, J = 16.2 Hz, 1H), 3.02 (d, J = 16.2 Hz, 1H), 3.20 (s, 3H), 3.78−3.88 (m, 2H), 6.80 (d, J = 7.8 Hz, 1H), 7.00 (d, J = 7.3 Hz, 1H), 7.11 (d, J = 7.1 Hz, 2H), 7.15−7.19 (m, 2H), 7.21−7.29 (m, 3H); ^{13}C NMR (100 MHz, $CDCl_3$) δ (ppm) = 24.4, 26.3, 27.3, 27.9, 35.3, 41.6, 45.5, 64.3, 108.0, 122.2, 122.3, 125.8, 128.1, 128.2, 128.3, 132.8, 141.8, 143.5, 169.8, 179.8; HRMS (ESI) for $C_{22}H_{26}NO_3^+$ $[M+H]^+$ calcd. 352.1907, found 352.1908.

hexadecyl 2-(1,3-dimethyl-2-oxoindolin-3-yl)acetate (8s)

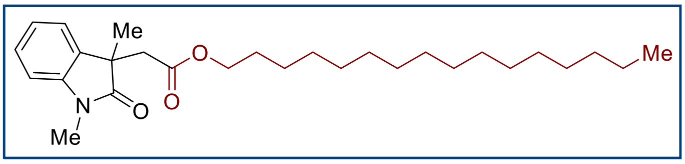

Purification by flash chromatography (*n*-hexane/ ethyl acetate = 6/1). Colorless oil; 75.4 mg, 85% yield; ^{1}H NMR (400 MHz, $CDCl_3$) δ (ppm) = 0.88 (t, J = 6.8 Hz, 3H), 1.20−1.32 (m, 28H), 1.37 (S, 3H), 2.84 (d, J = 16.2 Hz, 1H), 3.02 (d, J = 16.2 Hz,

1H), 3.25 (s, 3H), 3.75−3.86 (m, 2H), 6.84 (d, *J* = 7.8 Hz, 1H), 7.03 (td, *J* = 7.5, 0.8 Hz, 1H), 7.19 (d, *J* = 6.8 Hz, 1H), 7.26 (td, *J* = 7.7, 1.1 Hz, 1H); ^{13}C NMR (100 MHz, $CDCl_3$) δ (ppm) = 14.0, 22.6, 24.4, 25.6, 26.3, 28.3, 29.1, 29.3, 29.4, 29.5, 29.6, 29.6, 29.6, 31.9, 41.7, 45.5, 64.6, 108.0, 122.2, 122.3, 128.0, 132.9, 143.5, 169.8, 179.8; HRMS (ESI) for $C_{28}H_{46}NO_3^+$ $[M+H]^+$ calcd. 444.3472, found 444.3475.

1,7,7-trimethylbicyclo[2.2.1]heptan-2-yl 2-(1,3-dimethyl-2-oxoindolin-3-yl)acetate (8t)

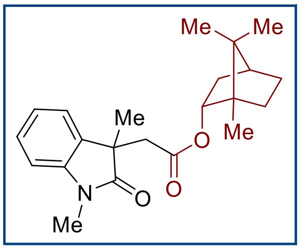

Purification by flash chromatography (*n*-hexane/ ethyl acetate = 8/1)**.** Colorless oil; 60.0 mg, 84% yield, 1:1 dr; ^{1}H NMR (400 MHz, $CDCl_3$) δ (ppm) = 0.59 (s, 3H), 0.62 (s, 3H), 0.77 (s, 6H), 0.79 (s, 6H), 0.85−1.26 (m, 6H), 1.38 (s, 3H), 1.38 (s, 3H), 1.51−1.55 (m, 2H), 1.61−1.69 (m, 4H), 2.03−2.16 (m, 2H), 2.82−2.88 (m, 2H), 3.02−3.12 (m, 2H), 3.25 (s, 3H), 3.26 (s, 3H), 4.61−4.67 (m, 2H), 6.83−6.86 (m, 2H), 7.01−7.06 (m, 2H), 7.20−7.28 (m, 4H); ^{13}C NMR (100 MHz, $CDCl_3$) δ (ppm) = 13.1, 13.1, 18.6, 19.6, 24.9, 25.2, 26.4, 26.9, 27.8, 36.0, 36.3, 41.8, 41.9, 44.6, 44.6, 45.7, 45.7, 47.6, 48.4, 48.5, 80.2, 80.3, 108.1, 108.2, 122.3, 122.4, 128.0, 132.8, 132.9, 143.4, 170.1, 179.6, 179.7; HRMS (ESI) for $C_{22}H_{29}NO_3Na$ $[M+Na]^+$ calcd. 378.2040, found 378.2053.

1,3,3-trimethylbicyclo[2.2.1]heptan-2-yl 2-(1,3-dimethyl-2-oxoindolin-3-yl)acetate (8u)

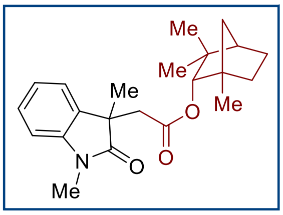

Purification by flash chromatography (*n*-hexane/ ethyl acetate = 6/1)**.** Colorless oil; 43.5 mg, 61% yield, 1.2:1 dr; ^{1}H NMR (400 MHz, $CDCl_3$) δ (ppm) = 0.45 (s, 3H), 0.51 (s, 3H), 0.72 (s, 3H), 0.82 (s, 3H), 0.91−0.99 (m, 8H), 1.06−1.09 (m, 2H), 1.30−1.38 (m, 8H), 1.41−1.52 (m, 4H), 1.58−1.61 (m, 2H), 1.72 (s, 2H), 2.85−2.93 (m, 2H), 3.05−3.13 (m, 2H), 3.25 (s, 6H), 4.13 (s, 2H), 6.83−6.85 (m, 2H), 7.00−7.04 (m, 2H), 7.21−7.28(m, 4H); ^{13}C NMR (100 MHz, $CDCl_3$) δ (ppm) = 18.9, 19.0, 19.7, 19.9, 25.1, 25.2, 25.6, 25.7, 26.4, 26.4, 26.4, 29.5, 39.1, 39.2, 41.2, 41.3, 41.5, 41.6, 45.5,

45.6, 47.9, 48.0, 48.1, 48.2, 86.8, 108.2, 122.2, 122.3, 122.3, 122.4, 132.9, 133.0, 143.5, 170.2, 170.2, 179.7; HRMS (ESI) for $C_{22}H_{29}NO_3Na$ $[M+Na]^+$ calcd. 378.2040, found 378.2040.

(1R,2S,5R)-2-isopropyl-5-methylcyclohexyl 2-(1,3-dimethyl-2-oxoindolin-3-yl)acetate (8v)

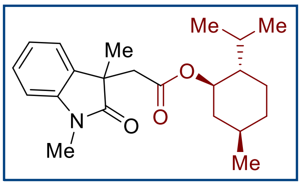

Purification by flash chromatography (*n*-hexane/ ethyl acetate = 6/1). Colorless oil; 50.0 mg, 70% yield, 1.3:1 dr; ^{1}H NMR (400 MHz, $CDCl_3$) δ (ppm) = 0.48−0.55 (m, 3H), 0.71−0.81 (m, 7H), 0.83−0.94 (m, 1H), 1.03−1.15 (m, 1H), 1.23−1.33 (m, 2H), 1.36 (s, 3H), 1.50−1.60 (m, 4H), 2.77−2.85 (m, 1H), 3.00−3.10 (m, 1H), 3.24 (s, 3H), 4.34−4.49 (m, 1H), 6.81−6.85 (m, 1H), 7.00−7.05 (m, 1H), 7.18−7.21 (m, 1H), 7.23−7.28 (m, 1H); ^{13}C NMR (100 MHz, $CDCl_3$) δ (ppm) = 15.8, 15.9, 20.7, 21.0, 21.9, 23.0, 23.2, 24.5, 24.7, 25.5, 25.9, 26.3, 31.1, 31.2, 34.1, 34.1, 40.2, 40.6, 42.0, 42.3, 45.6, 45.6, 46.8, 74.3, 74.3, 108.0, 108.0, 122.2, 122.3, 128.0, 132.9, 133.0, 143.6, 143.8, 169.3, 169.3, 179.8, 179.9; HRMS (ESI) for $C_{22}H_{31}NO_3Na$ $[M+Na]^+$ calcd. 380.2196, found 380.2196.

(3S,5S,8R,9S,10S,13R,14S,17R)-10,13-dimethyl-17-((R)-6-methylheptan-2-yl)hexadecahydro-1H-cyclopenta[a]phenanthren-3-yl 2-(1,3-dimethyl-2-oxoindolin-3-yl)acetate (8w)

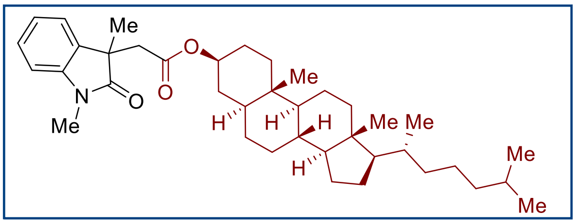

Purification by flash chromatography (*n*-hexane/ ethyl acetate = 6/1). White solid; 95.5 mg, 81% yield, m.p. = 115-117 °C; >20:1 dr; ^{1}H NMR (400 MHz, $CDCl_3$) δ (ppm) = 0.54 (td, J = 11.3, 3.9 Hz, 1H), 0.62 (s, 3H), 0.72 (s, 3H), 0.84−1.42 (m, 36H), 1.49−1.62 (m, 4H), 1.71−1.83 (m, 1H) , 1.91−1.94 (m, 1H), 2.78 (d, J = 16.0 Hz, 1H), 3.03 (d, J = 16.0 Hz, 1H), 3.24 (s, 3H), 6.83 (d, J = 7.8 Hz, 1H), 7.00−7.04 (m, 1H), 7.19 (d, J = 7.3 Hz, 1H), 7.23−7.27 (m, 1H); ^{13}C NMR (100 MHz, $CDCl_3$) δ (ppm) = 12.0, 12.1, 18.6, 21.1, 22.5, 22.8, 23.8, 24.1, 24.5, 26.3, 26.7, 27.0, 27.9, 28.1,

28.4, 31.8, 33.3, 33.6, 35.2, 35.3, 35.7, 36.1, 36.5, 39.4, 39.9, 42.1, 42.5, 44.3, 44.4, 45.6, 54.0, 56.2, 56.3, 73.7, 107.9, 122.3, 122.3, 128.0, 132.9, 143.6, 169.1, 179.9; HRMS (ESI) for $C_{39}H_{59}NO_3Na$ $[M+Na]^+$ calcd. 612.4387, found 612.4388.

(3R,5S,8R,9S,10S,13S,14S)-10,13-dimethyl-17-oxohexadecahydro-1H-cyclopenta[a]phenanthren-3-yl 2-(1,3-dimethyl-2-oxoindolin-3-yl)acetate (8x)

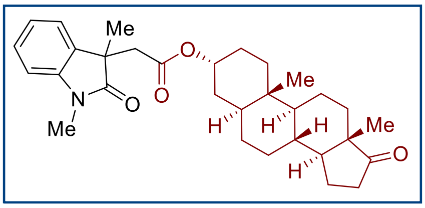

Purification by flash chromatography (*n*-hexane/ ethyl acetate = 4/1). Colorless oil; 80.0 mg, 81% yield, 1:1 dr; ^{1}H NMR (400 MHz, $CDCl_3$) δ (ppm) = 0.72 (s, 3H), 0.81−0.89 (m, 4H), 0.94−1.01 (m, 1H), 1.09−1.20 (m, 3H), 1.22−1.33 (m, 6H), 1.37 (s, 3H), 1.42−1.56 (m, 4H), 1.57−1.63 (m, 1H), 1.75−1.83 (m, 3H), 1.92−1.99 (m, 1H), 2.05−2.14 (m, 1H), 2.41−2.49 (m, 1H), 2.83−2.88 (m, 1H), 3.03−3.08 (m, 1H), 3.25 (s, 3H), 4.75−4.77 (m, 1H), 6.84 (d, *J* = 7.8 Hz, 1H), 7.03 (t, *J* = 7.6 Hz, 1H), 7.20−7.28 (m, 2H); ^{13}C NMR (100 MHz, $CDCl_3$) δ (ppm) = 11.1, 13.7, 19.9, 21.7, 25.0, 25.0, 25.7, 25.8, 26.3, 27.9, 30.5, 30.6, 31.5, 32.4, 32.5, 32.6, 34.9, 35.7, 35.8, 39.6, 39.7, 41.8, 41.9, 45.6, 45.7, 47.7, 51.5, 51.5, 53.9, 54.0, 70.4, 108.1, 108.1, 122.1, 122.3, 122.3, 128.0, 128.0, 133.0, 133.1, 143.4, 169.2, 179.7, 221.2; HRMS (ESI) for $C_{31}H_{41}NO_4Na$ $[M+Na]^+$ calcd. 514.2928, found 514.2936.

(3S,8S,9S,10R,13R,14S,17R)-10,13-dimethyl-17-((R)-6-methylheptan-2-yl)-2,3,4,7,8,9,10,11,12,13,14,15,16,17-tetradecahydro-1H-cyclopenta[a]phenanthren-3-yl 2-(1,3-dimethyl-2-oxoindolin-3-yl)acetate (8y)

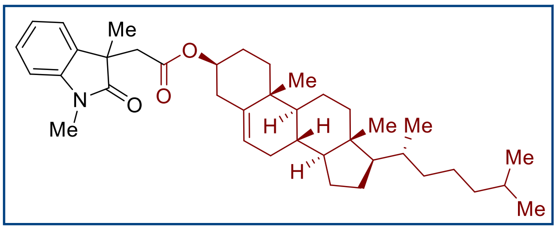

Purification by flash chromatography (*n*-hexane/ ethyl acetate = 6/1). Colorless oil; 68.0 mg, 58% yield, 1:1 dr; ^{1}H NMR (400 MHz, $CDCl_3$) δ (ppm) = 0.65 (s, 3H), 0.84−1.00 (m, 16H), 1.04−1.16 (m, 6H), 1.32−1.57 (m, 14H), 1.68−2.08 (m, 7H), 2.79 (d, *J* = 16.0 Hz, 1H), 3.02−3.06 (m, 1H), 3.25 (s, 3H), 4.26−4.37 (m, 1H), 5.22−5.26 (m, 1H), 6.84 (d, *J* = 7.7 Hz, 1H), 7.03 (t, *J* = 7.4 Hz, 1H), 7.19 (d, *J* = 7.4

Hz, 1H), 7.27 (t, J = 7.7 Hz, 1H); ^{13}C NMR (100 MHz, $CDCl_3$) δ (ppm) = 11.8, 18.7, 19.2, 20.9, 22.5, 22.8, 23.8, 24.2, 24.5, 24.5, 26.3, 27.1, 27.4, 28.0, 28.2, 31.8, 35.8, 36.1, 36.4, 36.7, 36.8, 37.4, 37.7, 39.5, 39.7, 42.1, 42.3, 45.6, 49.9, 56.1, 56.6, 74.0, 107.9, 122.3, 122.3, 122.6, 128.0, 128.1, 133.0, 133.0, 139.3, 139.3, 143.6, 169.0, 179.9, 179.9; HRMS (ESI) for $C_{39}H_{57}NO_3Na$ $[M+Na]^+$ calcd. 610.4231, found 610.4237.

(R)-2-(1,3-dioxoisoindolin-2-yl)-3-phenylpropyl 2-(1,3-dimethyl-2-oxoindolin-3-yl)acetate (8z)

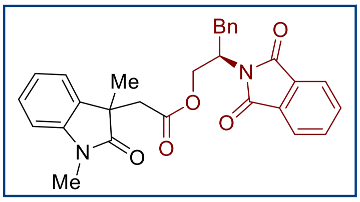

Purification by flash chromatography (n-hexane/ ethyl acetate = 3/1). Colorless oil; 77.1 mg, 80% yield, 1:1 dr; ^{1}H NMR (400 MHz, $CDCl_3$) δ (ppm) = 1.30 (s, 3H), 1.31 (s, 3H), 2.74−2.81 (m, 2H), 2.88−2.95 (m, 2H), 2.98−3.03 (m, 2H), 3.15−3.25 (m, 8H), 4.22−4.29 (m, 2H), 4.35−4.45 (m, 2H), 4.56−4.66 (m, 2H), 6.76−6.86 (m, 3H), 6.98 (td, J = 7.4, 0.6 Hz, 1H), 7.05−7.12 (m, 7H), 7.14−7.22 (m, 7H), 7.66−7.70 (m, 4H), 7.72−7.78 (m, 4H); ^{13}C NMR (100 MHz, $CDCl_3$) δ (ppm) = 24.0, 24.1, 26.2, 26.3, 34.7, 34.8, 41.1, 41.1, 45.3, 51.4, 51.5, 63.3, 63.4, 108.0, 108.0, 122.1, 122.2, 122.2, 122.3, 123.1, 123.2, 126.7, 128.0, 128.0, 128.5, 128.7, 131.5, 131.5, 132.7, 132.7, 133.9, 133.9, 136.6, 143.3, 167.9, 168.0, 169.3, 169.4, 179.5, 179.5; HRMS (ESI) for $C_{29}H_{26}N_2O_5Na$ $[M+Na]^+$ calcd. 505.1734, found 505.1740.

(S)-2-(1,3-dioxoisoindolin-2-yl)-3-phenylpropyl 2-(1,3-dimethyl-2-oxoindolin-3-yl)acetate (8aa)

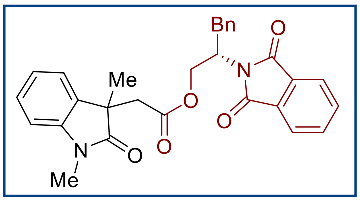

Purification by flash chromatography (n-hexane/ ethyl acetate = 3/1). Colorless oil; 79.0 mg, 82% yield, 1:1 dr; ^{1}H NMR (400 MHz, $CDCl_3$) δ (ppm) = 1.30 (s, 3H), 1.31 (s, 3H), 2.74−2.81 (m, 2H), 2.88−2.94 (m, 2H), 2.98−3.03 (m, 2H), 3.15−3.25 (m, 8H), 4.22−4.29 (m, 2H), 4.35−4.45 (m, 2H), 4.56−4.66 (m, 2H), 6.76−6.86 (m, 3H), 6.98 (td, J = 7.4, 0.6 Hz, 1H), 7.05−7.12 (m, 7H), 7.14−7.22 (m, 7H), 7.66−7.70 (m, 4H), 7.72−7.78 (m, 4H); ^{13}C NMR (100 MHz,

$CDCl_3$) δ (ppm) = 24.0, 24.1, 26.2, 26.3, 34.7, 34.8, 41.1, 41.2, 45.3, 51.5, 51.5, 63.3, 63.4, 108.0, 108.0, 122.1, 122.2, 122.3, 122.3, 123.1, 126.7, 128.0, 128.0, 128.5, 128.8, 131.5, 131.5, 132.7, 132.7, 133.9, 133.9, 136.6, 143.4, 167.9, 168.0, 169.3, 169.4, 179.5, 179.5; HRMS (ESI) for $C_{29}H_{26}N_2O_5Na$ $[M+Na]^+$ calcd. 505.1734, found 505.1734.

(1R,2R)-2-(1,3-dioxoisoindolin-2-yl)cyclohexyl 2-(1,3-dimethyl-2-oxoindolin-3-yl)acetate (8ab)

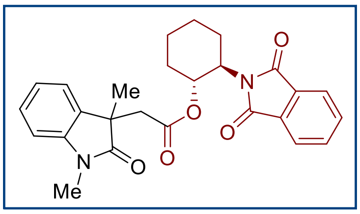

Purification by flash chromatography (*n*-hexane/ ethyl acetate = 3/1). Colorless oil; 72.5 mg, 81% yield, 1:1 dr; ^1H NMR (400 MHz, $CDCl_3$) δ (ppm) = 1.19 (s, 3H), 1.23−1.37 (m, 9H), 1.70−1.77 (m, 6H), 1.87−1.90 (m, 2H), 2.15−2.29 (m, 2H), 2.60 (d, *J* = 16.2 Hz, 1H), 2.70 (s, 2H), 2.84 (d, *J* = 16.2 Hz, 1H), 3.07 (s, 3H), 3.19 (s, 3H), 4.00−4.09 (m, 2H), 5.23−5.31 (m, 2H), 6.56 (td, J = 7.3, 0.9 Hz, 1H), 6.73−6.80 (m, 3H), 6.88 (td, J = 7.4, 0.8 Hz, 1H), 7.06−7.16 (m, 3H), 7.70−7.74 (m, 4H), 7.80−7.82 (m, 4H); ^{13}C NMR (100 MHz, $CDCl_3$) δ (ppm) = 23.6, 23.9, 24.0, 24.8, 26.1, 26.3, 28.5, 28.6, 31.1, 31.2, 41.4, 41.4, 45.2, 45.3, 53.4, 53.4, 71.8, 71.9, 107.8, 108.0, 121.6, 122.0, 122.0, 122.1, 123.1, 123.2, 127.7, 127.9, 131.7, 132.6, 132.7, 133.7, 133.8, 143.4, 143.4, 167.9, 168.0, 168.8, 169.0, 179.3, 179.5; HRMS (ESI) for $C_{26}H_{26}N_2O_5Na$ $[M+Na]^+$ calcd. 469.1734, found 469.1731.

(R)-2-(1,3-dioxoisoindolin-2-yl)-3,3-dimethylbutyl 2-(1,3-dimethyl-2-oxoindolin-3-yl)acetate (8ac)

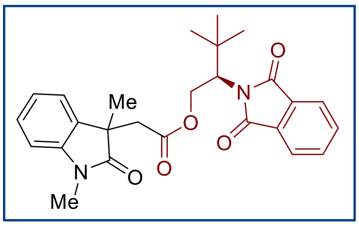

Purification by flash chromatography (*n*-hexane/ ethyl acetate = 3/1). Colorless oil; 60.1 mg, 67% yield, 1:1 dr; ^1H NMR (400 MHz, $CDCl_3$) δ (ppm) = 0.97 (s, 18H), 1.26 (s, 3H), 1.27 (s, 3H), 2.67−2.74 (m, 2H), 2.80−2.86 (m, 2H), 3.18 (s, 3H), 3.20 (s, 3H), 4.11−4.15 (m, 2H), 4.35−4.39 (m, 2H), 4.68−4.79 (m, 2H), 6.78 (d, *J* = 7.6 Hz, 2H), 6.90 (td, *J* = 7.5, 0.8 Hz, 1H), 6.98−7.06 (m, 2H), 7.12−7.23 (m, 3H), 7.73−7.77 (m, 4H), 7.81−7.88 (m, 4H); ^{13}C NMR (100 MHz, $CDCl_3$) δ (ppm) = 24.0, 24.0, 26.2, 26.3, 27.7, 27.7, 35.1, 35.2, 41.2, 45.3, 59.1, 59.2, 60.4, 60.4,

108.0, 108.0, 122.2, 122.3, 122.3, 123.1, 123.2, 123.3, 123.4, 128.0, 131.3, 131.9, 132.7, 133.9, 134.0, 134.1, 143.3, 143.4, 168.7, 168.9, 169.1, 169.5, 169.5, 179.5, 179.5; HRMS (ESI) for $C_{26}H_{28}N_2O_5Na$ $[M+Na]^+$ calcd. 471.1890, found 471.1888.

(1R,2S)-1-(1,3-dioxoisoindolin-2-yl)-2,3-dihydro-1H-inden-2-yl 2-(1,3-dimethyl-2-oxoindolin-3-yl)acetate (8ad)

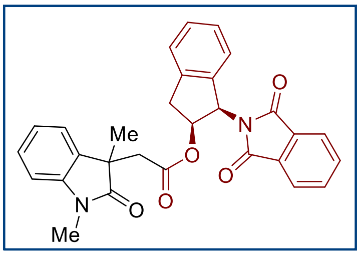

Purification by flash chromatography (*n*-hexane/ ethyl acetate = 2/1). Colorless oil; 68.9 mg, 72% yield, 1.1:1 dr; ^{1}H NMR (400 MHz, $CDCl_3$) δ (ppm) = 1.20 (s, 3H), 1.24 (s, 3H), 2.62 (d, J = 16.6 Hz, 1H), 2.70 (s, 2H), 2.86 (d, J = 16.6 Hz, 1H), 3.11−3.27 (m, 10H), 5.38−5.46 (m, 2H), 5.78 (d, J = 7.8 Hz, 1H), 5.85 (d, J = 7.7 Hz, 1H), 6.80−6.84 (m, 3H), 6.88 (dd, J = 7.6, 1.1 Hz, 1H), 6.97 (td, J = 7.5, 0.8 Hz, 1H), 7.10−7.12 (m, 3H), 7.17 (t, J = 3.6 Hz, 2H), 7.21−7.29 (m, 6H), 7.71−7.78 (m, 4H), 7.81−7.85 (m, 4H); ^{13}C NMR (100 MHz, $CDCl_3$) δ (ppm) = 23.9, 24.2, 26.3, 26.3, 37.6, 37.8, 41.0, 45.1, 45.3, 54.4, 54.5, 72.6, 73.1, 108.0, 121.9, 122.3, 122.3, 122.3, 123.3, 124.5, 124.6, 124.7, 124.7, 127.1, 127.2, 128.0, 128.0, 128.9, 129.0, 131.8, 132.6, 132.6, 134.0, 134.0, 136.5, 136.6, 140.6, 140.7, 143.4, 143.5, 167.4, 167.6, 169.0, 169.2, 179.3, 179.4; HRMS (ESI) for $C_{29}H_{24}N_2O_5Na$ $[M+Na]^+$ calcd. 503.1577, found 503.1581.

3a,8-dimethyl-3,3a,8,8a-tetrahydro-2H-furo[2,3-b]indole (9a)

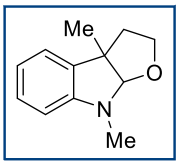

Purification by flash chromatography (*n*-hexane/ ethyl acetate = 20/1). Colorless oil; 27.6 mg, 73% yield; ^{1}H NMR (400 MHz, $CDCl_3$): δ (ppm)= 1.45 (s, 3H), 2.00–2.15 (m, 2H), 2.91 (s, 3H), 3.42–3.48 (m, 1H), 3.92–3.96 (m, 1H), 5.06 (s, 1H), 6.36 (d, J = 7.8 Hz, 1H), 6.65–6.69 (m, 1H), 7.03 (dd, J = 6.9, 0.6 Hz, 1H), 7.07–7.12 (m, 1H); ^{13}C NMR (100 MHz, $CDCl_3$) δ(ppm) = 24.7, 30.8, 41.7, 52.3, 67.3, 104.8, 105.0, 117.2, 122.4, 128.0, 134.4, 150.4; HRMS (ESI) for $C_{12}H_{16}NO$ $[M+H]^+$ calcd. 190.1226, found 190.1231.

3a,5,8-trimethyl-3,3a,8,8a-tetrahydro-2H-furo[2,3-b]indole (9b)

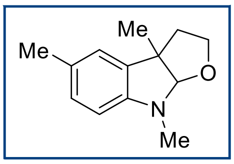

Purification by flash chromatography (*n*-hexane/ ethyl acetate = 20/1). Colorless oil; 24.4 mg, 60% yield; ^{1}H NMR (400 MHz, $CDCl_3$): δ (ppm) = 1.44 (s, 3H), 1.99–2.07 (m, 1H), 2.09–2.14 (m, 1H), 2.26 (s, 3H), 2.89 (s, 3H), 3.43–3.49 (m, 1H), 3.91–3.96 (m, 1H), 5.04 (s, 1H), 6.28 (d, *J* = 7.8 Hz, 1H), 6.86 (d, *J* = 0.4 Hz, 1H), 6.90 (dd, *J* = 7.9, 0.8 Hz, 1H); ^{13}C NMR (100 MHz, $CDCl_3$) δ(ppm) = 20.7, 24.7, 31.2, 41.6, 52.3, 67.3, 104.9, 105.4, 123.3, 126.5, 128.3, 134.7, 148.4; HRMS (ESI) for $C_{13}H_{18}NO$ $[M+H]^+$ calcd. 204.1383, found 204.1387.

5-(tert-butyl)-3a,8-dimethyl-3,3a,8,8a-tetrahydro-2H-furo[2,3-b]indole (9c)

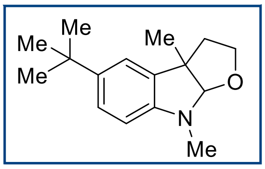

Purification by flash chromatography (*n*-hexane/ ethyl acetate = 20/1). Colorless oil; 30.4 mg, 62% yield; ^{1}H NMR (400 MHz, $CDCl_3$): δ (ppm) = 1.29 (s, 9H), 1.46 (s, 3H), 2.00–2.07 (m, 1H), 2.11–2.15 (m, 1H), 2.90 (s, 3H), 3.46–3.52 (m, 1H), 3.94 (t, *J* = 7.9 Hz, 1H), 5.06 (s, 1H), 6.31(d, *J* = 8.1 Hz, 1H), 7.08 (s, 1H), 7.12 (d, *J* = 8.1 Hz, 1H); ^{13}C NMR (100 MHz, $CDCl_3$) δ(ppm) = 24.9, 31.2, 31.8, 34.2, 41.7, 52.4, 67.3, 104.3, 105.6, 119.6, 124.5, 134.3, 140.4, 148.2; HRMS (ESI) for $C_{16}H_{24}NO$ $[M+H]^+$ calcd. 246.1852, found 246.1854.

5-(tert-butyl)-3a,8-dimethyl-3,3a,8,8a-tetrahydro-2H-furo[2,3-b]indole (9d)

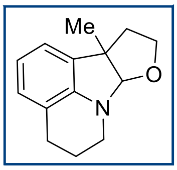

Purification by flash chromatography (*n*-hexane/ ethyl acetate = 20/1). Colorless oil; 29.3 mg, 68% yield; ^{1}H NMR (400 MHz, $CDCl_3$): δ (ppm) = 1.46 (s, 3H), 1.98–2.06 (m, 3H), 2.13 (dd, *J* = 11.8, 5.2 Hz, 1H), 2.60–2.72 (m, 2H), 3.21–3.26 (m, 1H), 3.34–3.39 (m, 1H), 3.47–3.53 (m, 1H), 3.96 (t, *J* = 7.9 Hz, 1H), 5.10 (s, 1H), 6.58 (d, *J* = 7.4 Hz, 1H), 6.82 (d, *J* = 7.5 Hz, 1H), 6.87 (d, *J* = 7.3 Hz, 1H); ^{13}C NMR (100 MHz, $CDCl_3$) δ (ppm) = 22.5, 24.2, 24.3, 41.3, 41.7, 53.4, 67.7, 103.9, 117.1, 117.2, 120.1, 126.6, 132.1, 146.8; HRMS (ESI) for $C_{14}H_{18}NO$ $[M+H]^+$ calcd. 216.1383, found 216.1384.

11a-methyl-4,5,6,7,8a,10,11,11a-octahydroazepino[3,2,1-hi]furo[2,3-b]indole (9e)

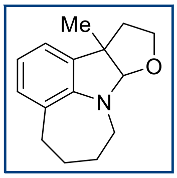

Purification by flash chromatography (*n*-hexane/ ethyl acetate = 20/1). Colorless oil; 29.8 mg, 65% yield; ^{1}H NMR (400 MHz, $CDCl_3$): δ (ppm) = 1.46 (s, 3H), 1.55–1.65 (m, 1H), 1.77–1.89 (m, 3H), 1.99–2.13 (m, 2H), 2.71–2.2.81 (m, 2H), 3.07–3.12 (m, 1H), 3.35–3.40 (m, 1H), 3.53–3.59 (m, 1H), 3.93 (t, J = 7.9 Hz, 1H), 5.01 (s, 1H), 6.72 (t, J = 7.3 Hz, 1H), 6.90–6.94 (m, 2H); ^{13}C NMR (100 MHz, $CDCl_3$) δ (ppm) = 25.8, 27.6, 30.6, 35.4, 41.7, 52.0, 52.2, 67.0, 107.8, 119.4, 120.3, 125.9, 129.3, 136.6, 150.4; HRMS (ESI) for $C_{15}H_{20}NO$ $[M+H]^+$ calcd. 230.1539, found 230.1547.

5-fluoro-3a,8-dimethyl-3,3a,8,8a-tetrahydro-2H-furo[2,3-b]indole (9f)

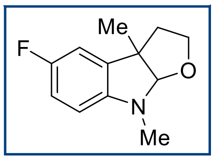

Purification by flash chromatography (*n*-hexane/ ethyl acetate = 20/1). Colorless oil; 25.0 mg, 60% yield; ^{1}H NMR (400 MHz, $CDCl_3$): δ (ppm) = 1.44 (s, 3H), 2.01–2.13 (m, 2H), 2.88 (s, 3H), 3.43–3.49 (m, 1H), 3.93–3.98 (m, 1H), 5.06 (s, 1H), 6.22–6.25 (m, 1H), 6.76–6.81 (m, 2H); ^{13}C NMR (100 MHz, $CDCl_3$) δ (ppm) = 24.4, 31.3, 41.5, 52.4 (d, J = 1.8 Hz), 67.3, 104.9 (d, J = 8.0 Hz), 105.4, 110.2 (d, J = 24.0 Hz), 113.7 (d, J = 22.8 Hz), 135.9 (d, J = 7.2 Hz), 146.6, 156.4 (d, J = 232.6 Hz); HRMS (ESI) for $C_{12}H_{15}FNO$ $[M+H]^+$ calcd. 208.1132, found 208.1140.

5-chloro-3a,8-dimethyl-3,3a,8,8a-tetrahydro-2H-furo[2,3-b]indole (9g)

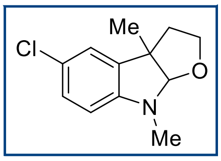

Purification by flash chromatography (*n*-hexane/ ethyl acetate = 20/1). Colorless oil; 28.4 mg, 63% yield; ^{1}H NMR (400 MHz, $CDCl_3$): δ (ppm) = 1.44 (s, 3H), 2.00–2.13 (m, 2H), 2.89 (s, 3H), 3.41–3.47 (m, 1H), 3.95 (t, J = 7.8 Hz, 1H), 5.06 (s, 1H), 6.25 (d, J = 8.3 Hz, 1H), 6.98 (s, 1H), 7.04 (d, J = 8.3 Hz, 1H); ^{13}C NMR (100 MHz, $CDCl_3$) δ (ppm) = 24.5, 30.9, 41.5, 52.3, 67.3, 105.0, 105.5, 121.8, 122.8, 127.8, 136.3, 149.0; HRMS (ESI) for $C_{12}H_{15}ClNO$ $[M+H]^+$ calcd. 224.0837, found 224.0846.

3a,8-dimethyl-5-(trifluoromethyl)-3,3a,8,8a-tetrahydro-2H-furo[2,3-b]indole (9h)

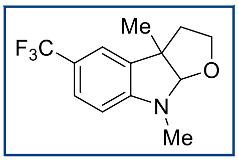

Purification by flash chromatography (*n*-hexane/ ethyl acetate = 20/1). Colorless oil; 27.1 mg, 53% yield; ^{1}H NMR (400 MHz, $CDCl_3$): δ (ppm) = 1.48 (s, 3H), 2.03–2.16 (m, 2H), 2.95 (s, 3H), 3.41–3.47 (m, 1H), 3.95–3.99 (m, 1H), 5.13 (s, 1H), 6.34 (d, *J* = 8.2 Hz, 1H), 7.22 (d, *J* = 0.2 Hz, 1H), 7.35 (td, *J* = 8.2, 0.2 Hz, 1H); ^{13}C NMR (100 MHz, $CDCl_3$) δ (ppm) = 24.6, 30.4, 41.7, 52.1, 67.2, 103.6, 104.8, 119.0 (q, *J* = 32.1 Hz), 119.6 (q, *J* = 3.6 Hz), 125.2 (q, *J* = 268.8 Hz), 126.2 (q, *J* = 3.9 Hz), 134.8, 152.8; HRMS (ESI) for $C_{13}H_{15}F_3NO$ $[M+H]^+$ calcd. 258.1100, found 258.1103.

6b,10-dimethyl-6b,7,9a,10-tetrahydro-8H-benzo[g]furo[2,3-b]indole (9i)

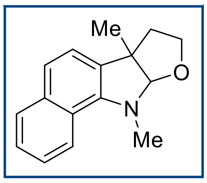

Purification by flash chromatography (*n*-hexane/ ethyl acetate = 20/1). White solid; 31.1 mg, 65% yield, m.p. = 68-70 °C; ^{1}H NMR (400 MHz, $CDCl_3$): δ (ppm) = 1.43 (s, 3H), 2.21–2.29 (m, 1H), 2.54–2.59 (m, 1H), 3.20 (s, 3H), 3.49–3.56 (m, 1H), 3.80–3.84 (m, 1H), 4.86 (s, 1H), 6.63 (d, *J* = 7.6 Hz, 1H), 7.24–7.27 (m, 1H), 7.34–7.38 (m, 2H), 7.44 (t, *J* = 7.6 Hz, 1H), 7.66 (d, *J* = 8.1 Hz, 1H); ^{13}C NMR (100 MHz, $CDCl_3$) δ (ppm) = 26.5, 38.7, 41.0, 44.3, 64.1, 97.7, 105.5, 117.8, 120.5, 120.9, 126.1, 126.2, 126.8, 133.8, 137.1, 141.1; HRMS (ESI) for $C_{16}H_{18}NO$ $[M+H]^+$ calcd. 240.1383, found 240.1389.

8-ethyl-3a-methyl-3,3a,8,8a-tetrahydro-2H-furo[2,3-b]indole (9j)

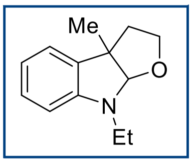

Purification by flash chromatography (*n*-hexane/ ethyl acetate = 20/1). Colorless oil; 27.6 mg, 68% yield; ^{1}H NMR (400 MHz, $CDCl_3$): δ (ppm) = 1.23 (t, *J* = 7.3 Hz, 3H), 1.45 (s, 3H), 2.00–2.07 (m, 1H), 2.10–2.15 (m, 1H), 3.28–3.42 (m, 2H), 3.43–3.50 (m, 1H), 3.93 (t, *J* = 7.9 Hz, 1H), 5.17 (s, 1H), 6.37 (d, *J* = 7.8 Hz, 1H), 6.65 (t, *J* = 7.3 Hz, 1H), 7.03–7.10 (m, 2H); ^{13}C NMR (100 MHz, $CDCl_3$) δ(ppm) = 12.9, 25.0, 38.9, 41.9, 52.2, 66.9, 103.3, 104.8, 117.0, 122.6, 127.9, 134.6, 149.5; HRMS (ESI) for $C_{13}H_{18}NO$ $[M+H]^+$ calcd. 204.1383, found 204.1388.

8-isopropyl-3a-methyl-3,3a,8,8a-tetrahydro-2H-furo[2,3-b]indole (9k)

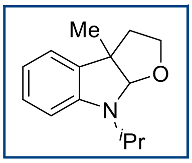

Purification by flash chromatography (n-hexane/ ethyl acetate = 20/1). Colorless oil; 23.6 mg, 54% yield; ^{1}H NMR (400 MHz, $CDCl_3$): δ (ppm) = 1.31 (d, J = 6.7 Hz, 6H), 1.43 (s, 3H), 1.99–2.07 (m, 1H), 2.11–2.15 (m, 1H), 3.43–3.50 (m, 1H), 3.78–3.92 (m, 2H), 5.28 (s, 1H), 6.40 (d, J = 7.8 Hz, 1H), 6.64 (t, J = 7.3 Hz, 1H), 7.02–7.09 (m, 2H); ^{13}C NMR (100 MHz, $CDCl_3$) δ(ppm) = 19.3, 21.1, 25.2, 42.0, 45.5, 52.2, 66.6, 100.7, 105.4, 116.8, 122.7, 127.9, 134.8, 149.2; HRMS (ESI) for $C_{14}H_{20}NO$ $[M+H]^+$ calcd. 218.1539, found 218.1546.

3a-benzyl-8-methyl-3,3a,8,8a-tetrahydro-2H-furo[2,3-b]indole (9l)

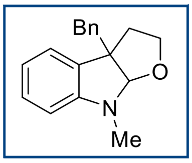

Purification by flash chromatography (n-hexane/ ethyl acetate = 20/1). Colorless oil; 37.9 mg, 71% yield; ^{1}H NMR (400 MHz, $CDCl_3$): δ (ppm) = 2.08–2.12 (m, 1H), 2.24–2.32 (m, 1H), 2.82 (s, 3H), 2.95 (d, J = 13.4 Hz, 1H), 3.13 (d, J = 13.4 Hz, 1H), 3.35–3.42 (m, 1H), 3.86–3.93 (m, 1H), 5.19 (s, 1H), 6.32 (d, J = 7.8 Hz, 1H), 6.62 (td, J = 7.4, 0.8 Hz, 1H), 6.76 (dd, J = 7.3, 0.8 Hz, 1H), 7.02–7.04 (m, 2H), 7.09 (td, J = 7.6, 1.2 Hz, 1H), 7.17–7.24 (m, 3H); ^{13}C NMR (100 MHz, $CDCl_3$) δ(ppm) = 30.9, 39.4, 43.5, 57.3, 67.4, 102.5, 105.2, 117.1, 123.7, 126.4, 127.9, 128.3, 130.1, 132.2, 137.8, 151.2; HRMS (ESI) for $C_{18}H_{20}NO$ $[M+H]^+$ calcd. 266.1539, found 266.1546.

5-methoxy-3a,8-dimethyl-3,3a,8,8a-tetrahydro-2H-furo[2,3-b]indole (9m)

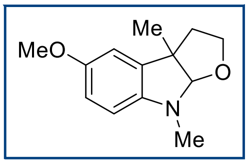

Purification by flash chromatography (n-hexane/ ethyl acetate = 20/1). Colorless oil; 20.2 mg, 46% yield; ^{1}H NMR (400 MHz, $CDCl_3$): δ (ppm) = 1.44 (s, 3H), 2.00–2.08 (m, 1H), 2.10–2.15 (m, 1H), 2.87 (s, 3H), 3.44–3.50 (m, 1H), 3.75 (s, 3H), 3.92–3.97 (m, 1H), 5.03 (s, 1H), 6.28 (d, J = 8.3 Hz, 1H), 6.64–6.70 (m, 2H); ^{13}C NMR (100 MHz, $CDCl_3$) δ(ppm) = 24.5, 31.6, 41.4, 52.5, 56.1, 67.4, 105.3, 105.6, 110.4, 112.2, 136.0, 145.0, 152.7; HRMS (ESI) for $C_{13}H_{18}NO_2$ $[M+H]^+$ calcd. 220.1332, found 220.1334.

ethyl (*E*)-3-(4-((1H-imidazol-1-yl)methyl)phenyl)acrylate (10i)

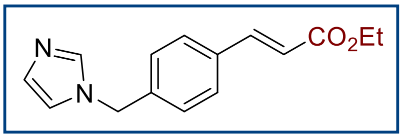

Purification by flash chromatography (DCM/EtOH = 30/1) afforded **10i**. White solid; 49.5 mg, 70% yield, m.p. = 50-52 °C; **^{1}H NMR (400 MHz, CDCl$_3$)** δ (ppm) = 1.34 (t, *J* = 7.2 Hz, 3H), 4.26 (q, *J* = 7.1 Hz, 2H), 5.15 (s, 2H), 6.43 (d, *J* = 16.1 Hz, 1H), 6.93 (s, 1H), 7.10−7.17 (m, 3H), 7.51 (d, *J* = 8.0 Hz, 2H), 7.58−7.67 (m, 2H); **^{13}C NMR (100 MHz, CDCl$_3$)** δ (ppm) = 14.2, 50.4, 60.5, 118.9, 119.3, 127.6, 128.5, 129.8, 134.4, 137.4, 138.1, 143.4, 166.7; HRMS (ESI) for $C_{15}H_{17}N_2O_2$ $[M+H]^+$ calcd. 257.1285, found 257.1289.

ethyl 6-chloro-3-phenylhex-3-enoate (11b)

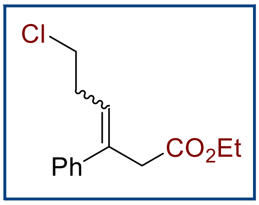

Purification by flash chromatography (PE/EA = 20/1) afforded **8b**. Colorless oil; 11.6 mg, 23% yield; **^{1}H NMR (400 MHz, CDCl$_3$)** δ (ppm) = 1.20 (t, *J* = 7.2 Hz, 3H), 2.72 (q, *J* = 7.1 Hz, 2H), 3.53 (s, 2H), 3.64 (t, *J* = 7.0 Hz, 2H), 4.11 (q, *J* = 7.2 Hz, 2H), 5.96 (t, *J* = 7.2 Hz, 1H), 7.24−7.27 (m, 1H), 7.32 (t, *J* = 7.1 Hz, 2H), 7.37−7.40 (m, 2H); **^{13}C NMR (100 MHz, CDCl$_3$)** δ (ppm) = 14.1, 14.2, 21.0, 32.3, 36.3, 43.8, 60.4, 60.9, 126.0, 127.3, 127.6, 128.3, 135.3, 141.8, 171.0, 171.2; HRMS (ESI) for $C_{14}H_{17}ClO_2Na$ $[M+Na]^+$ calcd. 275.0809, found 275.0822.

ethyl 3-cyclopropyl-3-phenylacrylate (11c)

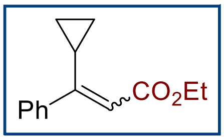

Purification by flash chromatography (PE/EA = 20/1) afforded **8c**. Colorless oil; 8.7 mg, 20% yield; **^{1}H NMR (400 MHz, CDCl$_3$)** δ (ppm) = 0.58−0.62 (m, 2H), 0.80−0.85 (m, 2H), 1.05 (t, *J* = 7.1 Hz, 3H), 1.68−1.75 (m, 1H), 3.95 (q, *J* = 7.1 Hz, 2H), 5.86 (s, 1H), 7.09−7.11 (m, 2H), 7.28−7.36 (m, 3H); **^{13}C NMR (100 MHz, CDCl$_3$)** δ (ppm) = 6.9, 13.9, 14.1, 19.7, 21.0, 59.6, 60.4, 115.0, 127.4, 127.5, 127.6, 138.1, 161.8, 165.8, 171.2; HRMS (ESI) for $C_{14}H_{16}O_2Na$ $[M+Na]^+$ calcd. 239.1043, found 239.1053.

HPLC traces of compounds

HPLC traces of **3u**

CHIRALPAK IB-H, n-hexane/*i*-PrOH, 98:2, 1.0 mL/min, 210 nm

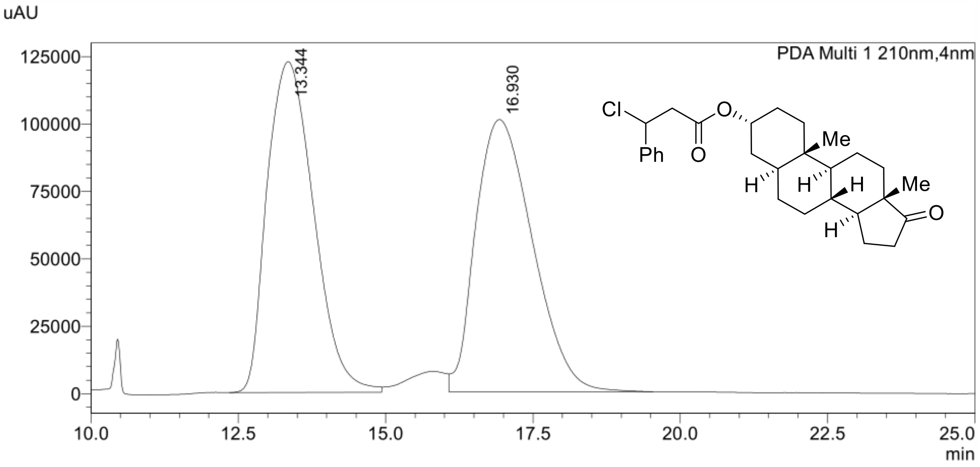

<Peak Table>

PDA Ch1 210nm

Peak#	Ret. Time	Area	Height	Aera%
1	13.344	6758161	122637	49.812
2	16.930	6809124	101029	50.188

Supplementary Figure 17. HPLC traces of **3u**

HPLC traces of **3v**

CHIRALPAK IB-H, n-hexane/*i*-PrOH, 98:2, 1.0 mL/min, 210 nm

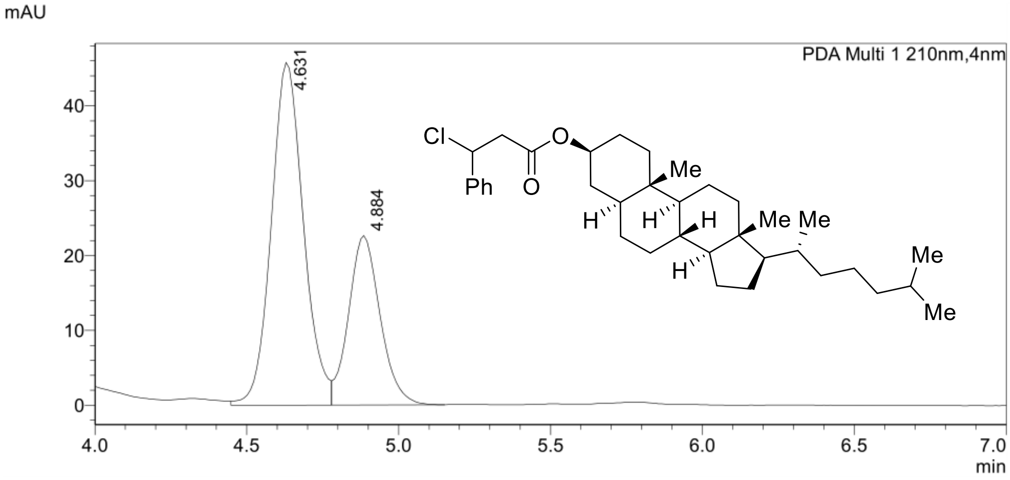

<Peak Table>

PDA Ch1 210nm

Peak#	Ret. Time	Area	Height	Aera%
1	4.631	337711	45745	67.505
2	4.884	162566	22613	32.495

Supplementary Figure 18. HPLC traces of **3v**

NMR spectra of compounds

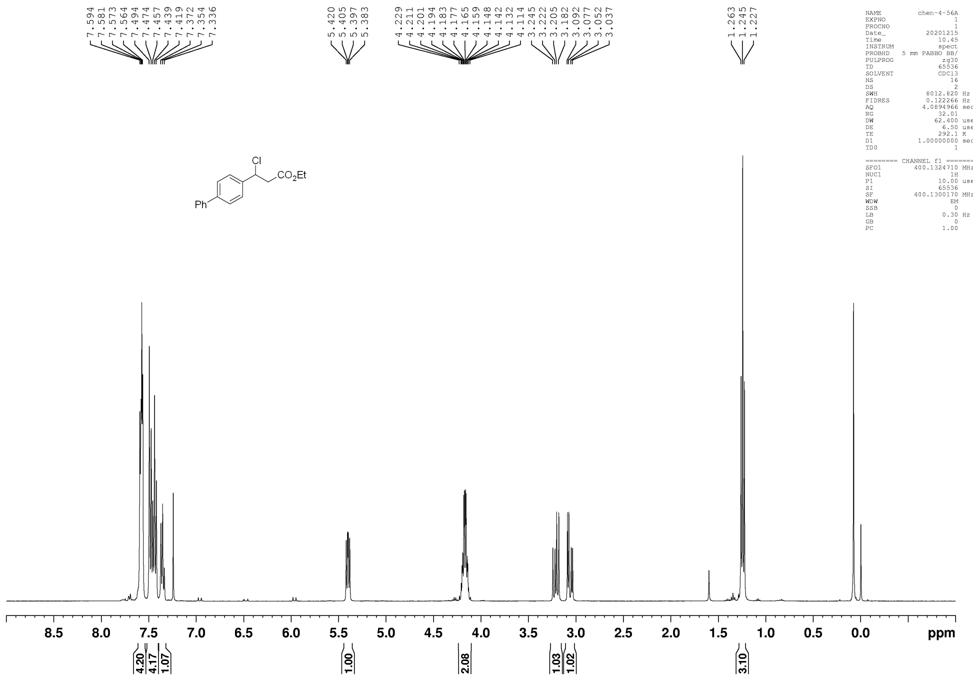

Supplementary Figure 19. ^{1}H NMR spectrum for compound **3a**

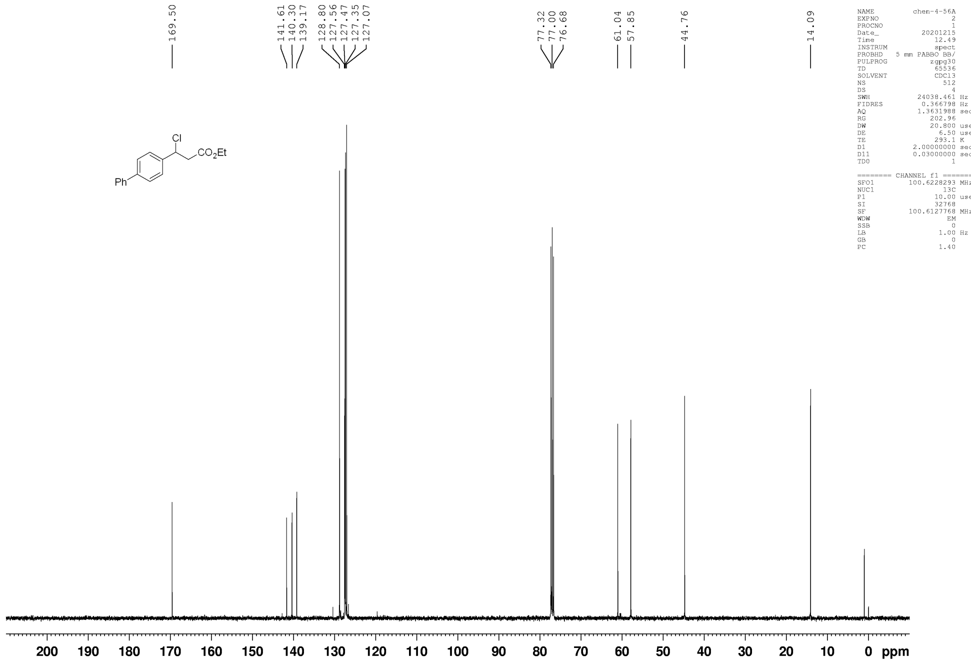

Supplementary Figure 20. ^{13}C NMR spectrum for compound **3a**

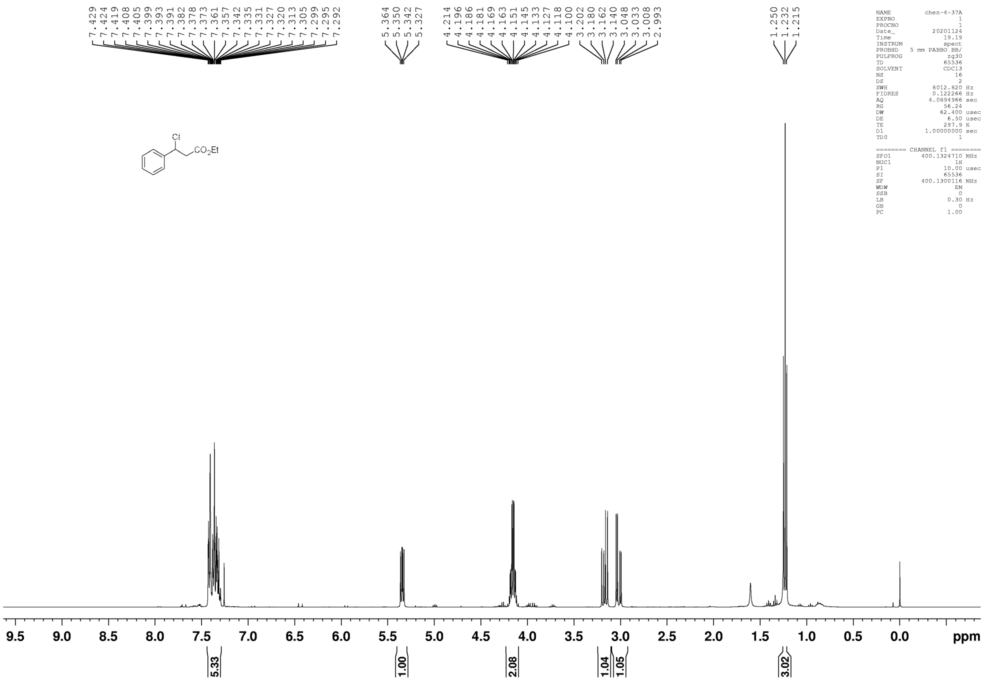

Supplementary Figure 21. ^{1}H NMR spectrum for compound **3b**

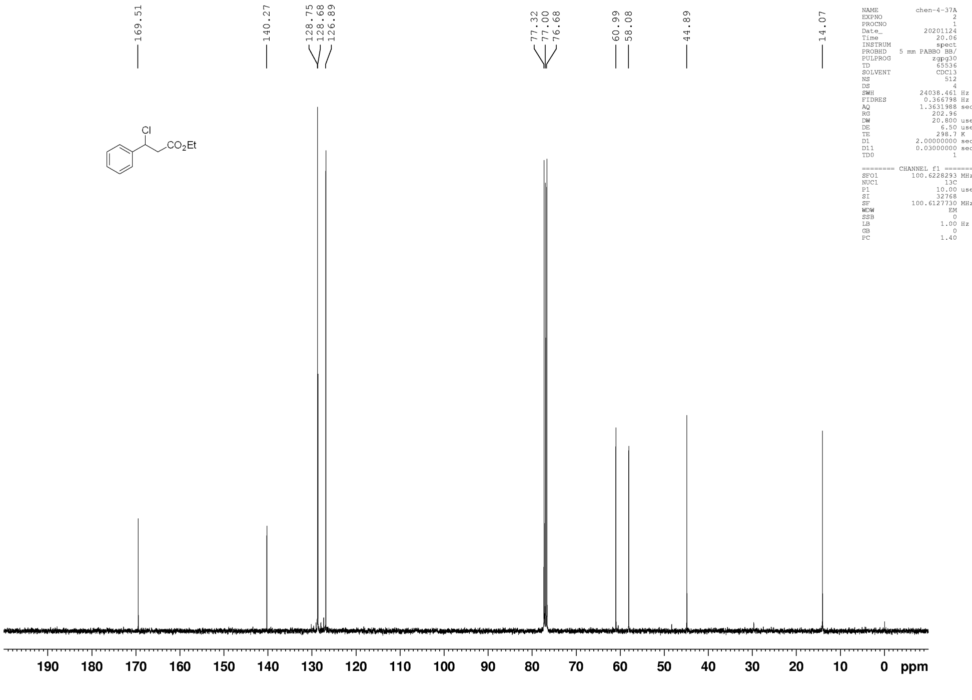

Supplementary Figure 22. ^{13}C NMR spectrum for compound **3b**

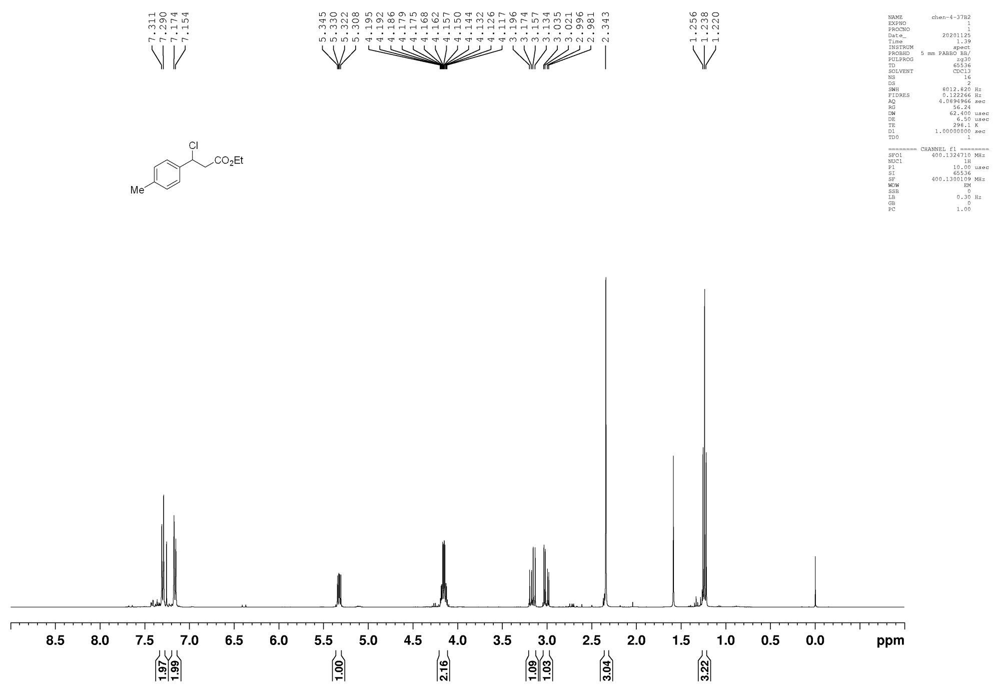

Supplementary Figure 23. ^{1}H NMR spectrum for compound **3c**

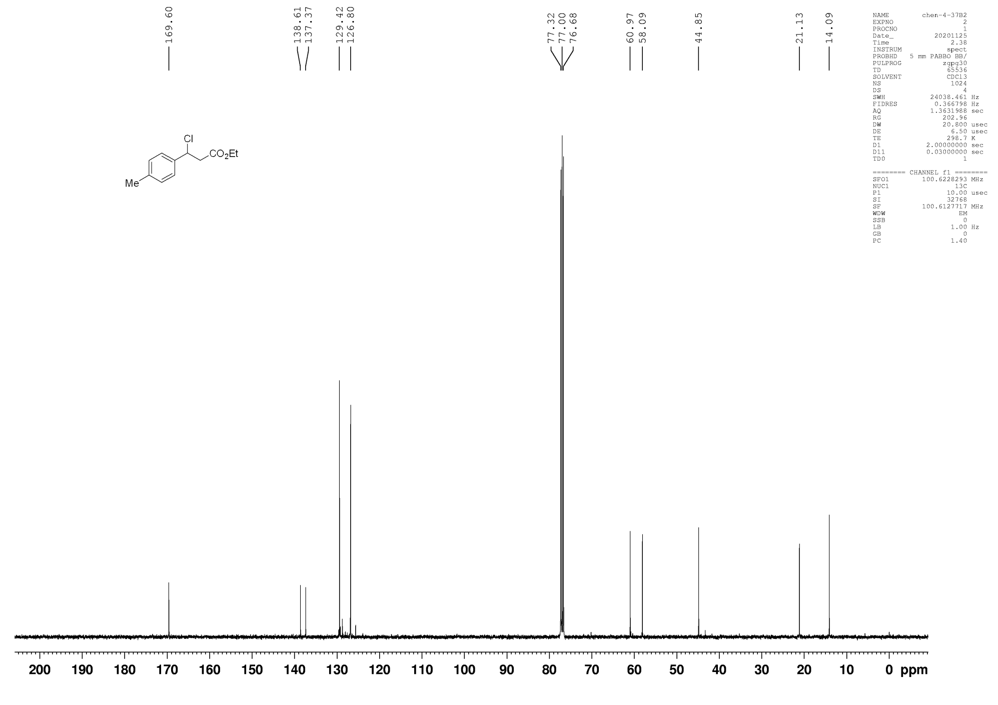

Supplementary Figure 24. ^{13}C NMR spectrum for compound **3c**

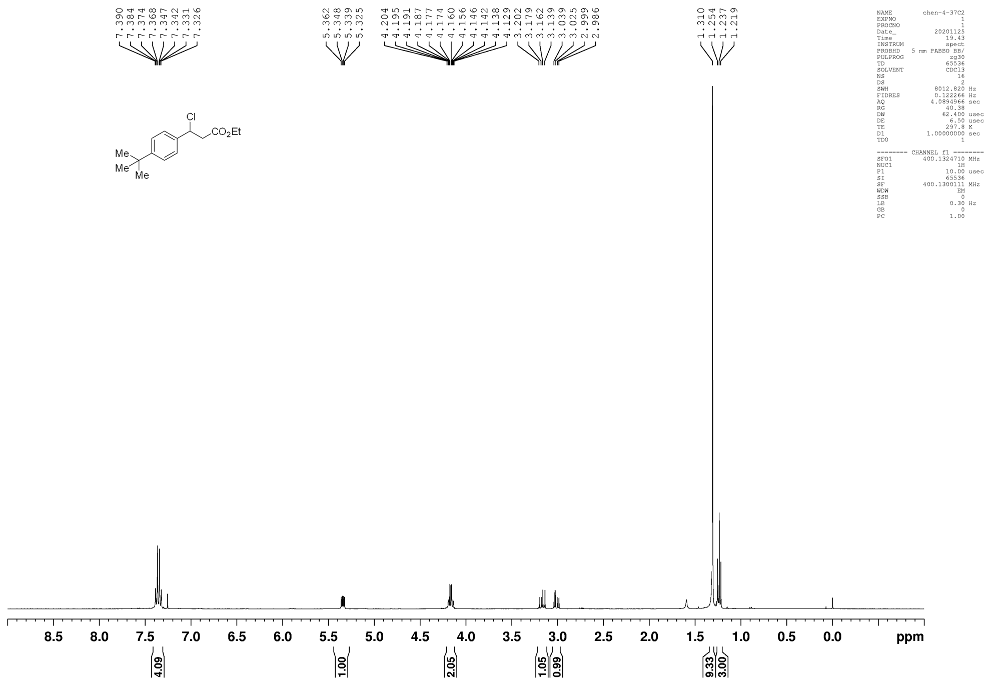

Supplementary Figure 25. ^{1}H NMR spectrum for compound **3d**

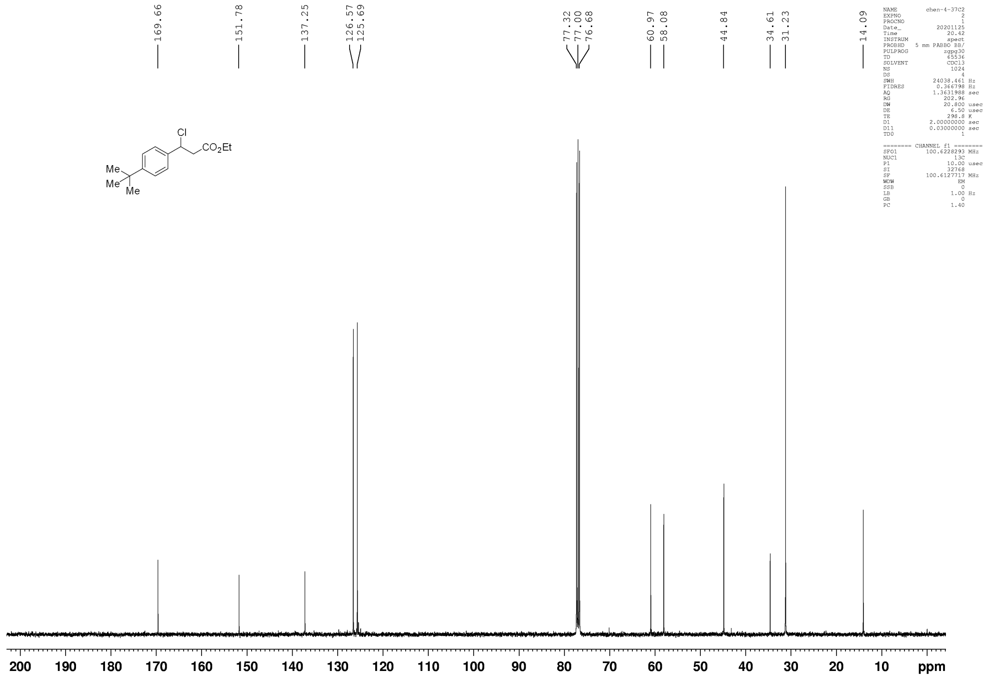

Supplementary Figure 26. ^{13}C NMR spectrum for compound **3d**

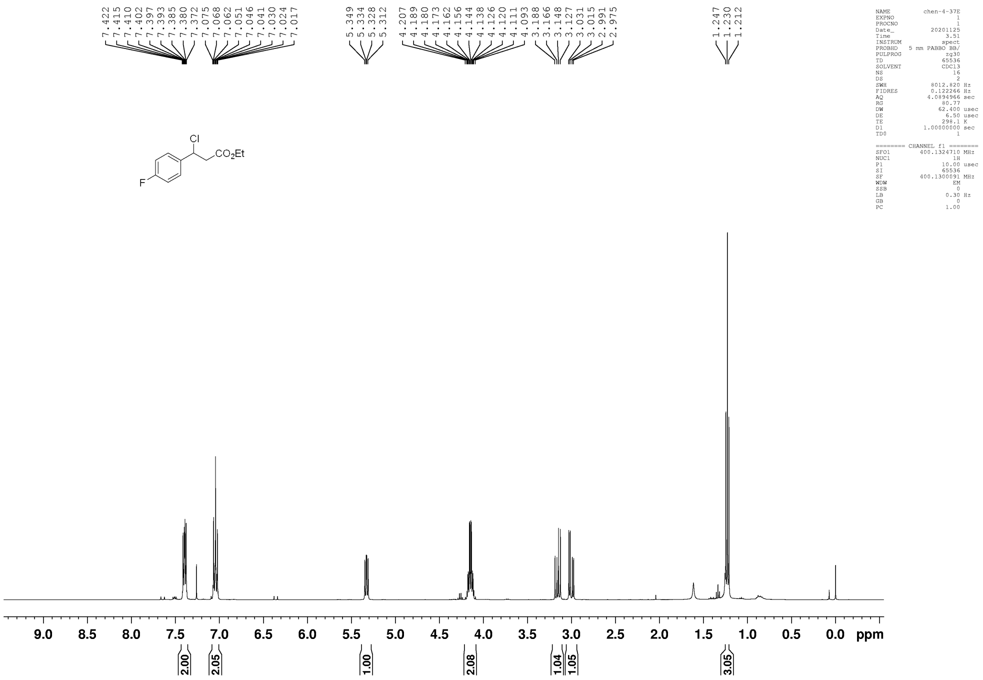

Supplementary Figure 27. ^{1}H NMR spectrum for compound **3e**

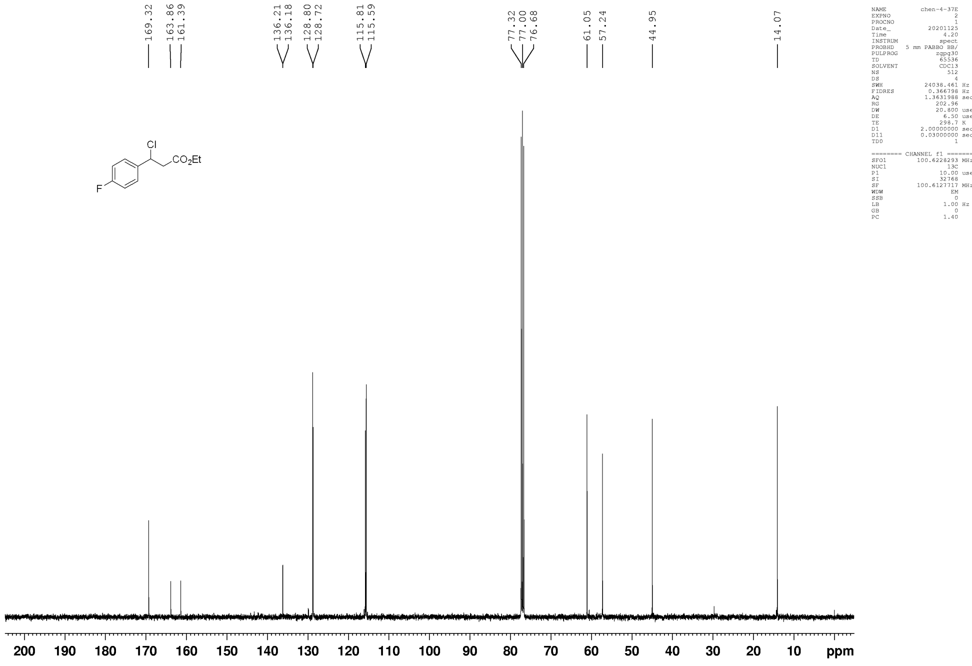

Supplementary Figure 28. ^{13}C NMR spectrum for compound **3e**

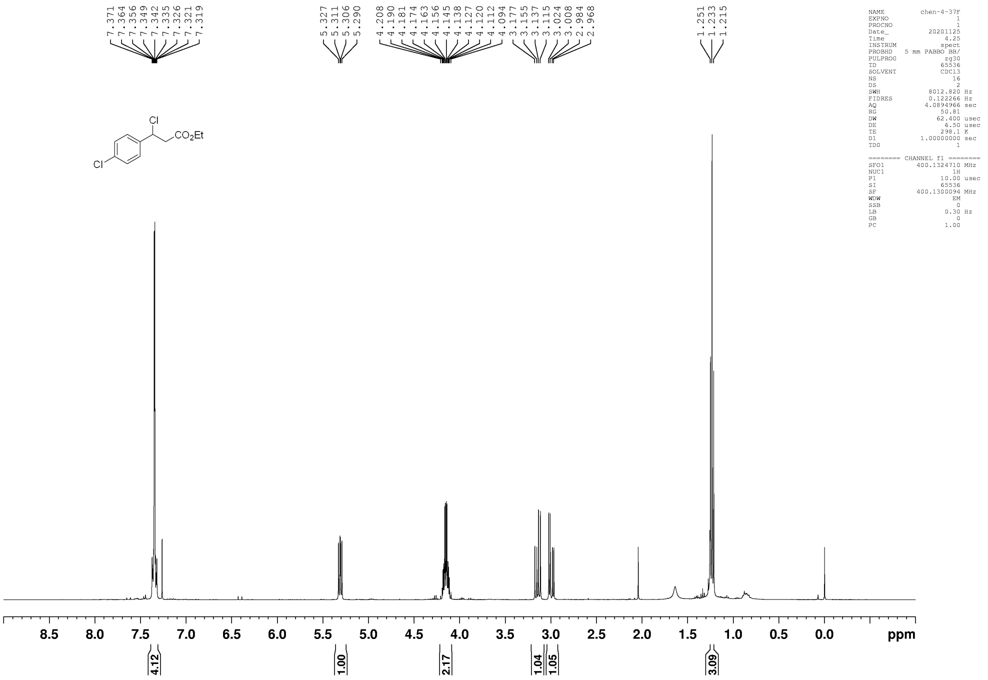

Supplementary Figure 29. ^{1}H NMR spectrum for compound **3f**

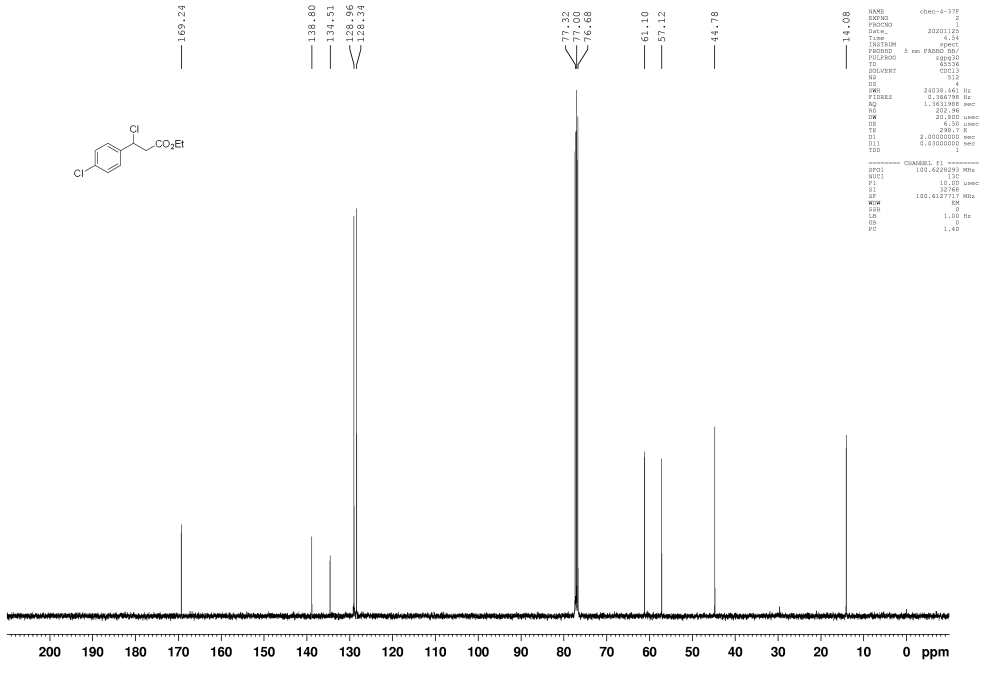

Supplementary Figure 30. ^{13}C NMR spectrum for compound **3f**

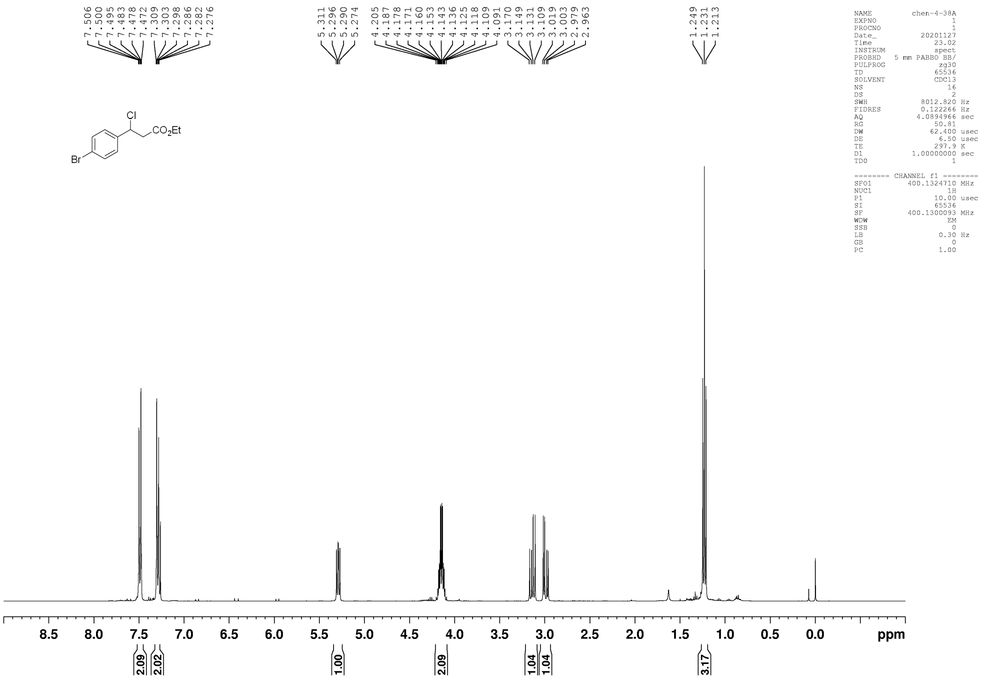

Supplementary Figure 31. ^{1}H NMR spectrum for compound **3g**

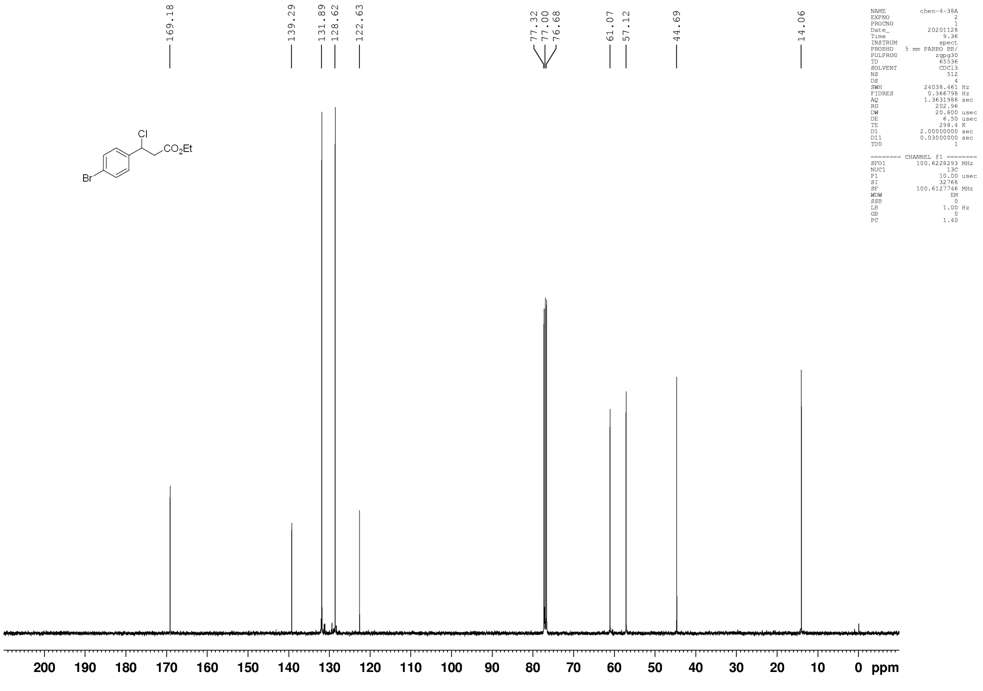

Supplementary Figure 32. ^{13}C NMR spectrum for compound **3g**

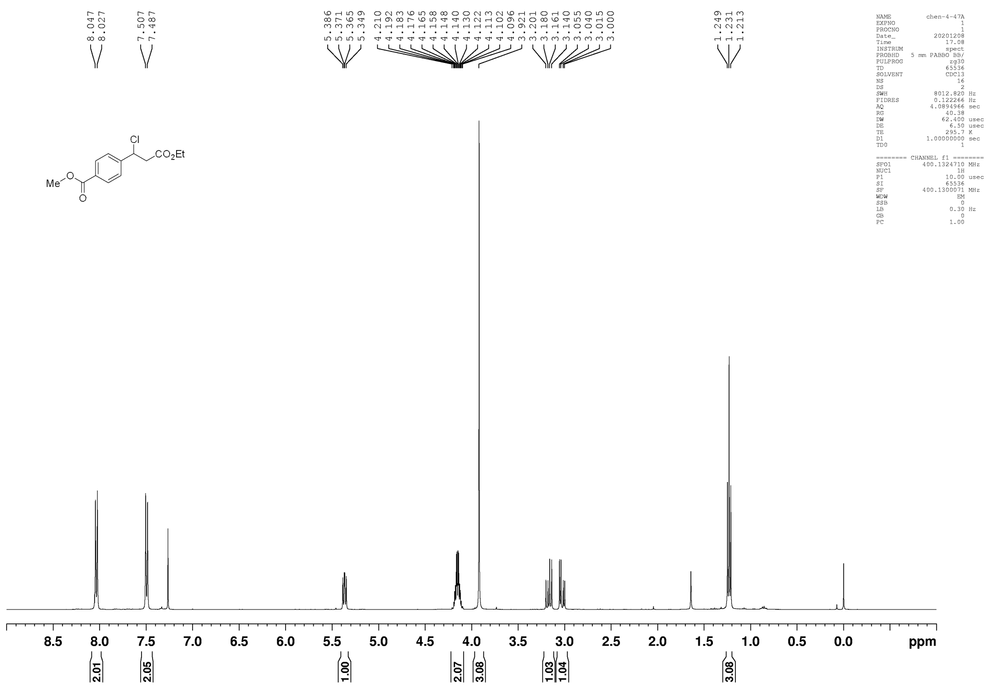

Supplementary Figure 33. ^{1}H NMR spectrum for compound **3h**

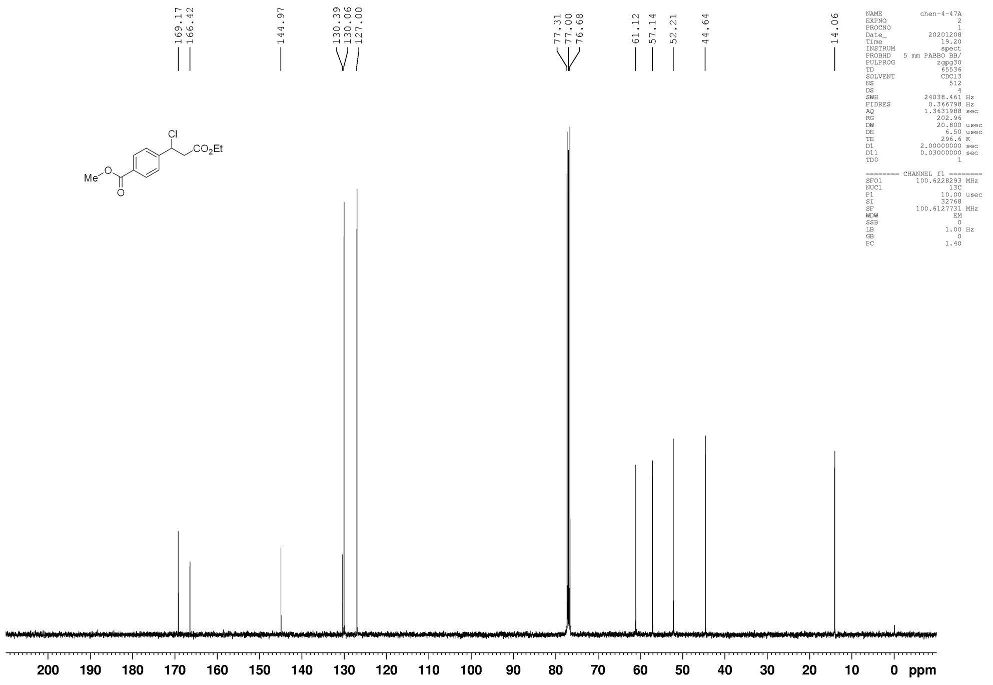

Supplementary Figure 34. ^{13}C NMR spectrum for compound **3h**

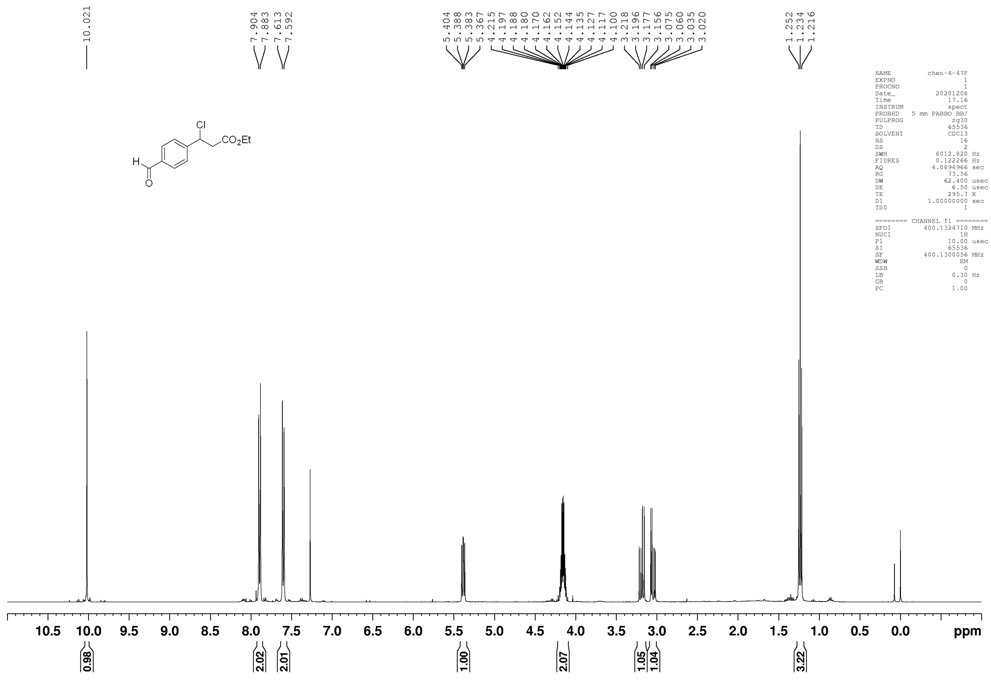

Supplementary Figure 35. ^{1}H NMR spectrum for compound **3i**

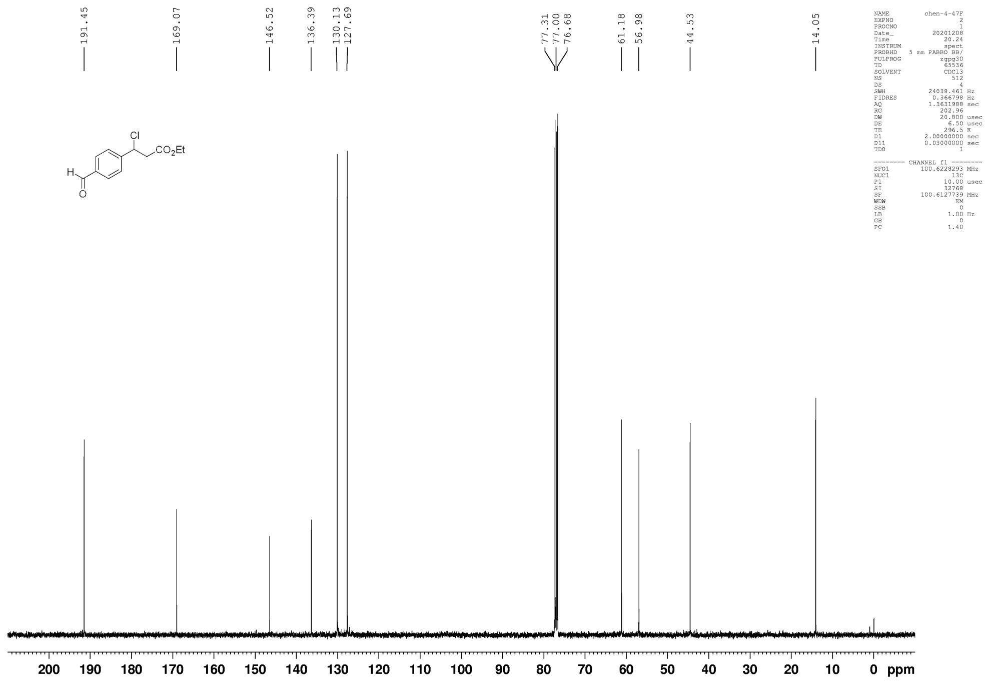

Supplementary Figure 36. ^{13}C NMR spectrum for compound **3i**

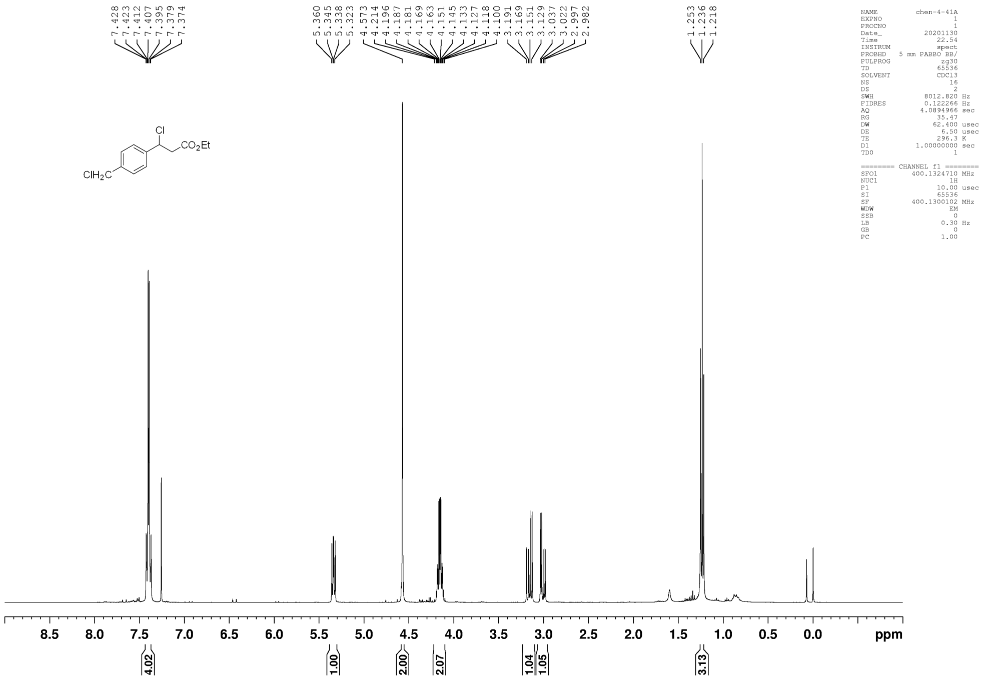

Supplementary Figure 37. ^{1}H NMR spectrum for compound **3j**

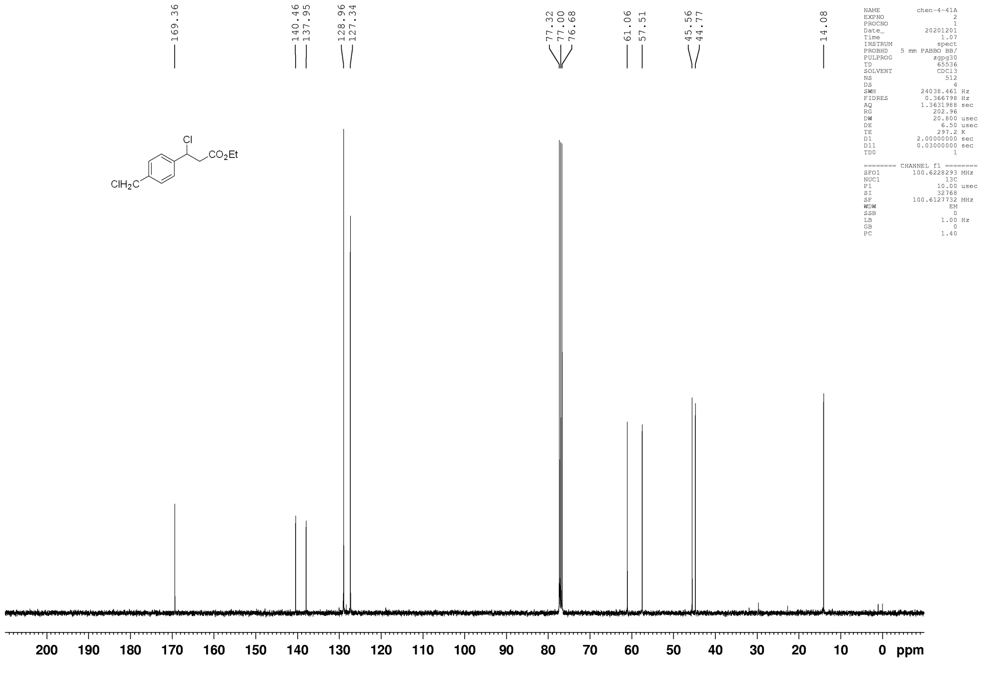

Supplementary Figure 38. ^{13}C NMR spectrum for compound **3j**

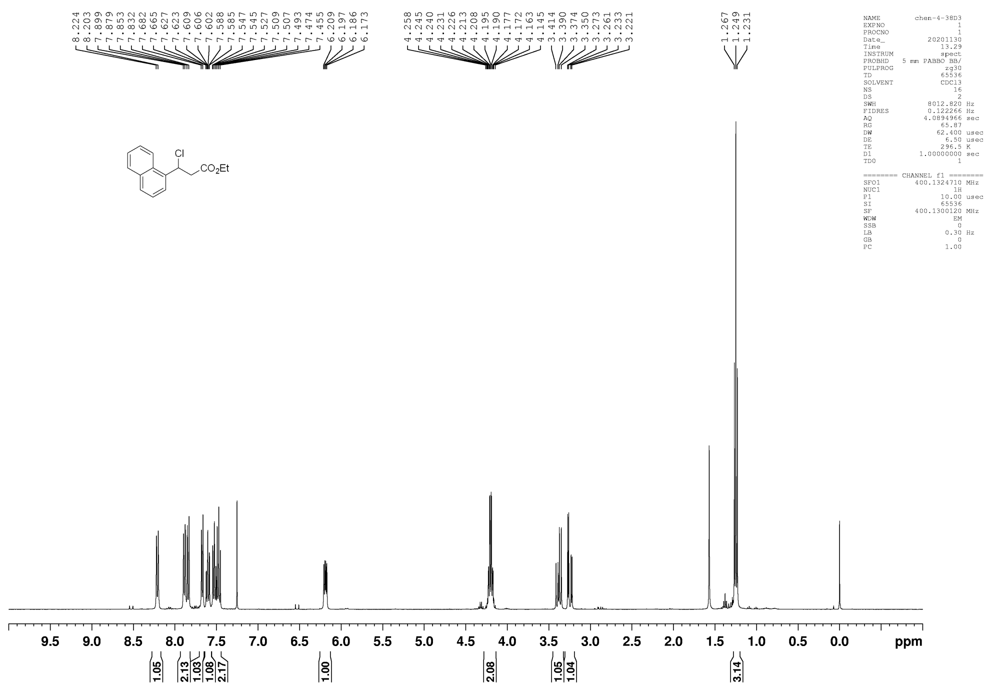

Supplementary Figure 39. ^{1}H NMR spectrum for compound **3k**

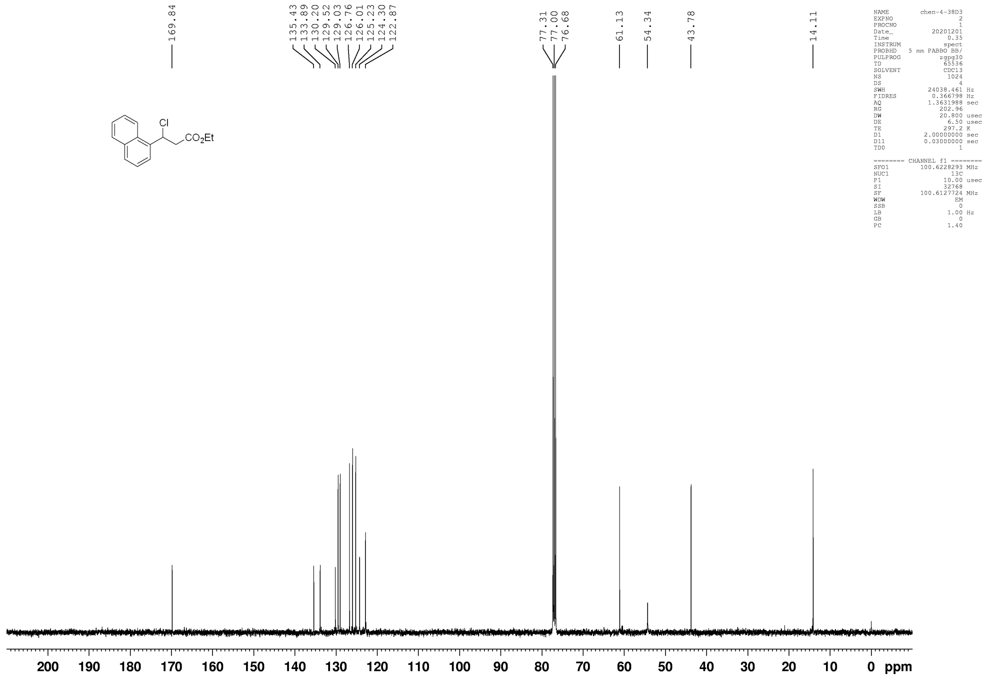

Supplementary Figure 40. ^{13}C NMR spectrum for compound **3k**

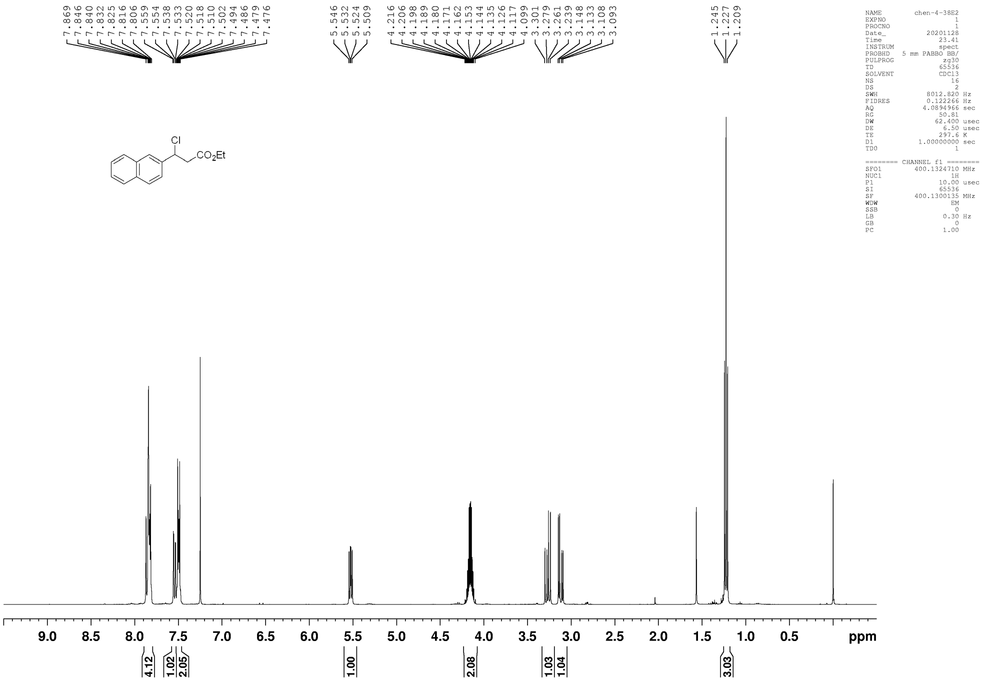

Supplementary Figure 41. ^{1}H NMR spectrum for compound **3l**

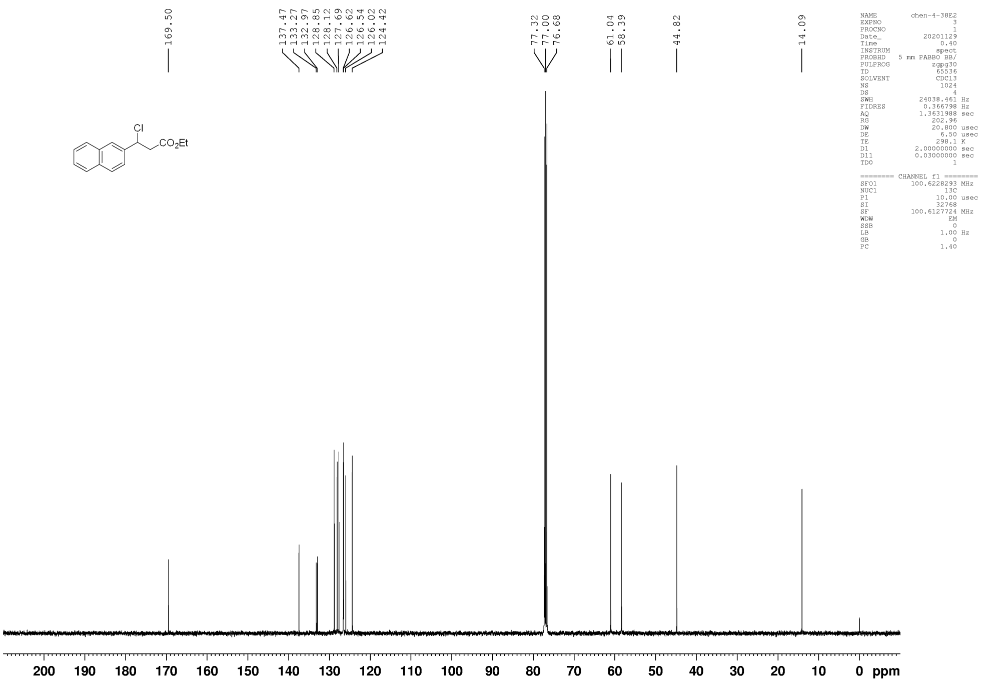

Supplementary Figure 42. ^{13}C NMR spectrum for compound **3l**

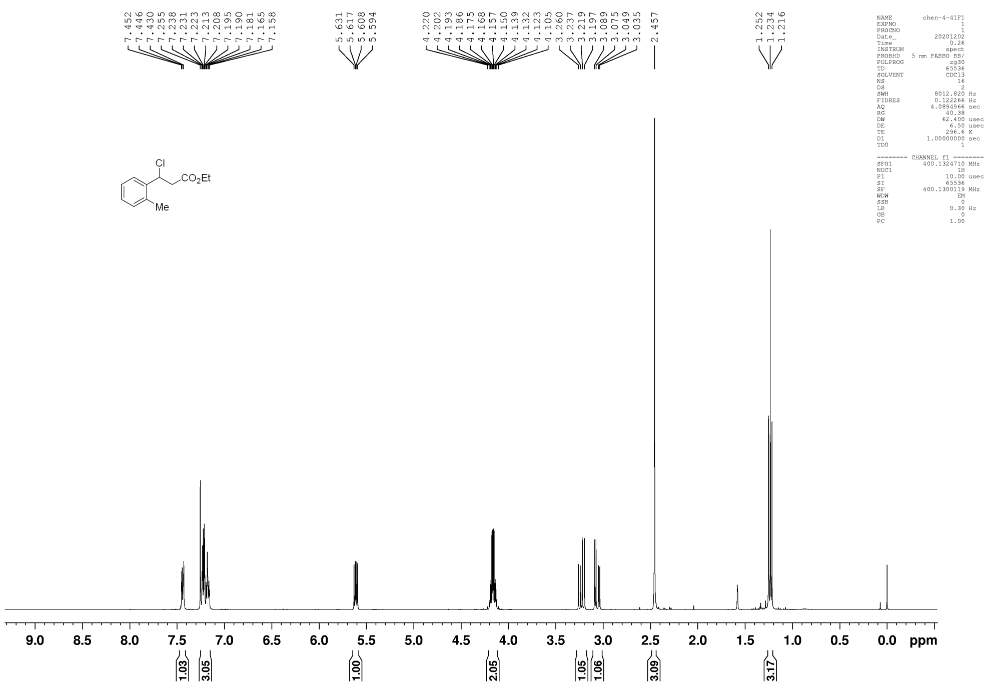

Supplementary Figure 43. ^{1}H NMR spectrum for compound **3m**

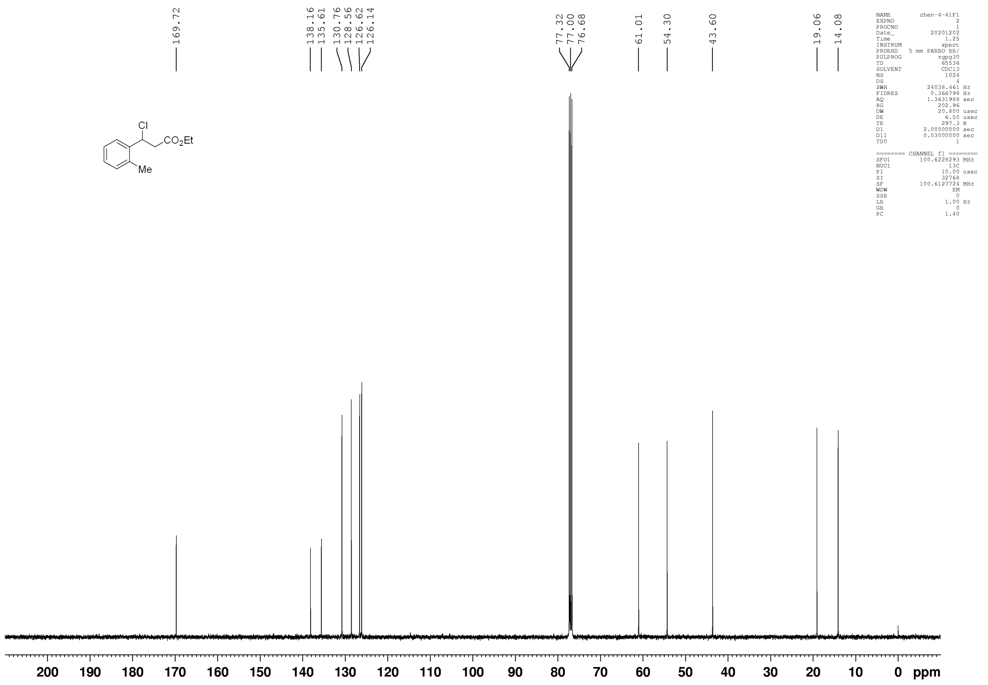

Supplementary Figure 44. ^{13}C NMR spectrum for compound **3m**

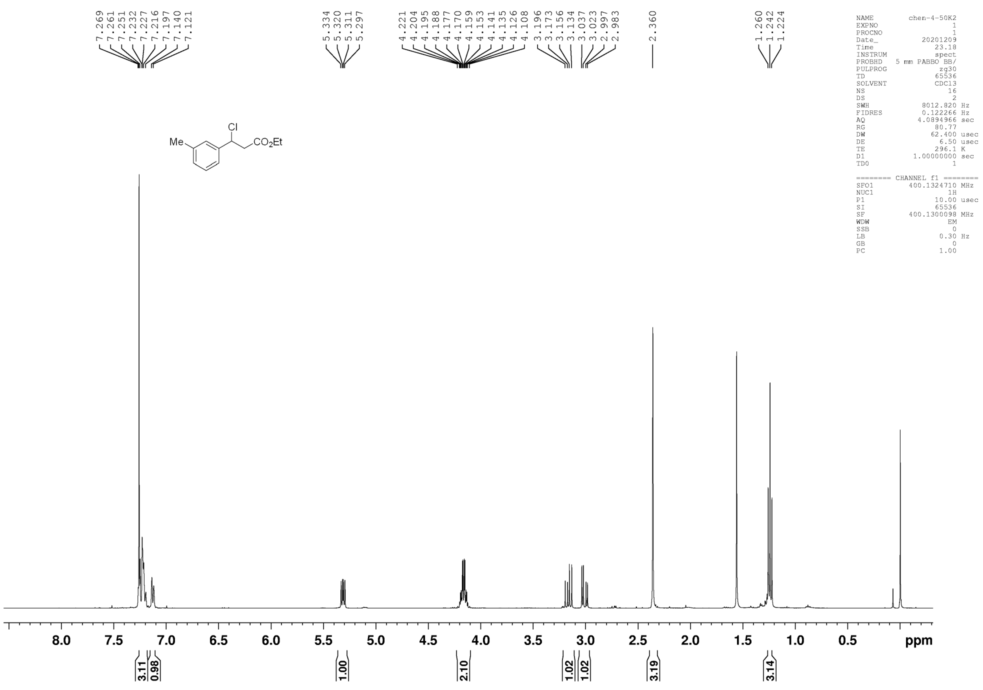

Supplementary Figure 45. ^{1}H NMR spectrum for compound **3n**

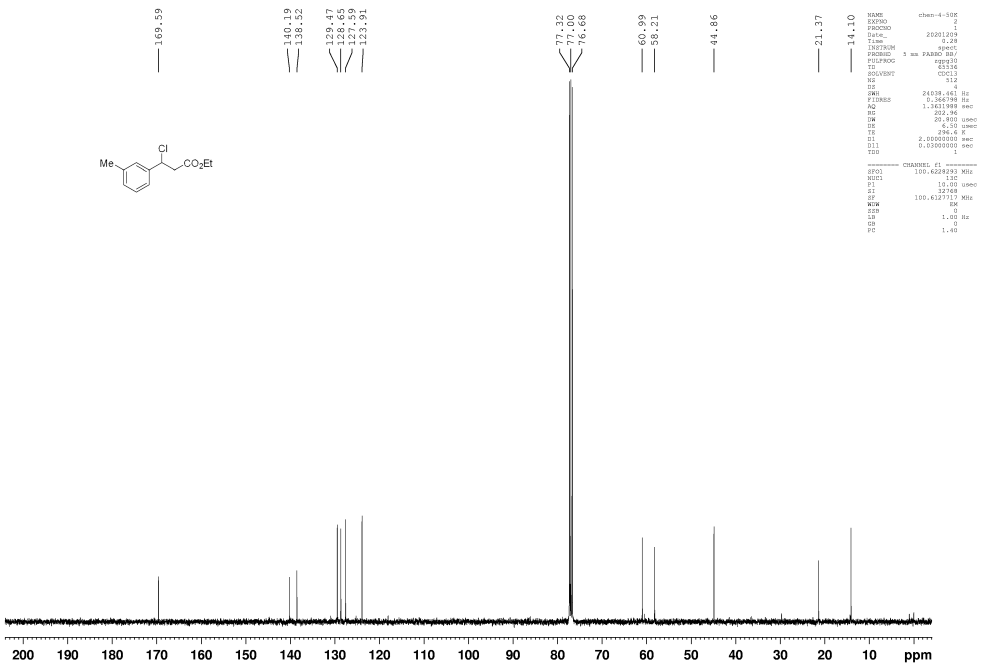

Supplementary Figure 46. ^{13}C NMR spectrum for compound **3n**

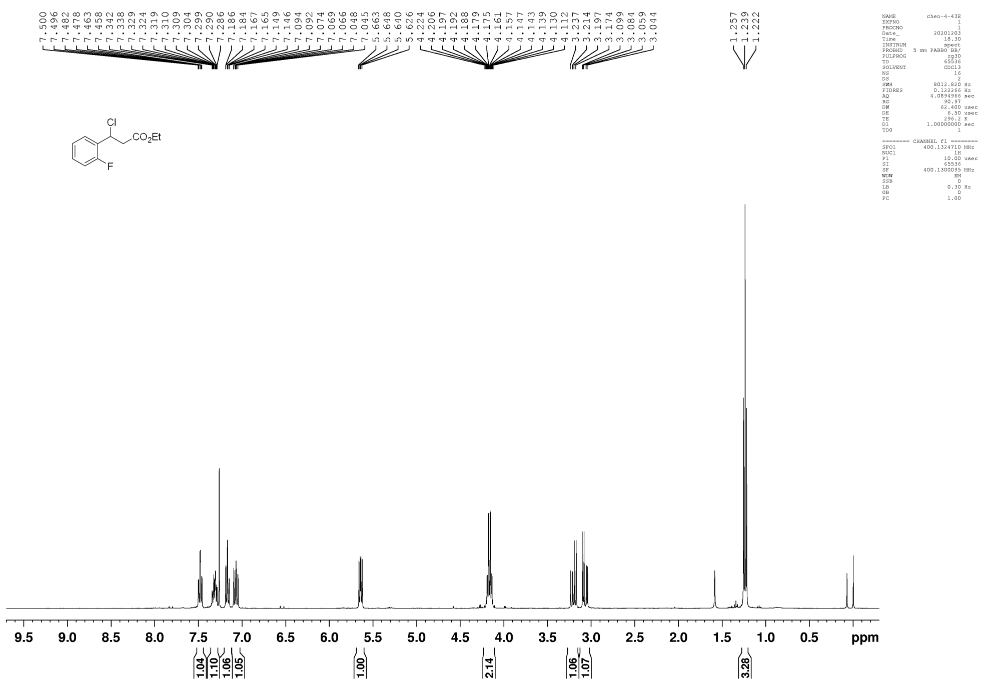

Supplementary Figure 47. ^{1}H NMR spectrum for compound **3o**

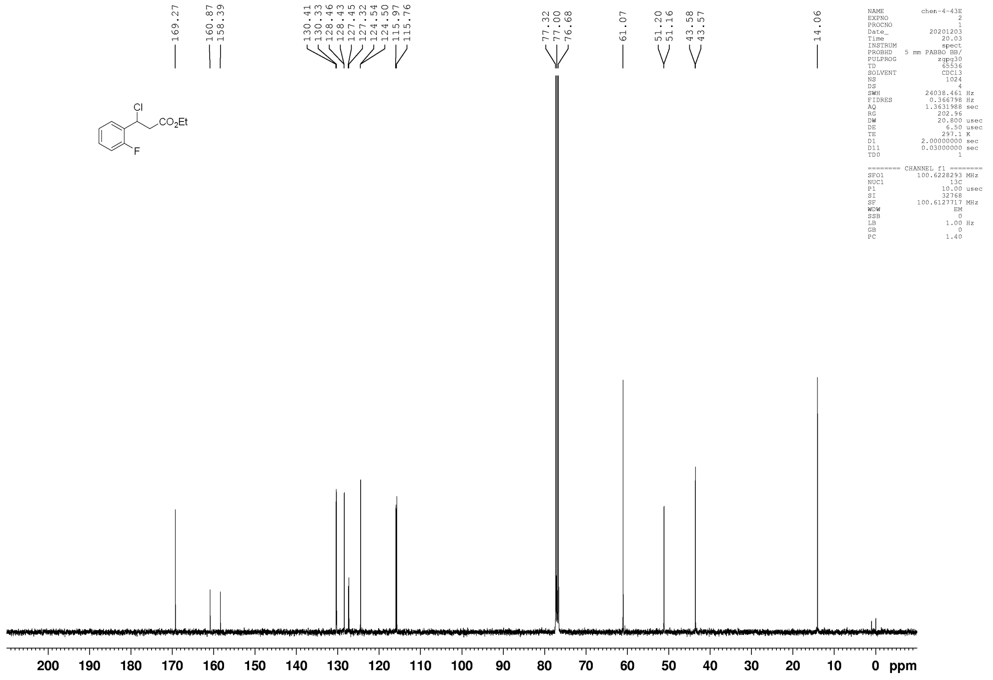

Supplementary Figure 48. ^{13}C NMR spectrum for compound **3o**

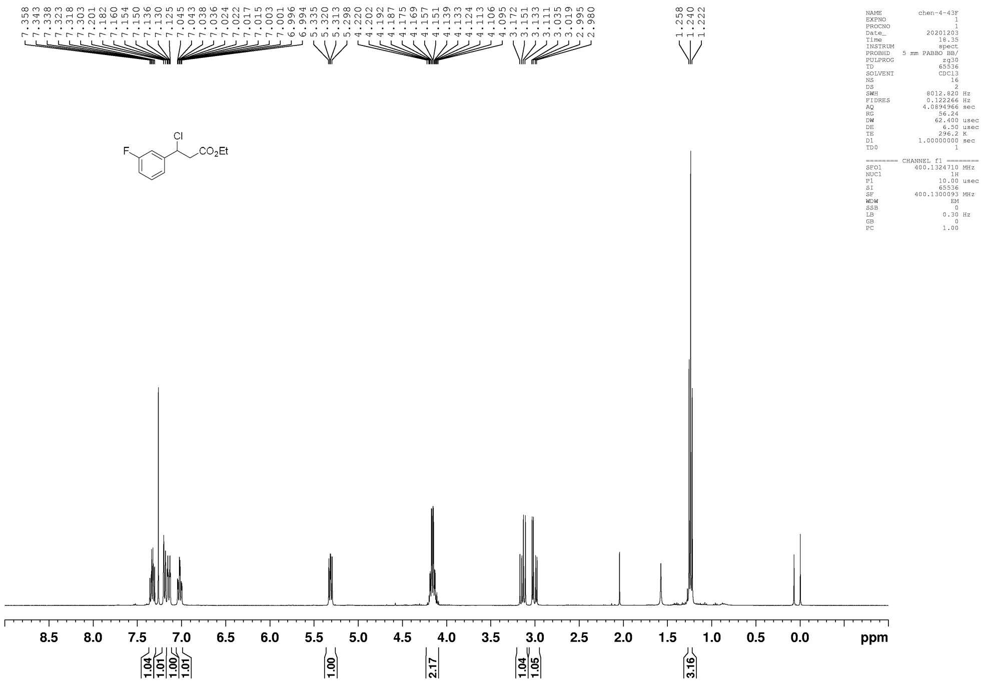

Supplementary Figure 49. ^{1}H NMR spectrum for compound **3p**

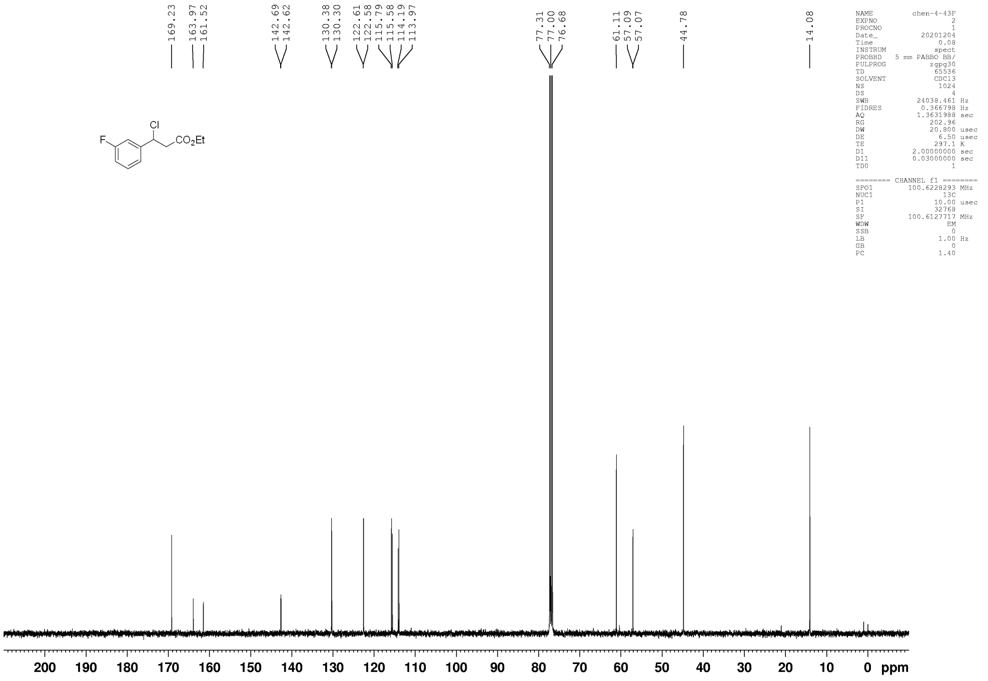

Supplementary Figure 50. ^{13}C NMR spectrum for compound **3p**

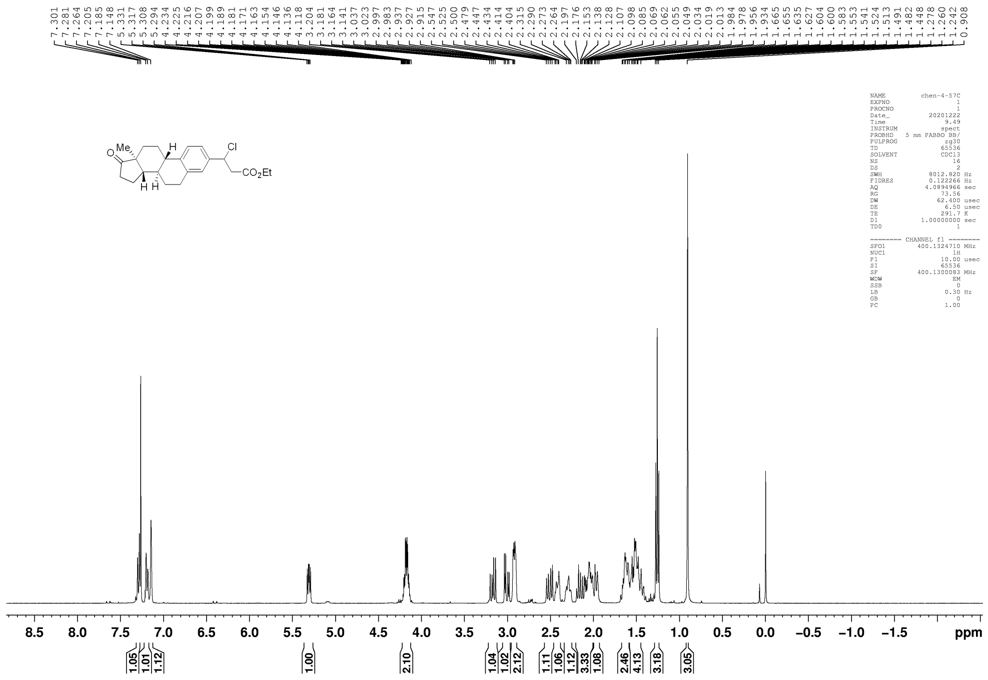

Supplementary Figure 51. ^{1}H NMR spectrum for compound **3q**

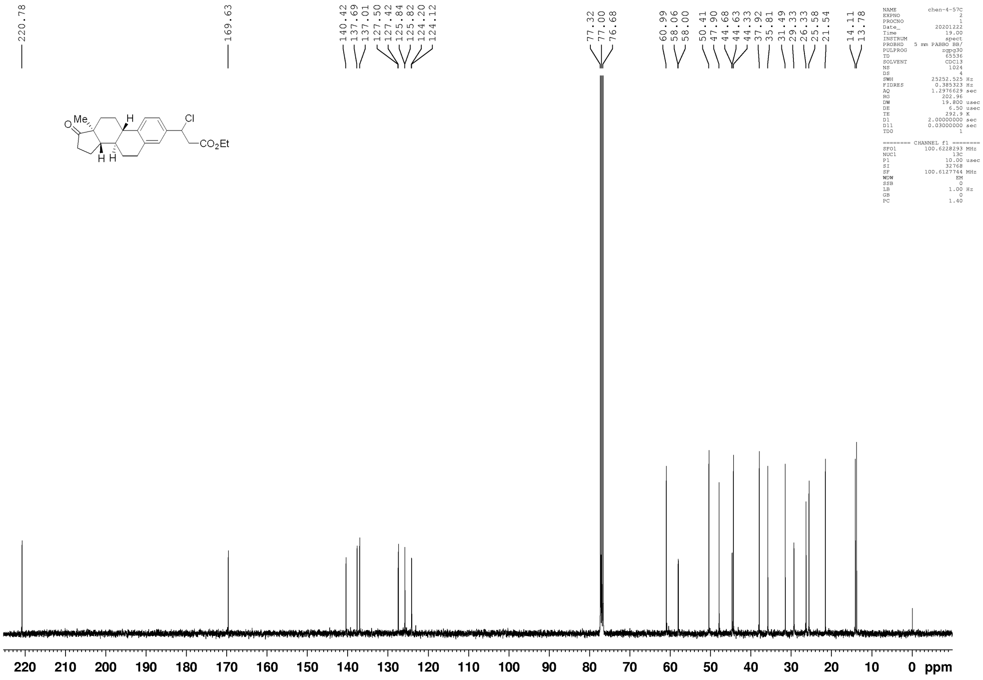

Supplementary Figure 52. ^{13}C NMR spectrum for compound **3q**

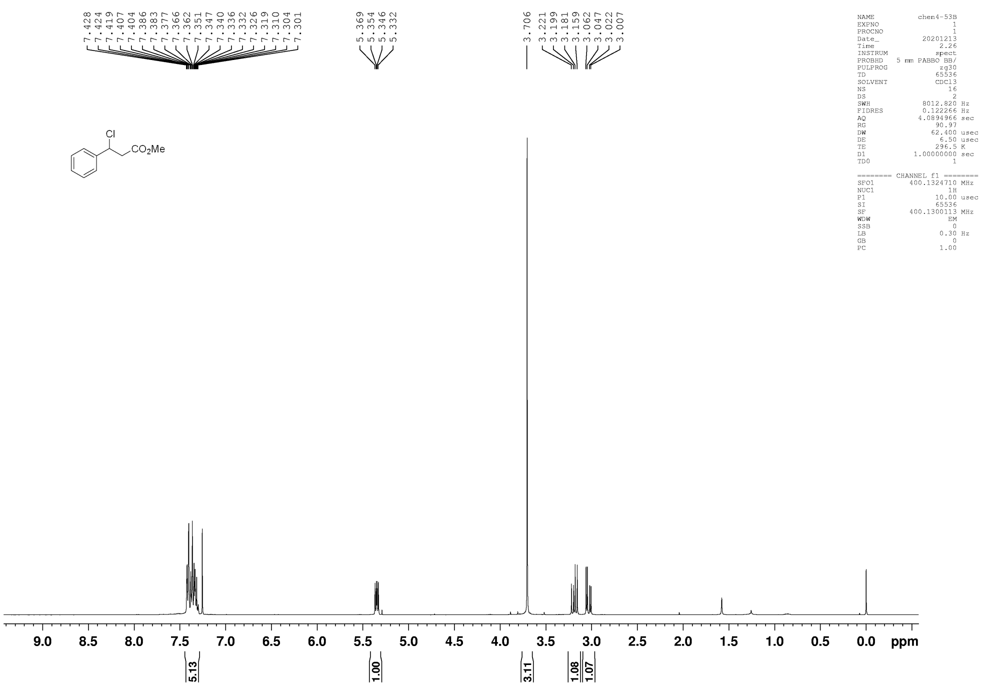

Supplementary Figure 53. ^{1}H NMR spectrum for compound **3r**

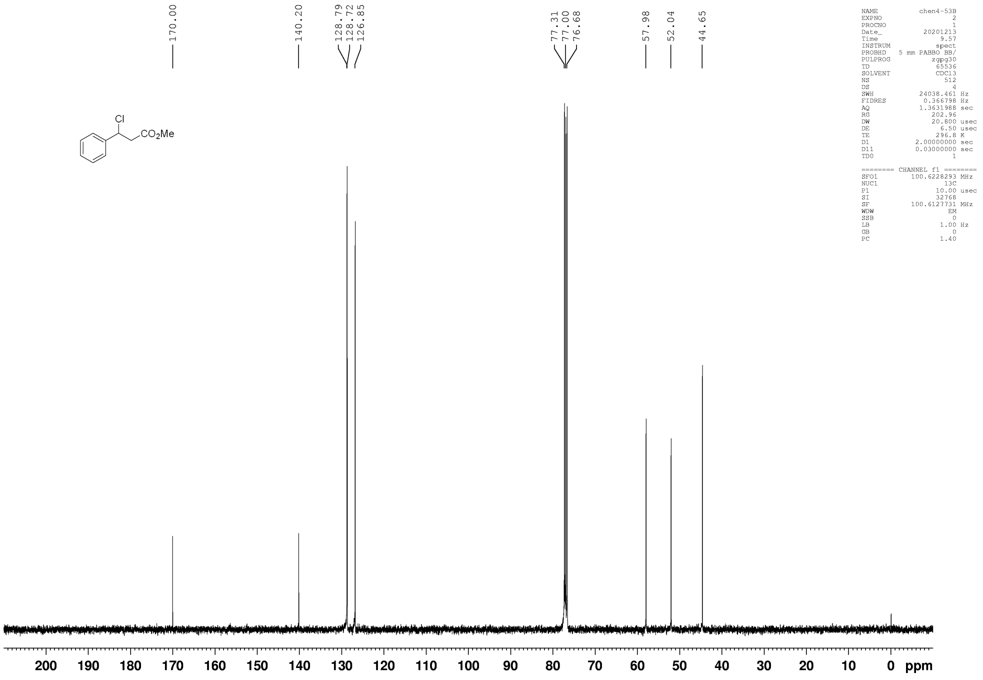

Supplementary Figure 54. ^{13}C NMR spectrum for compound **3r**

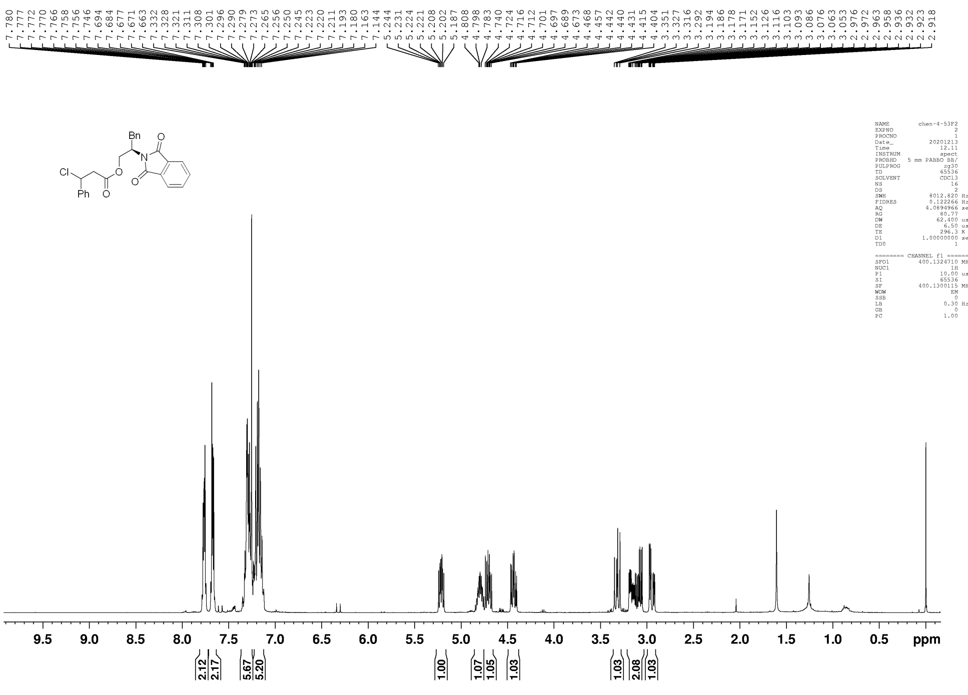

Supplementary Figure 55. ^{1}H NMR spectrum for compound **3s**

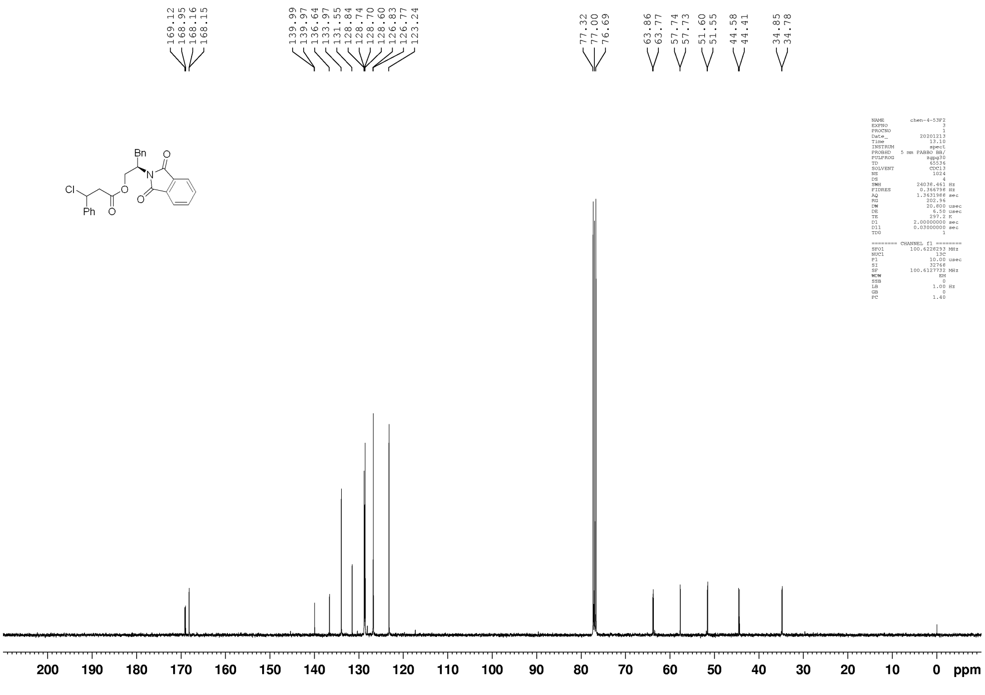

Supplementary Figure 56. ^{13}C NMR spectrum for compound **3s**

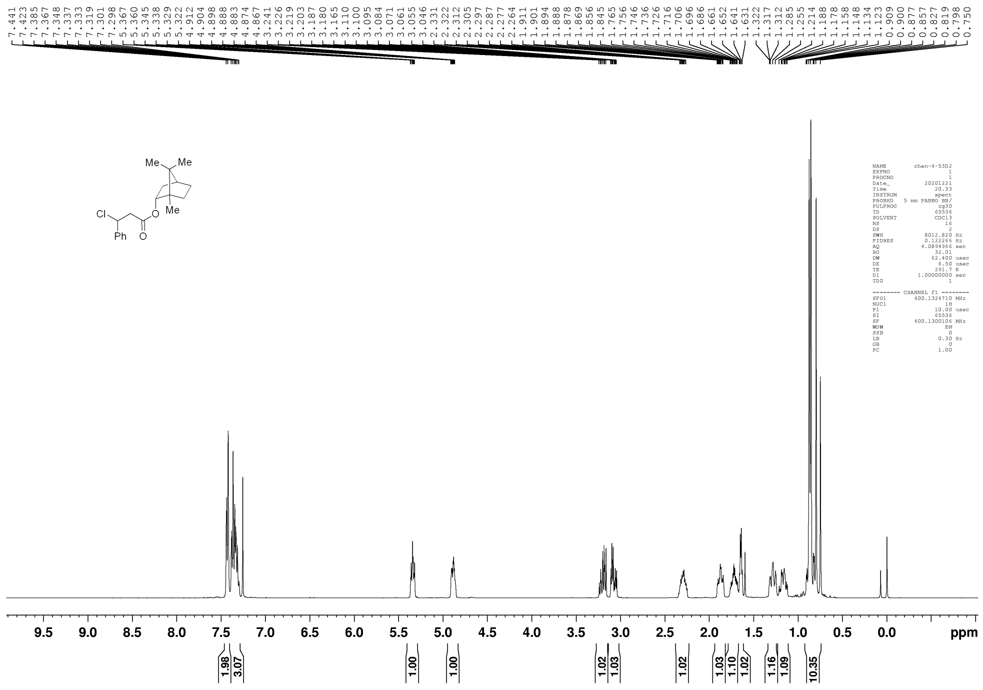

Supplementary Figure 57. ^{1}H NMR spectrum for compound **3t**

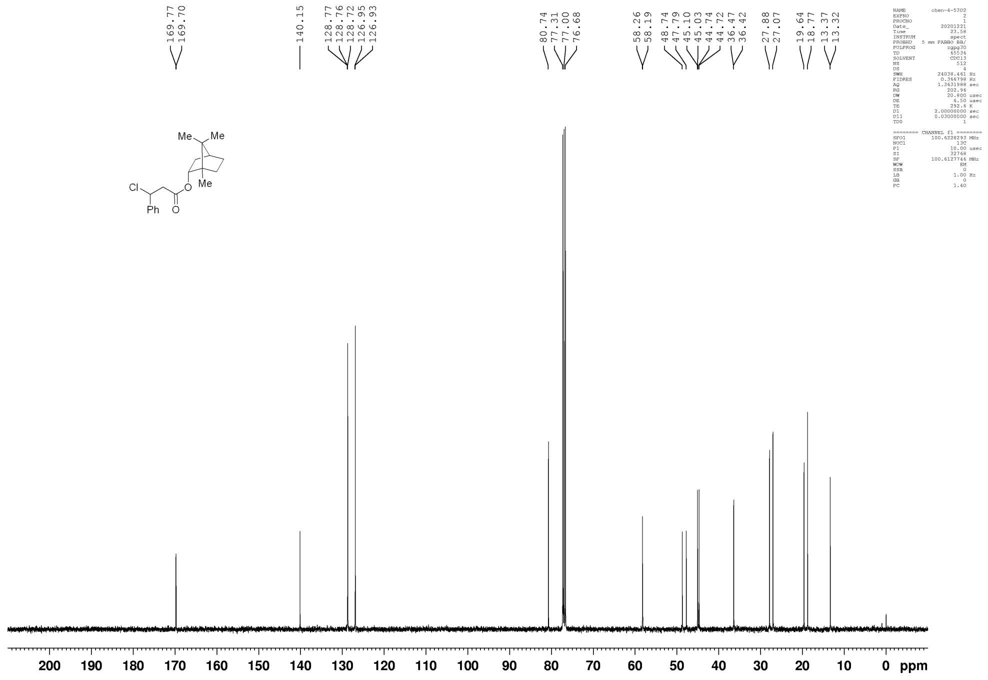

Supplementary Figure 58. ^{13}C NMR spectrum for compound **3t**

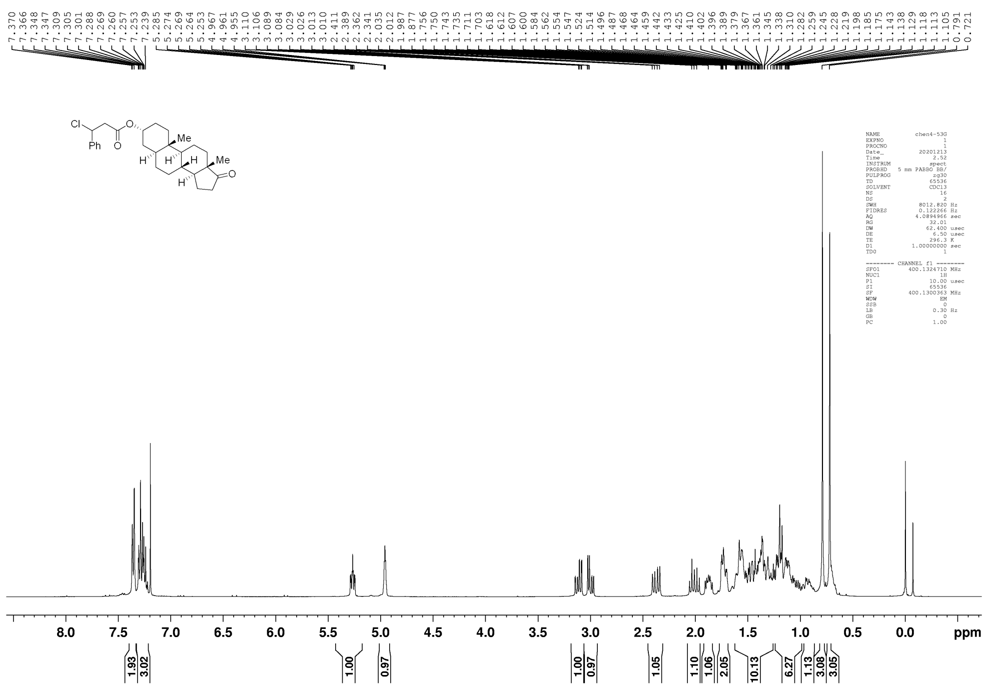

Supplementary Figure 59. ^{1}H NMR spectrum for compound **3u**

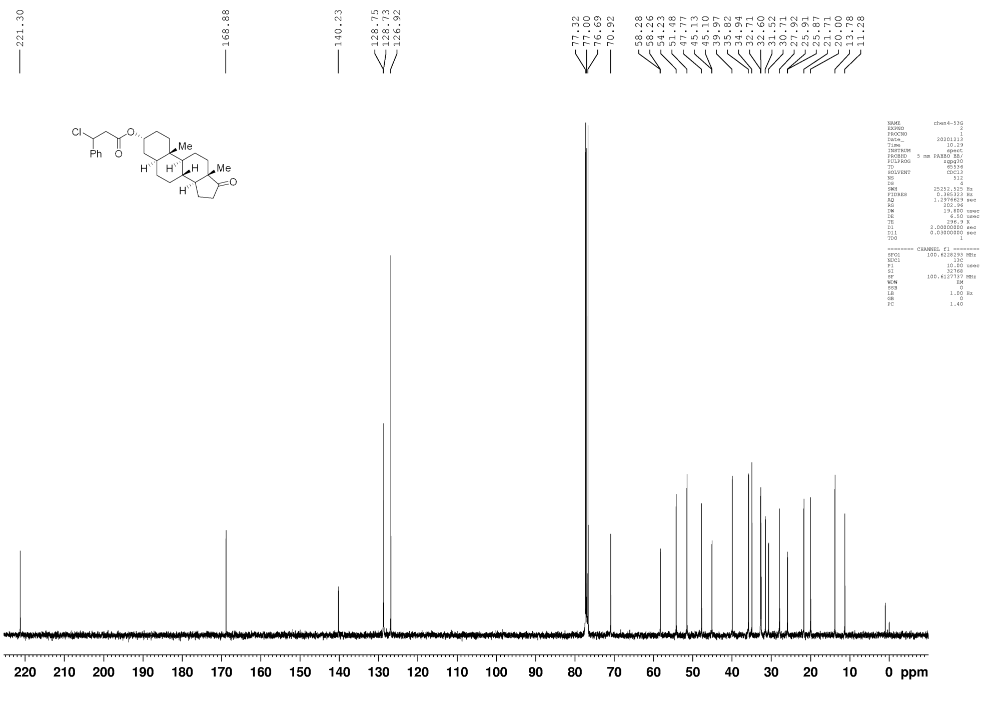

Supplementary Figure 60. ^{13}C NMR spectrum for compound **3u**

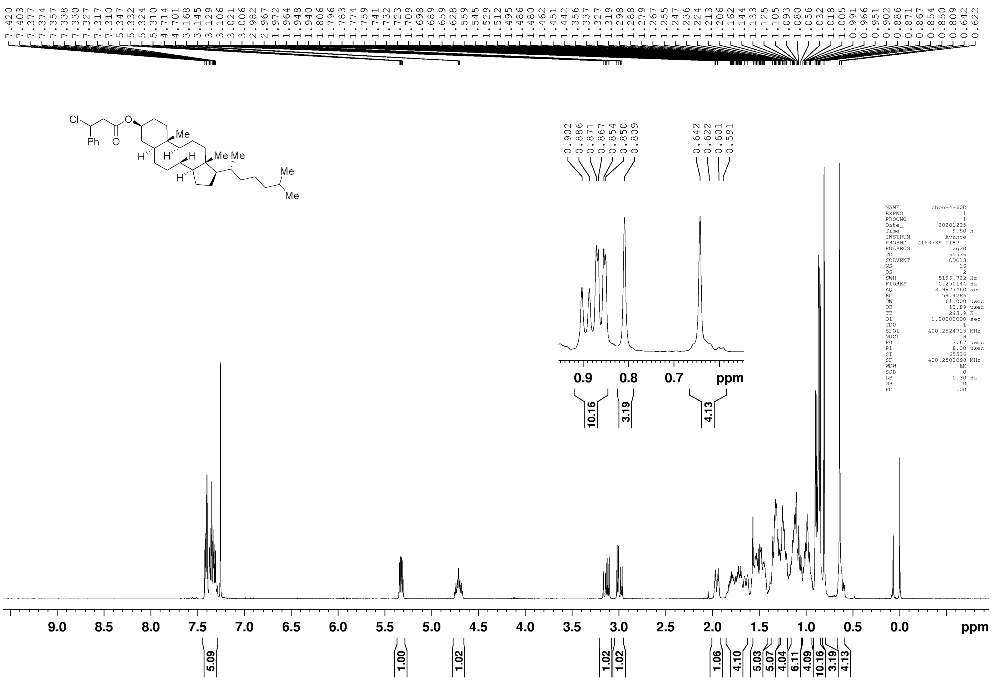

Supplementary Figure 61. ^{1}H NMR spectrum for compound **3v**

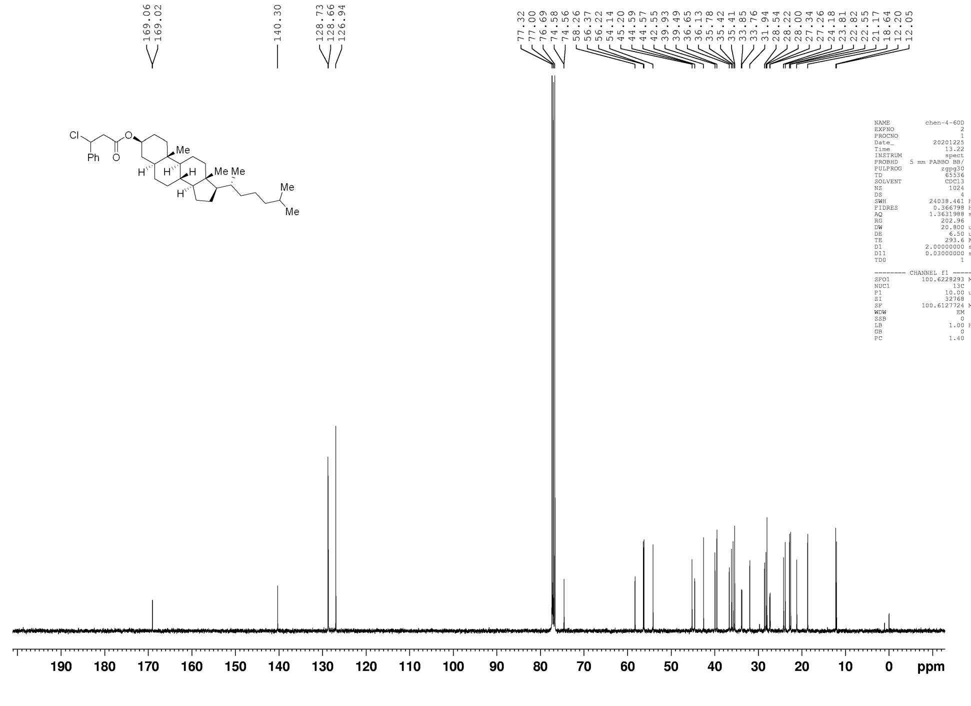

Supplementary Figure 62. ^{13}C NMR spectrum for compound **3v**

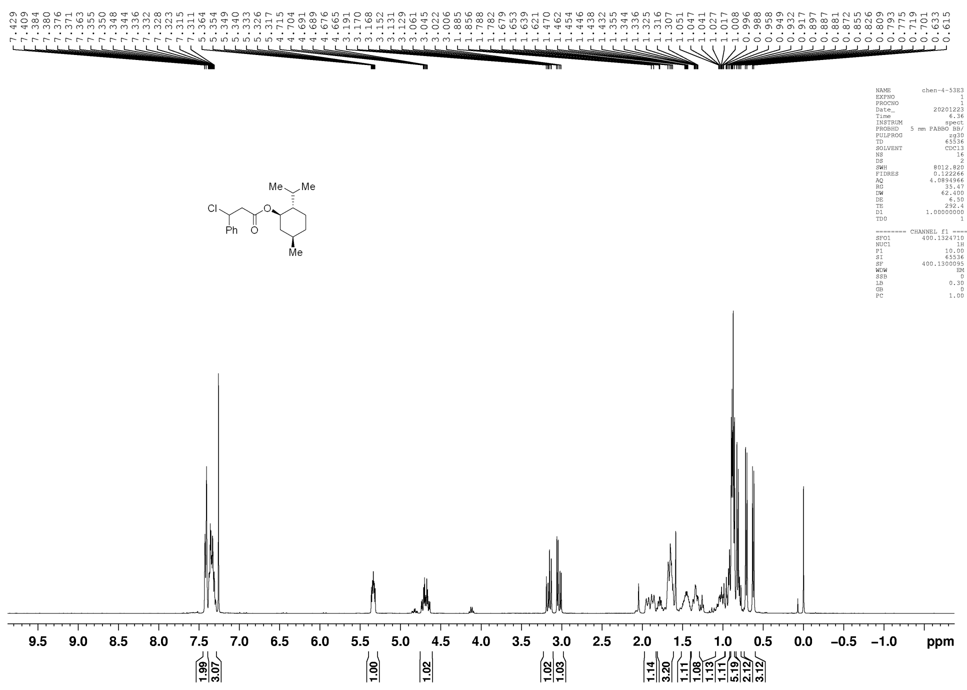

Supplementary Figure 63. ^{1}H NMR spectrum for compound **3w**

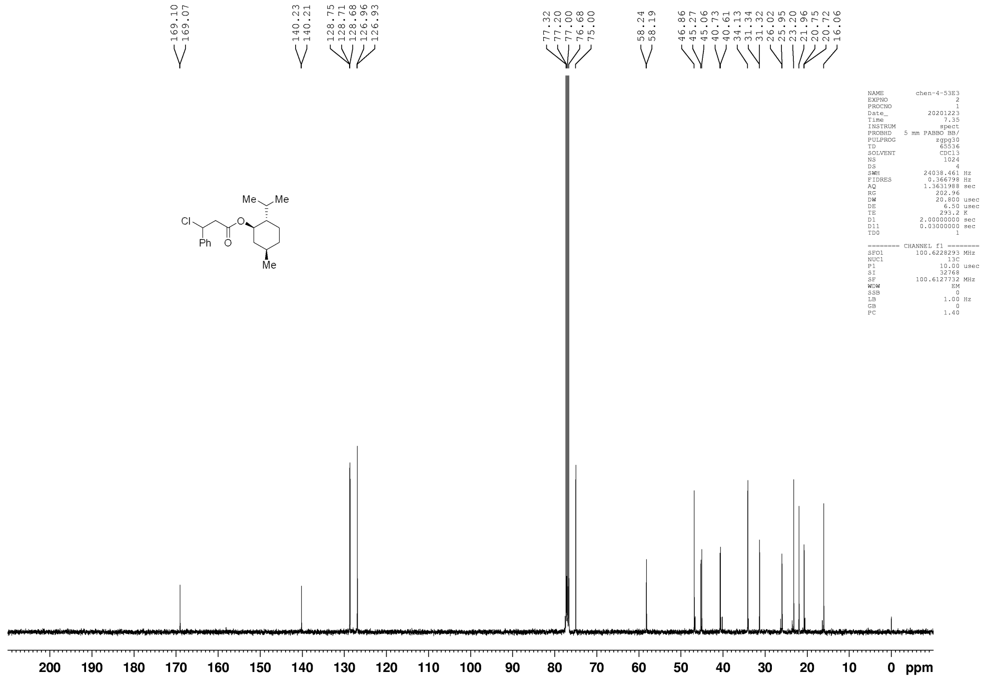

Supplementary Figure 64. ^{13}C NMR spectrum for compound **3w**

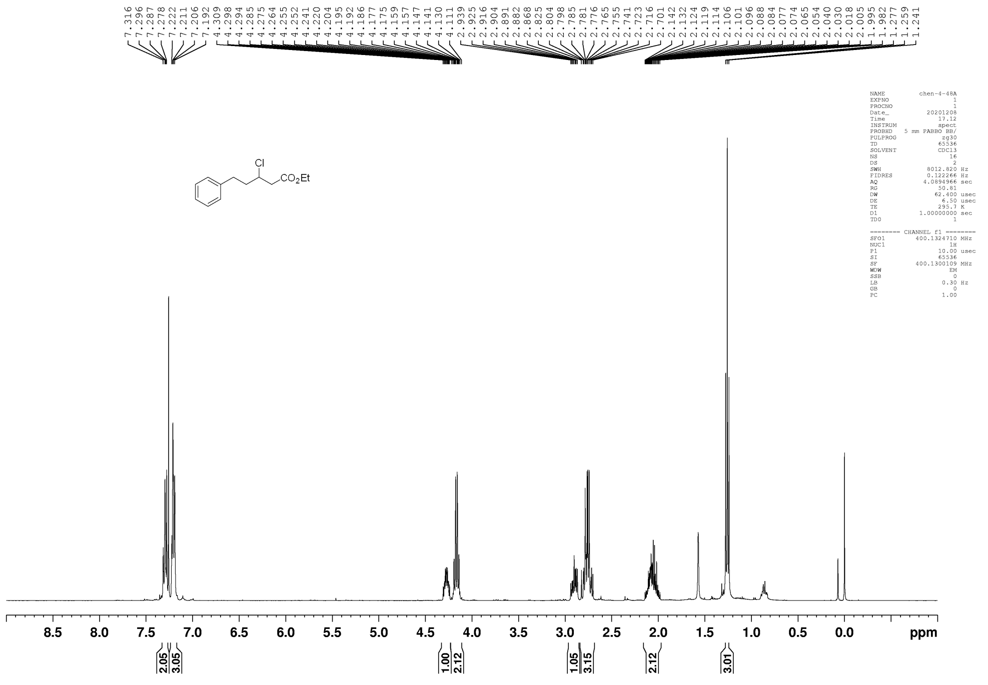

Supplementary Figure 65. ^{1}H NMR spectrum for compound **5a**

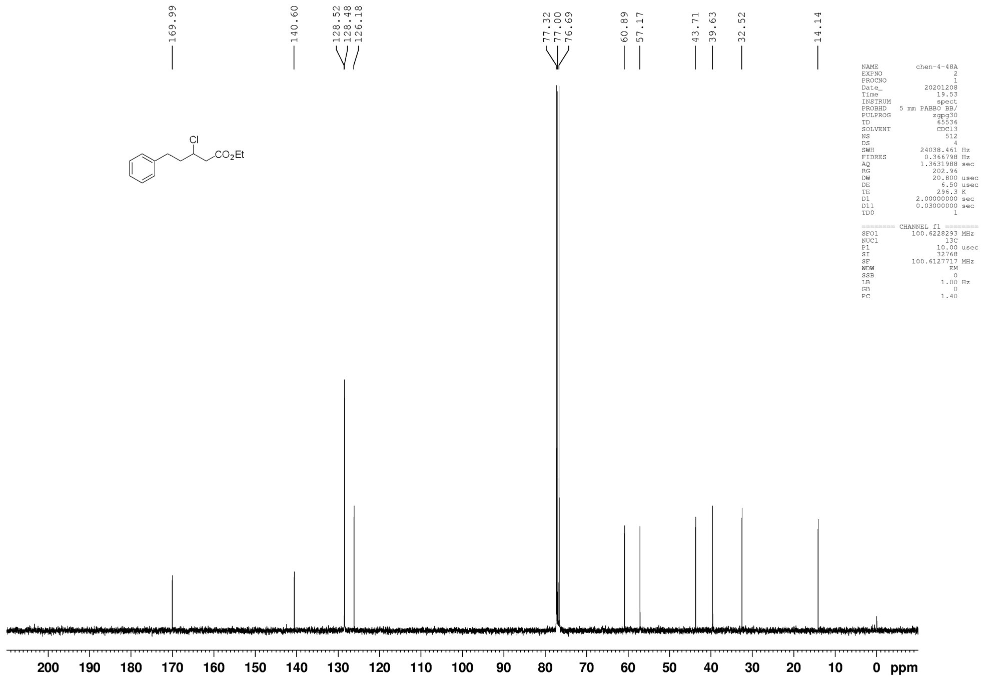

Supplementary Figure 66. ^{13}C NMR spectrum for compound **5a**

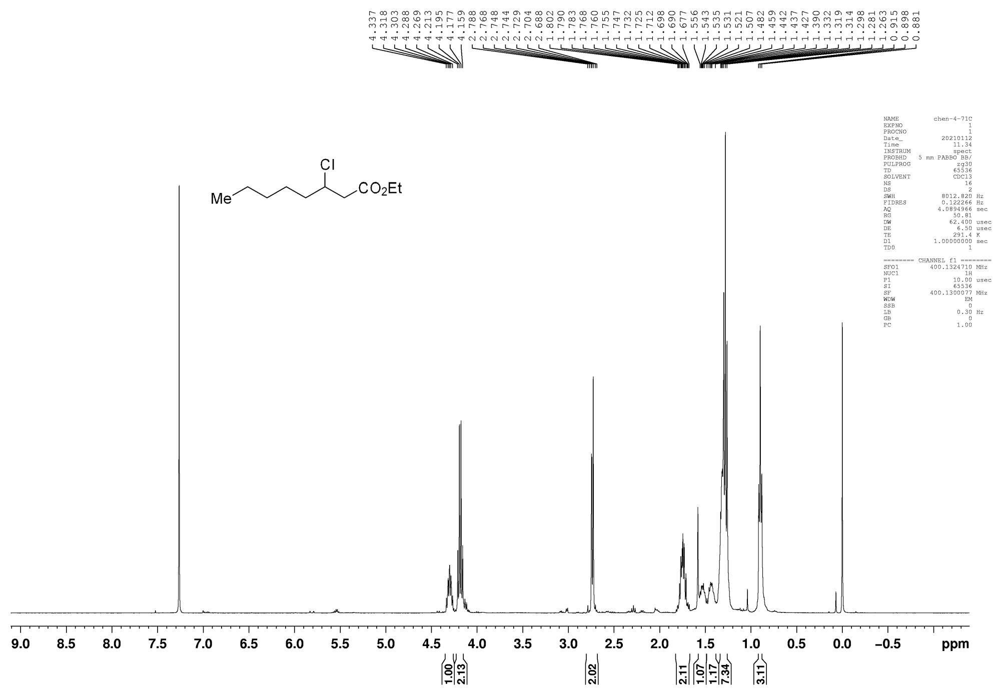

Supplementary Figure 67. ^{1}H NMR spectrum for compound **5b**

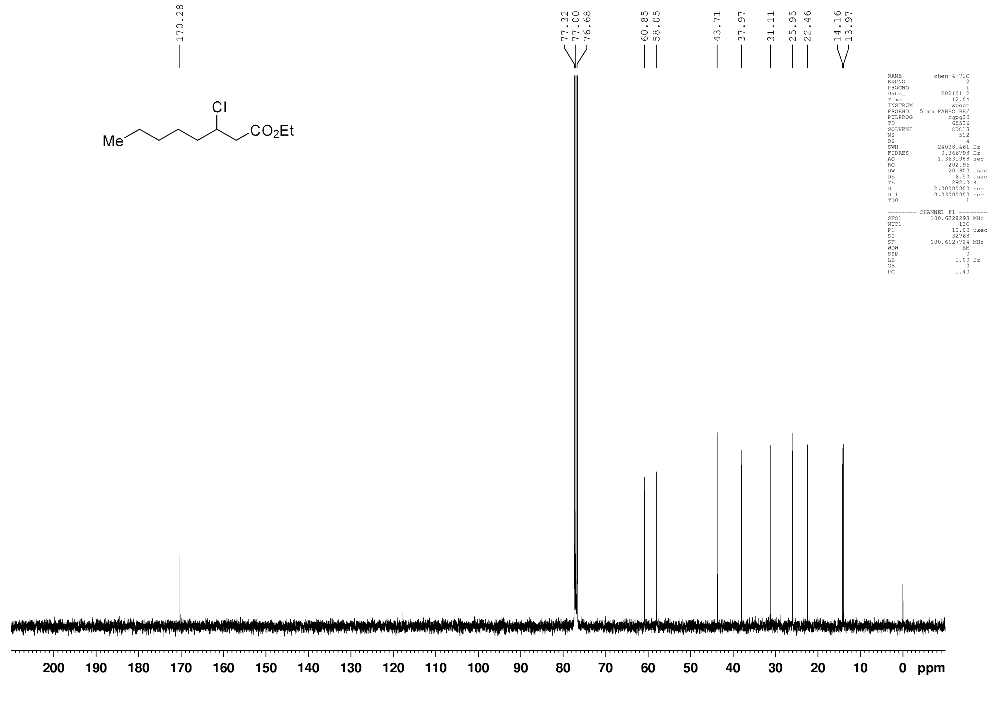

Supplementary Figure 68. ^{13}C NMR spectrum for compound **5b**

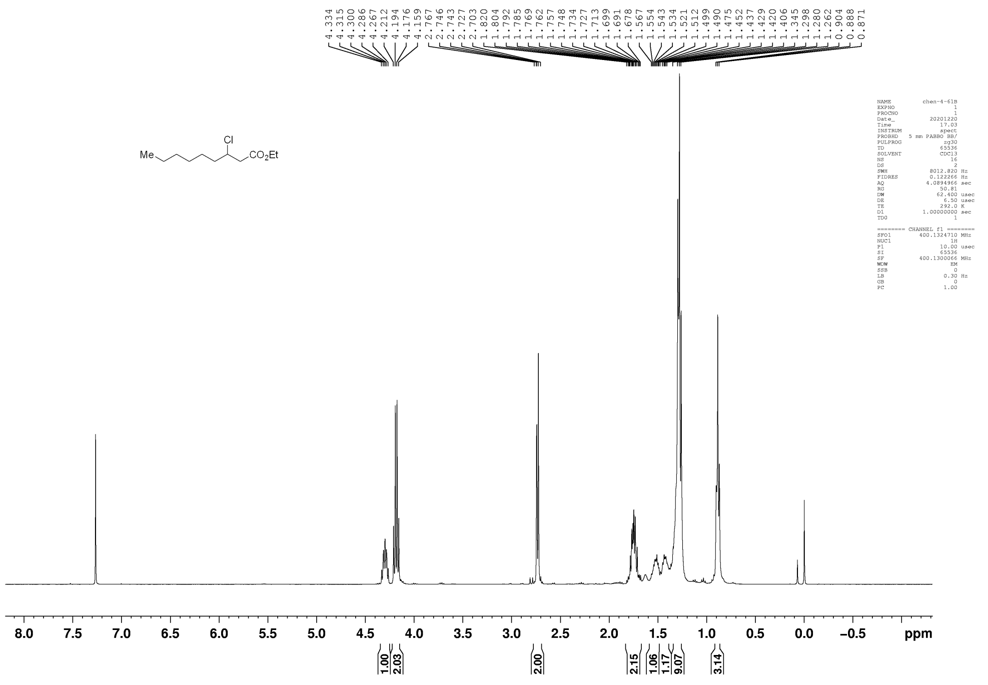

Supplementary Figure 69. ^{1}H NMR spectrum for compound **5c**

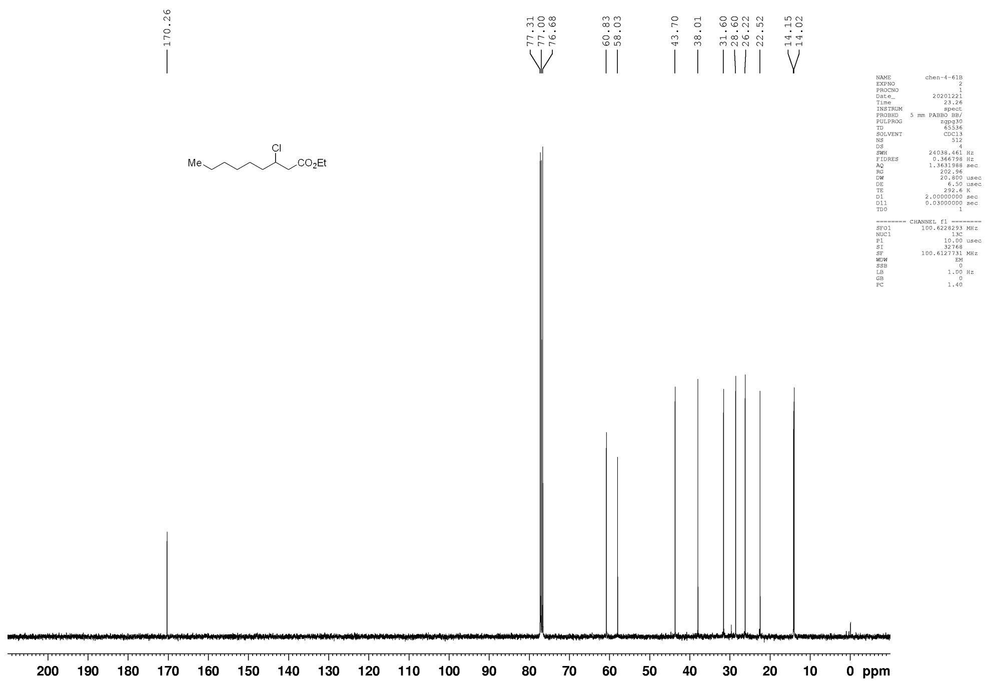

Supplementary Figure 70. ^{13}C NMR spectrum for compound **5c**

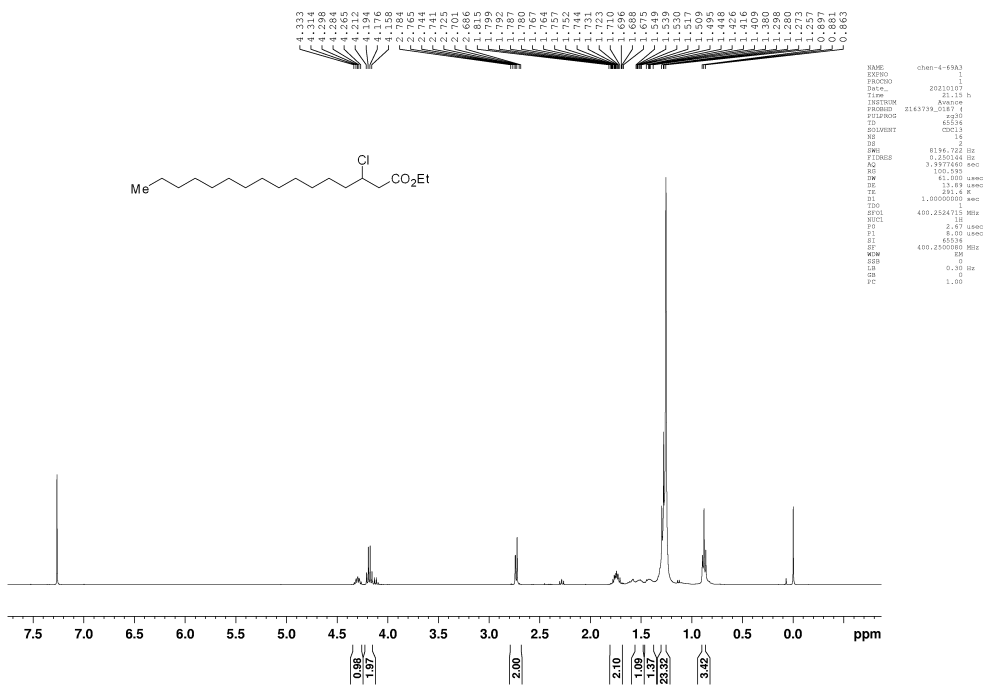

Supplementary Figure 71. ^{1}H NMR spectrum for compound **5d**

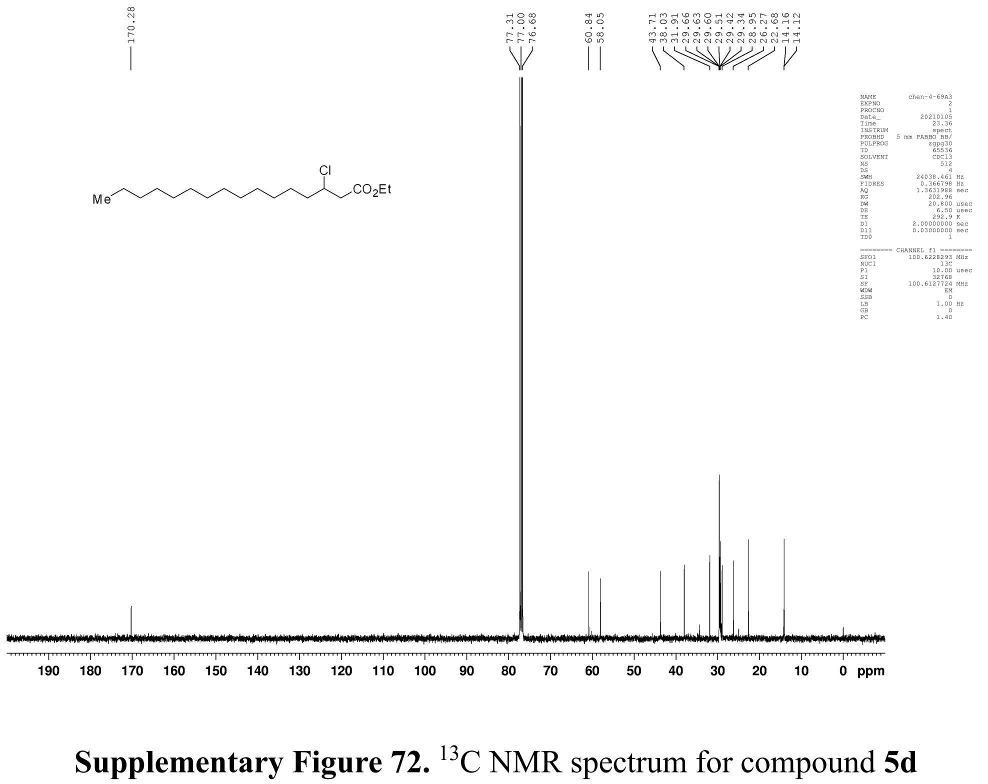

Supplementary Figure 72. ^{13}C NMR spectrum for compound **5d**

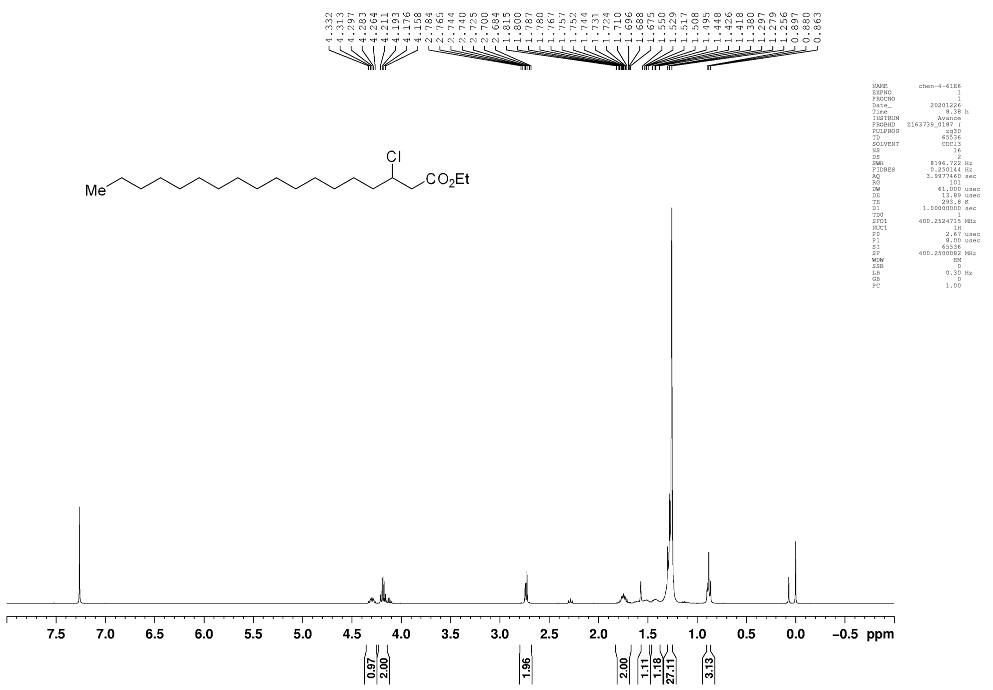

Supplementary Figure 73. ^{1}H NMR spectrum for compound **5e**

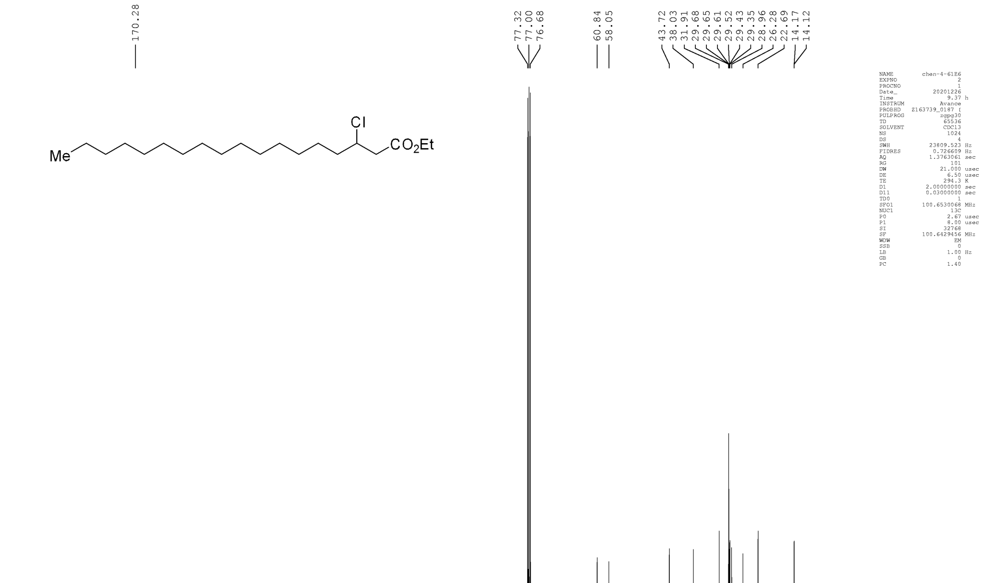

Supplementary Figure 74. ^{13}C NMR spectrum for compound **5e**

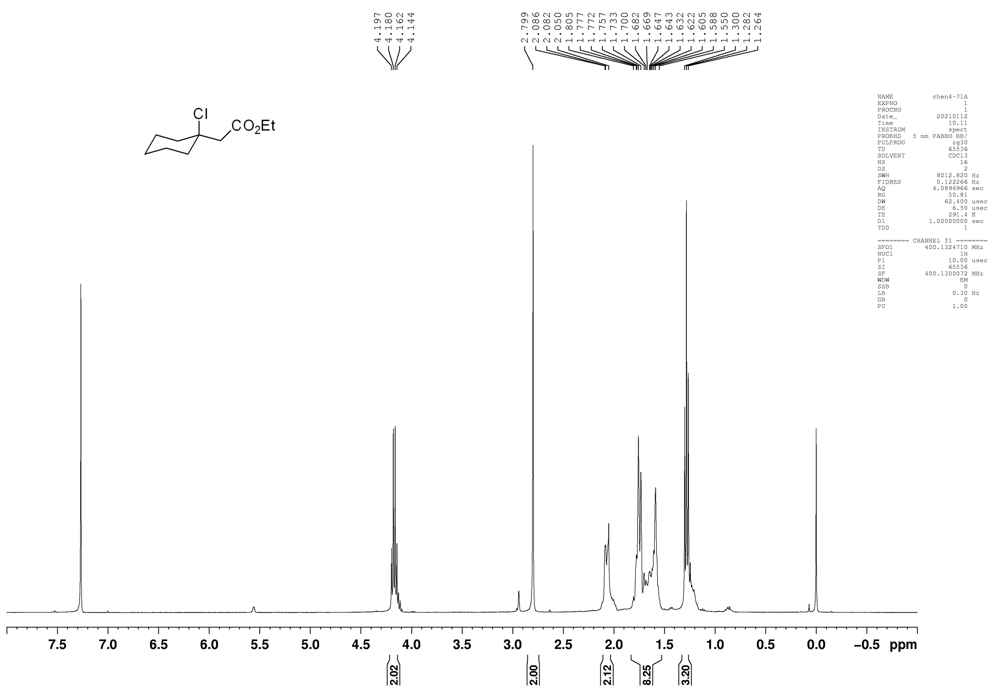

Supplementary Figure 75. ^{1}H NMR spectrum for compound **5f**

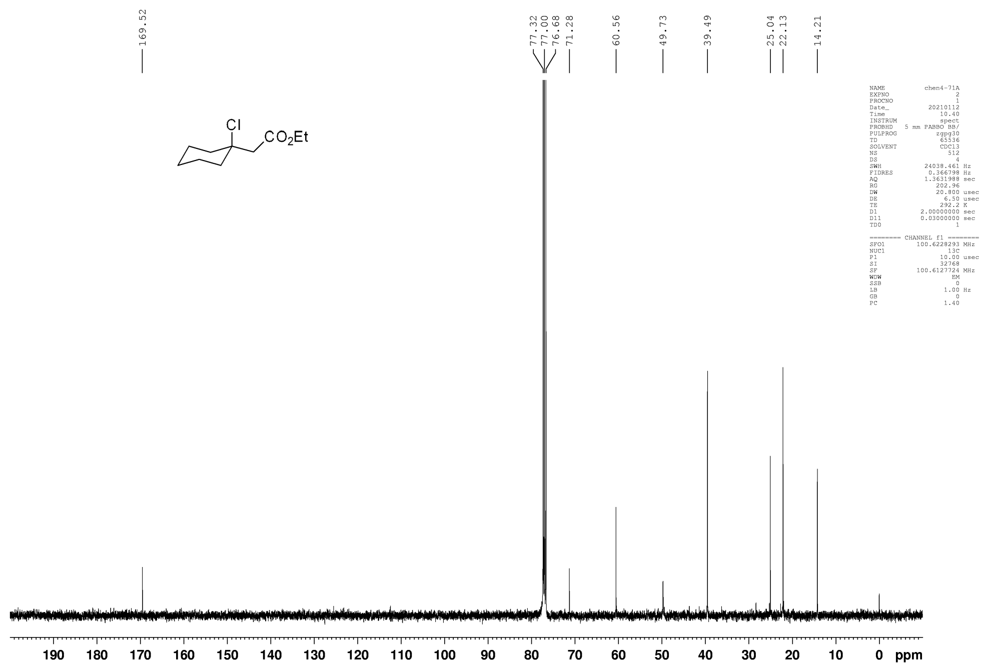

Supplementary Figure 76. ^{13}C NMR spectrum for compound **5f**

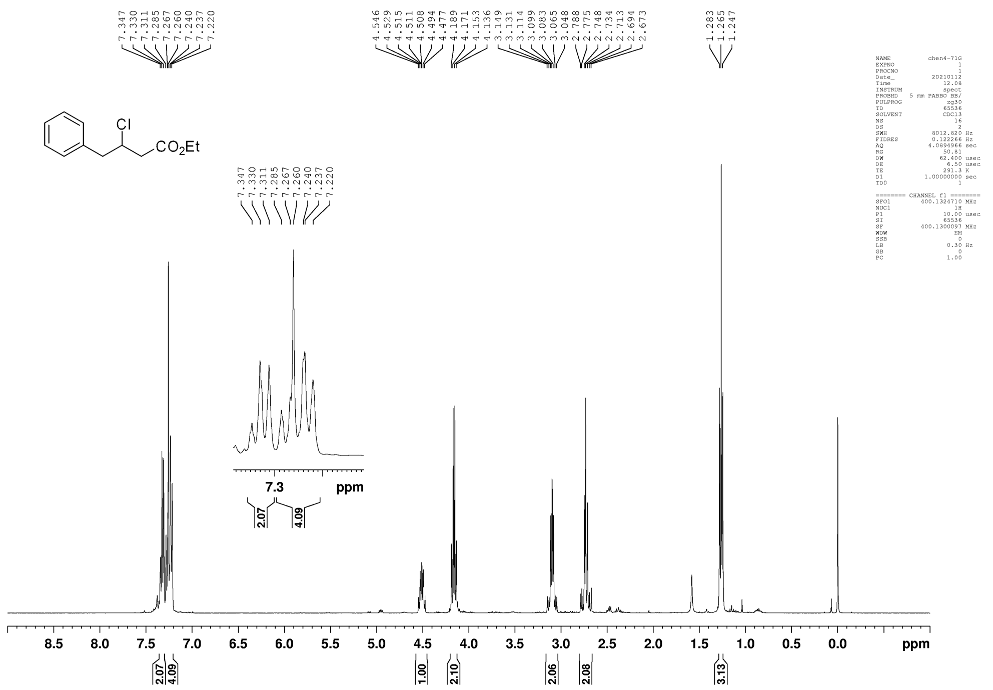

Supplementary Figure 77. ^{1}H NMR spectrum for compound **5g**

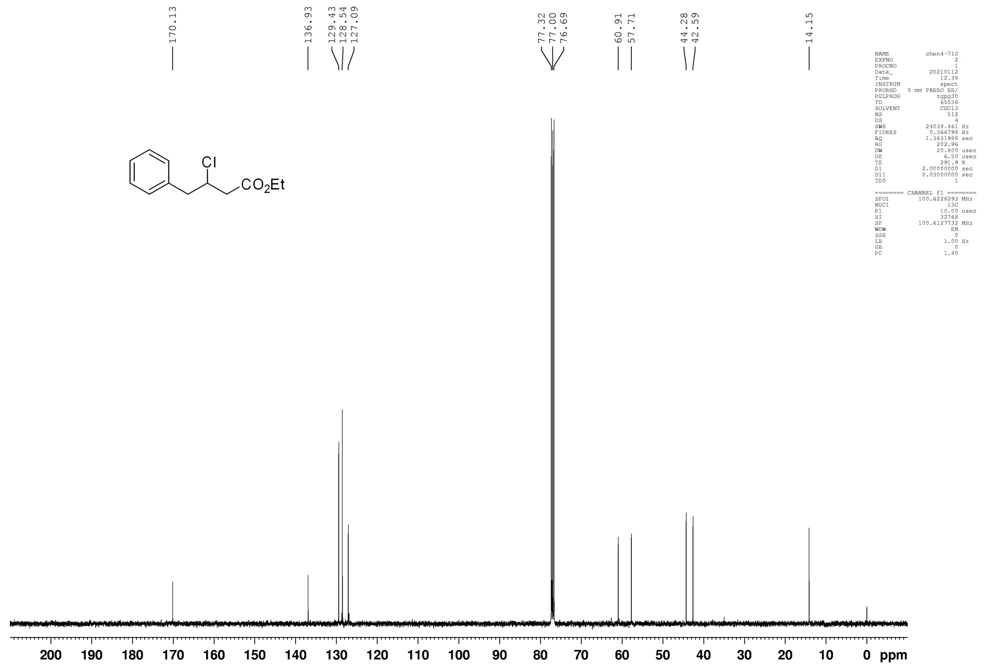

Supplementary Figure 78. ^{13}C NMR spectrum for compound **5g**

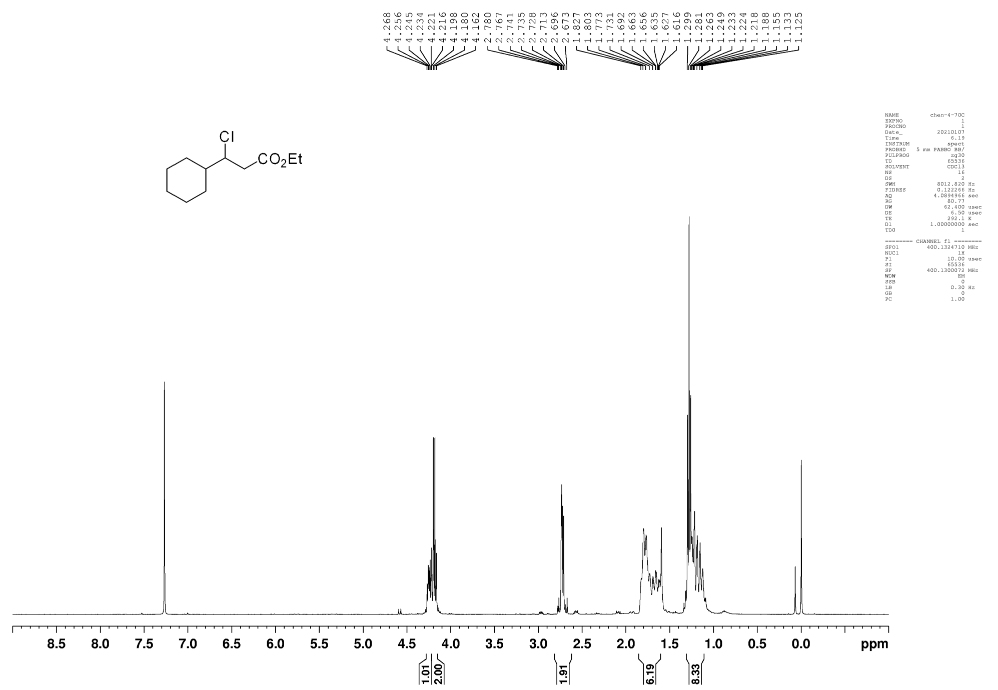

Supplementary Figure 79. ^{1}H NMR spectrum for compound **5h**

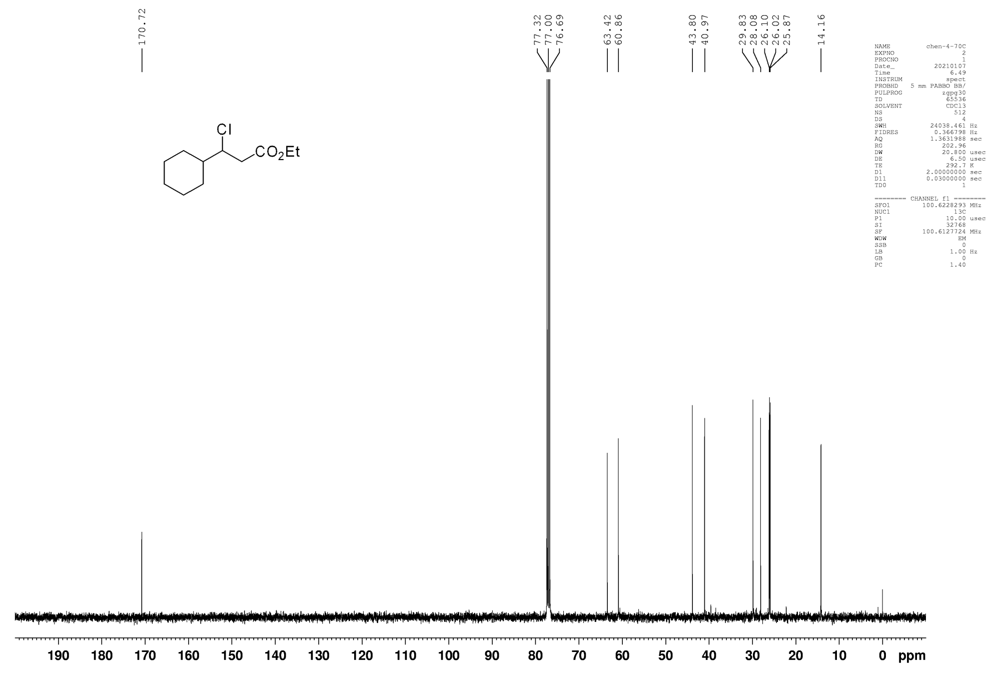

Supplementary Figure 80. ^{13}C NMR spectrum for compound **5h**

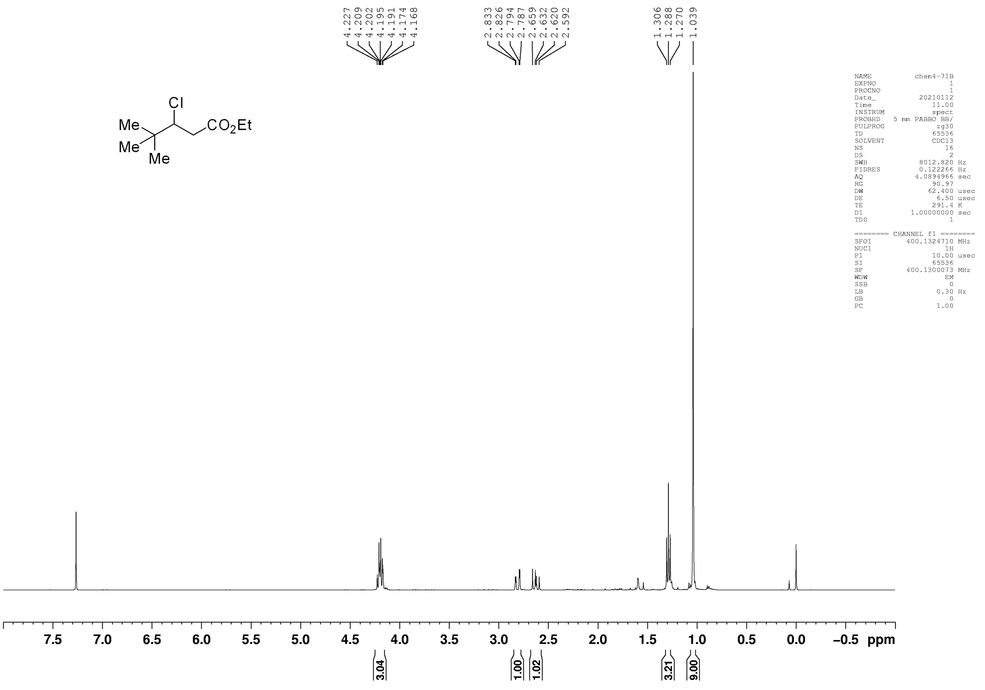

Supplementary Figure 81. ^{1}H NMR spectrum for compound **5i**

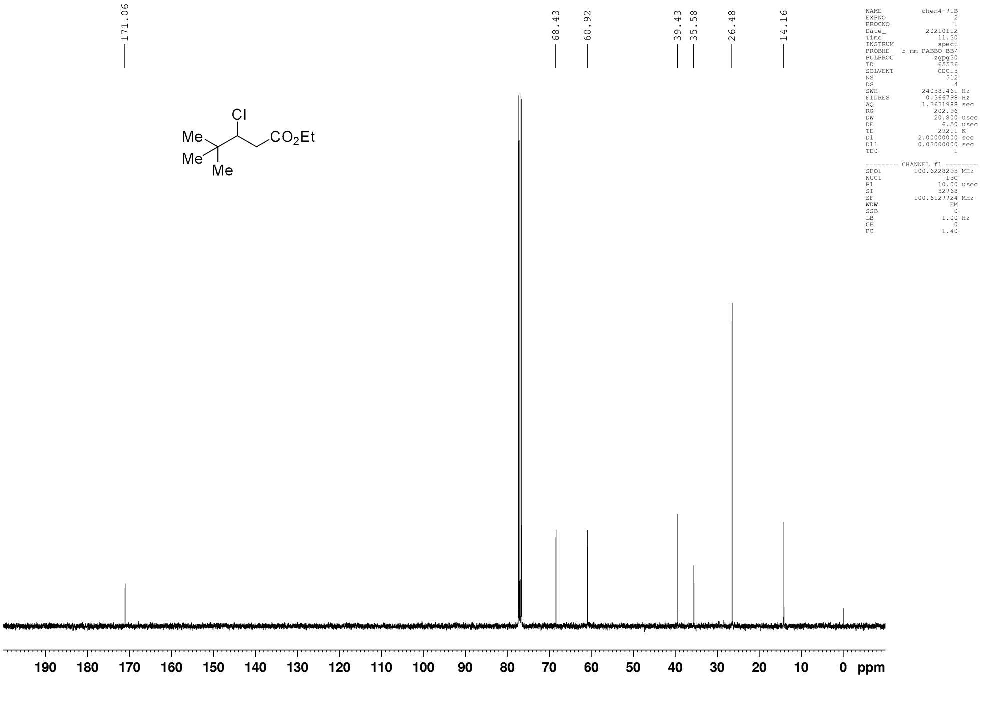

Supplementary Figure 82. ^{13}C NMR spectrum for compound **5i**

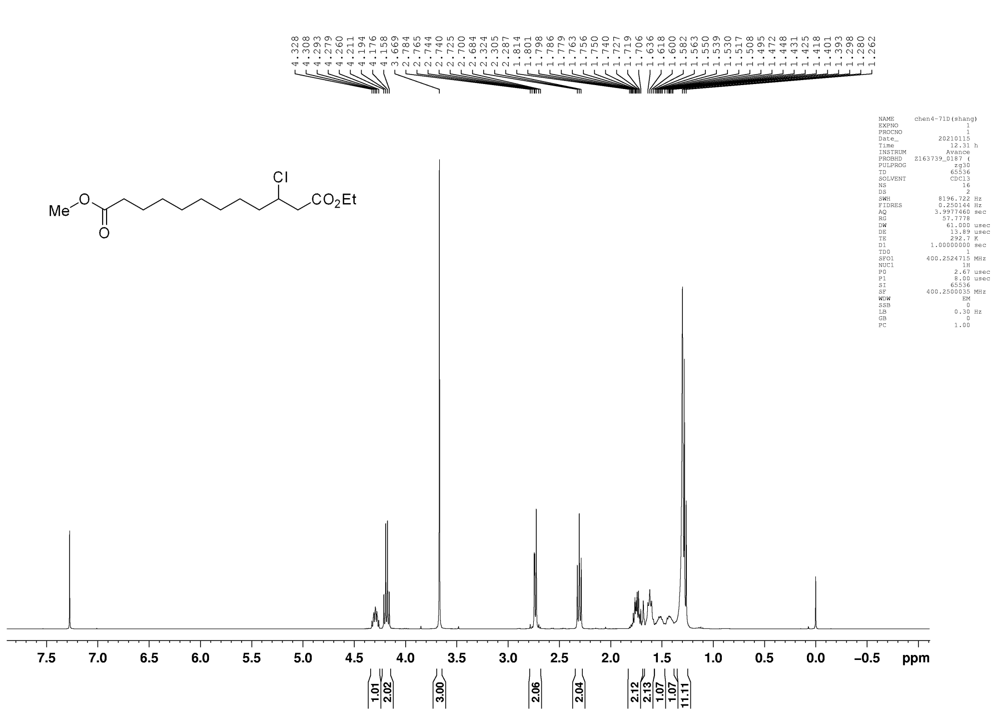

Supplementary Figure 83. ^{1}H NMR spectrum for compound **5j**

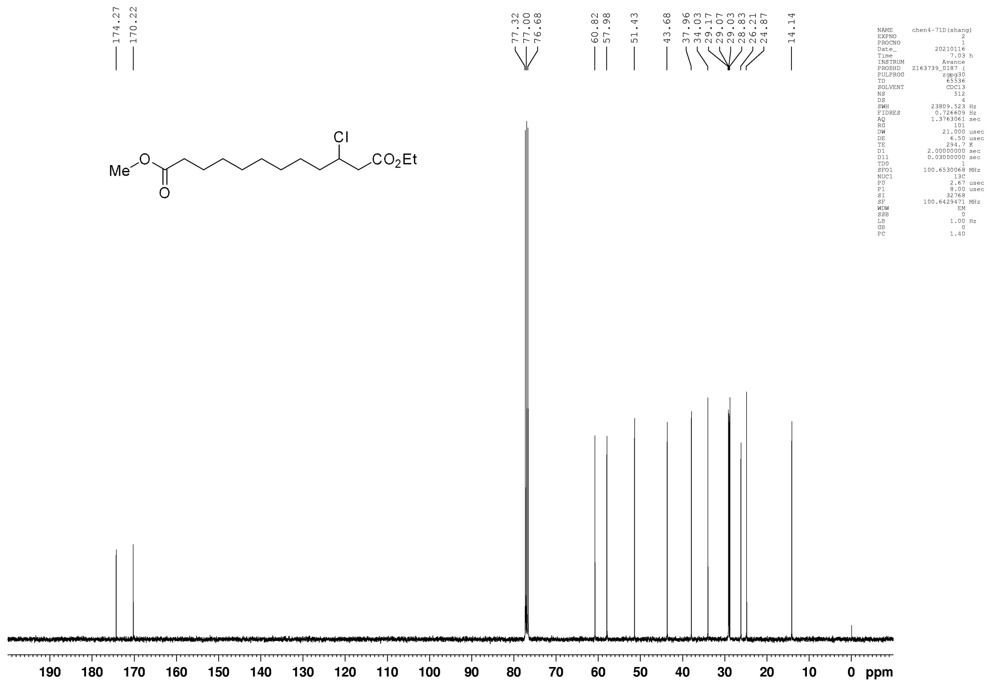

Supplementary Figure 84. ^{13}C NMR spectrum for compound **5j**

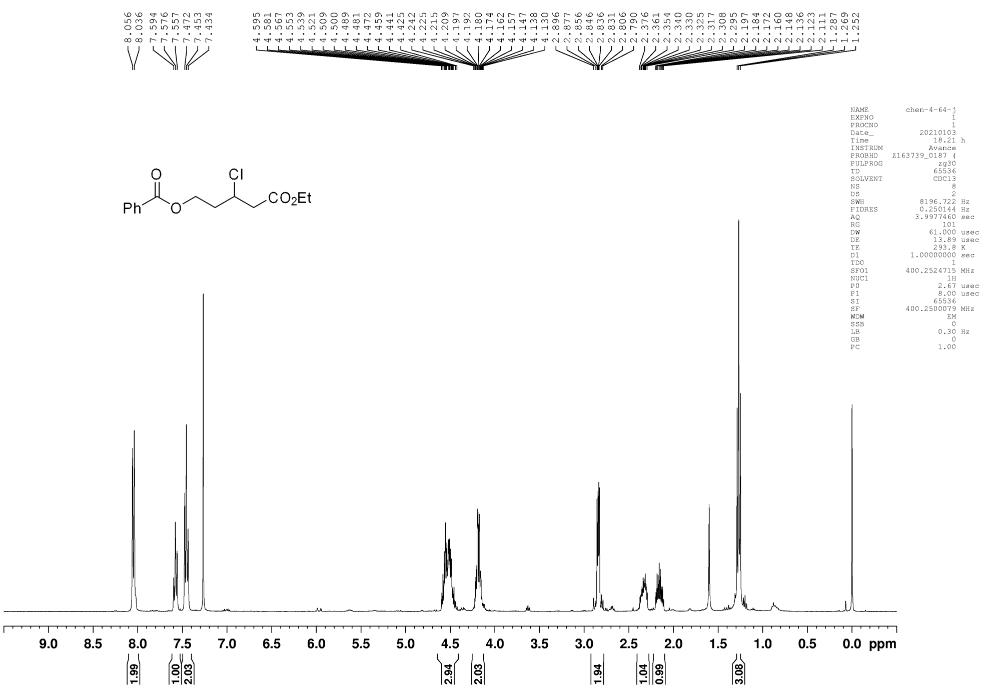

Supplementary Figure 85. ^{1}H NMR spectrum for compound **5k**

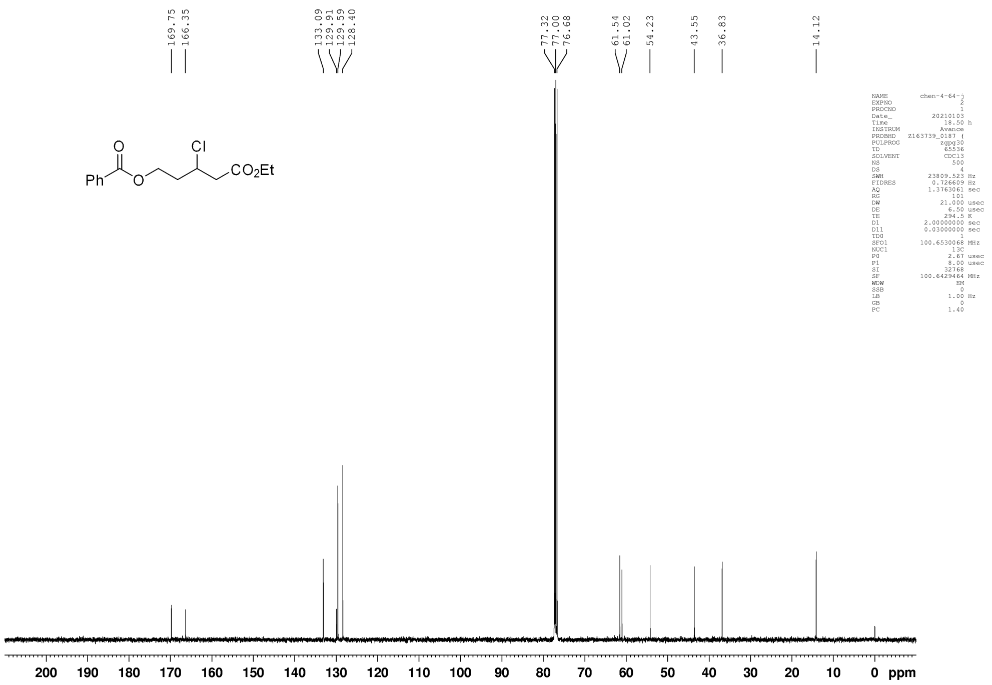

Supplementary Figure 86. ^{13}C NMR spectrum for compound **5k**

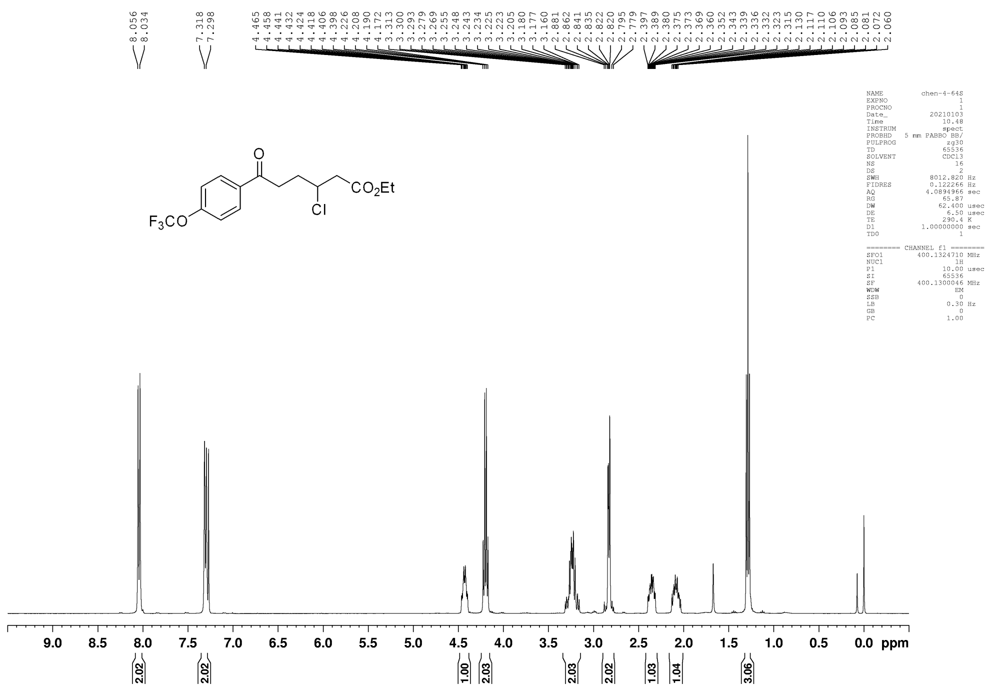

Supplementary Figure 87. ^{1}H NMR spectrum for compound **5l**

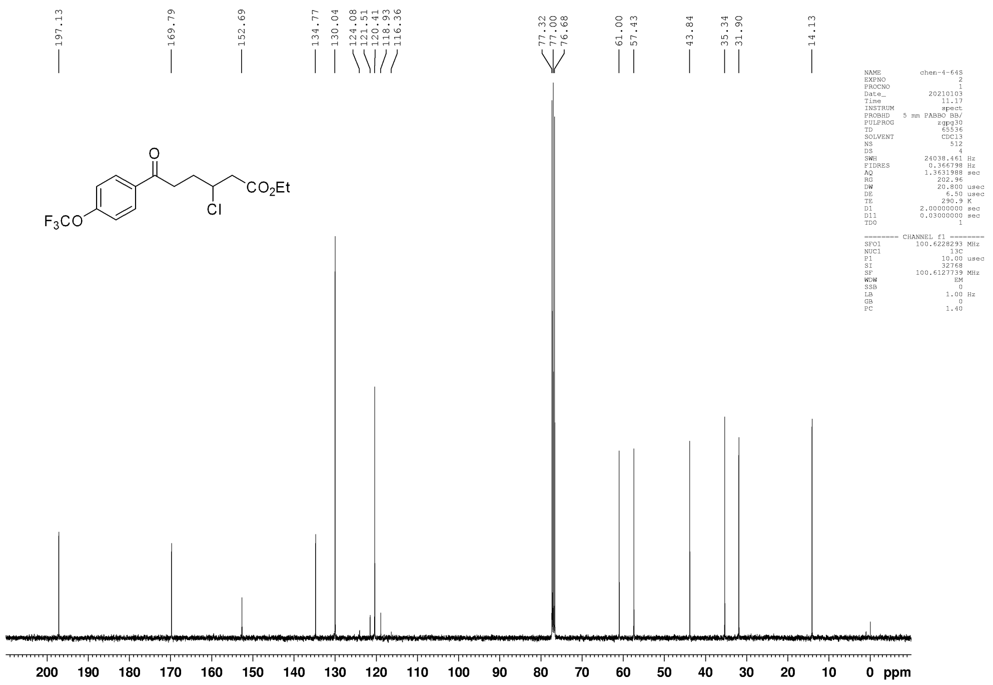

Supplementary Figure 88. ^{13}C NMR spectrum for compound **5l**

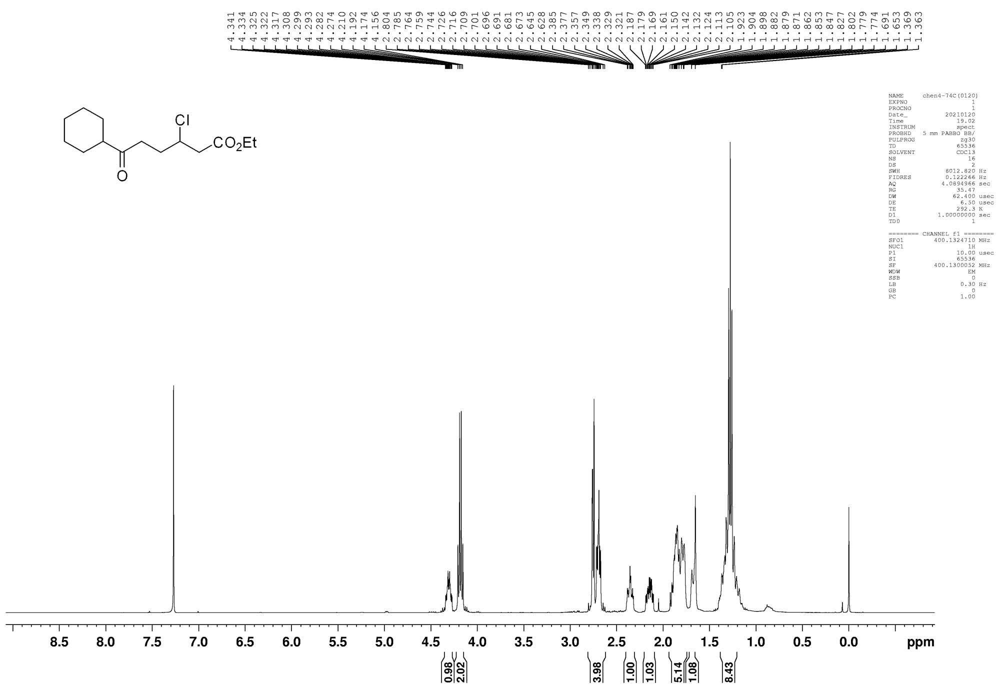

Supplementary Figure 89. ^{1}H NMR spectrum for compound **5m**

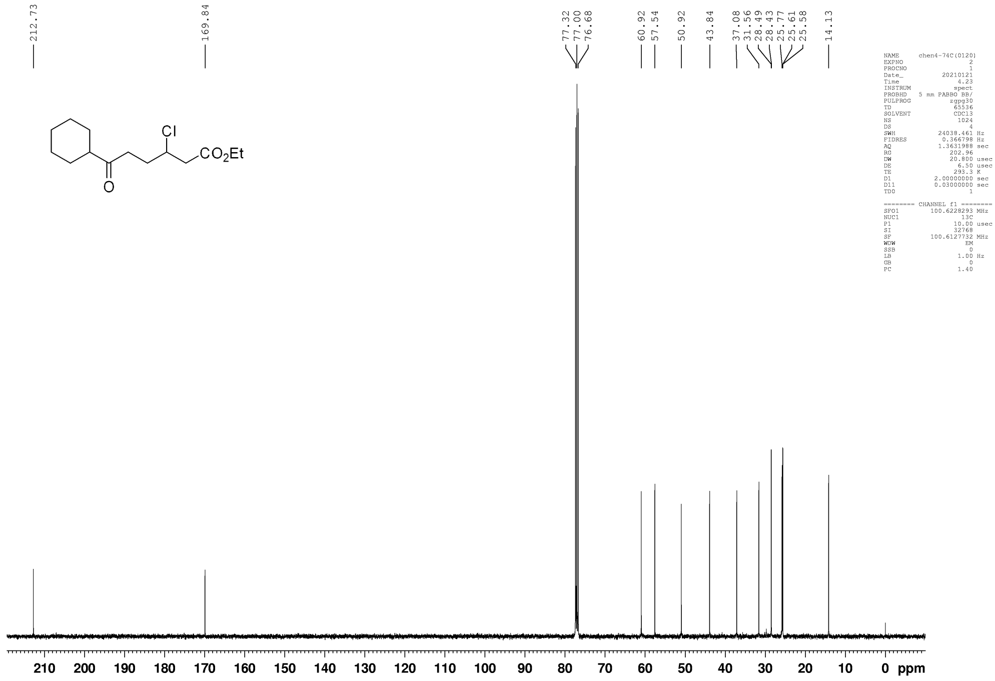

Supplementary Figure 90. ^{13}C NMR spectrum for compound **5m**

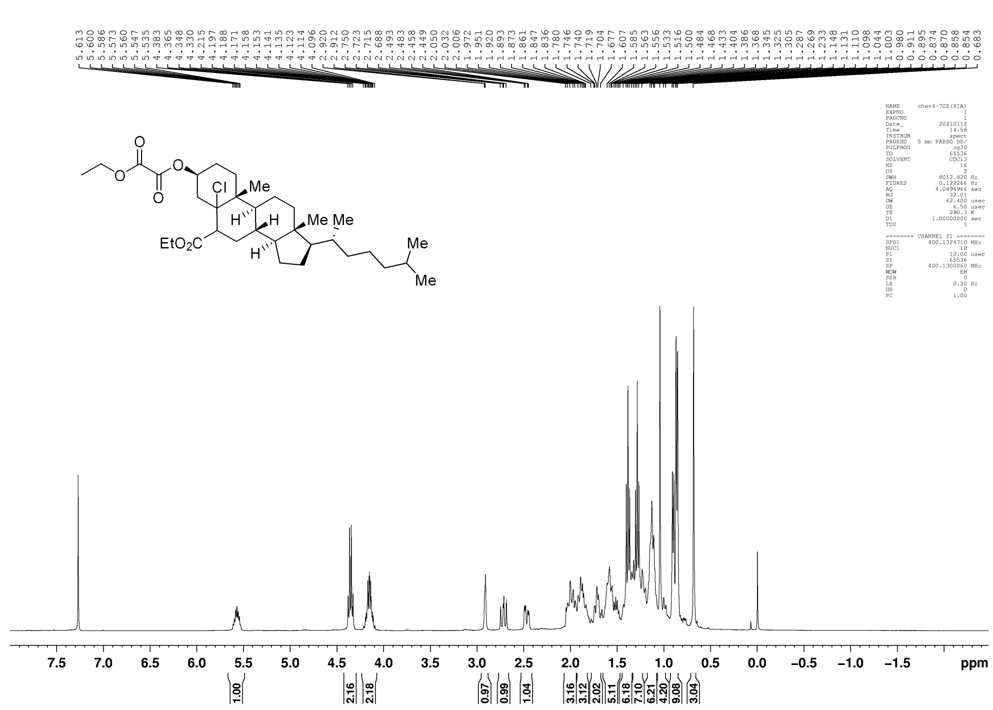

Supplementary Figure 91. ^{1}H NMR spectrum for compound **5n**

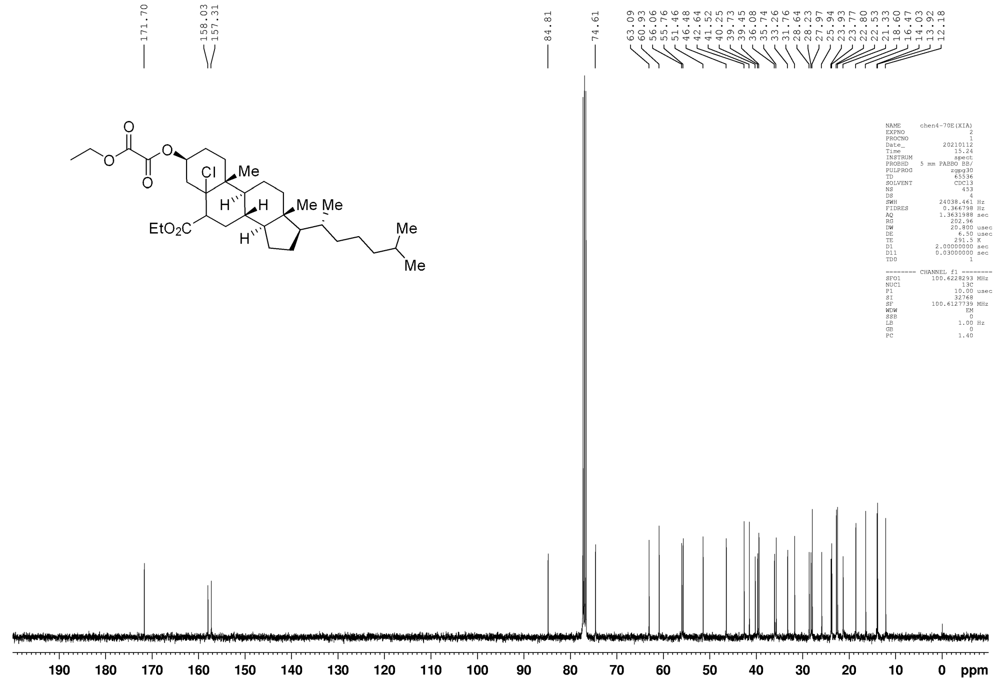

Supplementary Figure 92. ^{13}C NMR spectrum for compound **5n**

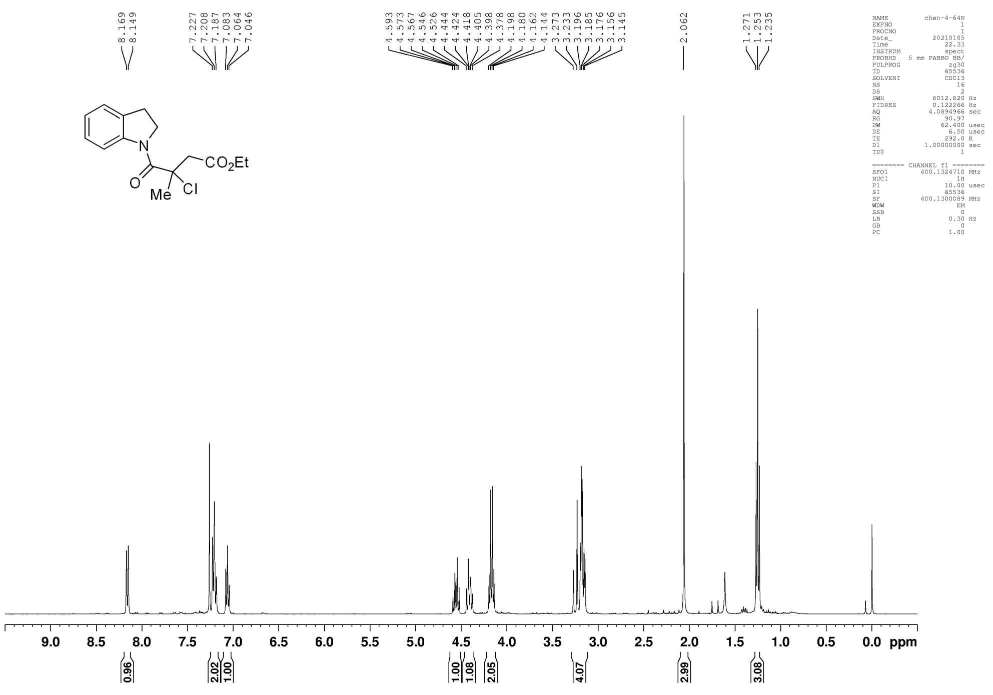

Supplementary Figure 93. ^{1}H NMR spectrum for compound **5o**

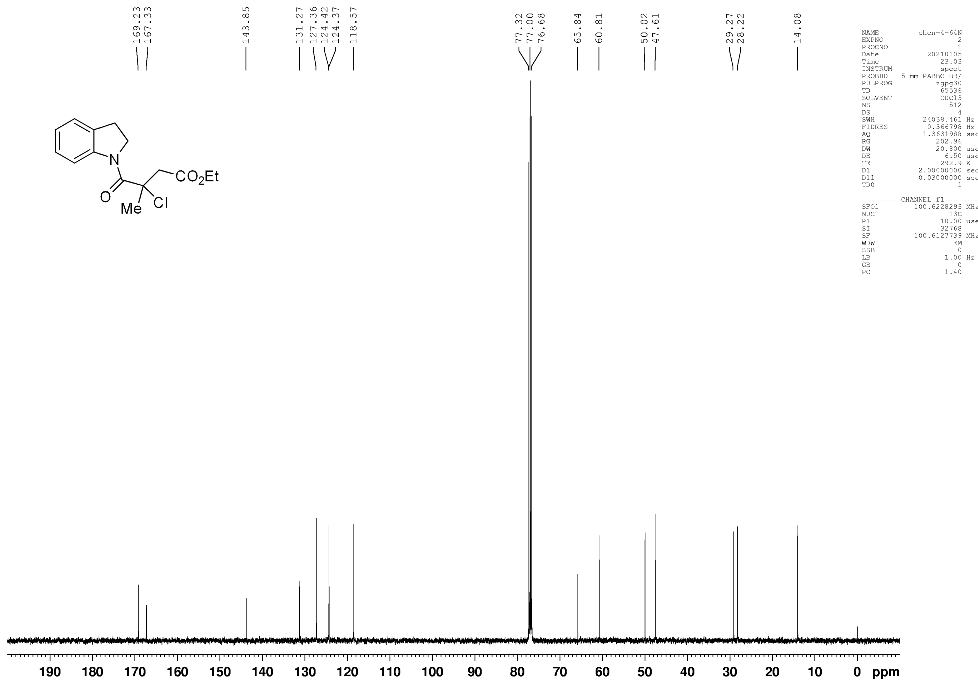

Supplementary Figure 94. ^{13}C NMR spectrum for compound **5o**

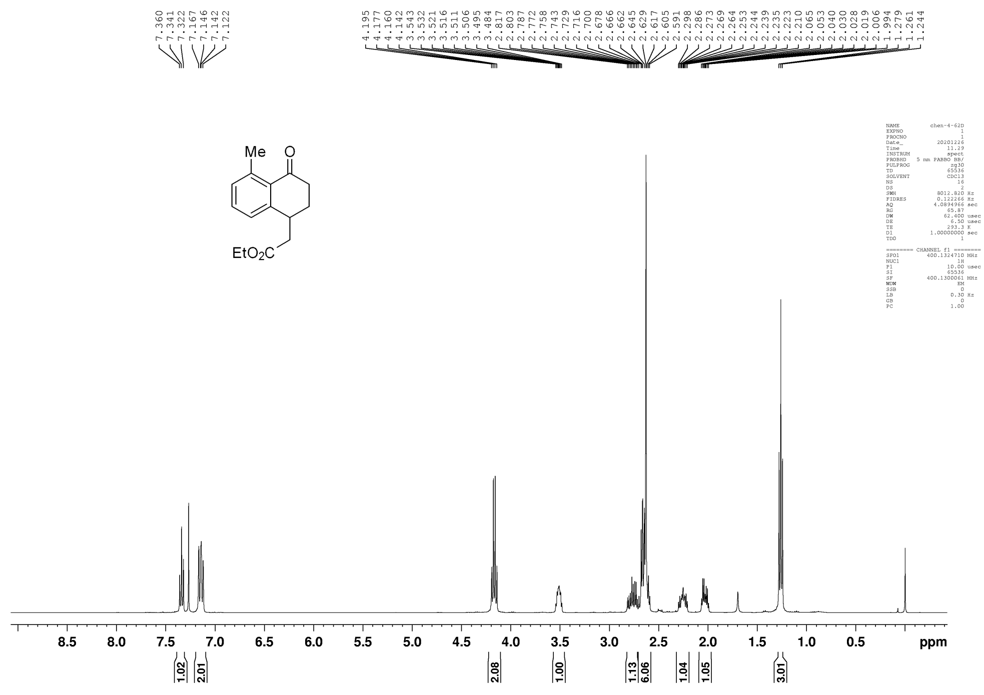

Supplementary Figure 95. ^{1}H NMR spectrum for compound **5a'**

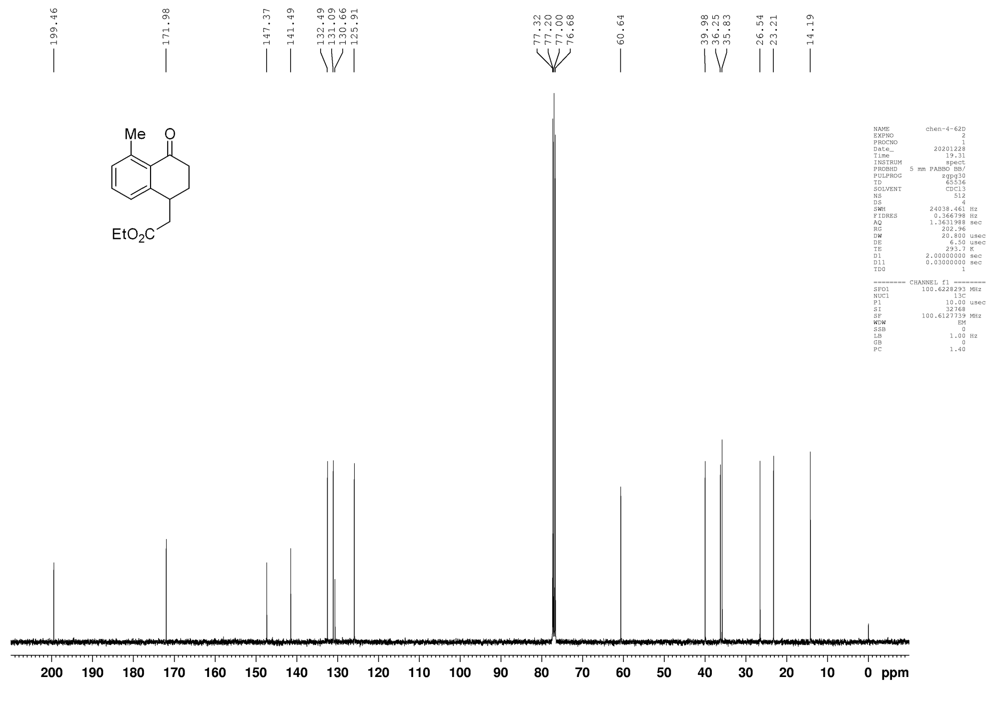

Supplementary Figure 96. ^{13}C NMR spectrum for compound **5a'**

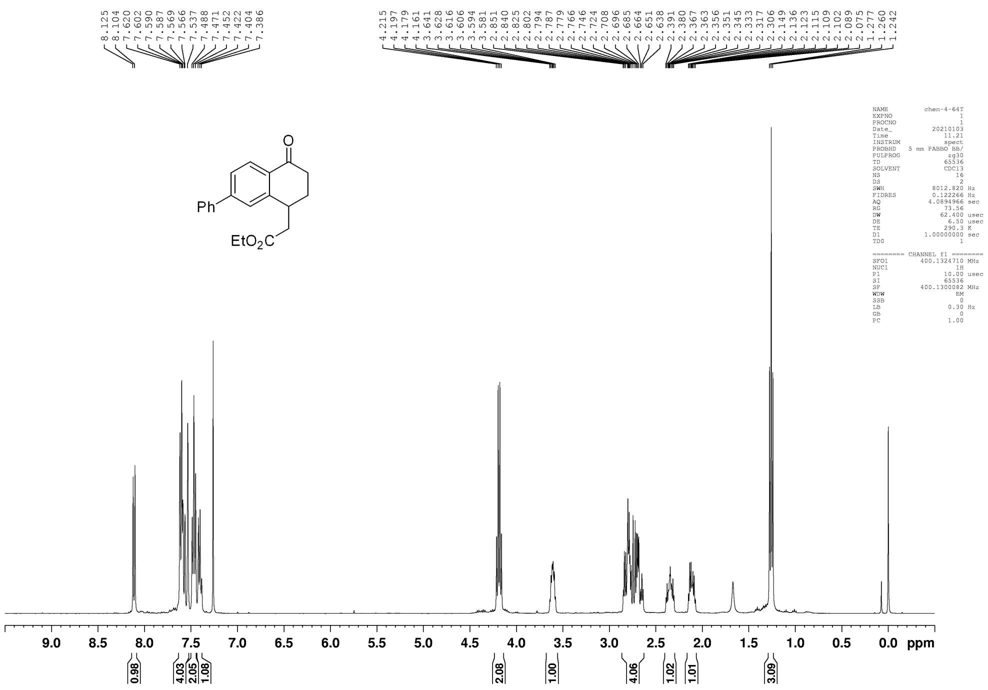

Supplementary Figure 97. ^{1}H NMR spectrum for compound **5b’**

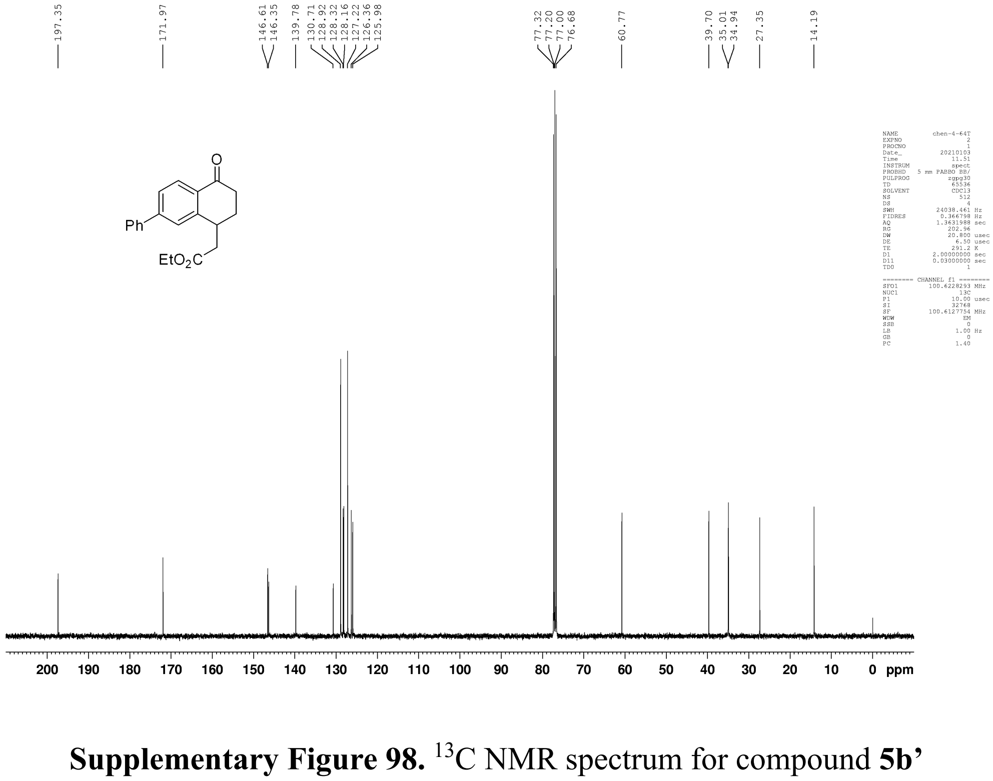

Supplementary Figure 98. ^{13}C NMR spectrum for compound **5b’**

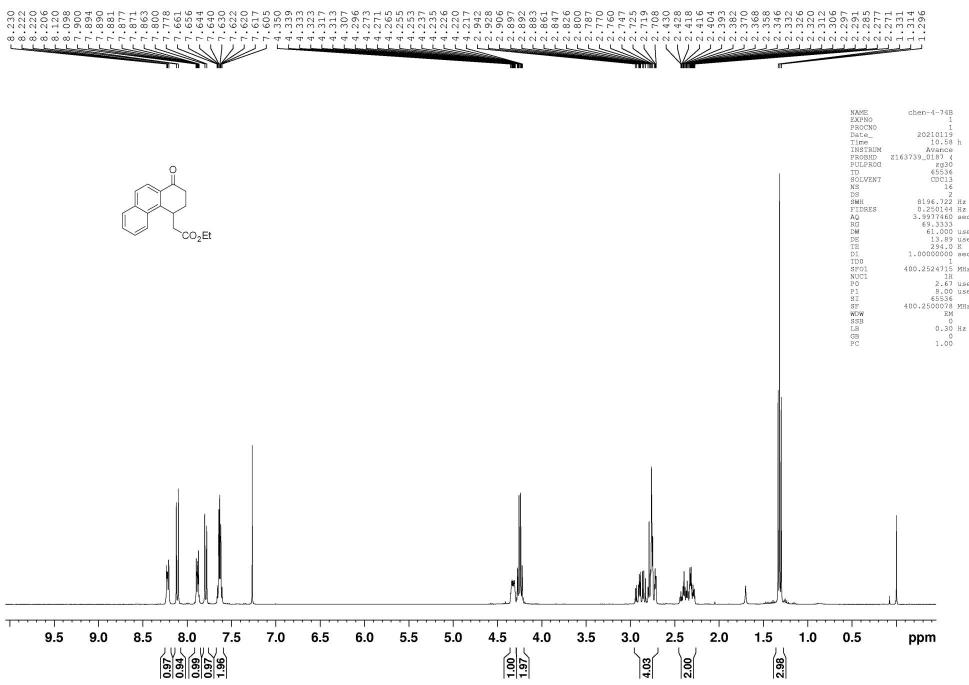

Supplementary Figure 99. ^{1}H NMR spectrum for compound **5c'**

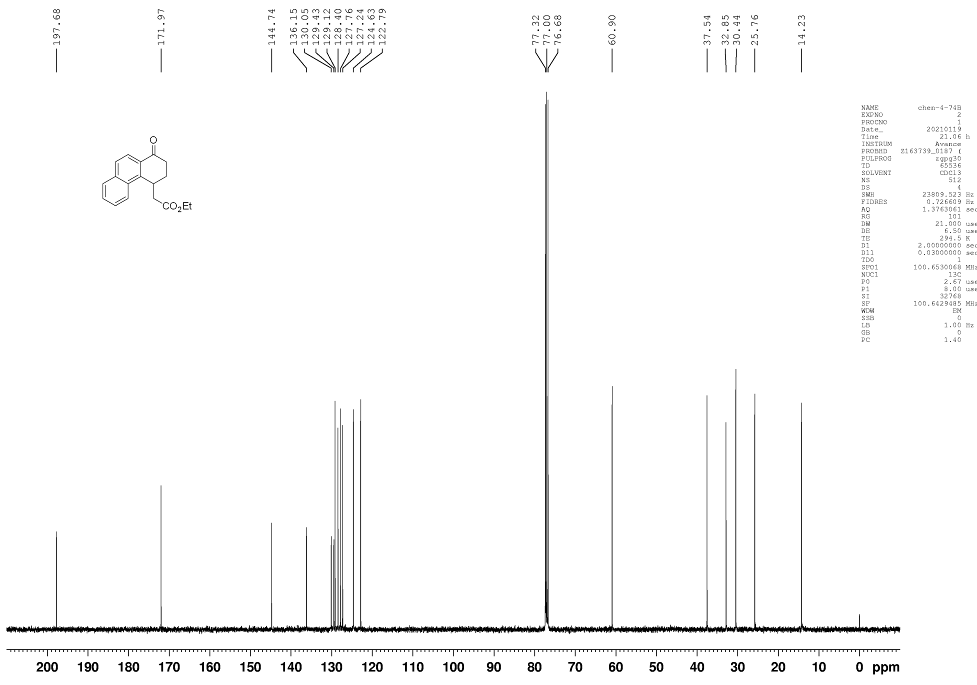

Supplementary Figure 100. ^{13}C NMR spectrum for compound **5c'**

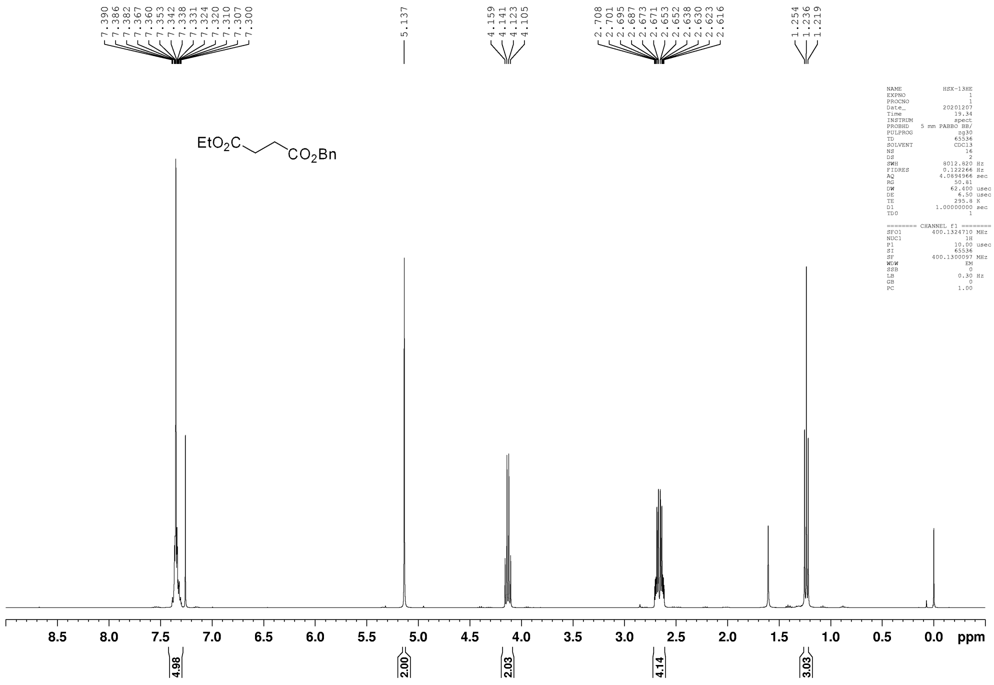

Supplementary Figure 101. ^{1}H NMR spectrum for compound **5d’**

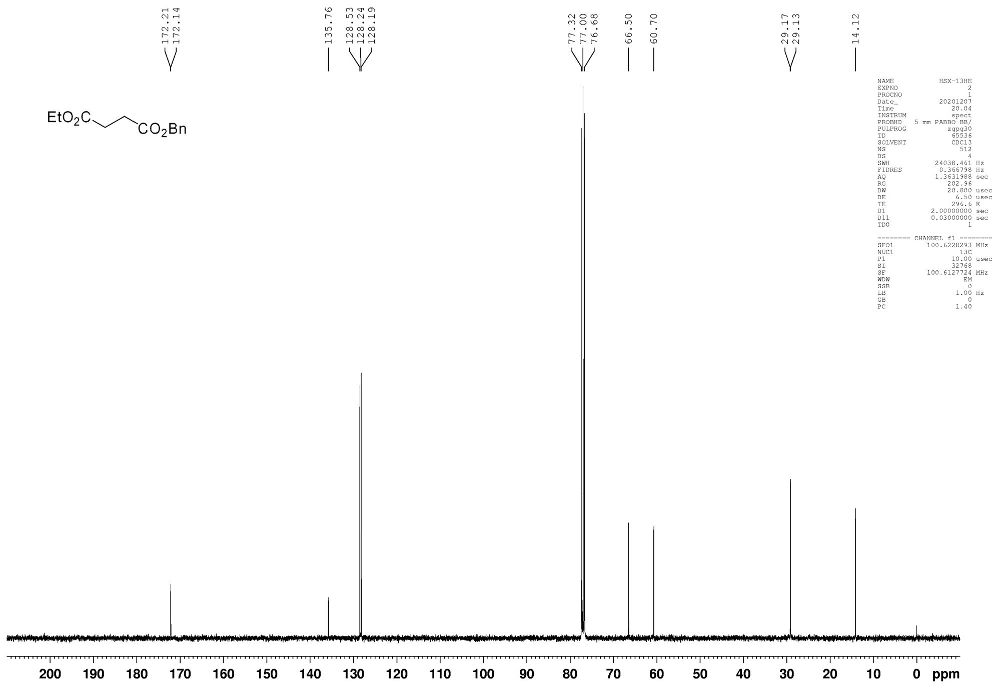

Supplementary Figure 102. ^{13}C NMR spectrum for compound **5d’**

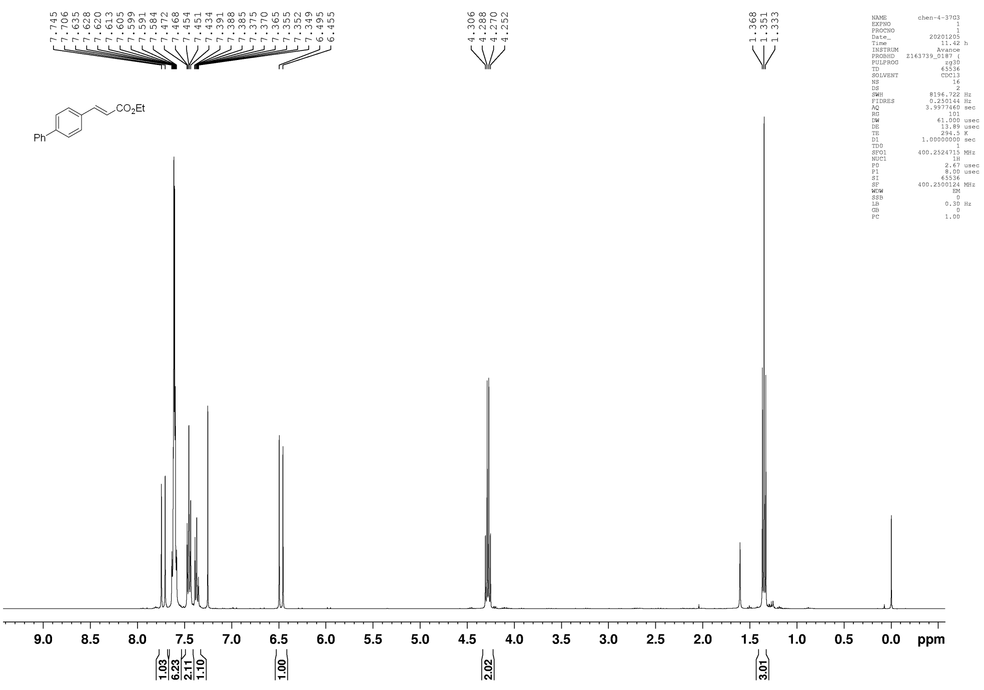

Supplementary Figure 103. ^{1}H NMR spectrum for compound **6a**

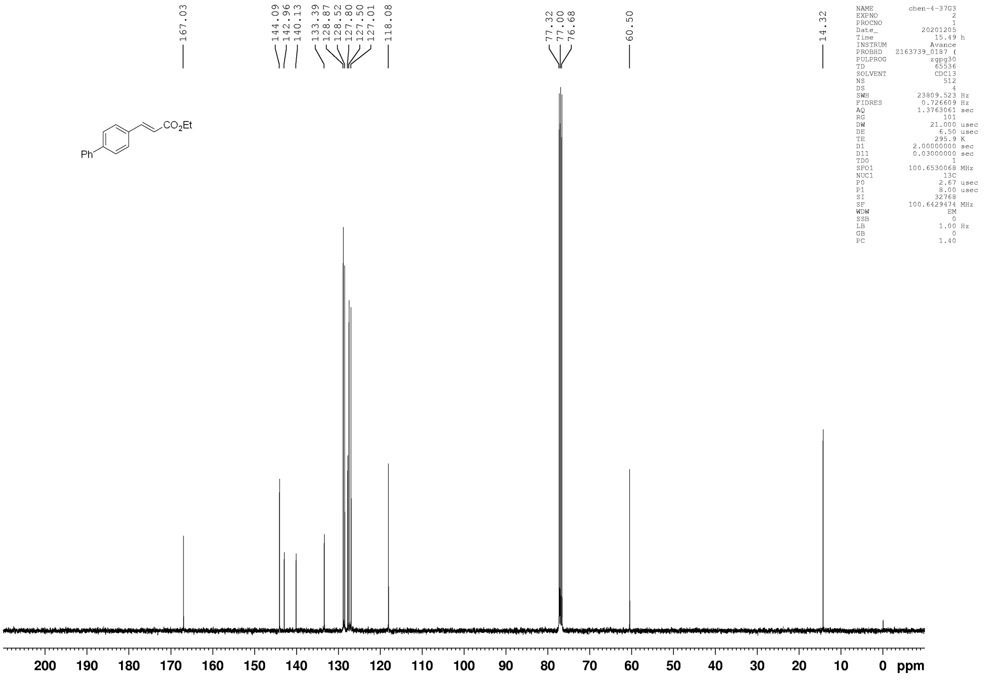

Supplementary Figure 104. ^{13}C NMR spectrum for compound **6a**

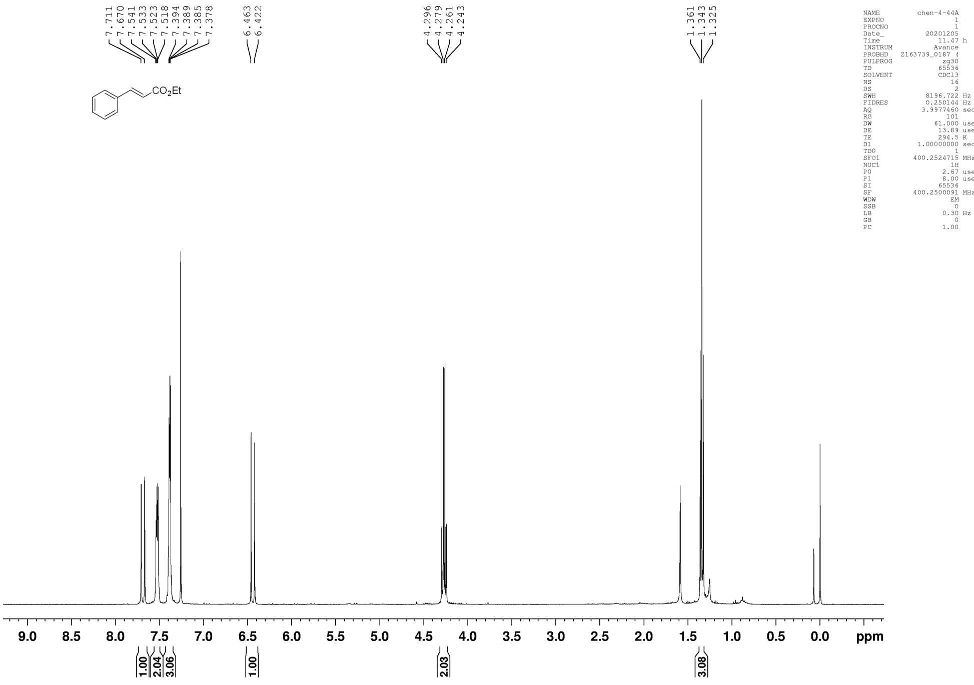

Supplementary Figure 105. ^{1}H NMR spectrum for compound **6b**

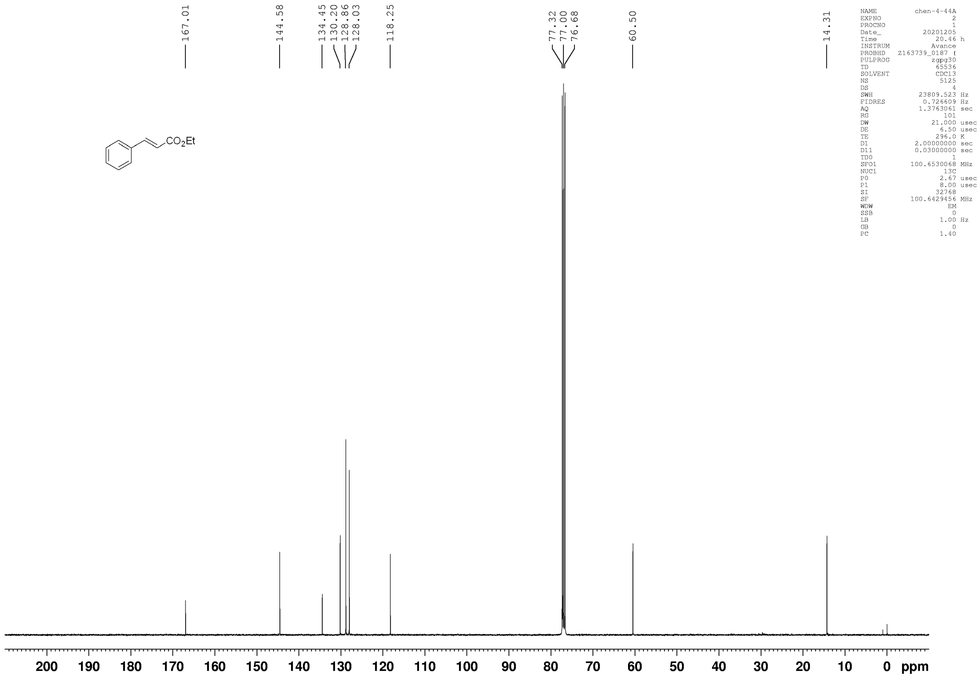

Supplementary Figure 106. ^{13}C NMR spectrum for compound **6b**

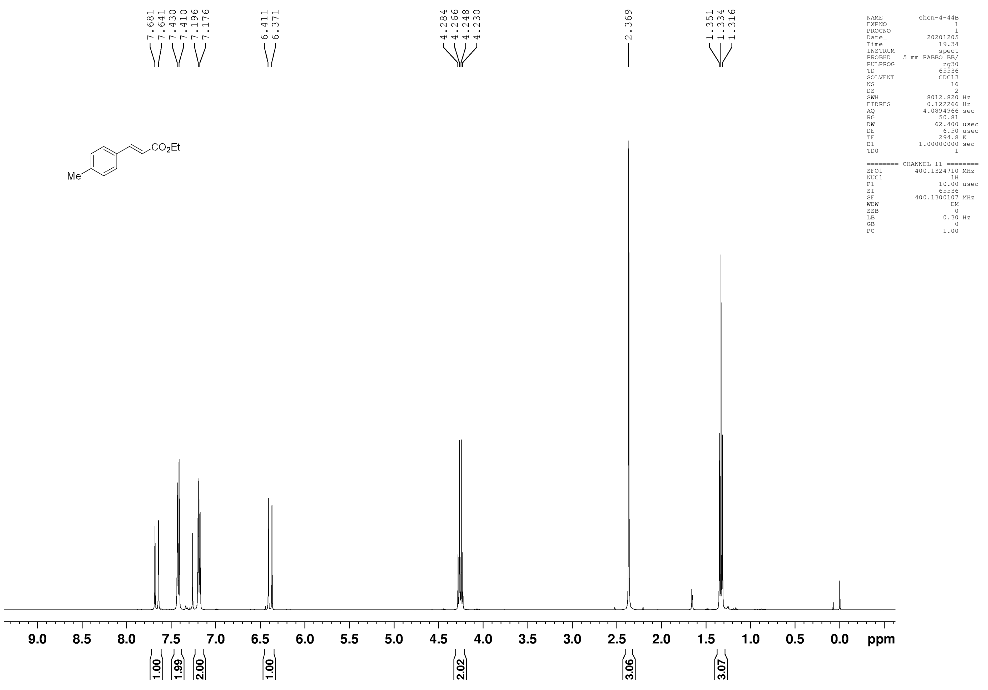

Supplementary Figure 107. ^{1}H NMR spectrum for compound **6c**

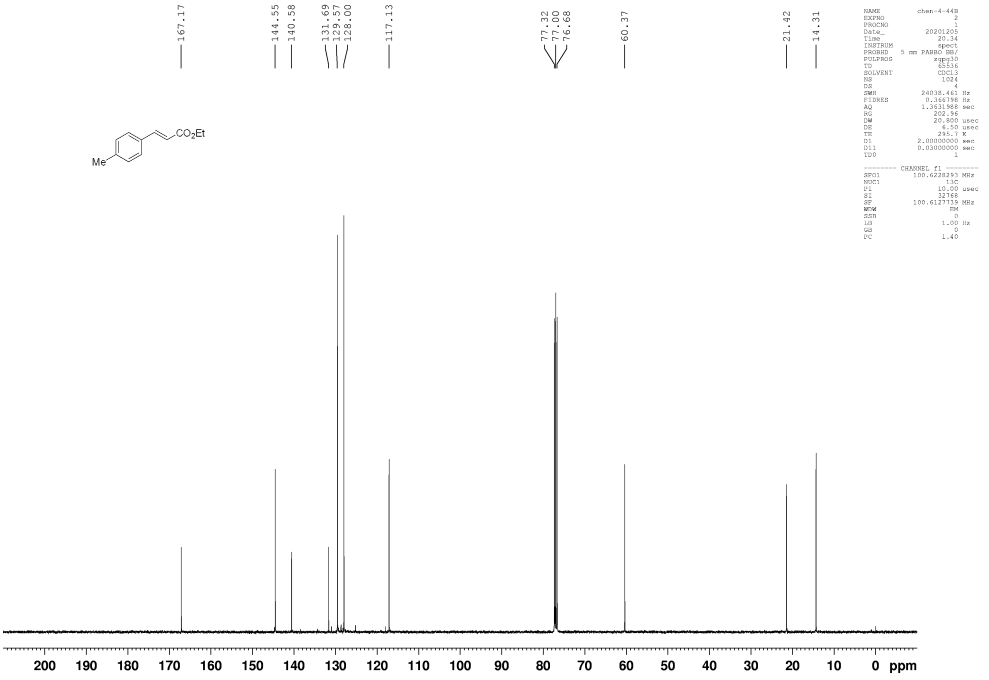

Supplementary Figure 108. ^{13}C NMR spectrum for compound **6c**

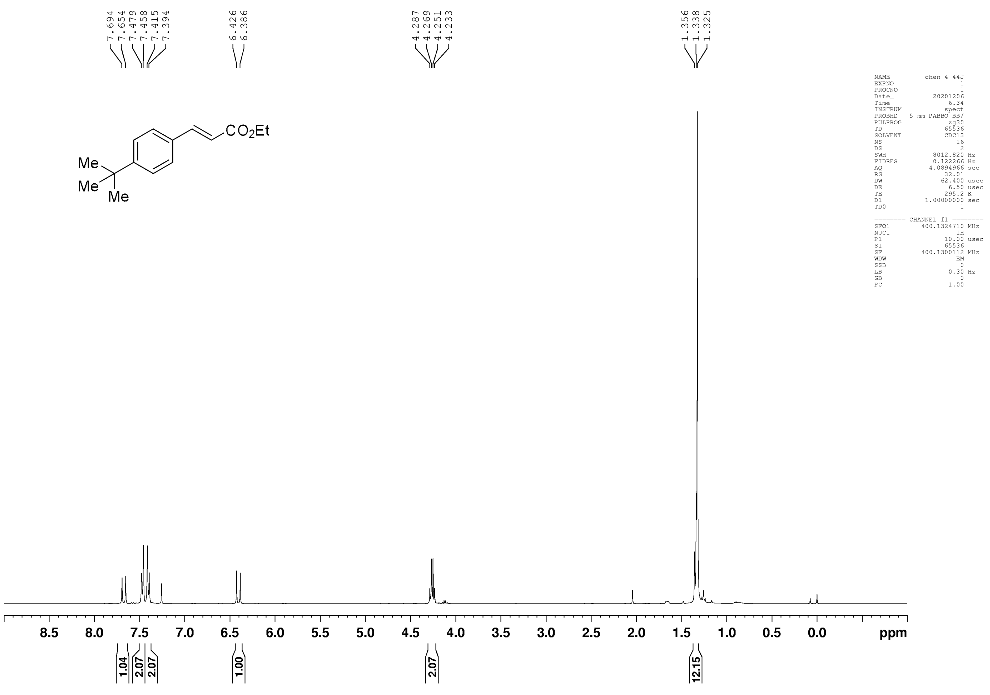

Supplementary Figure 109. ^{1}H NMR spectrum for compound **6d**

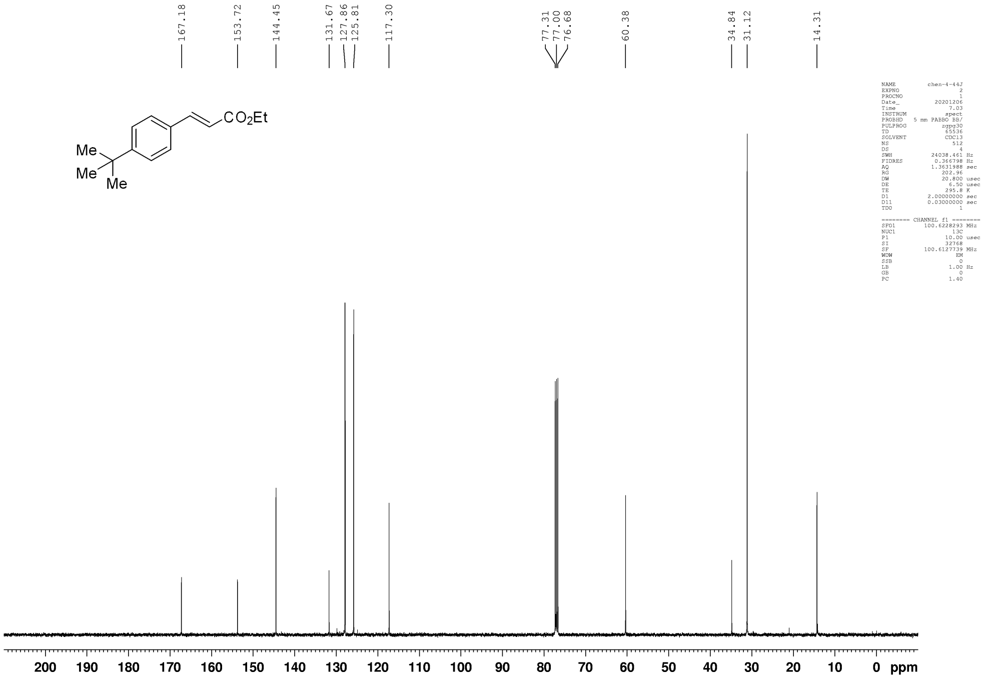

Supplementary Figure 110. ^{13}C NMR spectrum for compound **6d**

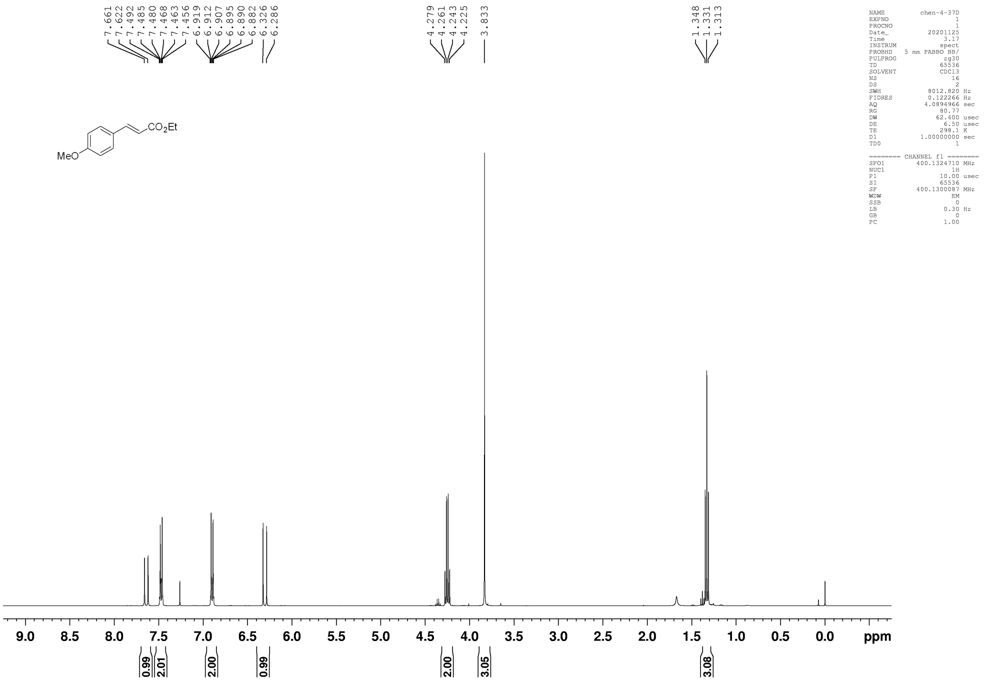

Supplementary Figure 111. ^{1}H NMR spectrum for compound **6e**

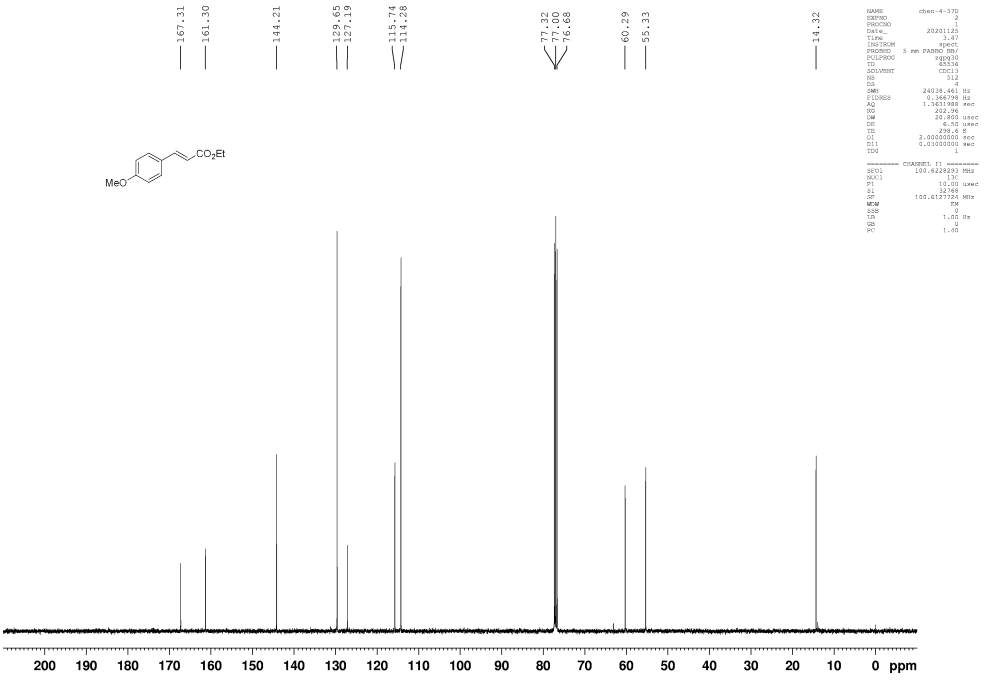

Supplementary Figure 112. ^{13}C NMR spectrum for compound **6e**

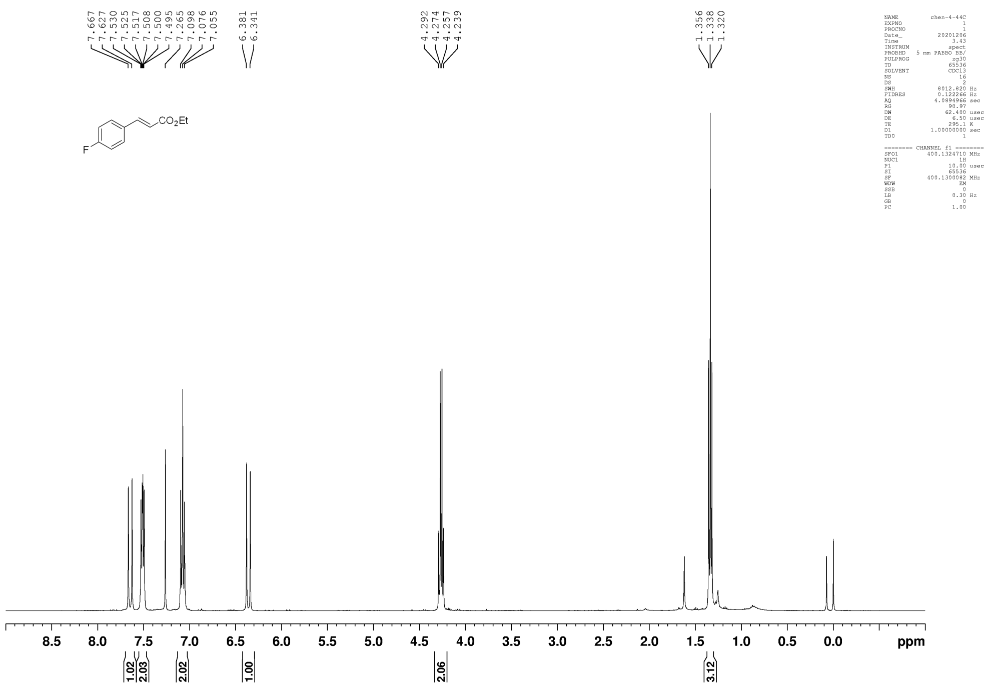

Supplementary Figure 113. ^{1}H NMR spectrum for compound **6f**

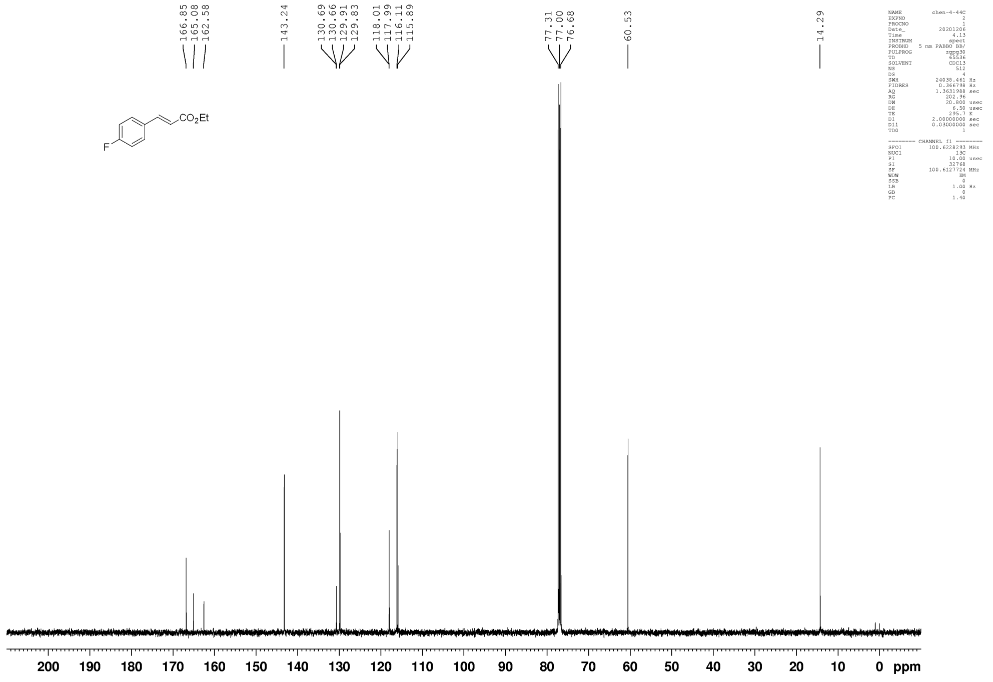

Supplementary Figure 114. ^{13}C NMR spectrum for compound **6f**

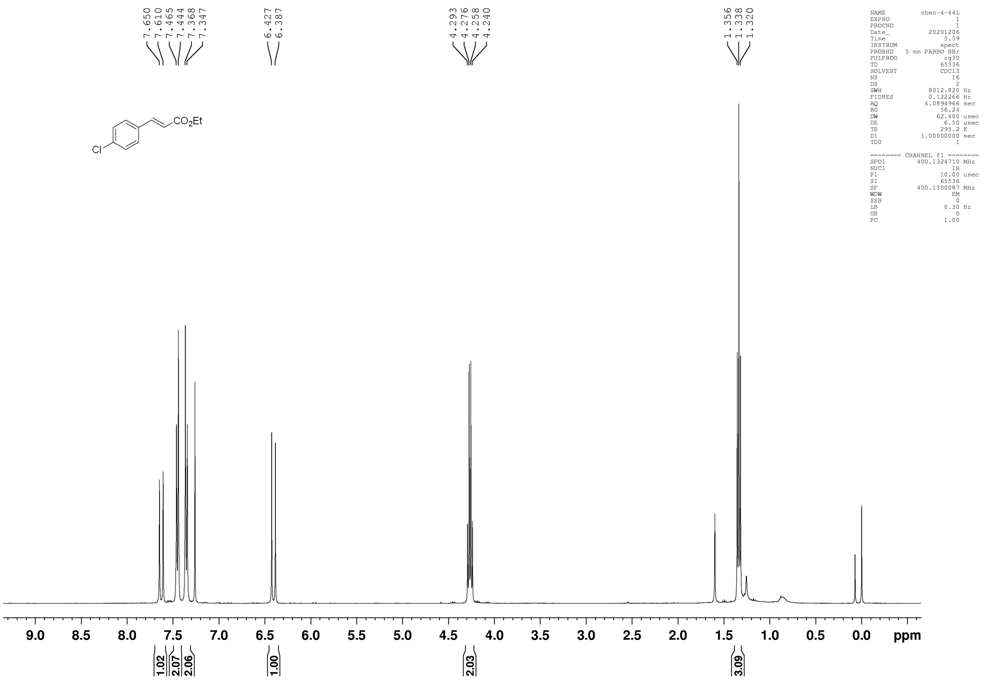

Supplementary Figure 115. ^{1}H NMR spectrum for compound **6g**

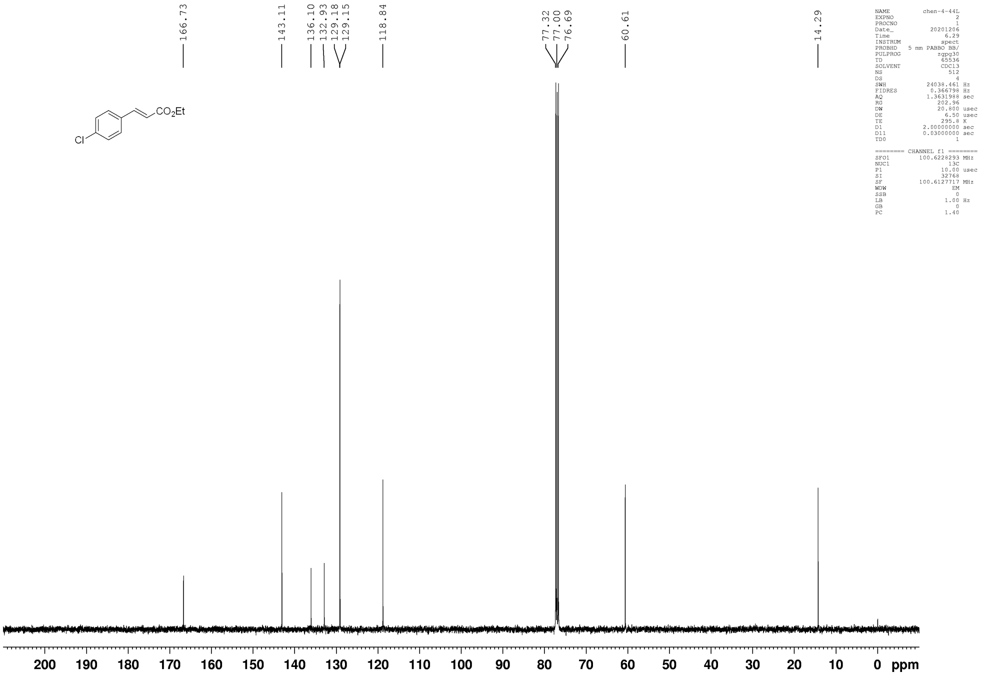

Supplementary Figure 116. ^{13}C NMR spectrum for compound **6g**

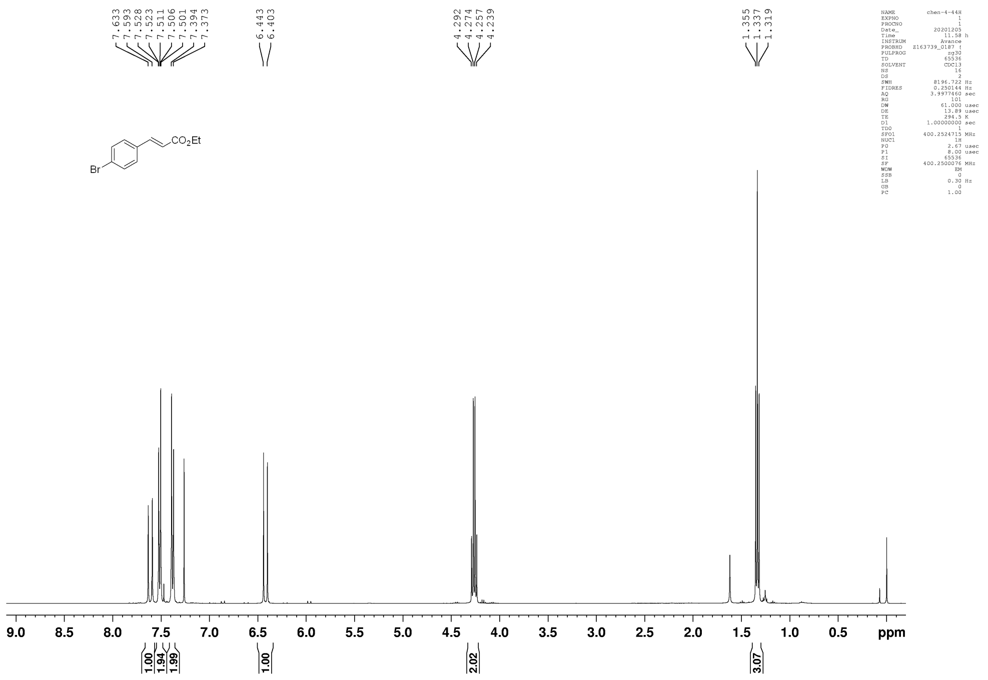

Supplementary Figure 117. ^{1}H NMR spectrum for compound **6h**

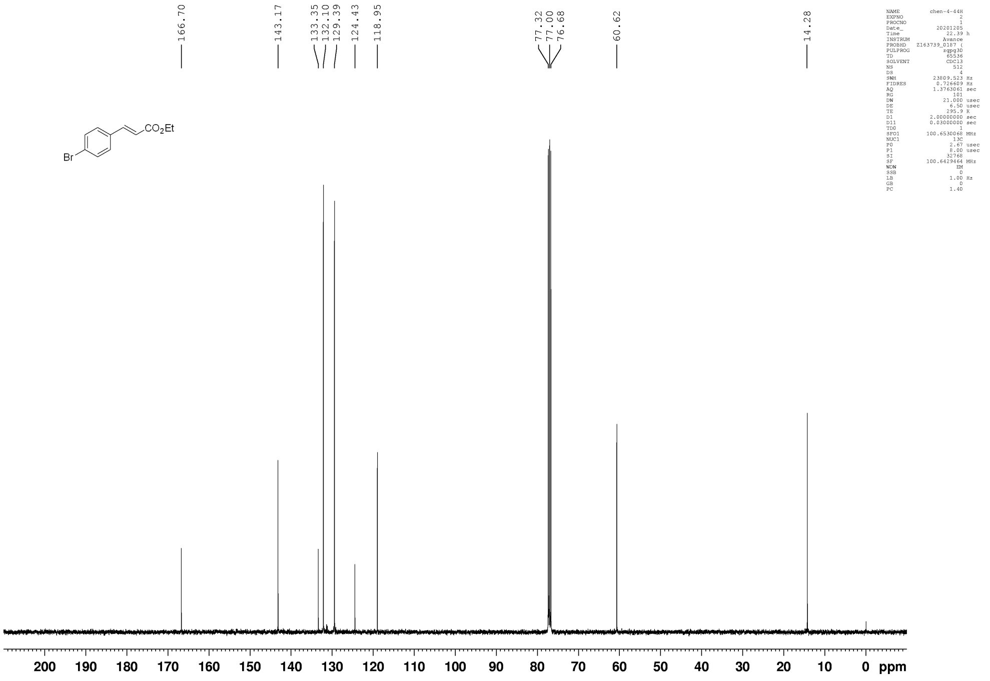

Supplementary Figure 118. ^{13}C NMR spectrum for compound **6h**

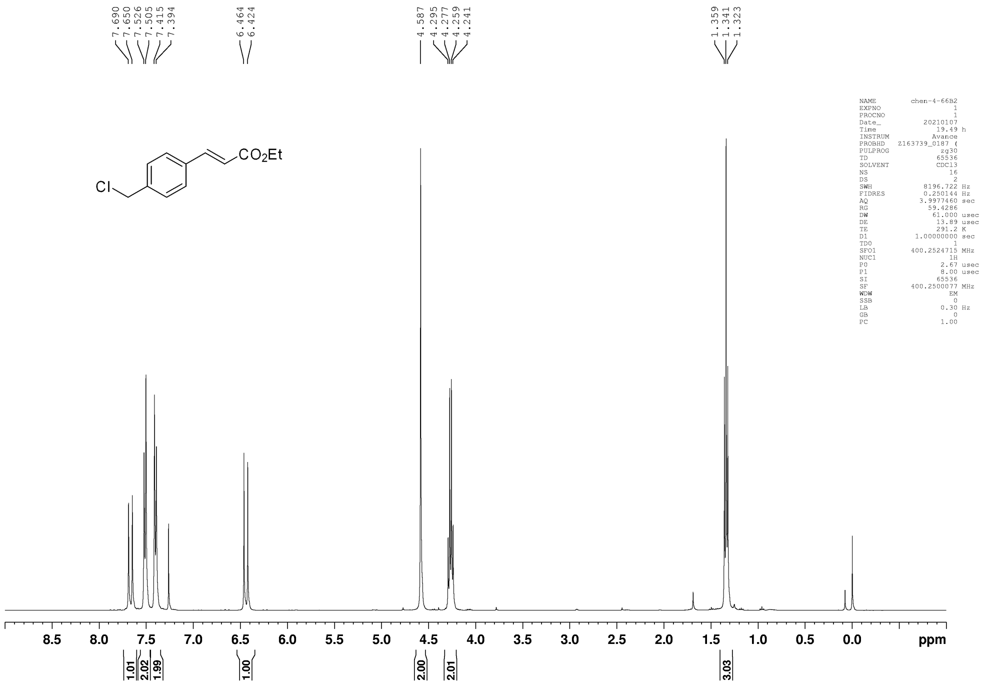

Supplementary Figure 119. ^{1}H NMR spectrum for compound **6i**

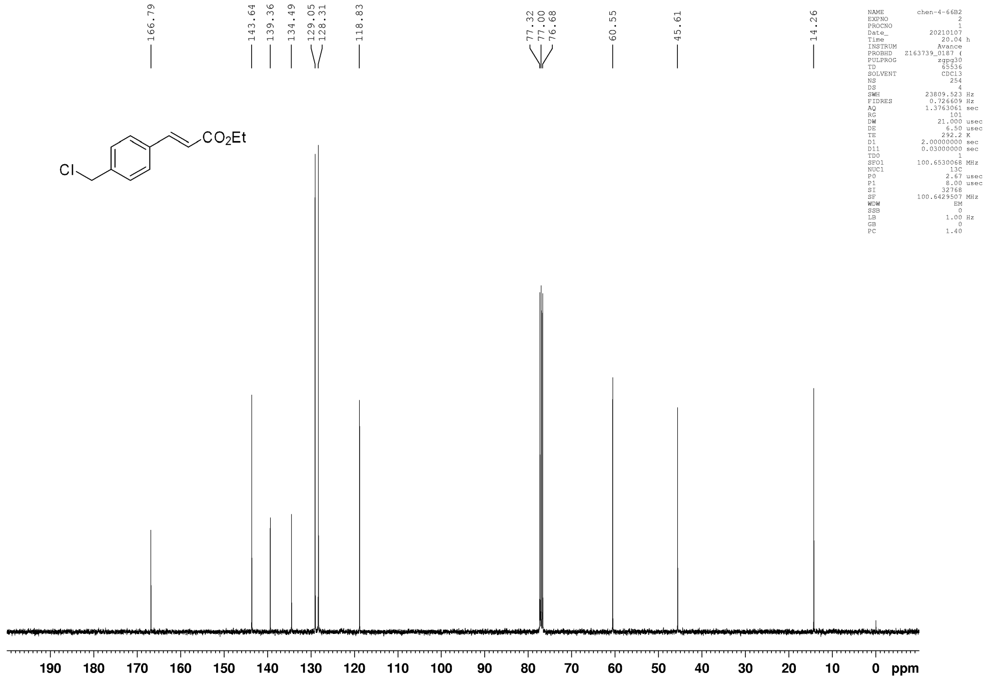

Supplementary Figure 120. ^{13}C NMR spectrum for compound **6i**

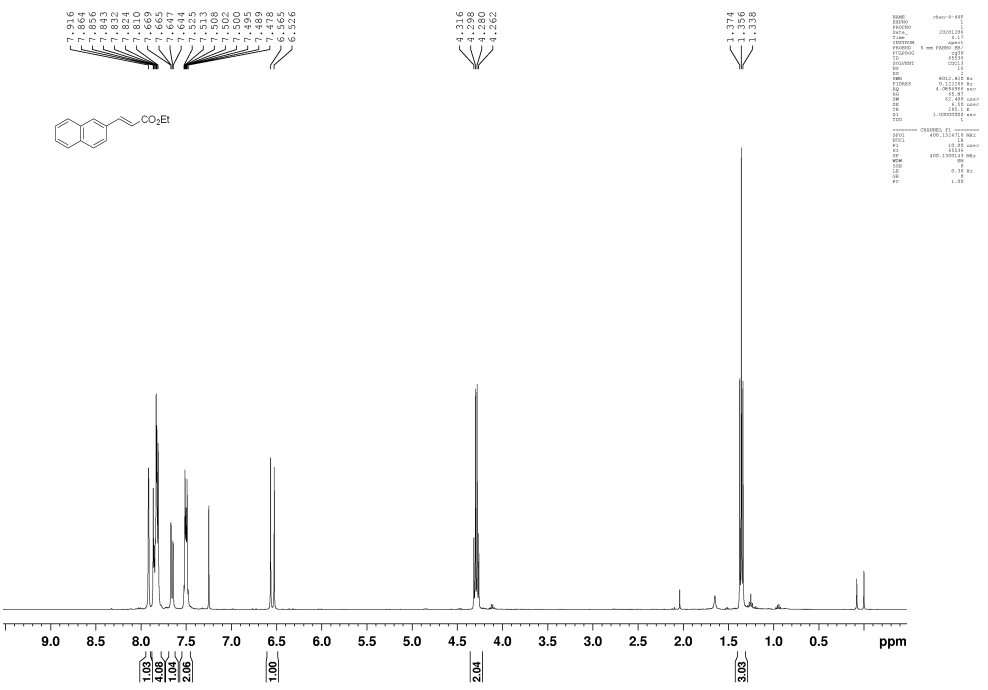

Supplementary Figure 121. ^{1}H NMR spectrum for compound **6j**

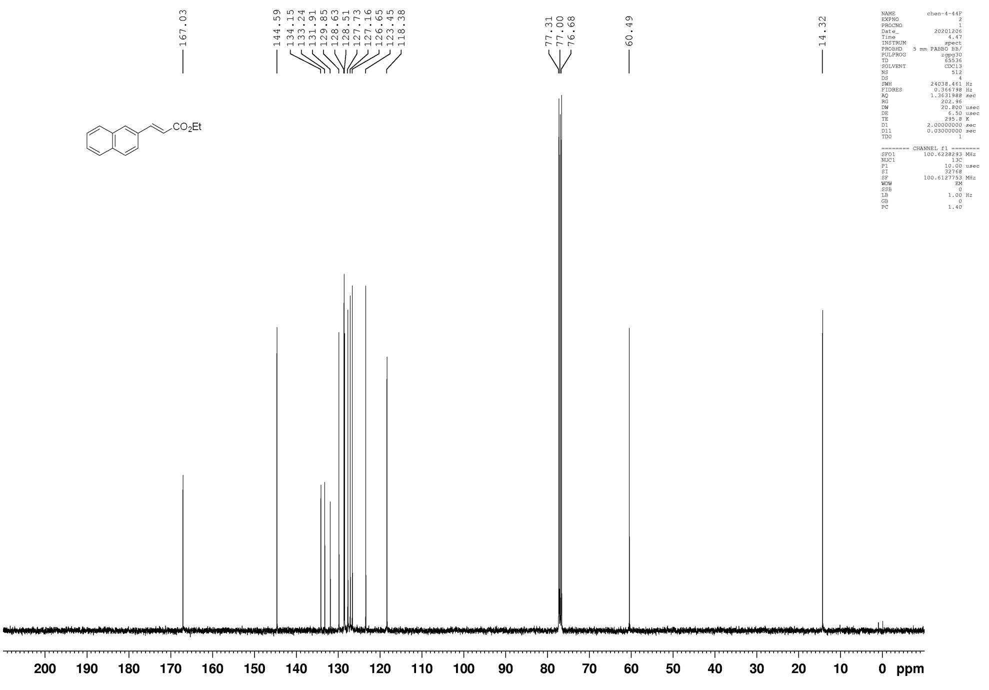

Supplementary Figure 122. ^{13}C NMR spectrum for compound **6j**

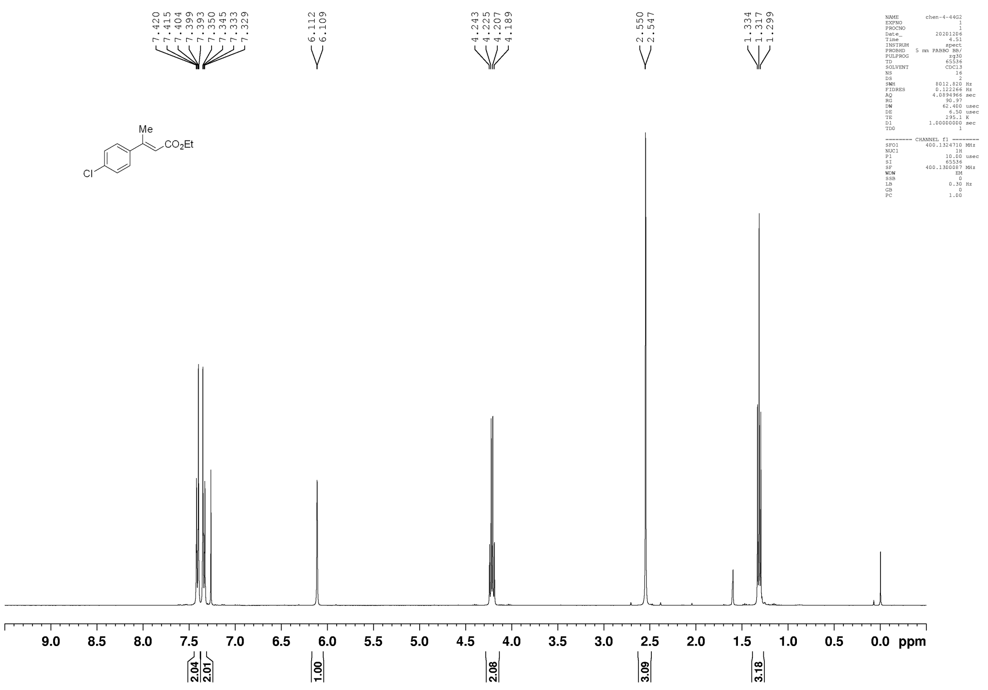

Supplementary Figure 123. ^{1}H NMR spectrum for compound **6k**

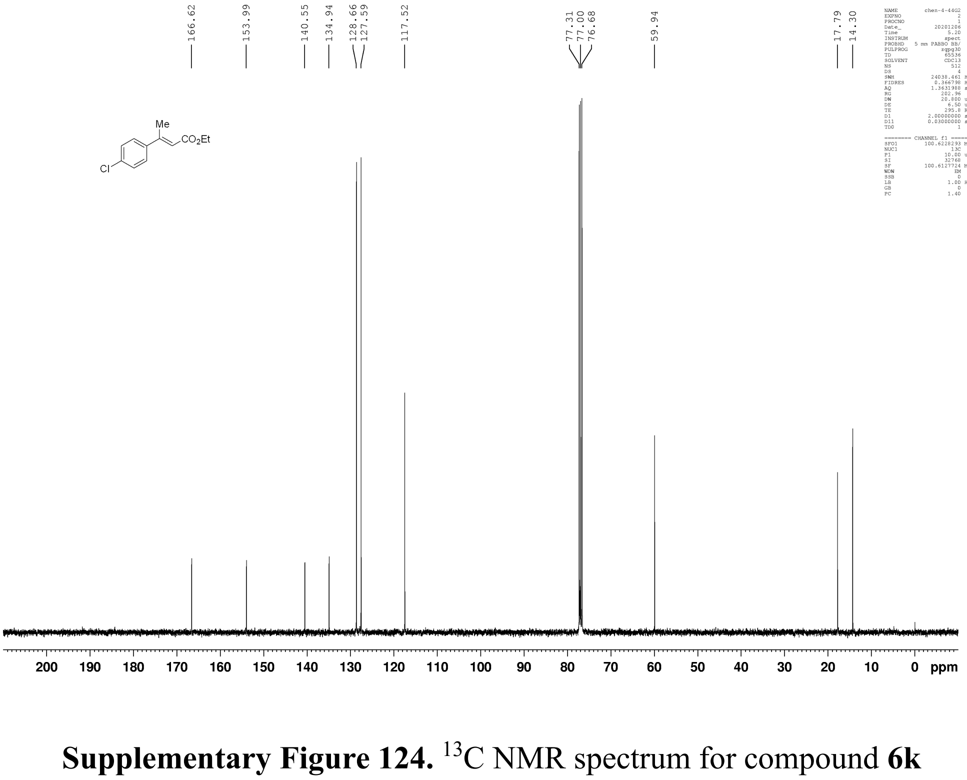

Supplementary Figure 124. ^{13}C NMR spectrum for compound **6k**

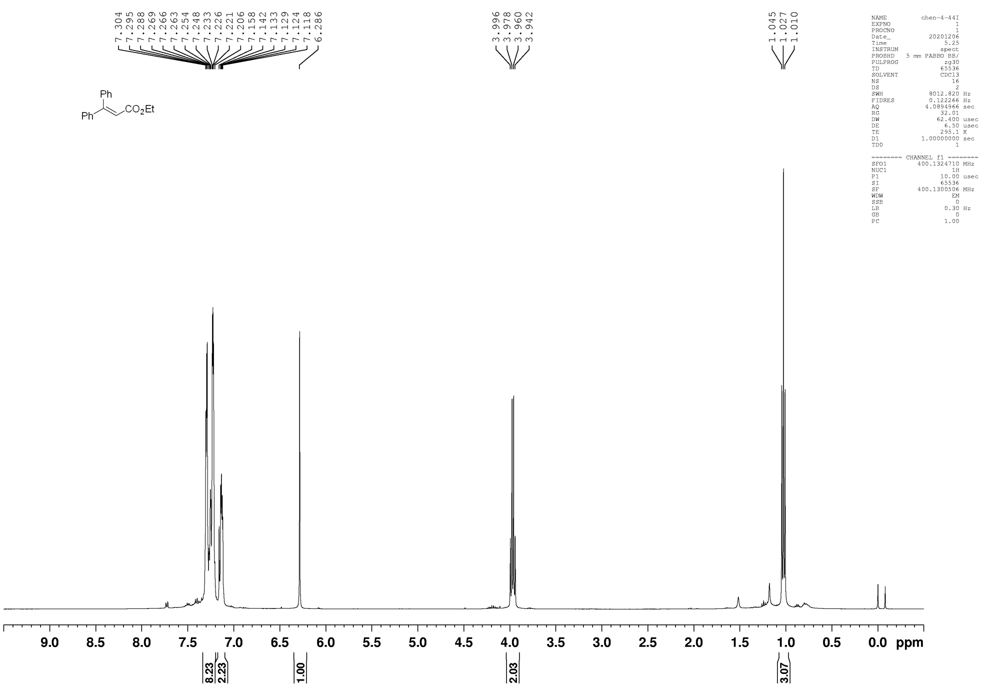

Supplementary Figure 125. ^{1}H NMR spectrum for compound **6l**

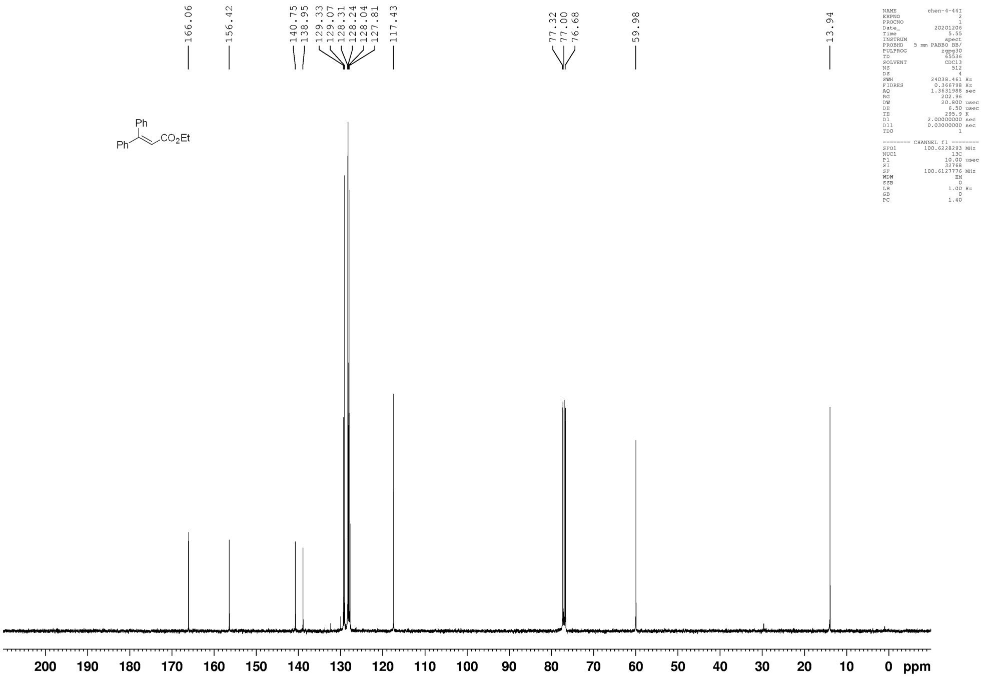

Supplementary Figure 126. ^{13}C NMR spectrum for compound **6l**

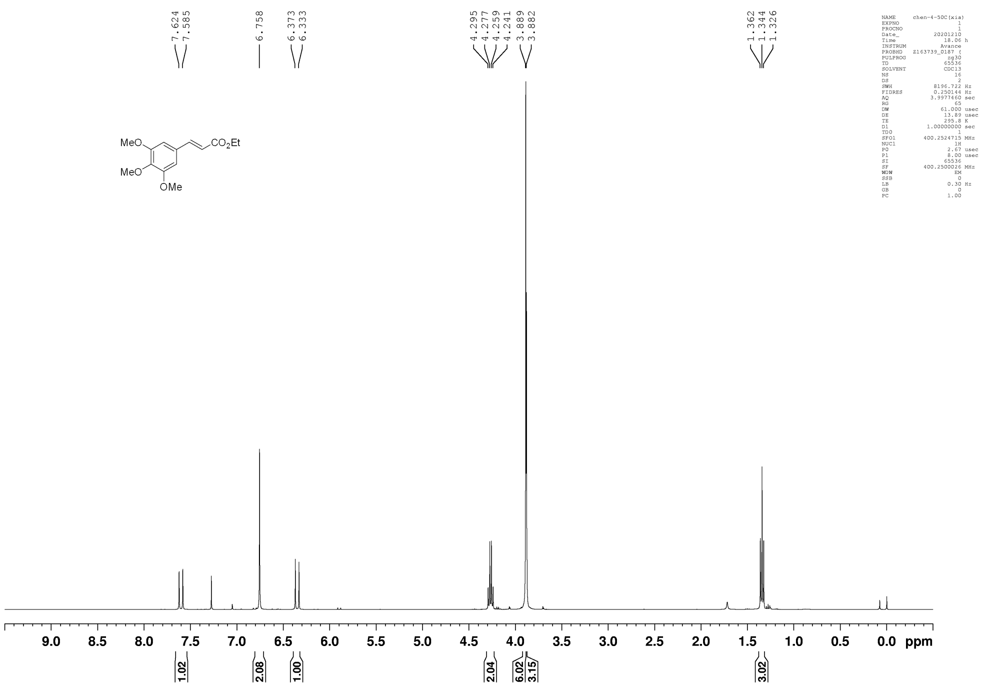

Supplementary Figure 127. ^{1}H NMR spectrum for compound **6m**

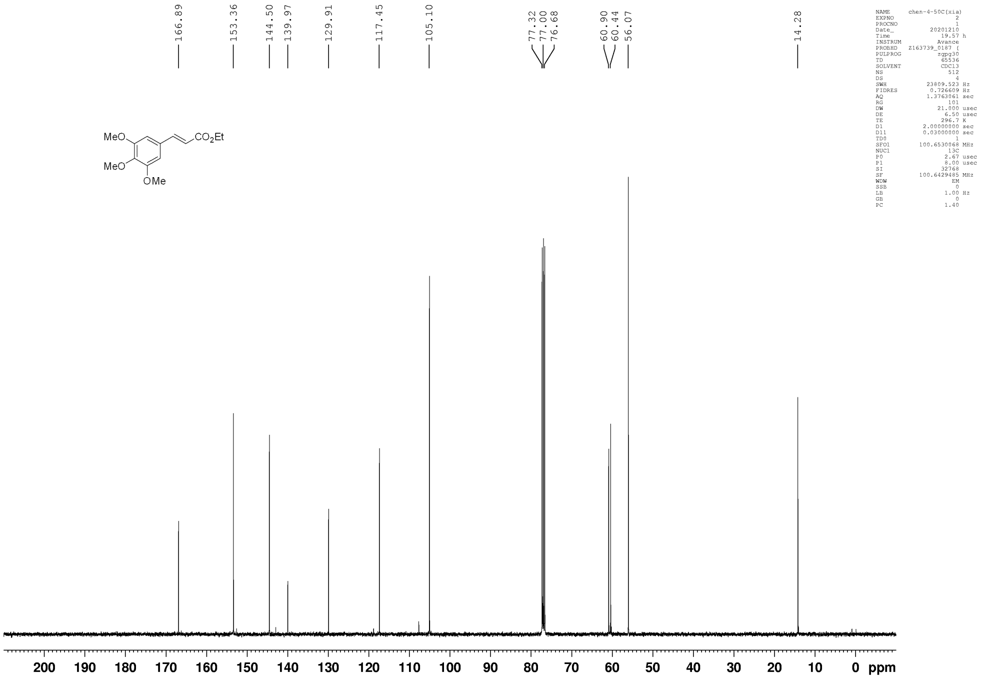

Supplementary Figure 128. ^{13}C NMR spectrum for compound **6m**

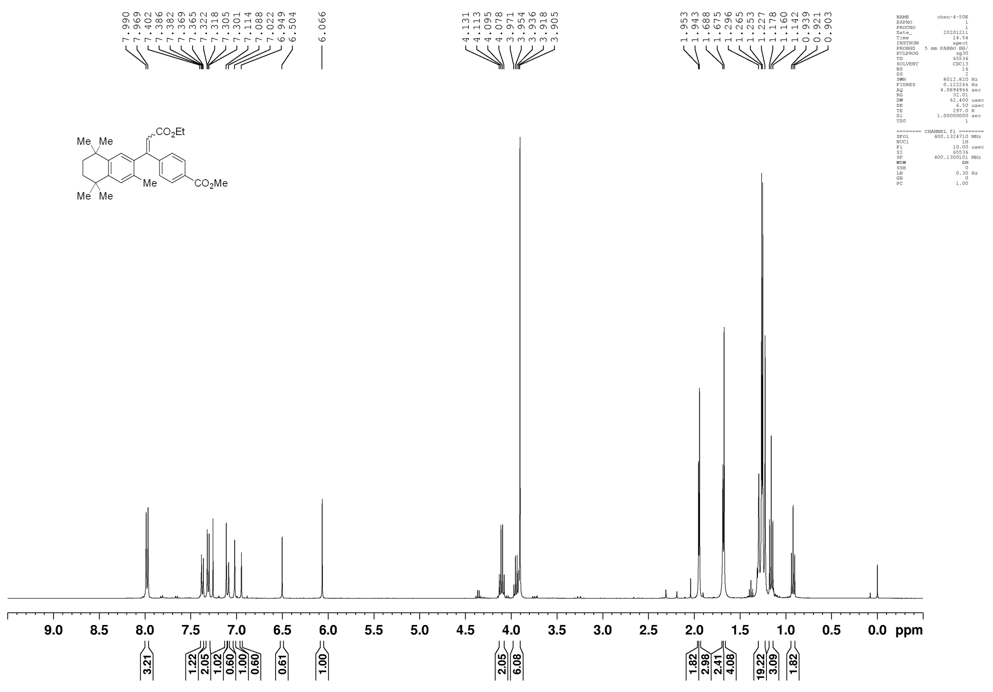

Supplementary Figure 129. ^{1}H NMR spectrum for compound **6n**

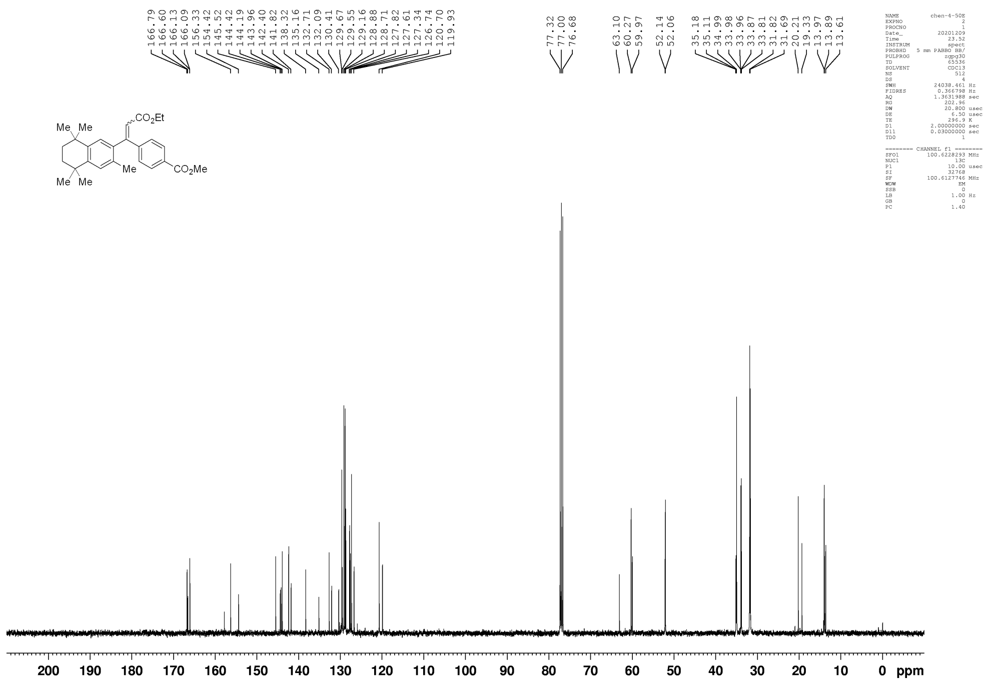

Supplementary Figure 130. ^{13}C NMR spectrum for compound **6n**

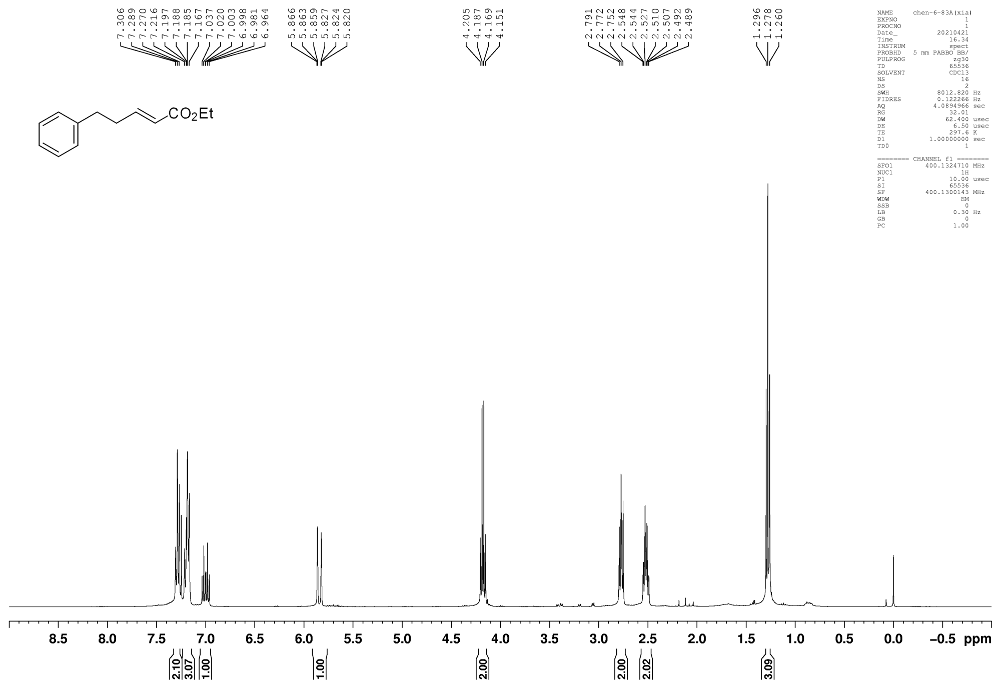

Supplementary Figure 131. ^{1}H NMR spectrum for compound **6o**

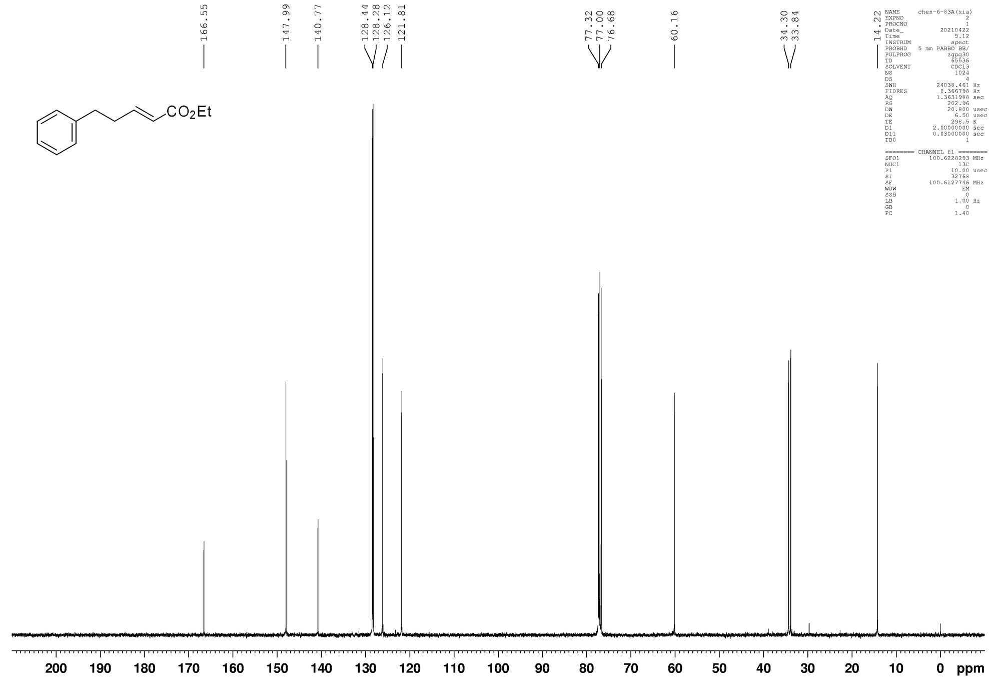

Supplementary Figure 132. ^{13}C NMR spectrum for compound **6o**

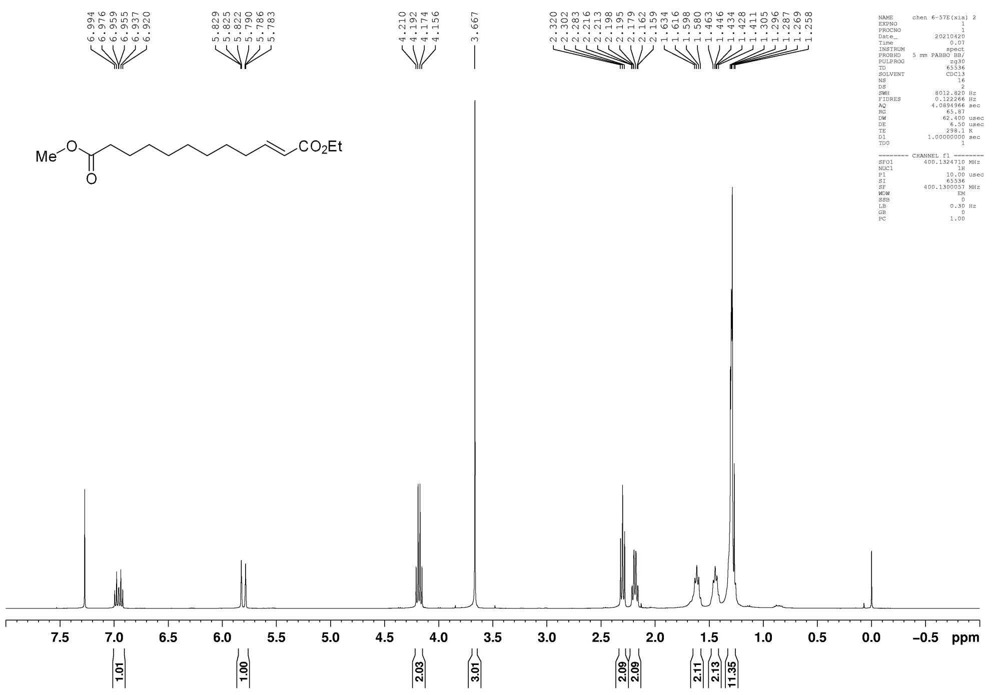

Supplementary Figure 133. ^{1}H NMR spectrum for compound **6p**

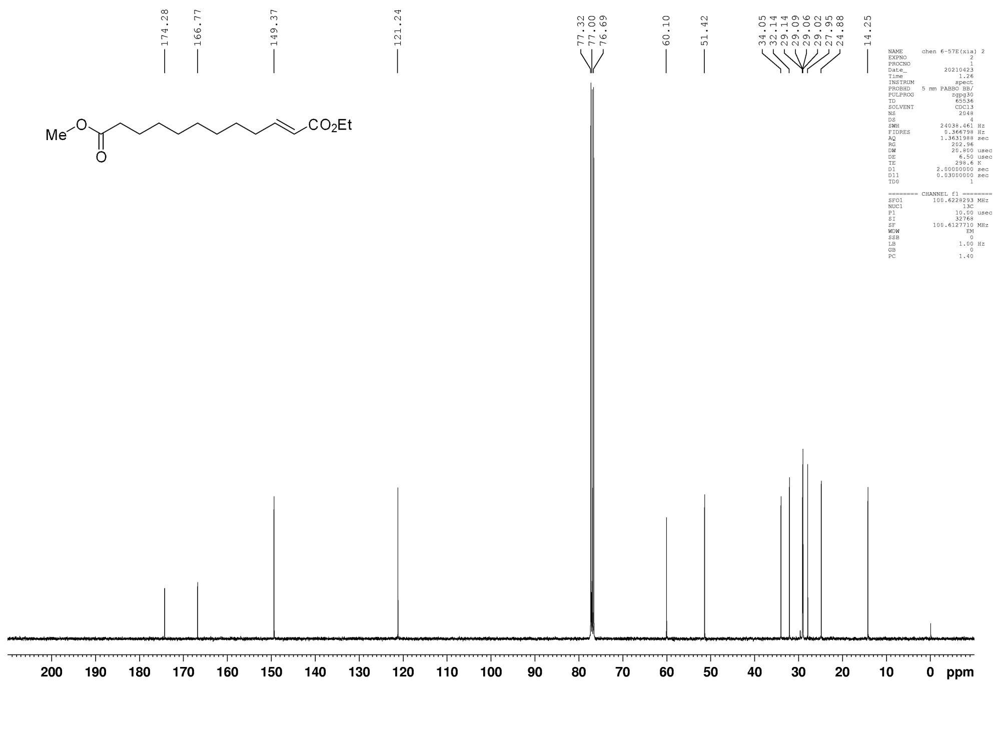

Supplementary Figure 134. ^{13}C NMR spectrum for compound **6p**

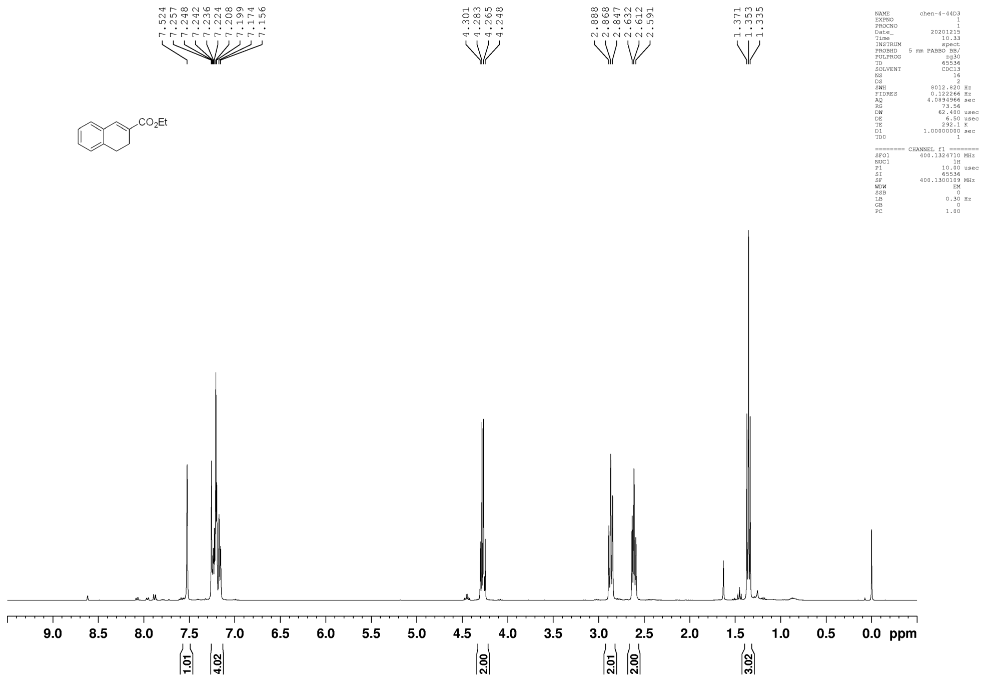

Supplementary Figure 135. ^{1}H NMR spectrum for compound **6q**

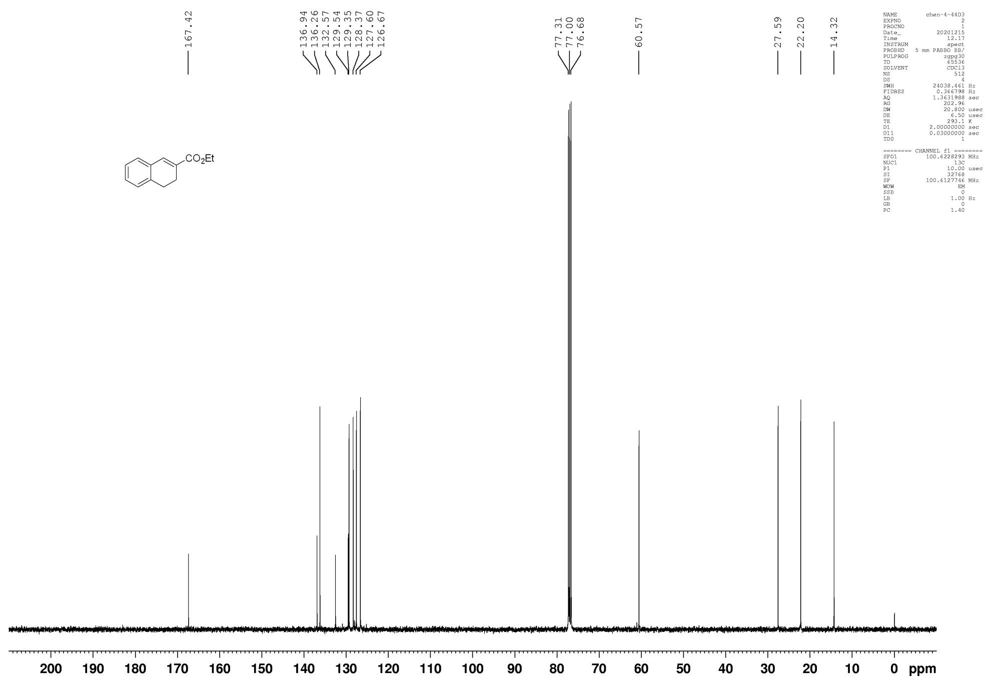

Supplementary Figure 136. ^{13}C NMR spectrum for compound **6q**

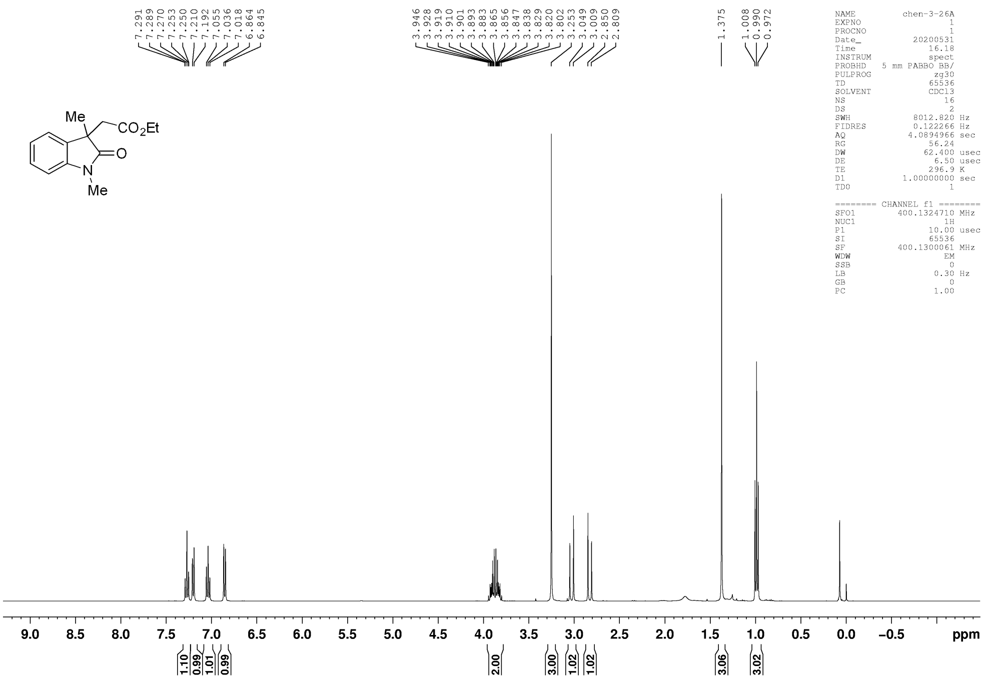

Supplementary Figure 137. ^{1}H NMR spectrum for compound **8a**

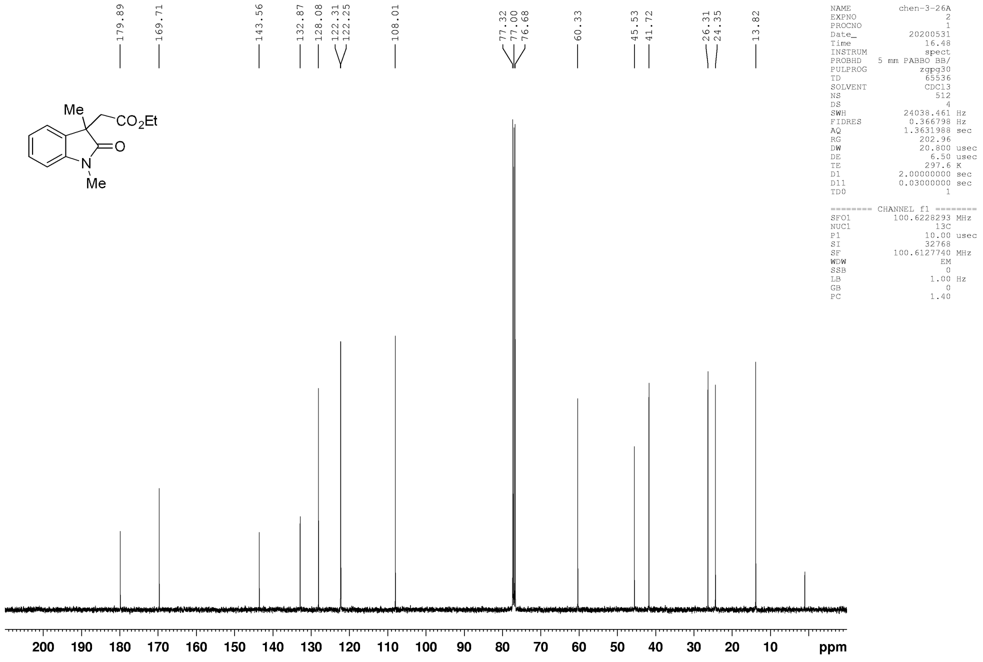

Supplementary Figure 138. ^{13}C NMR spectrum for compound **8a**

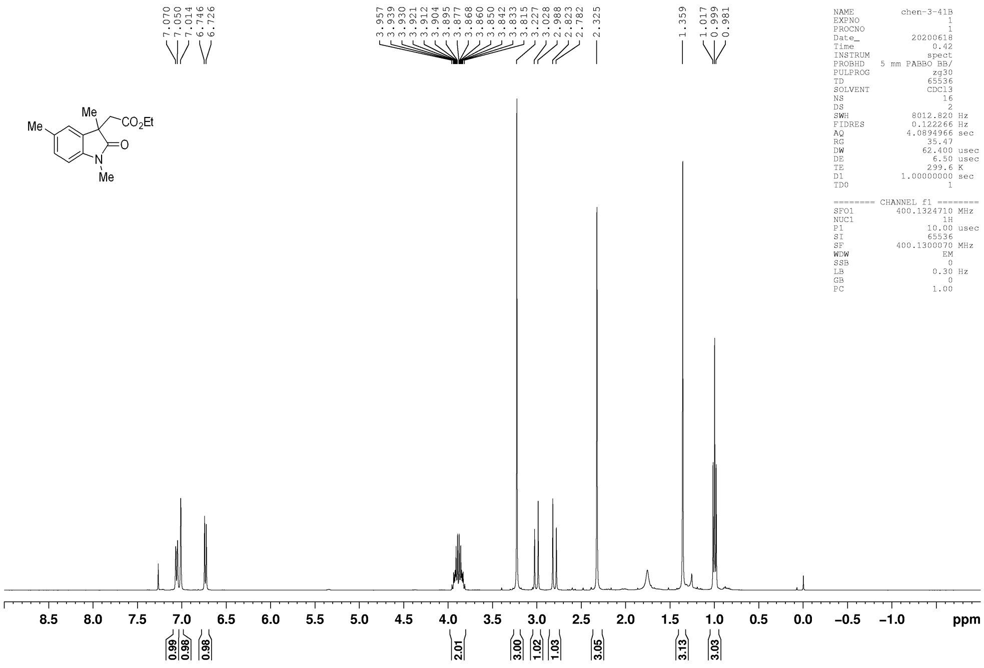

Supplementary Figure 139. ^{1}H NMR spectrum for compound **8b**

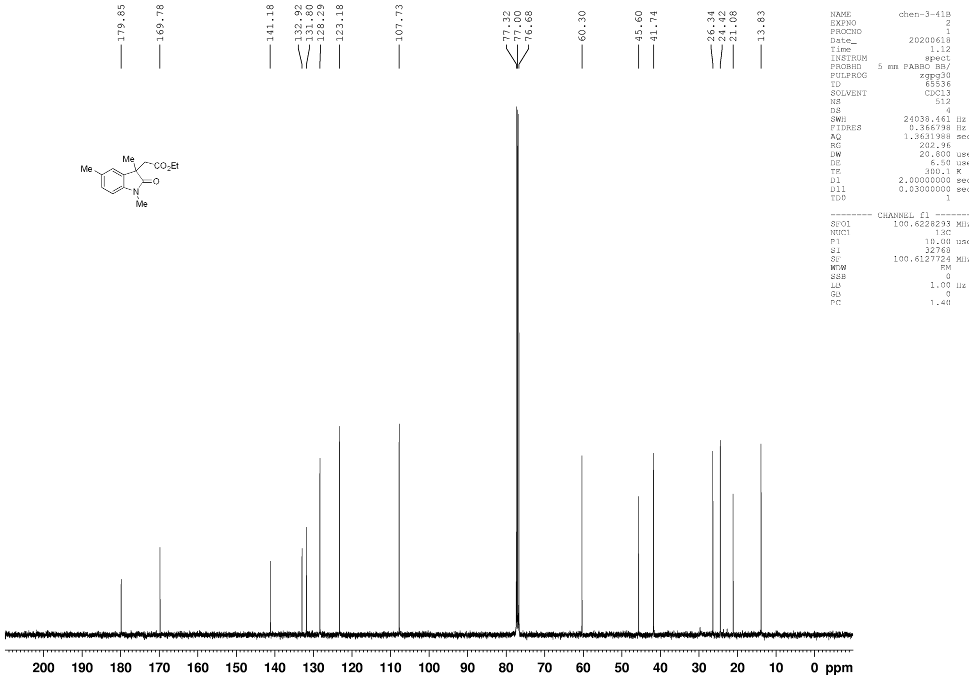

Supplementary Figure 140. ^{13}C NMR spectrum for compound **8b**

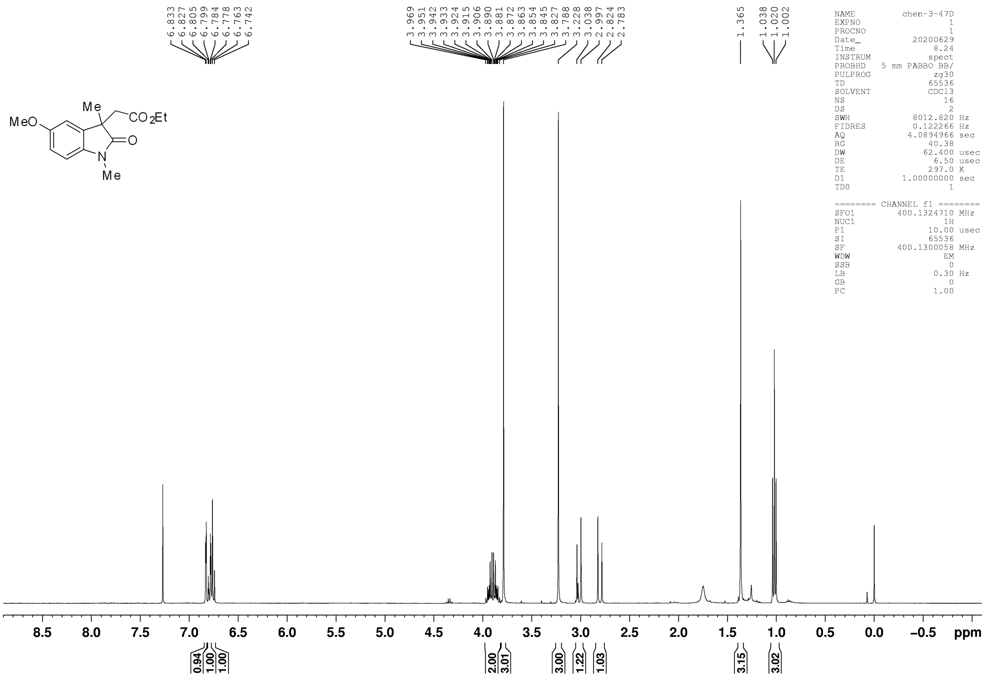

Supplementary Figure 141. ^{1}H NMR spectrum for compound **8c**

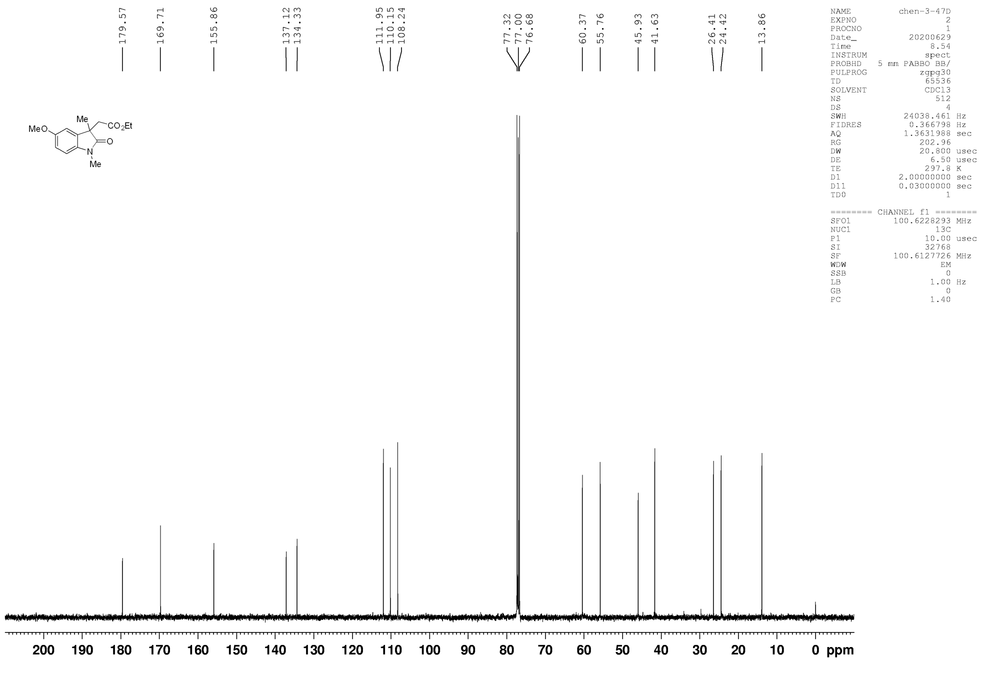

Supplementary Figure 142. ^{13}C NMR spectrum for compound **8c**

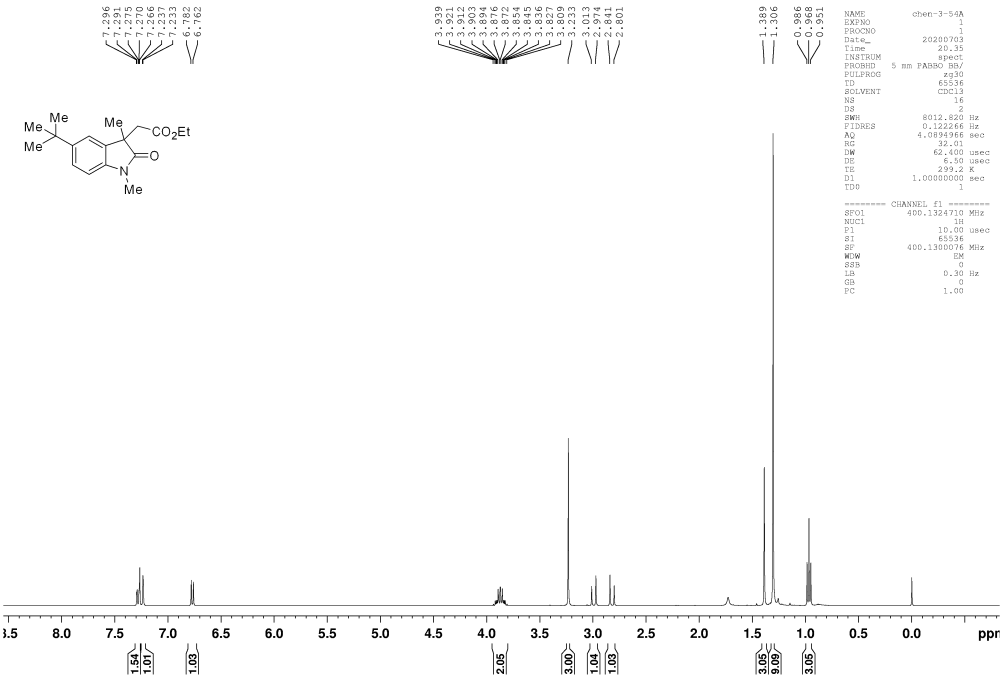

Supplementary Figure 143. ^{1}H NMR spectrum for compound **8d**

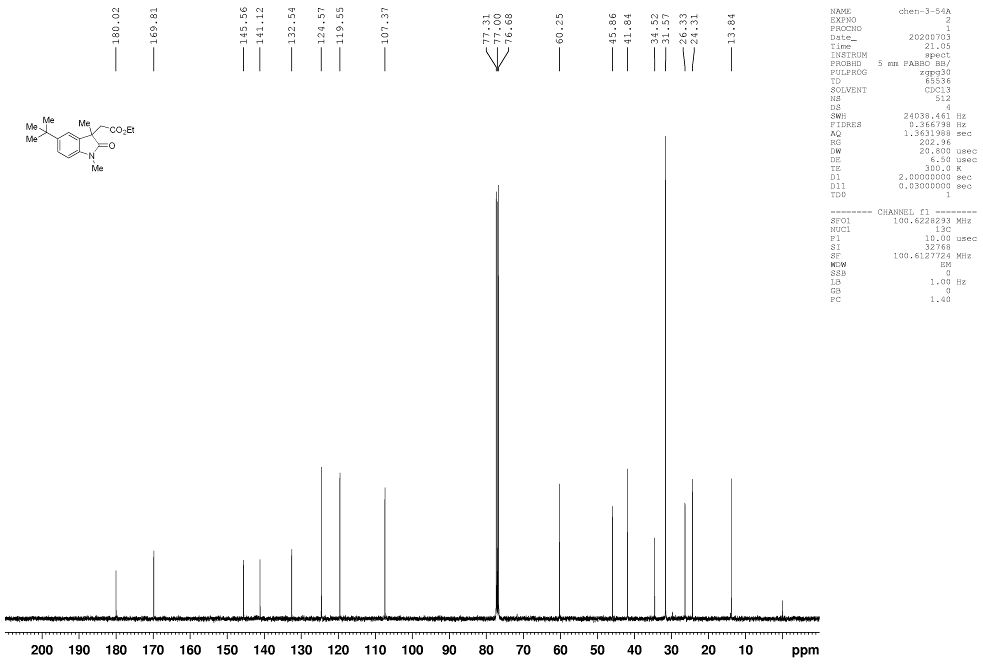

Supplementary Figure 144. ^{13}C NMR spectrum for compound **8d**

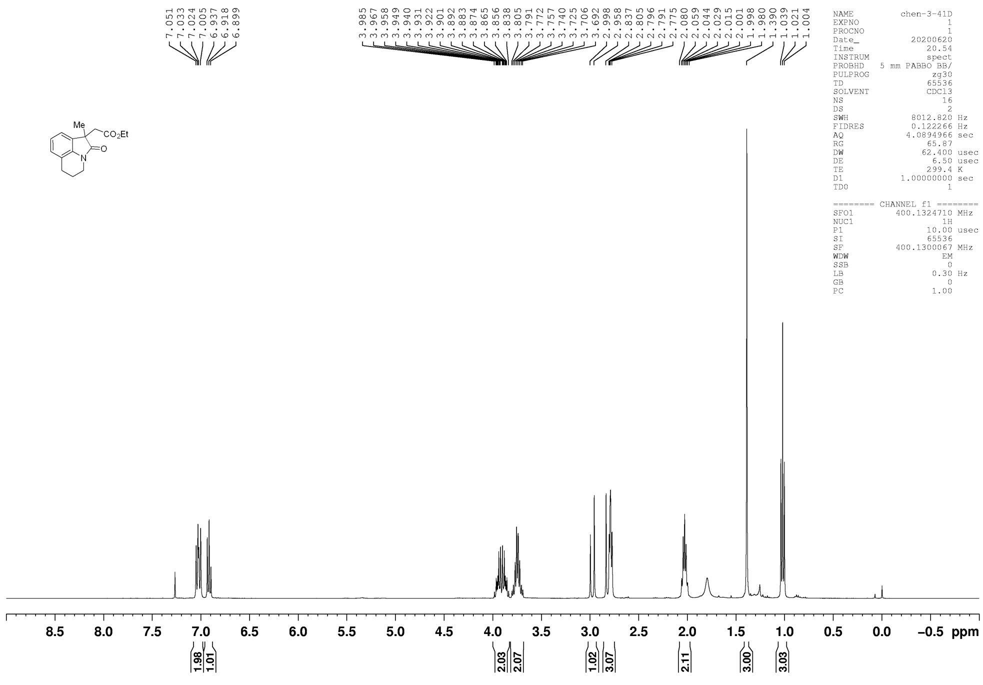

Supplementary Figure 145. ^{1}H NMR spectrum for compound **8e**

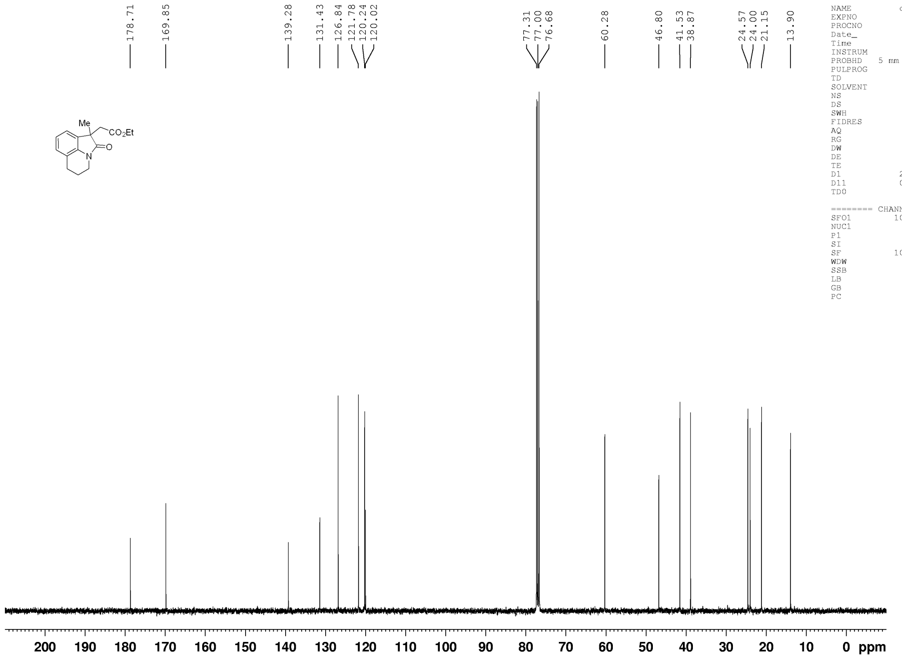

Supplementary Figure 146. ^{13}C NMR spectrum for compound **8e**

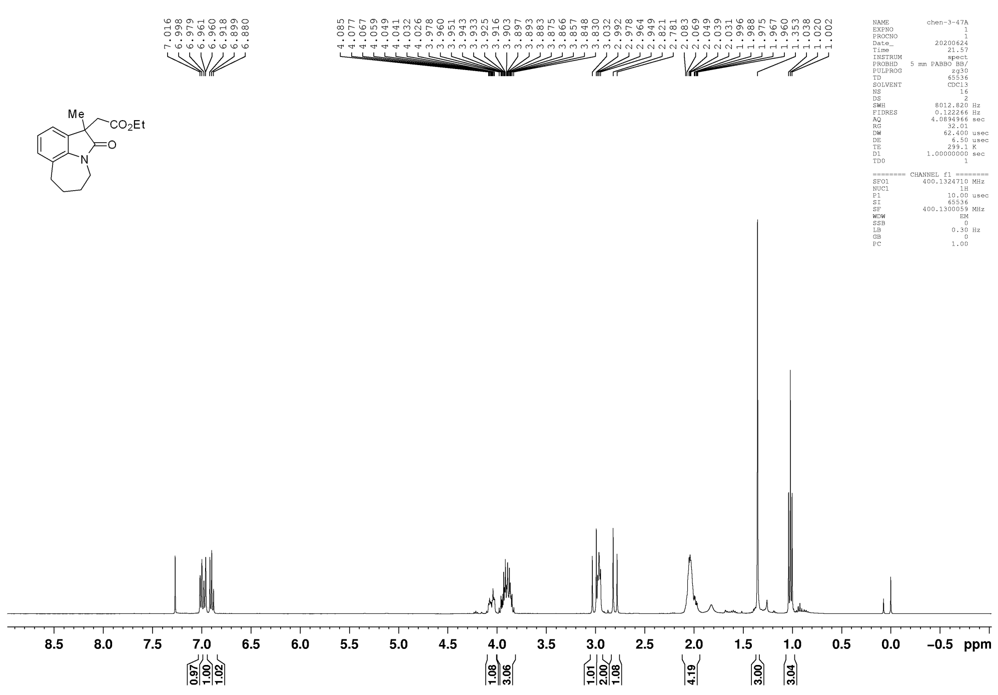

Supplementary Figure 147. ^{1}H NMR spectrum for compound **8f**

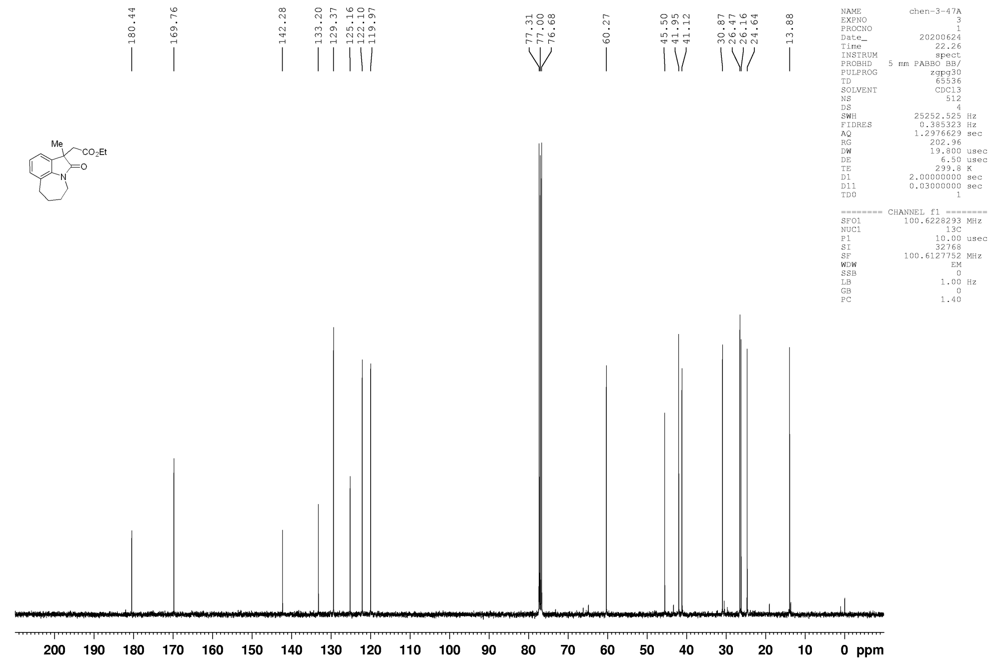

Supplementary Figure 148. ^{13}C NMR spectrum for compound **8f**

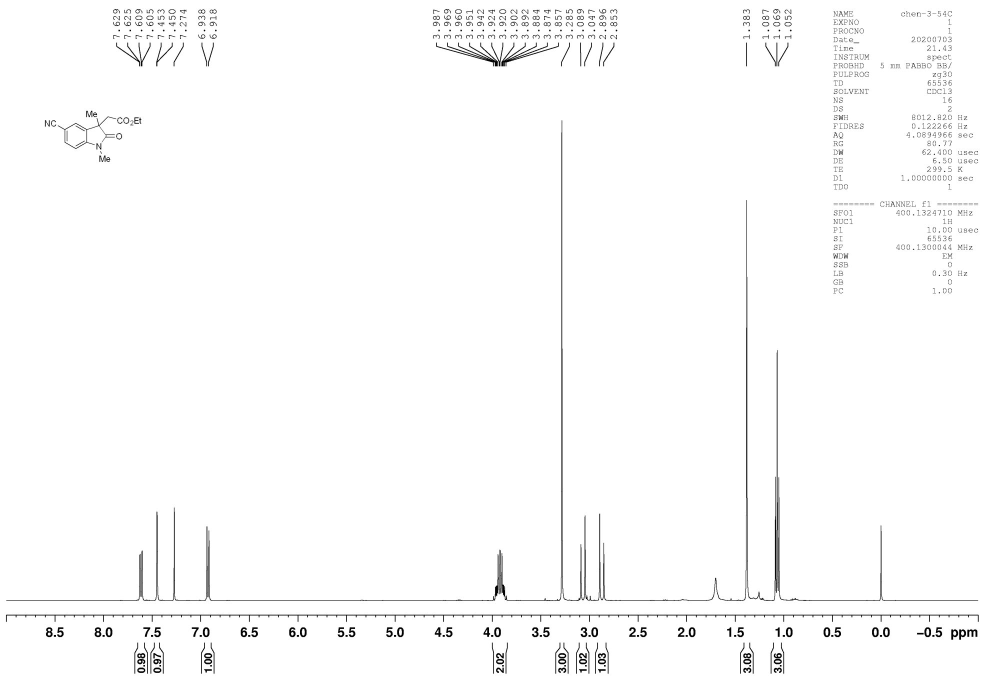

Supplementary Figure 149. ^{1}H NMR spectrum for compound **8g**

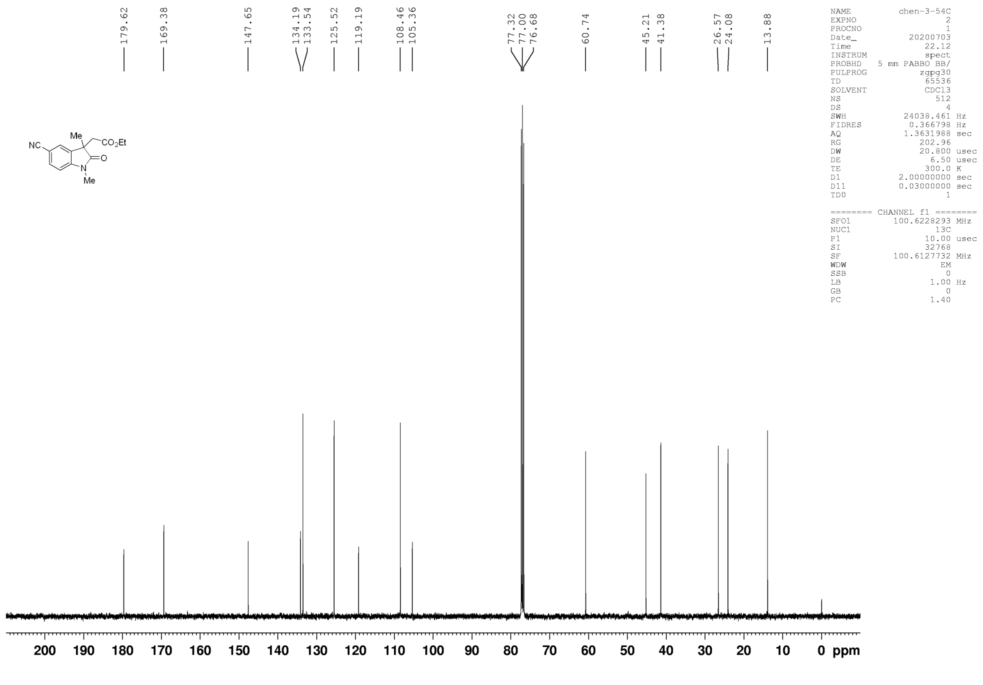

Supplementary Figure 150. ^{13}C NMR spectrum for compound **8g**

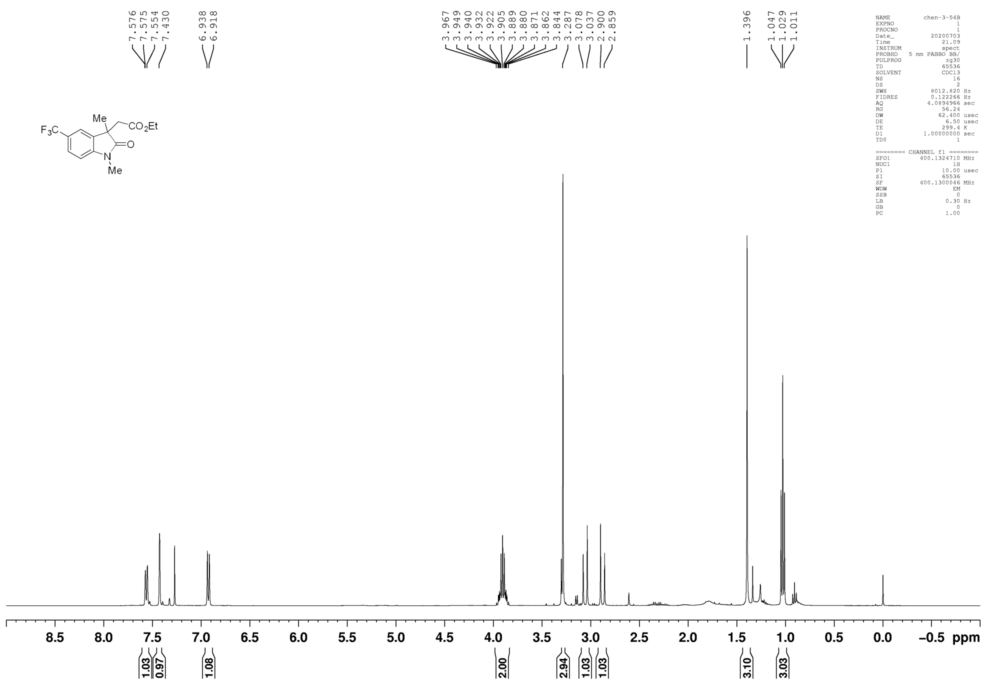

Supplementary Figure 151. ^{1}H NMR spectrum for compound **8h**

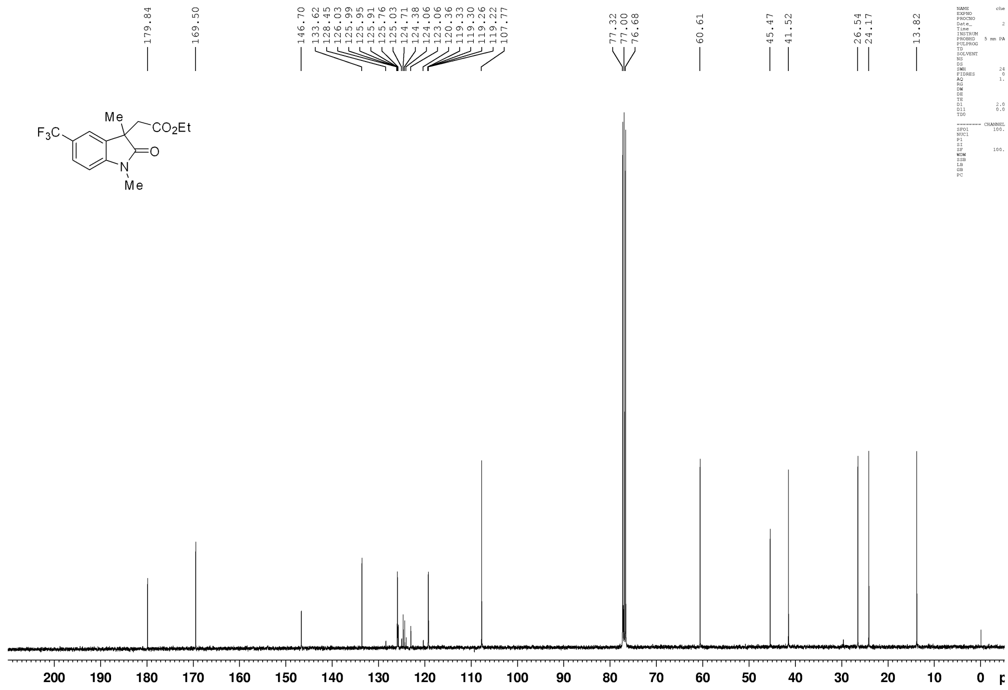

Supplementary Figure 152. ^{13}C NMR spectrum for compound **8h**

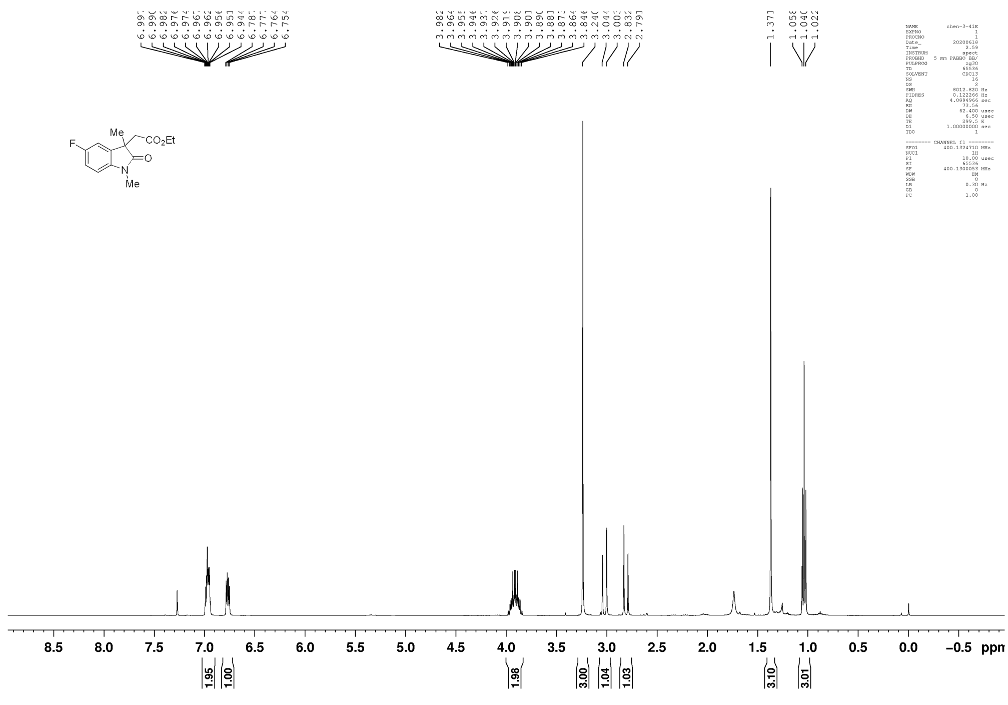

Supplementary Figure 153. ^{1}H NMR spectrum for compound **8i**

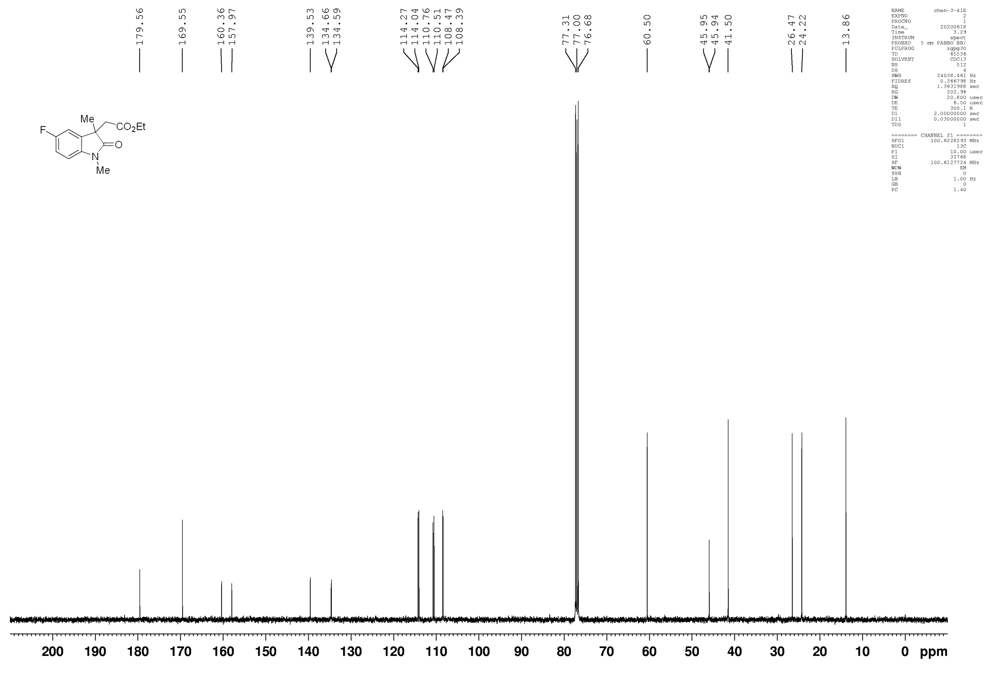

Supplementary Figure 154. ^{13}C NMR spectrum for compound **8i**

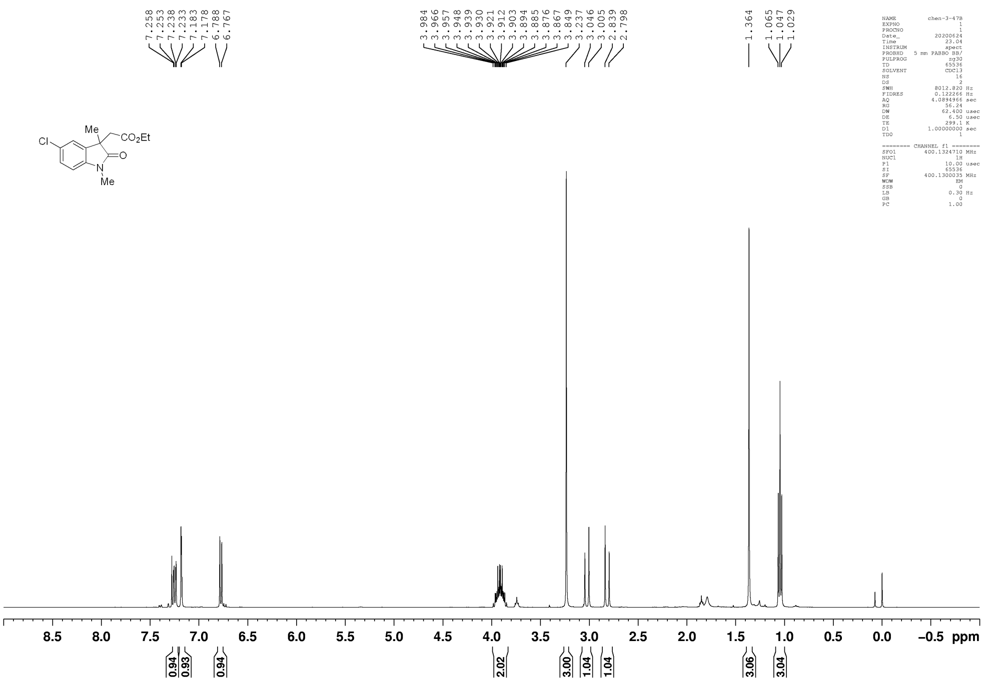

Supplementary Figure 155. ^{1}H NMR spectrum for compound **8j**

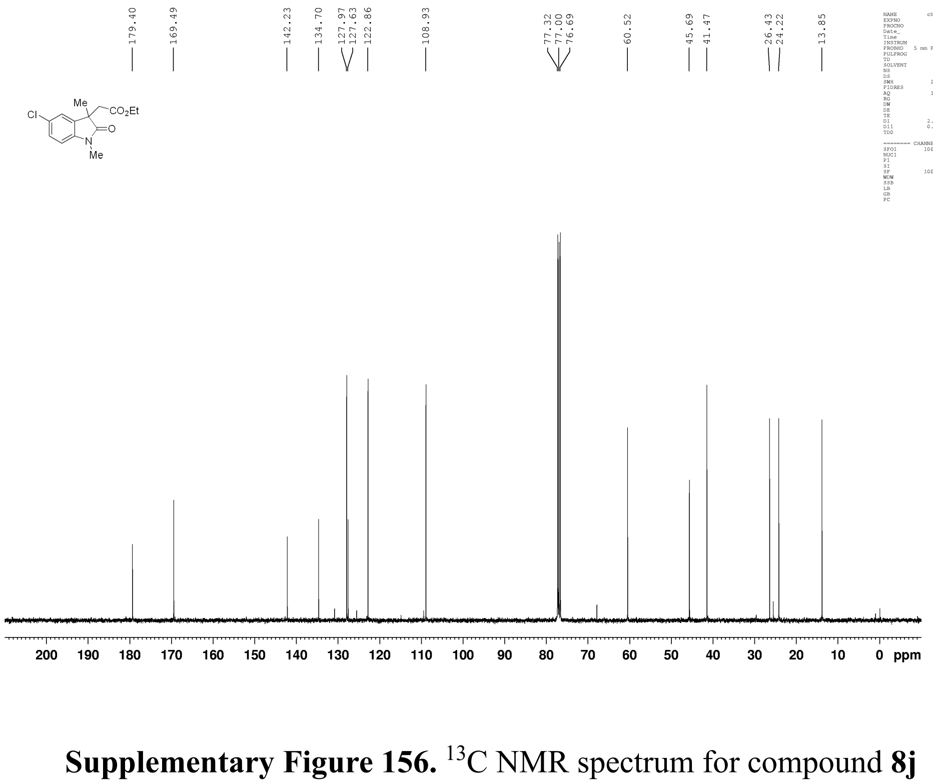

Supplementary Figure 156. ^{13}C NMR spectrum for compound **8j**

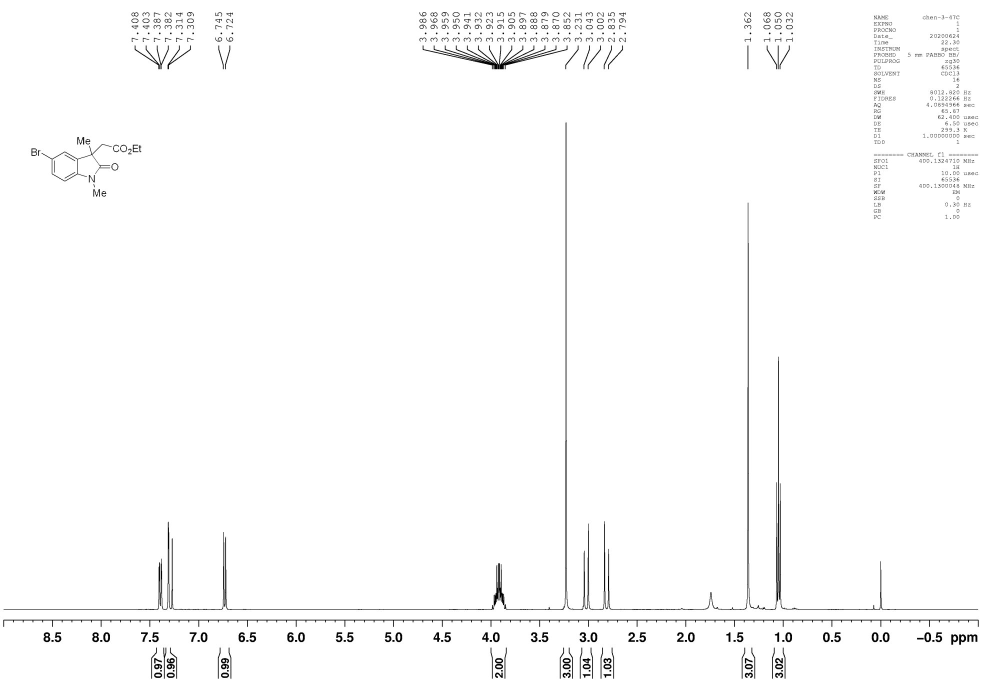

Supplementary Figure 157. ^{1}H NMR spectrum for compound **8k**

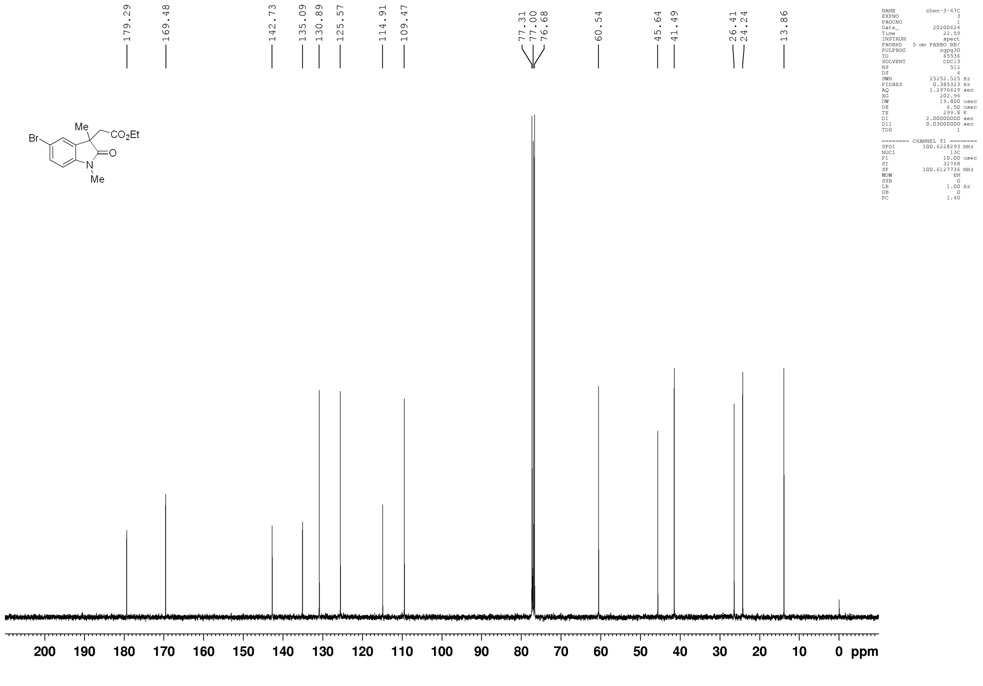

Supplementary Figure 158. ^{13}C NMR spectrum for compound **8k**

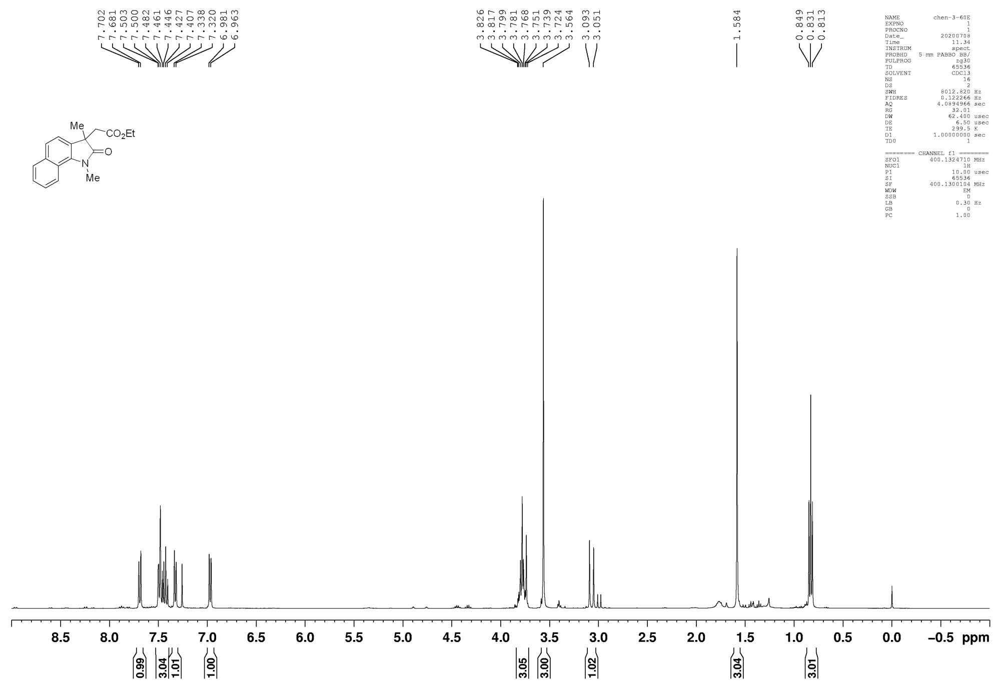

Supplementary Figure 159. ^{1}H NMR spectrum for compound **8l**

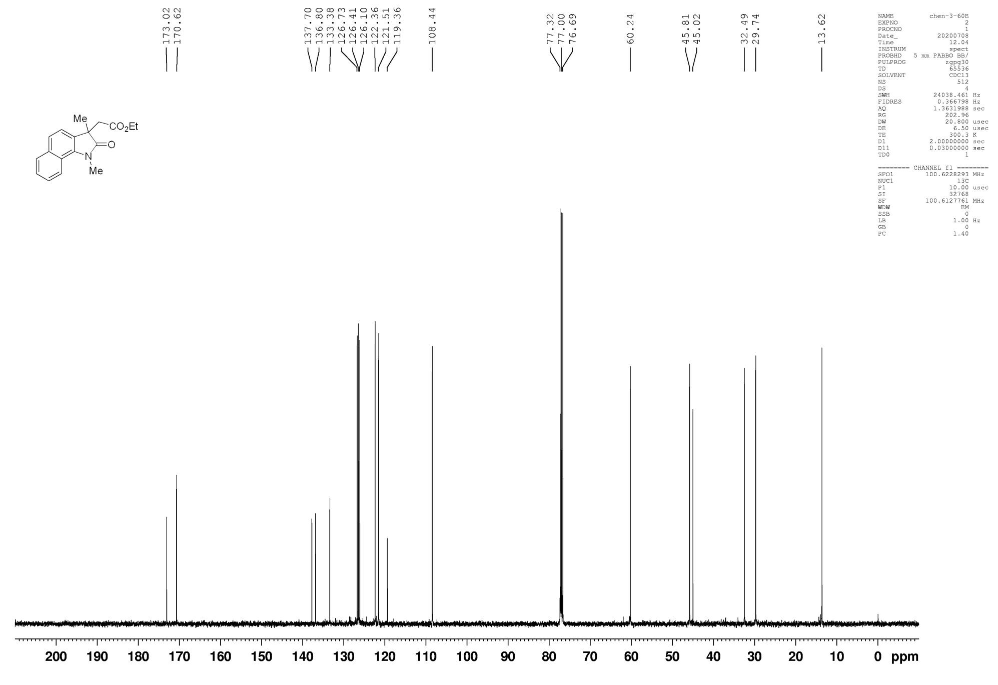

Supplementary Figure 160. ^{13}C NMR spectrum for compound **8l**

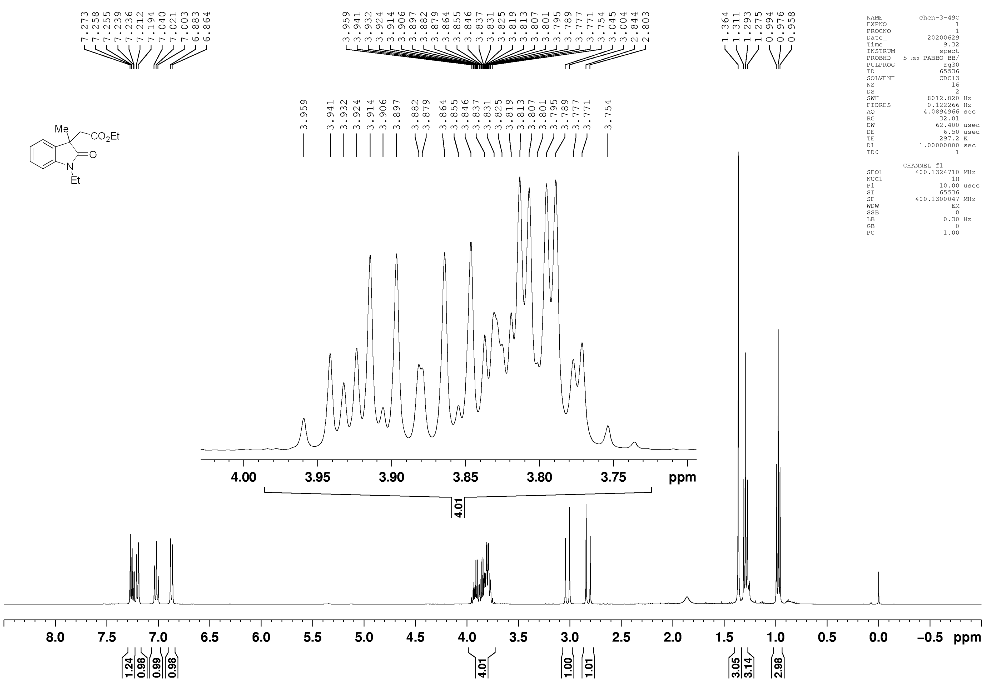

Supplementary Figure 161. ^{1}H NMR spectrum for compound **8m**

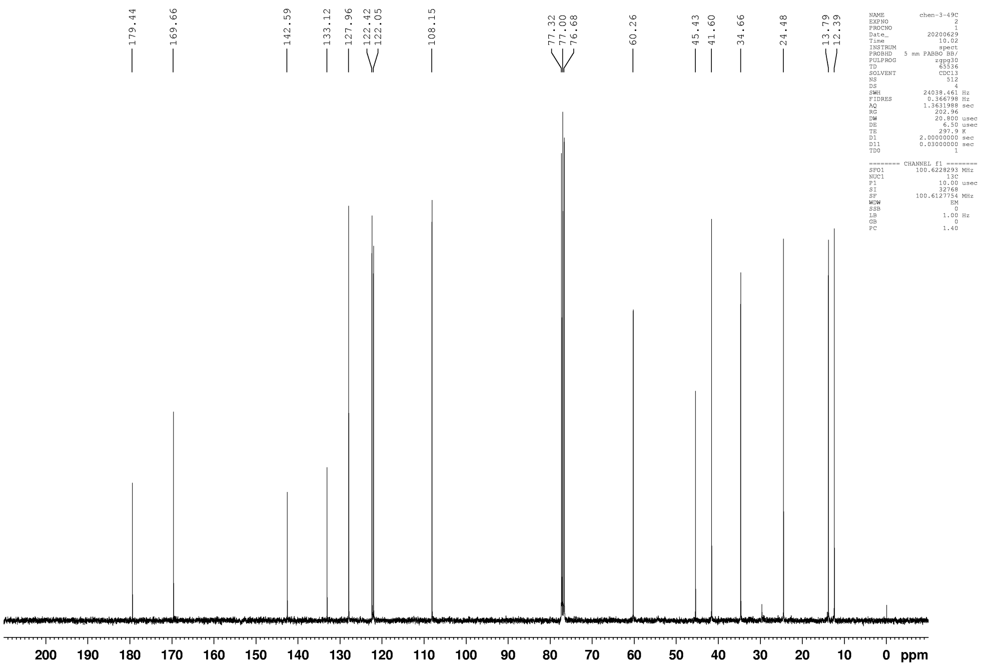

Supplementary Figure 162. ^{13}C NMR spectrum for compound **8m**

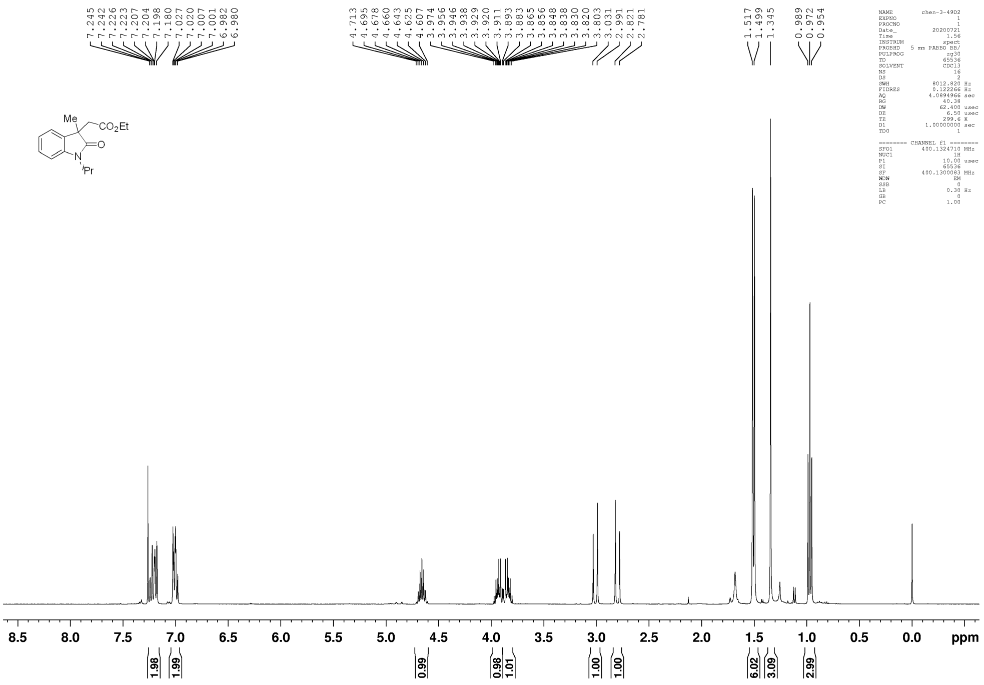

Supplementary Figure 163. ^{1}H NMR spectrum for compound **8n**

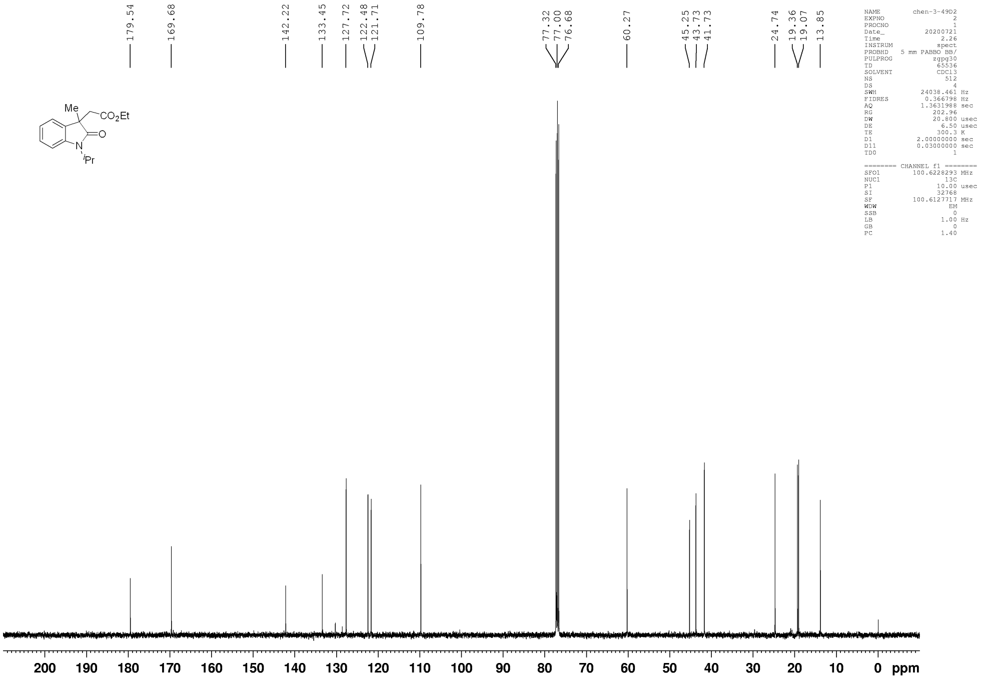

Supplementary Figure 164. ^{13}C NMR spectrum for compound **8n**

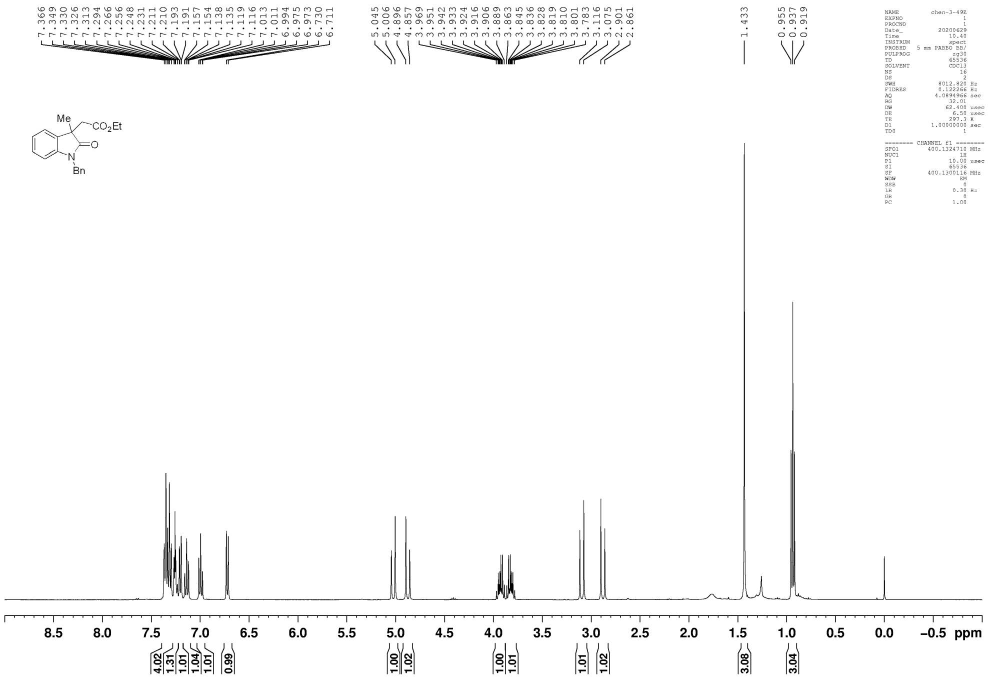

Supplementary Figure 165. ^{1}H NMR spectrum for compound **8o**

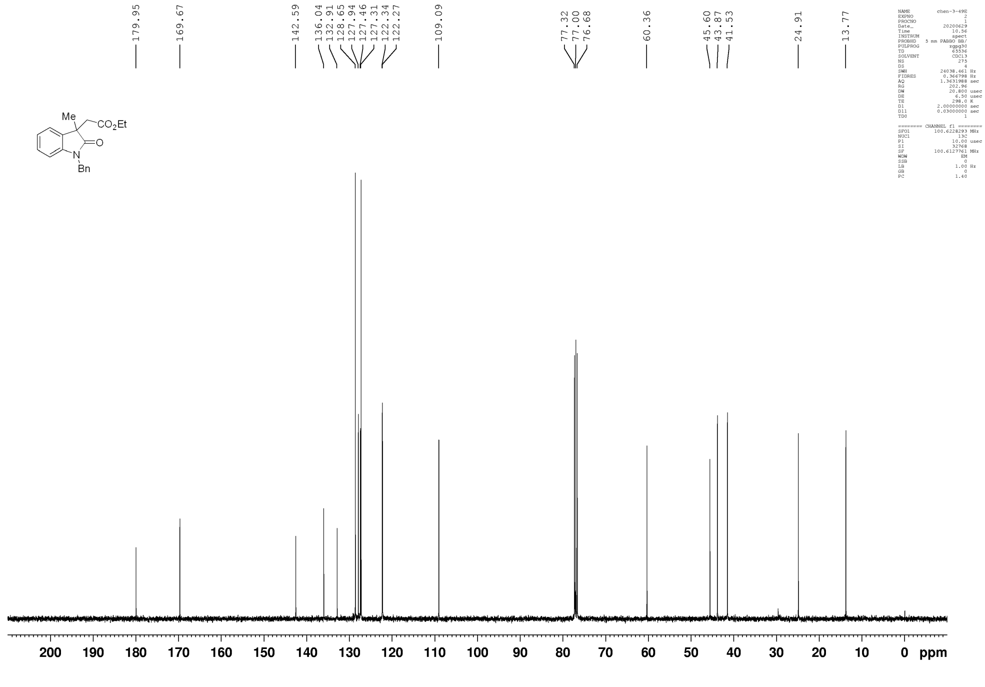

Supplementary Figure 166. ^{13}C NMR spectrum for compound **8o**

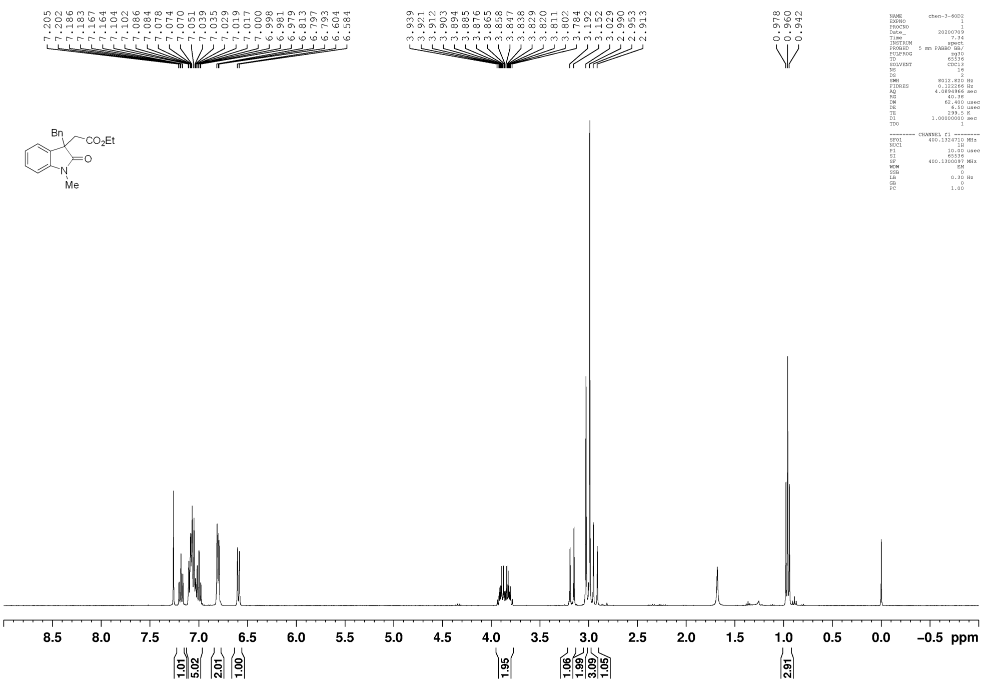

Supplementary Figure 167. ^{1}H NMR spectrum for compound **8p**

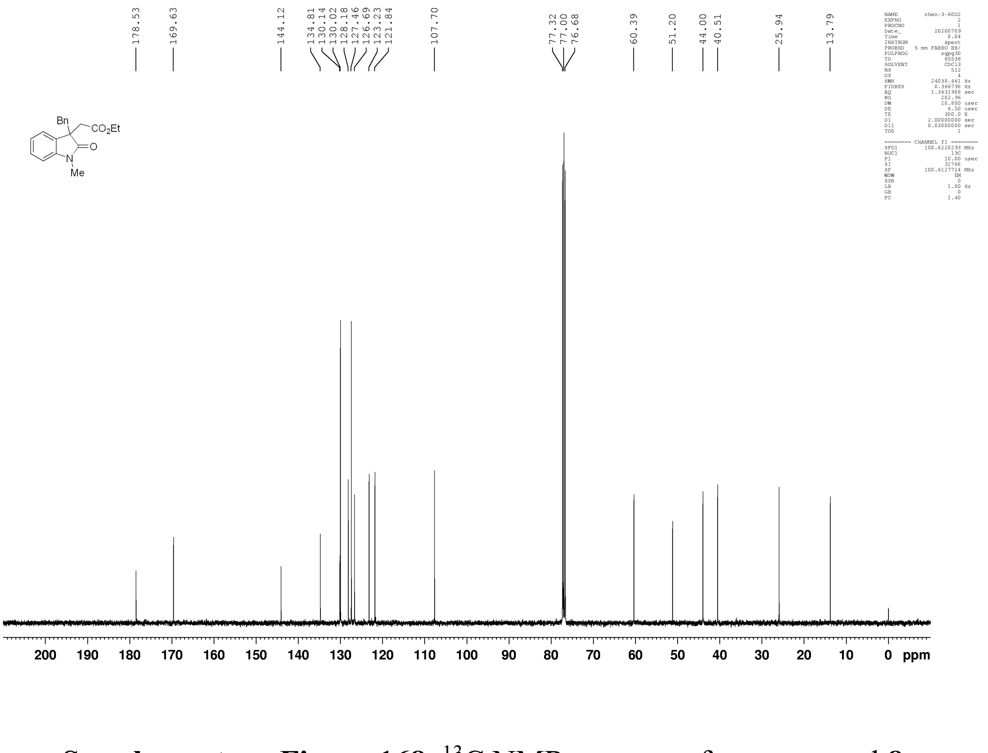

Supplementary Figure 168. ^{13}C NMR spectrum for compound **8p**

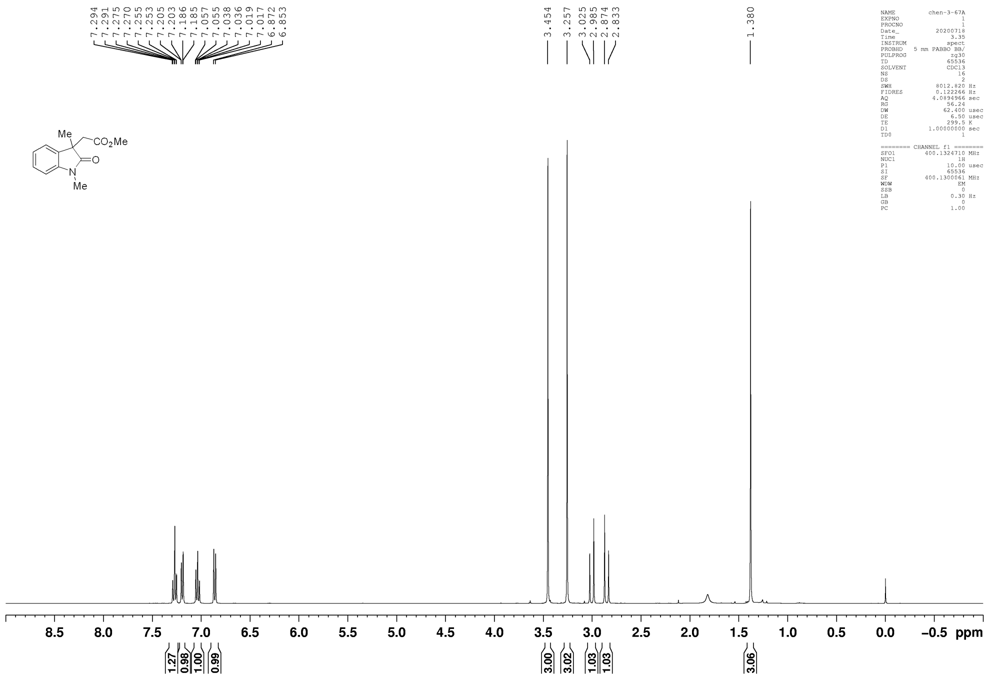

Supplementary Figure 169. ^{1}H NMR spectrum for compound **8q**

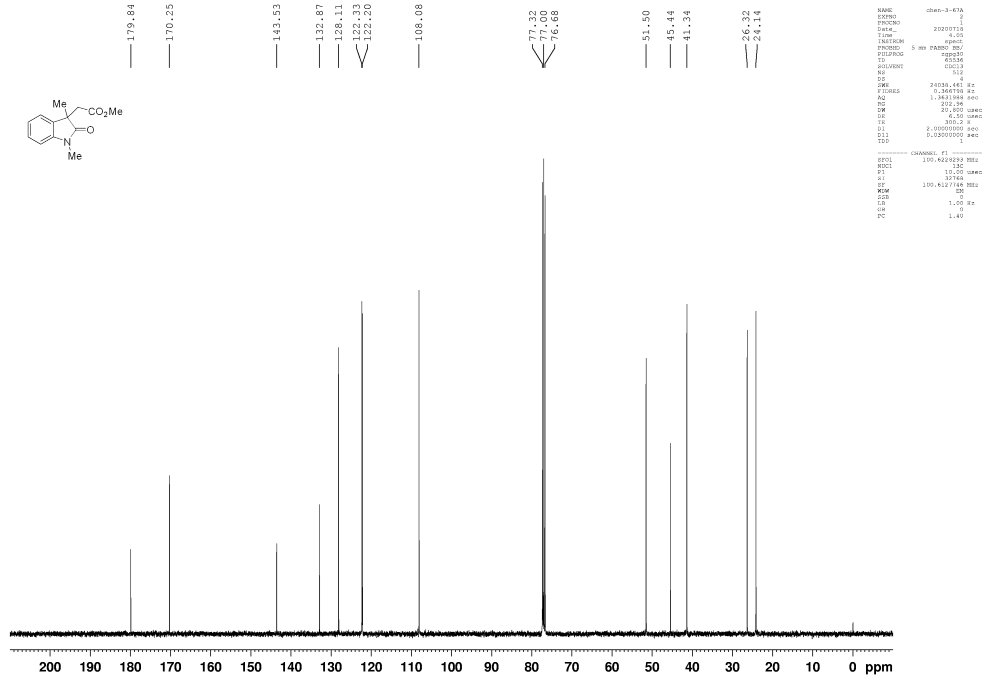

Supplementary Figure 170. ^{13}C NMR spectrum for compound **8q**

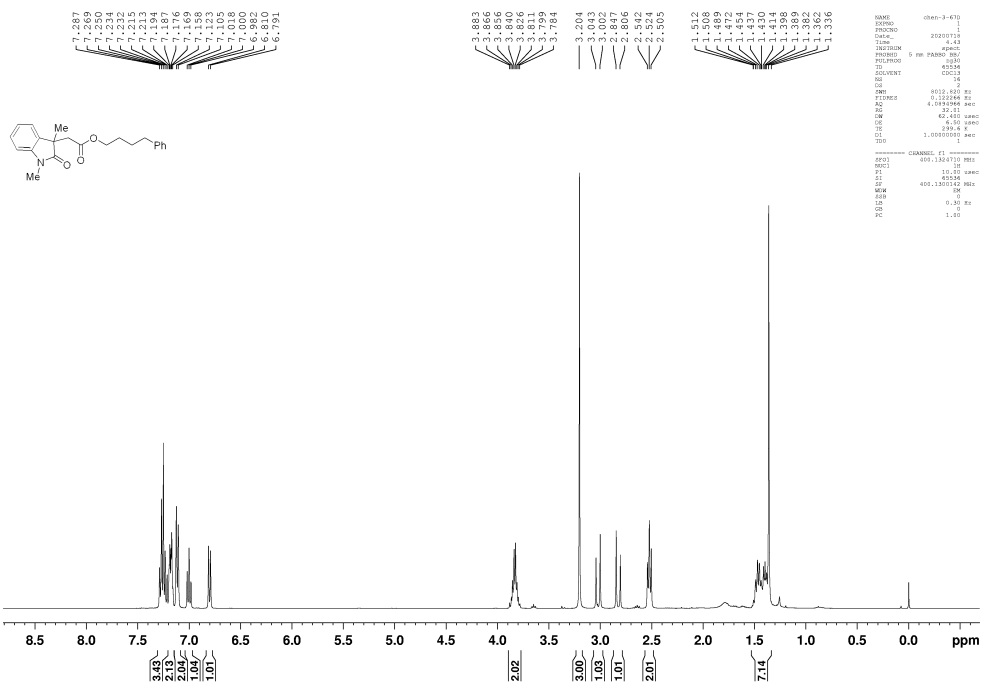

Supplementary Figure 171. ^{1}H NMR spectrum for compound **8r**

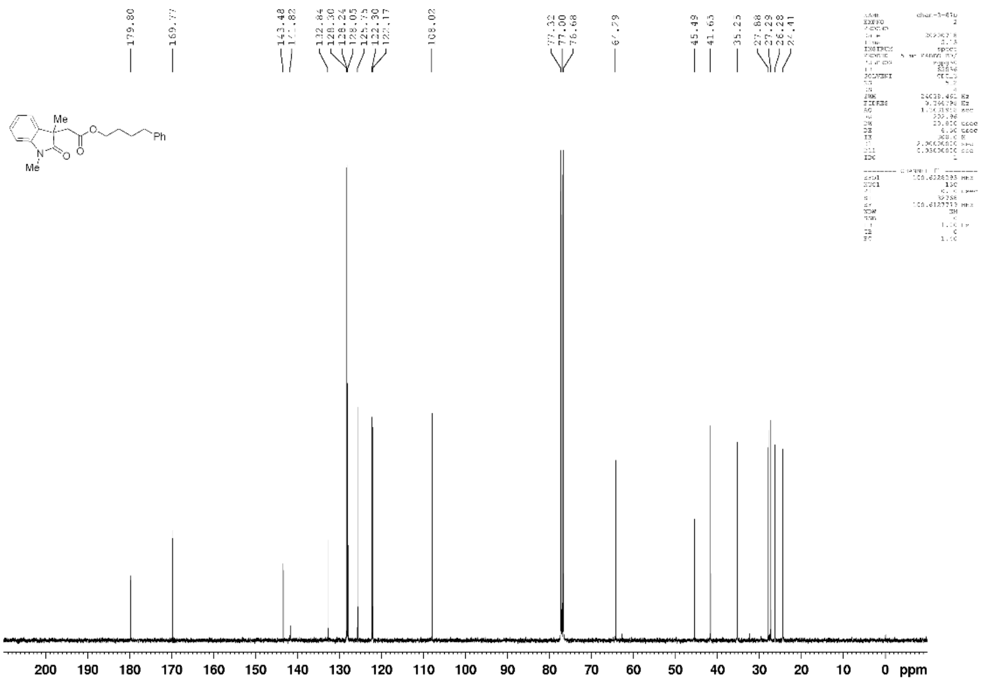

Supplementary Figure 172. ^{13}C NMR spectrum for compound **8r**

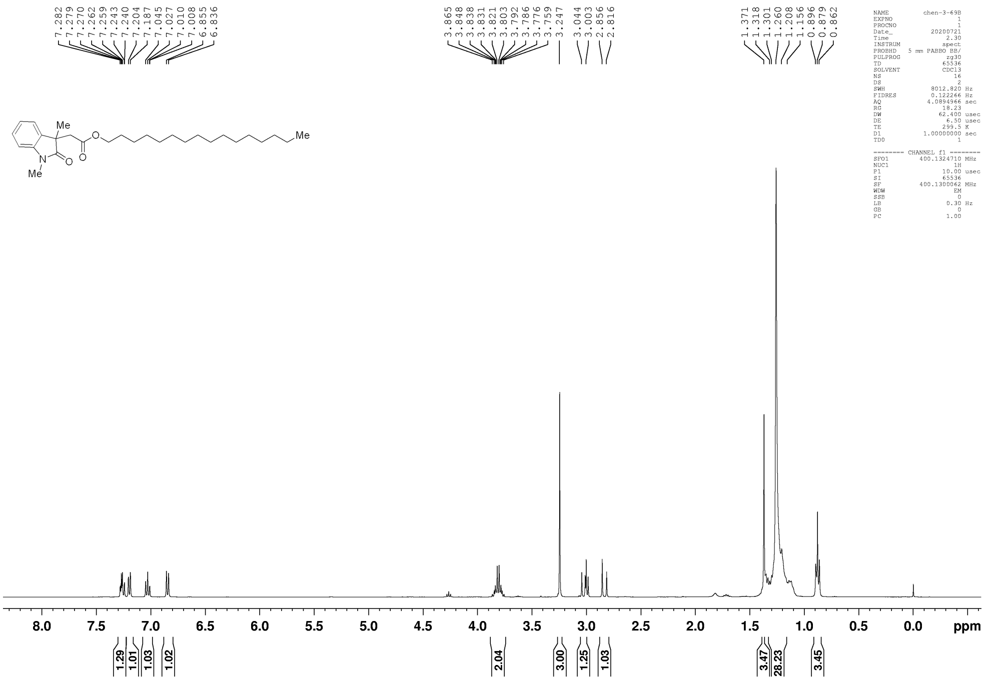

Supplementary Figure 173. ^{1}H NMR spectrum for compound **8s**

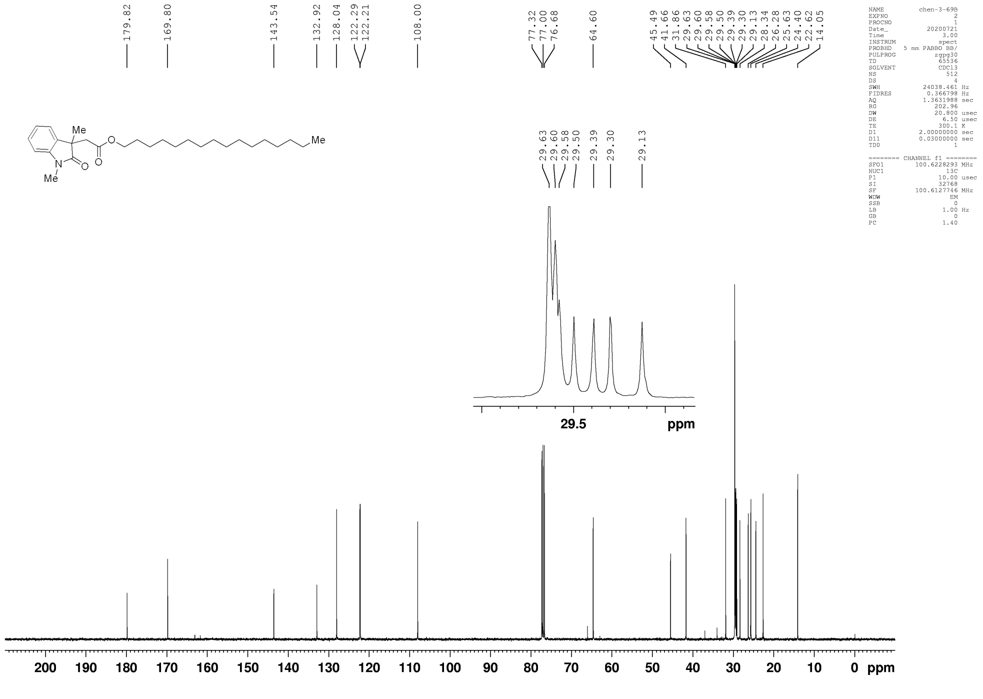

Supplementary Figure 174. ^{13}C NMR spectrum for compound **8s**

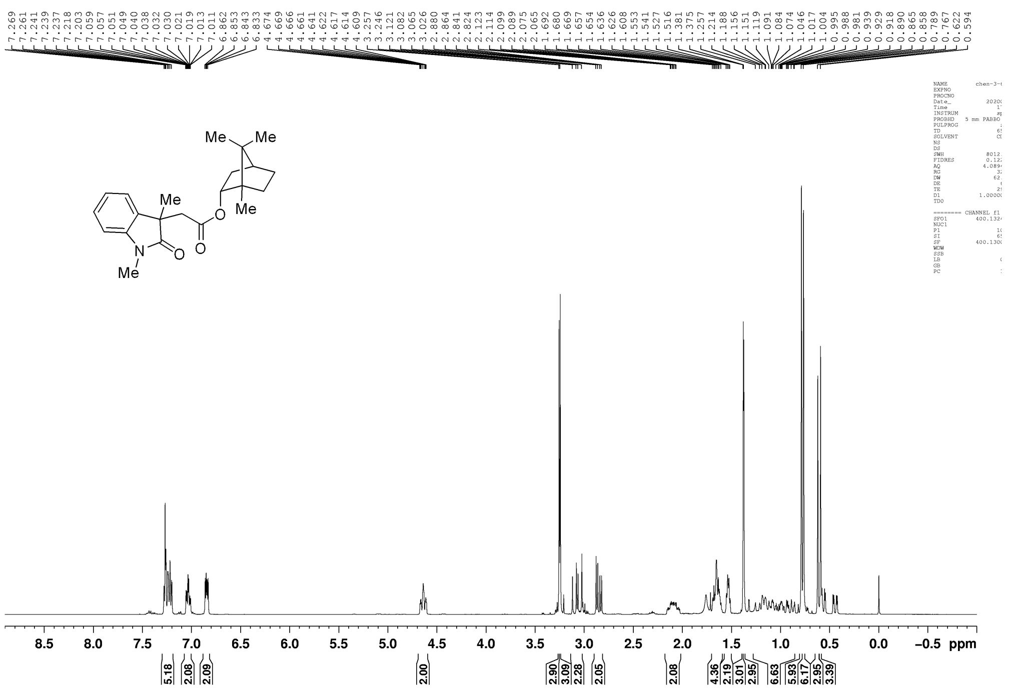

Supplementary Figure 175. ^{1}H NMR spectrum for compound **8t**

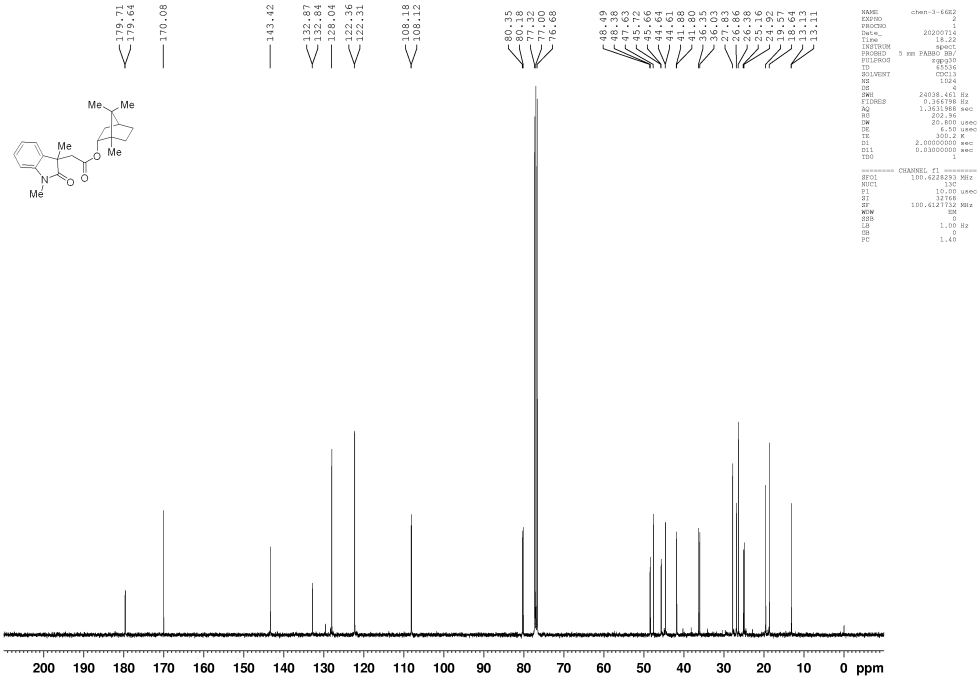

Supplementary Figure 176. ^{13}C NMR spectrum for compound **8t**

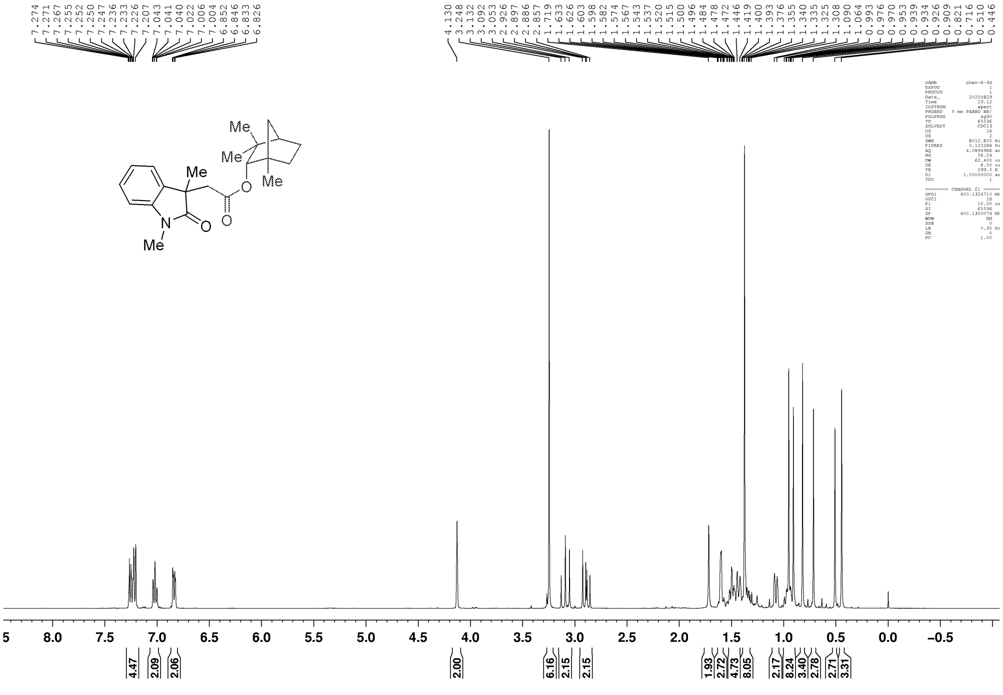

Supplementary Figure 177. ^{1}H NMR spectrum for compound **8u**

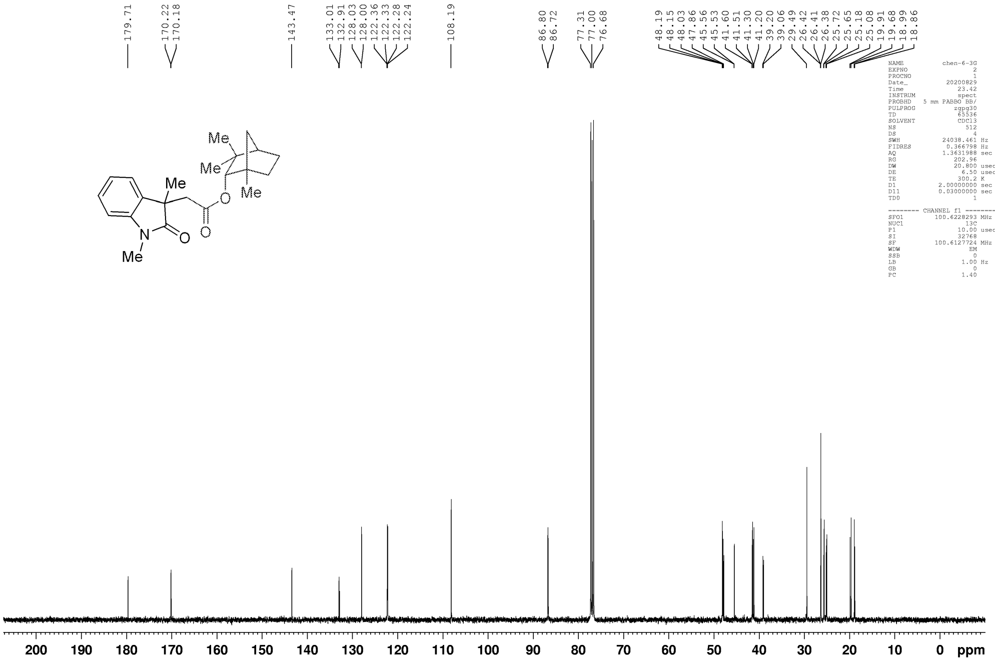

Supplementary Figure 178. ^{13}C NMR spectrum for compound **8u**

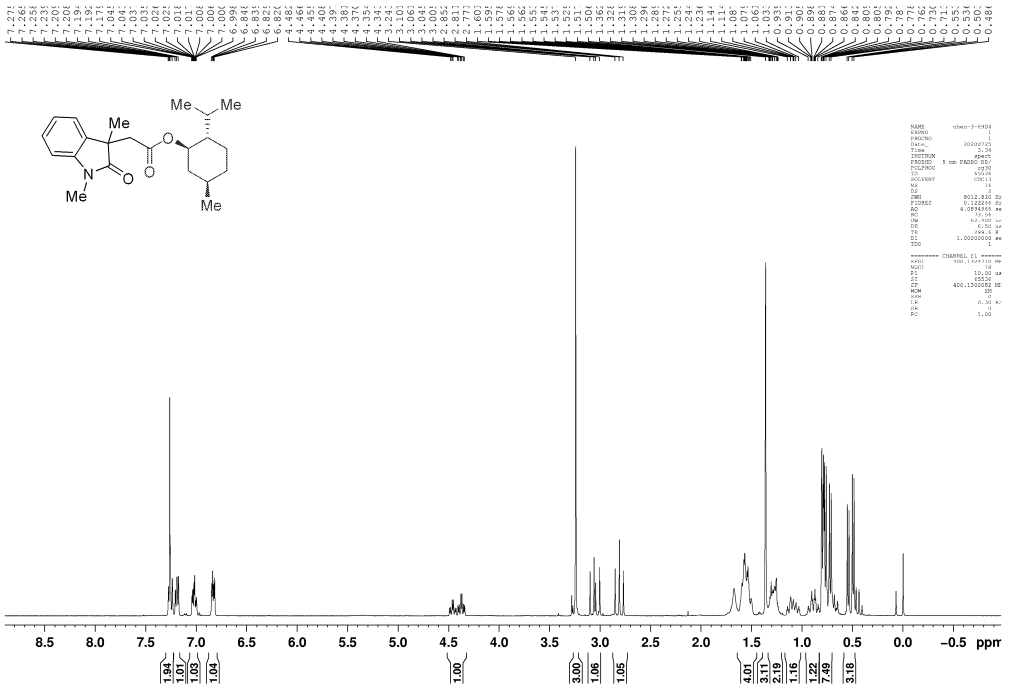

Supplementary Figure 179. ^{1}H NMR spectrum for compound **8v**

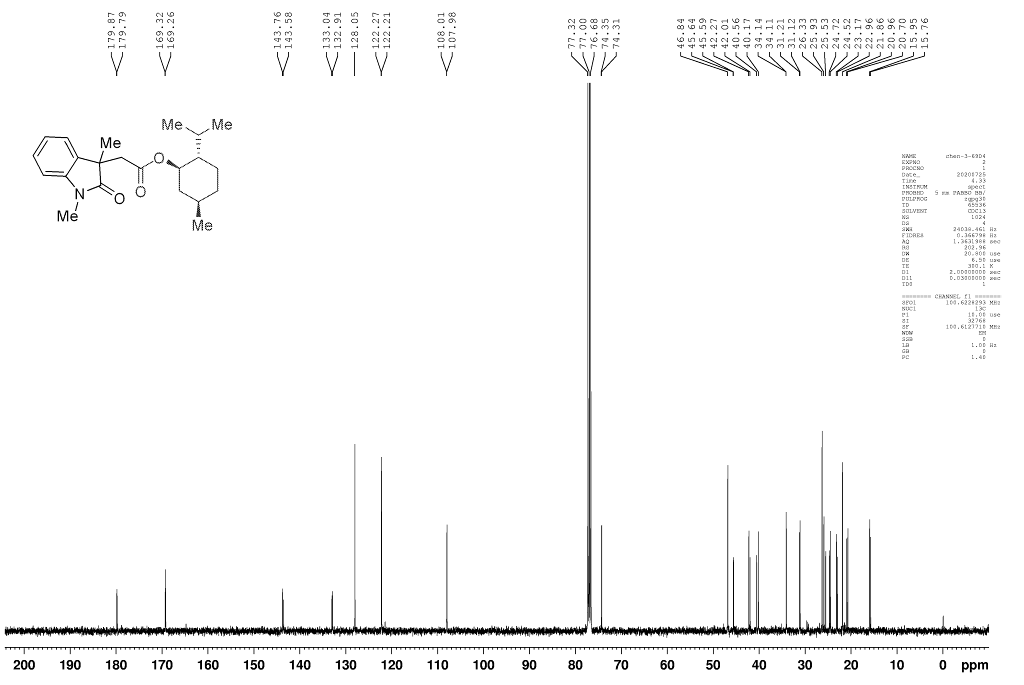

Supplementary Figure 180. ^{13}C NMR spectrum for compound **8v**

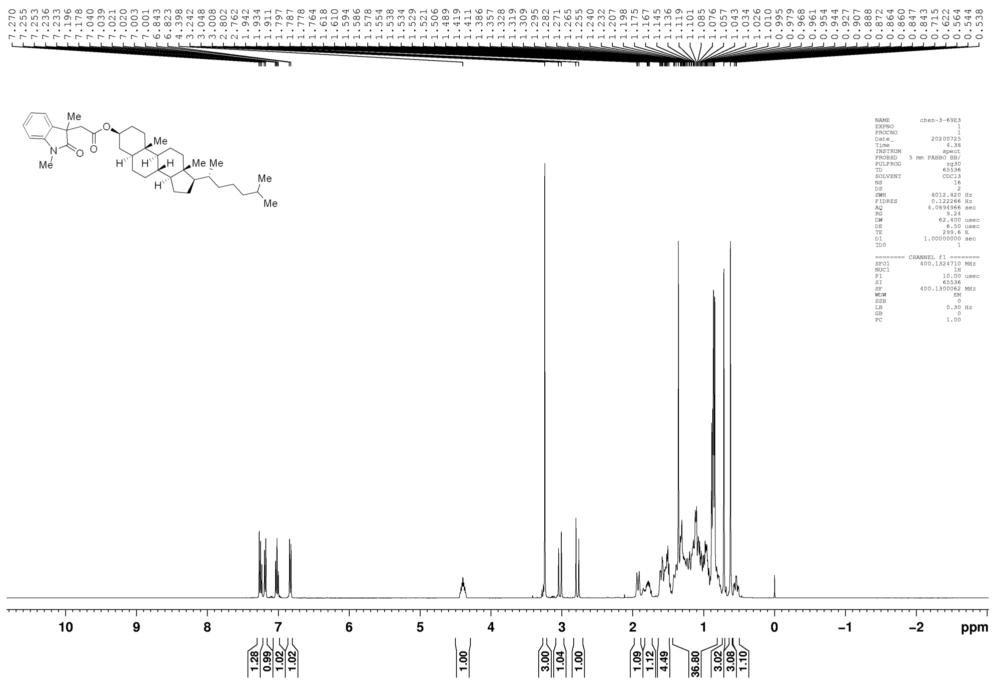

Supplementary Figure 181. ^{1}H NMR spectrum for compound **8w**

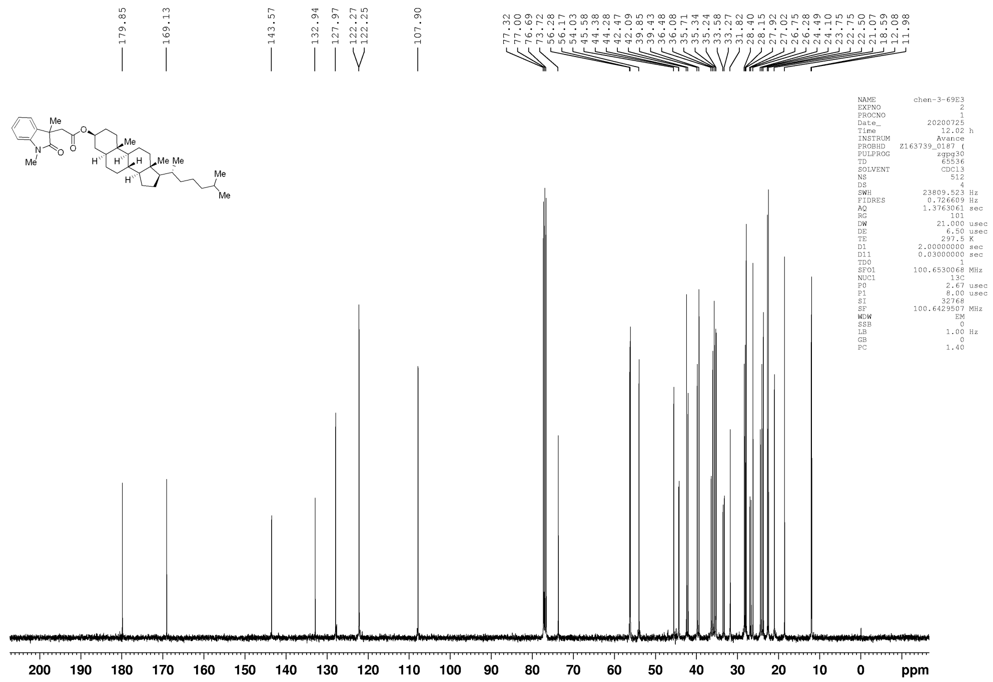

Supplementary Figure 182. ^{13}C NMR spectrum for compound **8w**

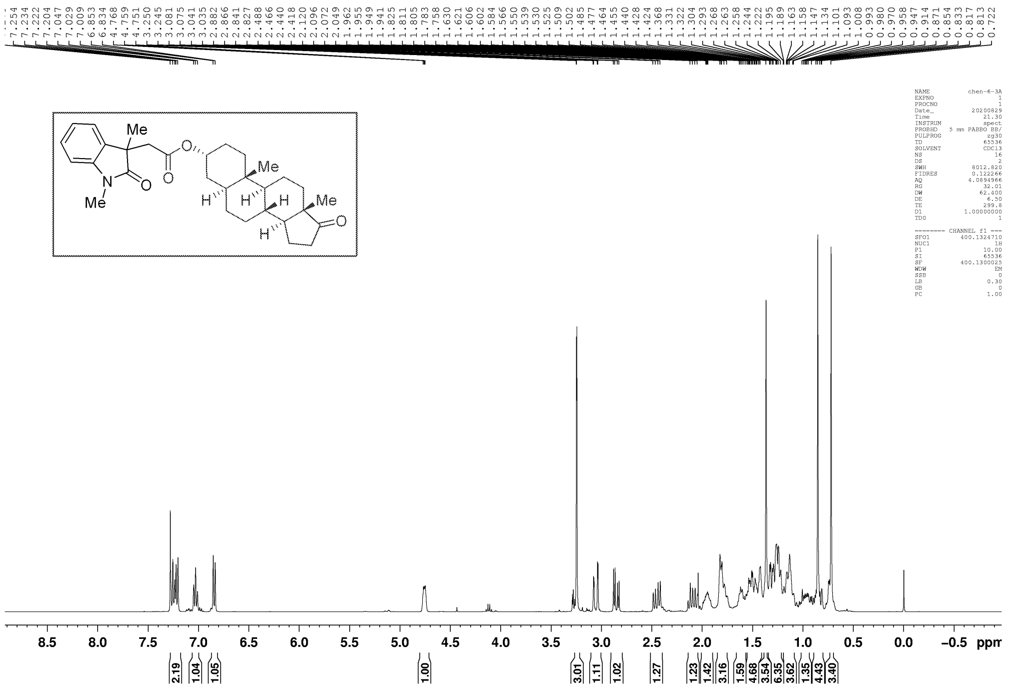

Supplementary Figure 183. ^{1}H NMR spectrum for compound **8x**

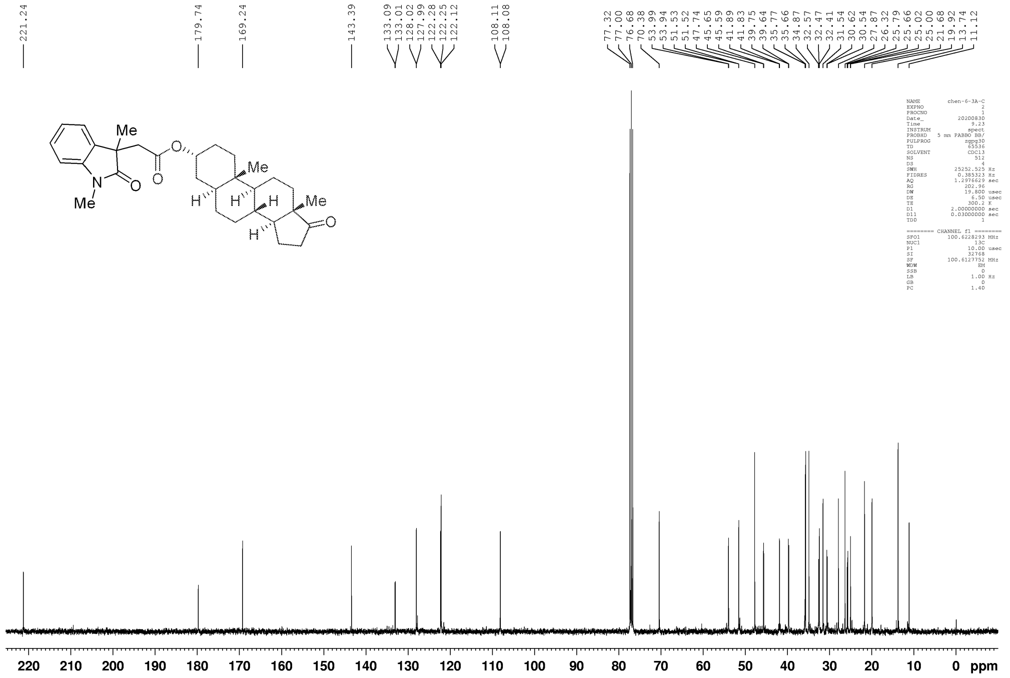

Supplementary Figure 184. ^{13}C NMR spectrum for compound **8x**

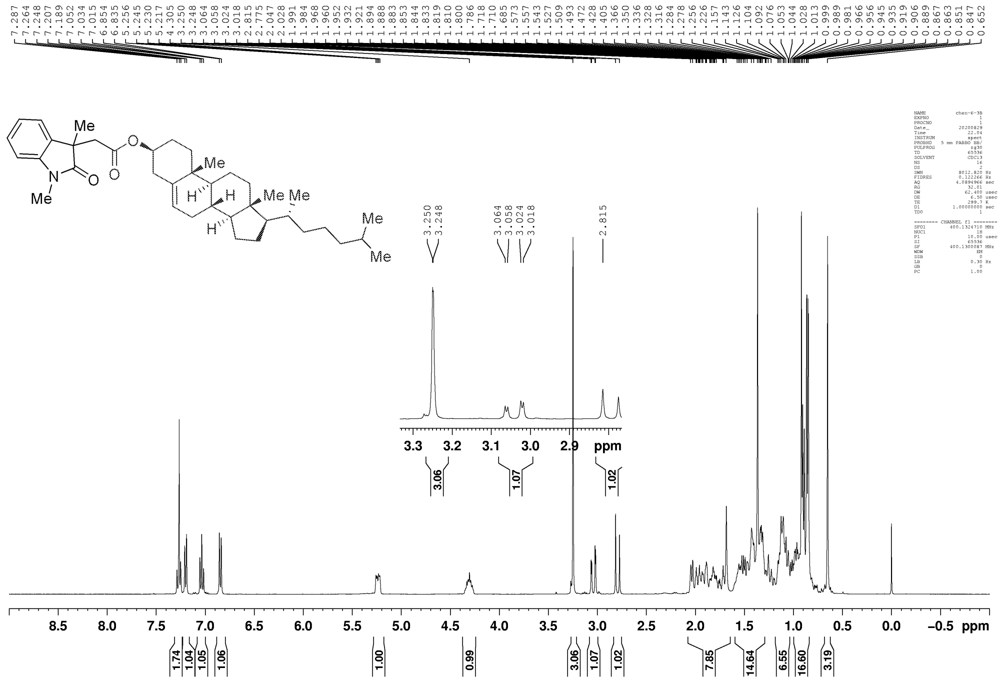

Supplementary Figure 185. ^{1}H NMR spectrum for compound **8y**

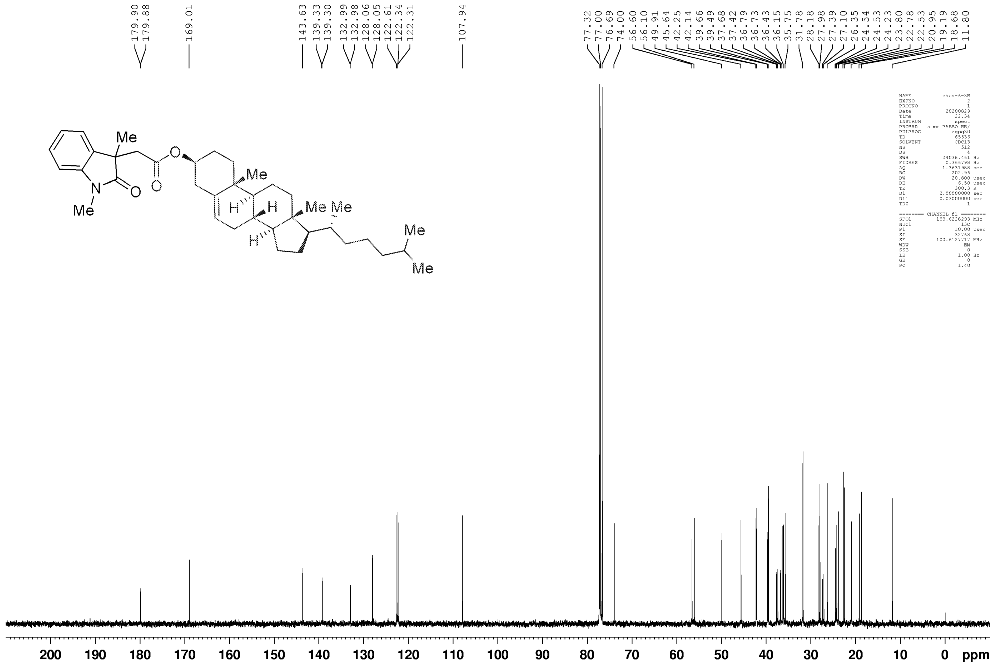

Supplementary Figure 186. ^{13}C NMR spectrum for compound **8y**

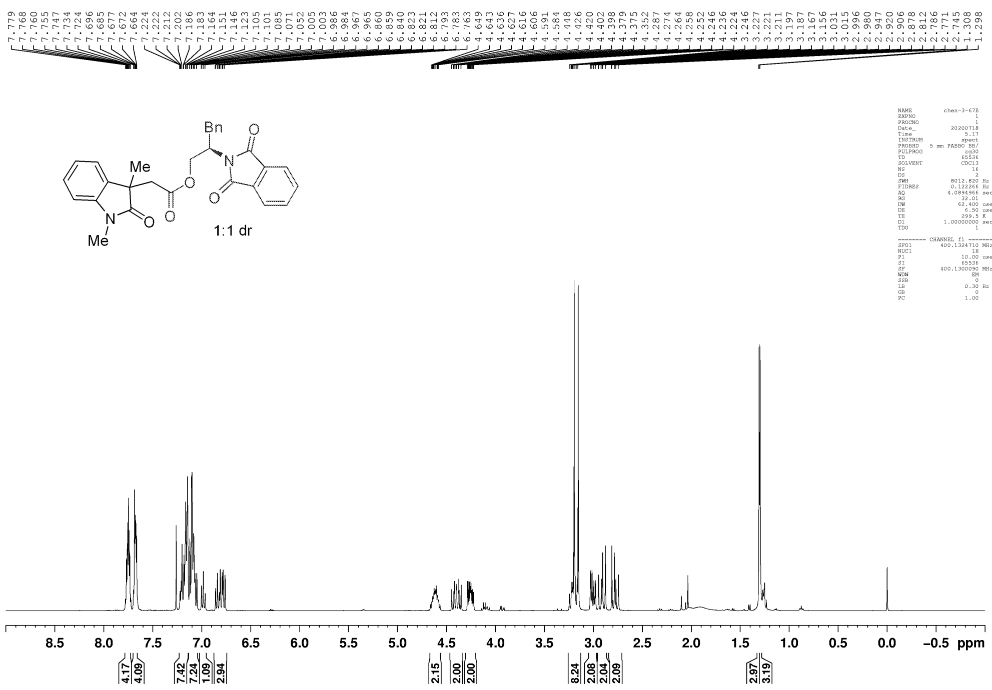

Supplementary Figure 187. ^{1}H NMR spectrum for compound **8z**

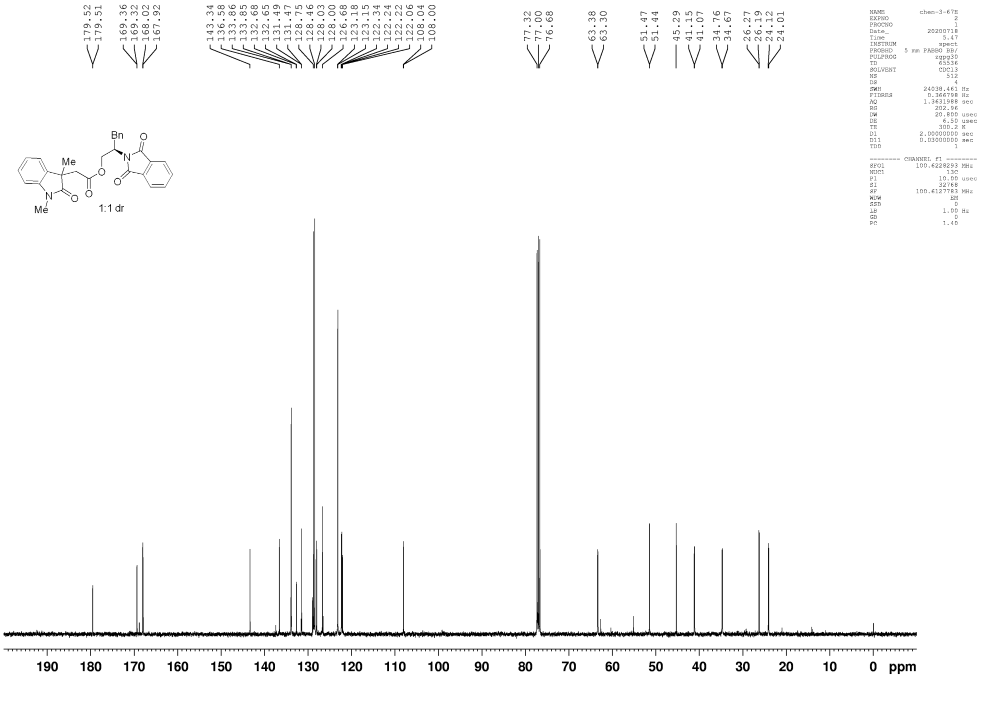

Supplementary Figure 188. ^{13}C NMR spectrum for compound **8z**

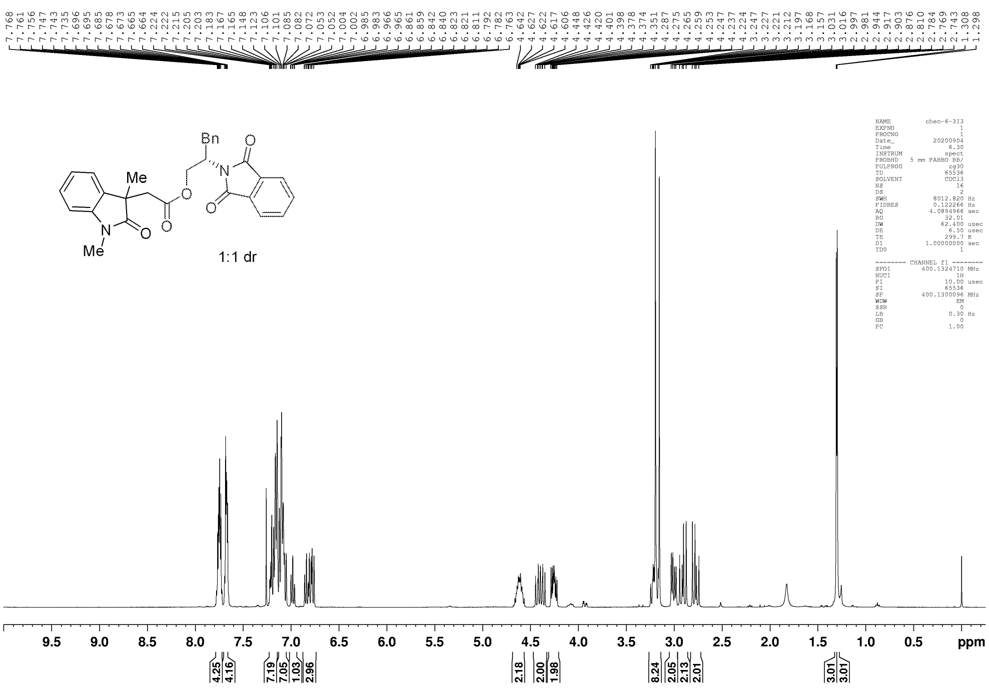

Supplementary Figure 189. ^{1}H NMR spectrum for compound **8aa**

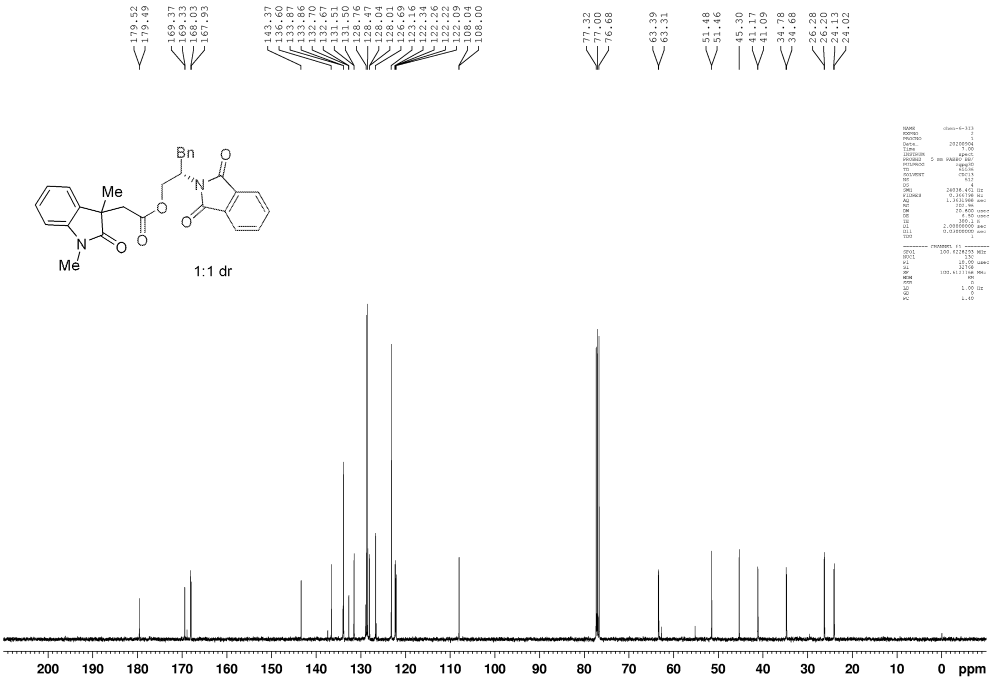

Supplementary Figure 190. ^{13}C NMR spectrum for compound **8aa**

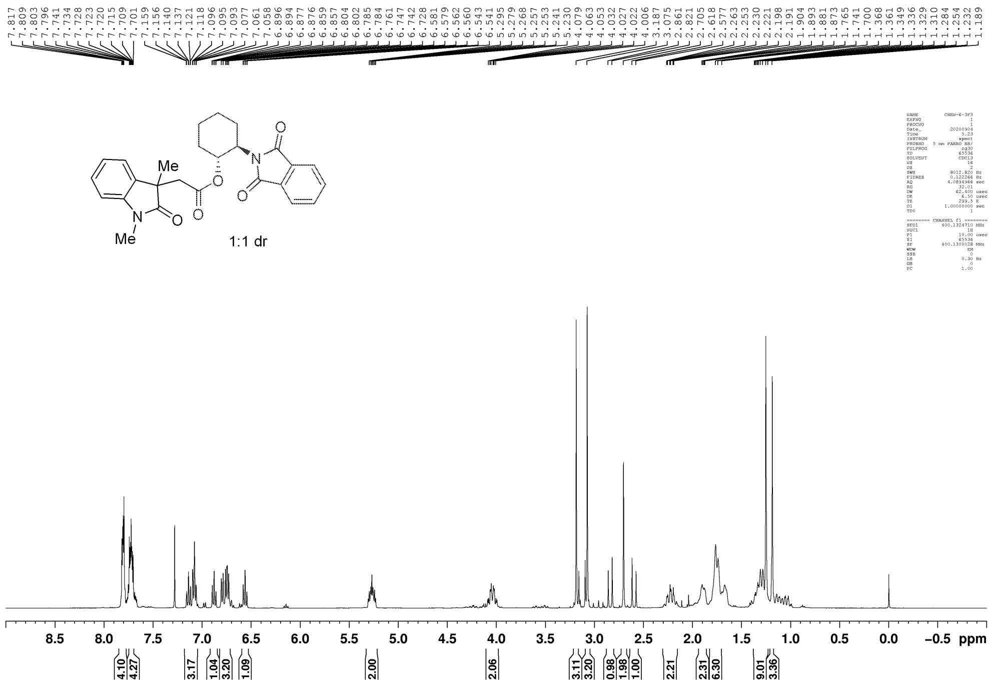

Supplementary Figure 191. ^{1}H NMR spectrum for compound **8ab**

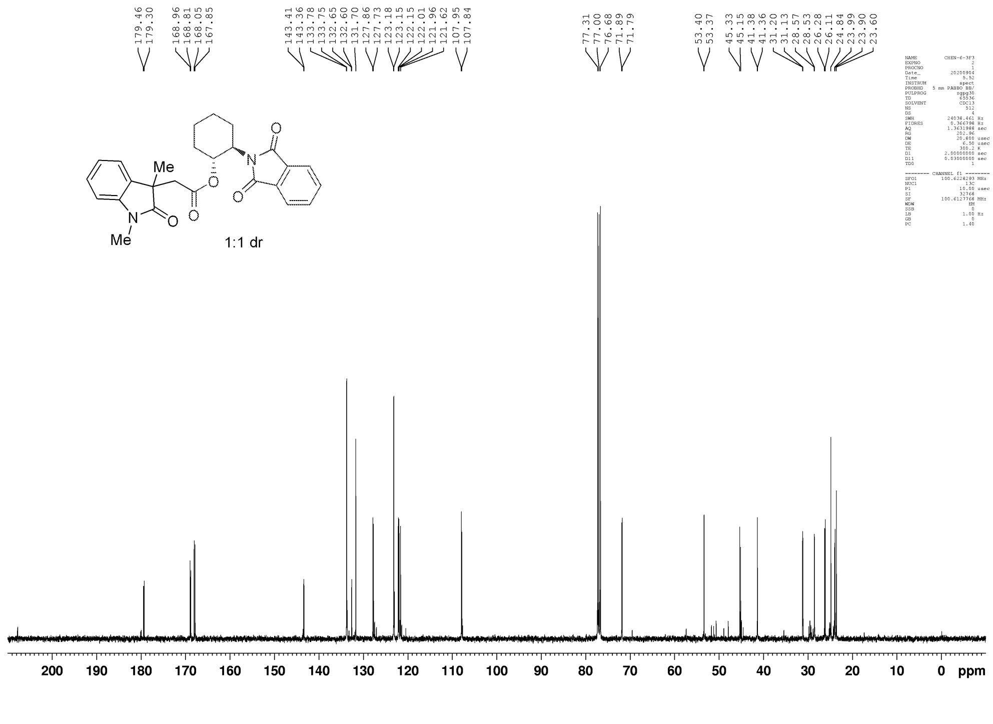

Supplementary Figure 192. ^{13}C NMR spectrum for compound **8ab**

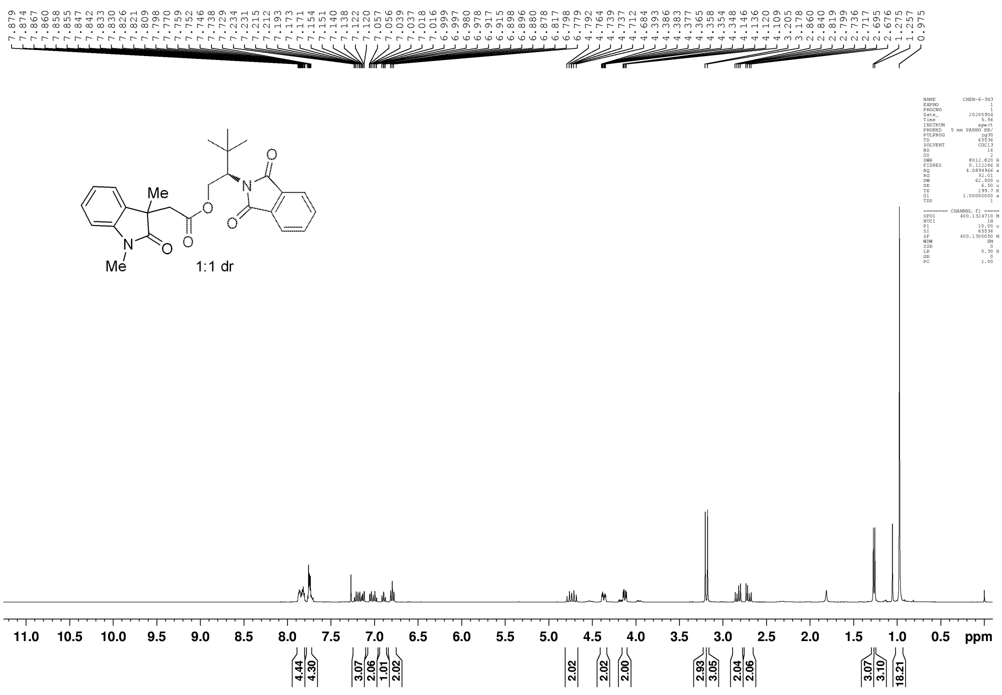

Supplementary Figure 193. ^{1}H NMR spectrum for compound **8ac**

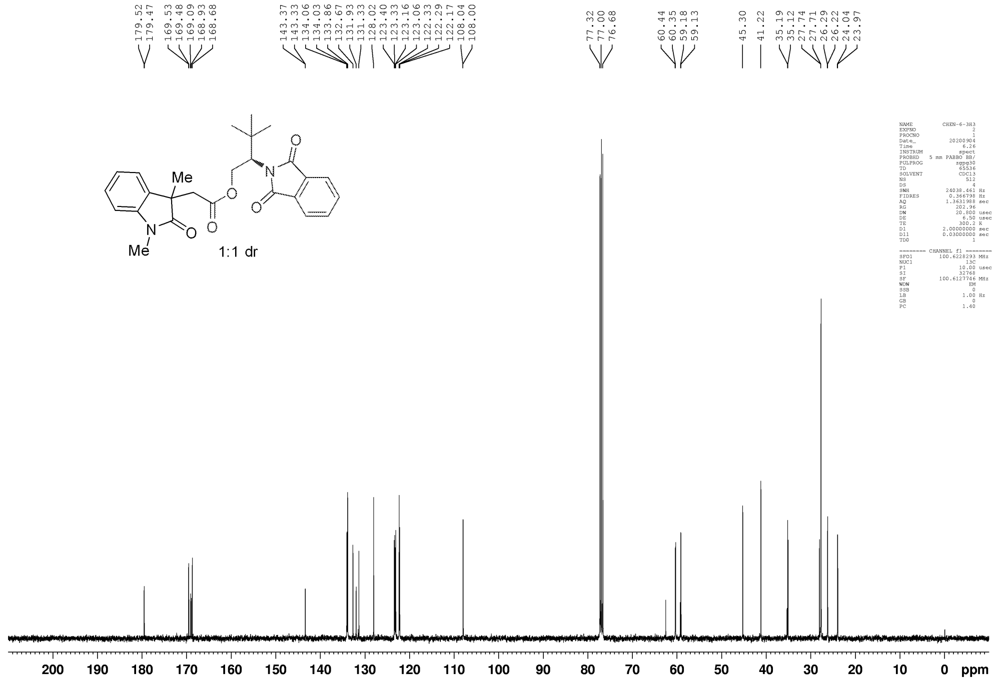

Supplementary Figure 194. ^{13}C NMR spectrum for compound **8ac**

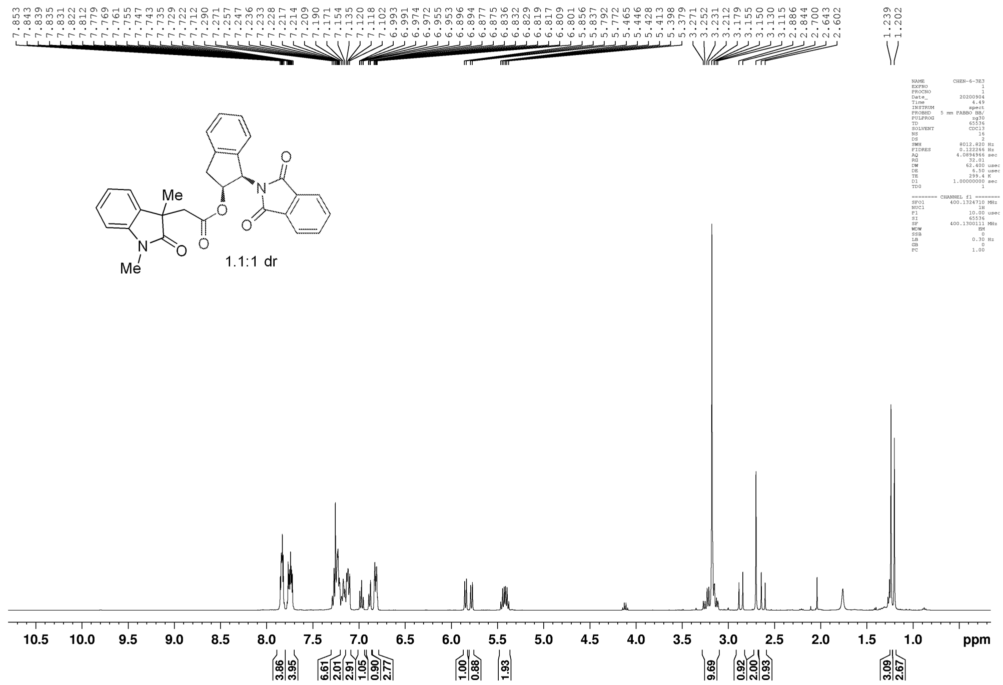

Supplementary Figure 195. ^{1}H NMR spectrum for compound **8ad**

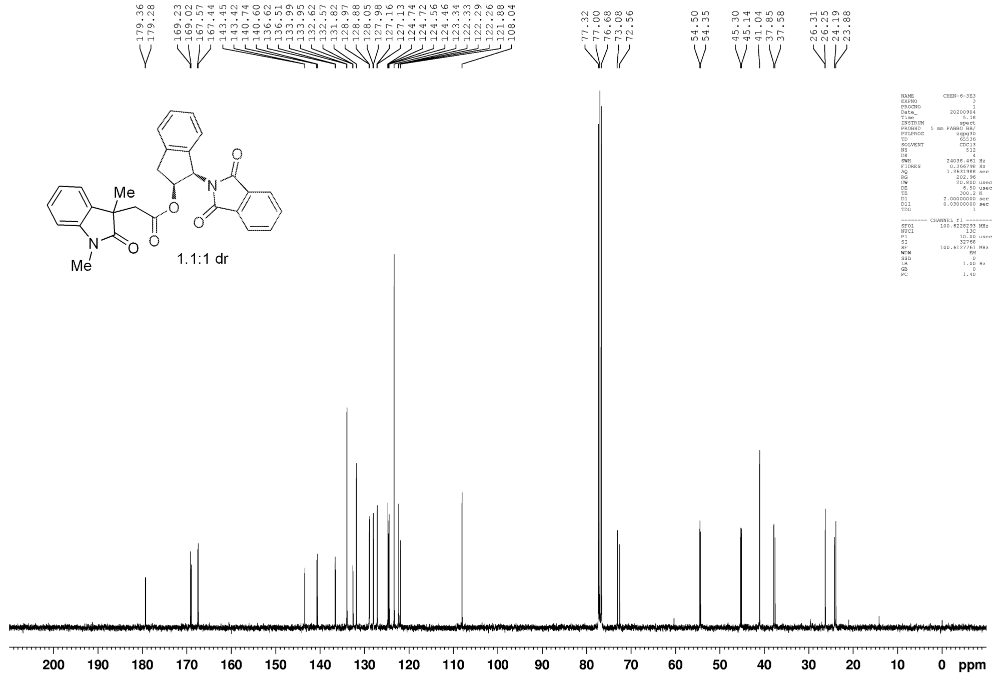

Supplementary Figure 196. ^{13}C NMR spectrum for compound **8ad**

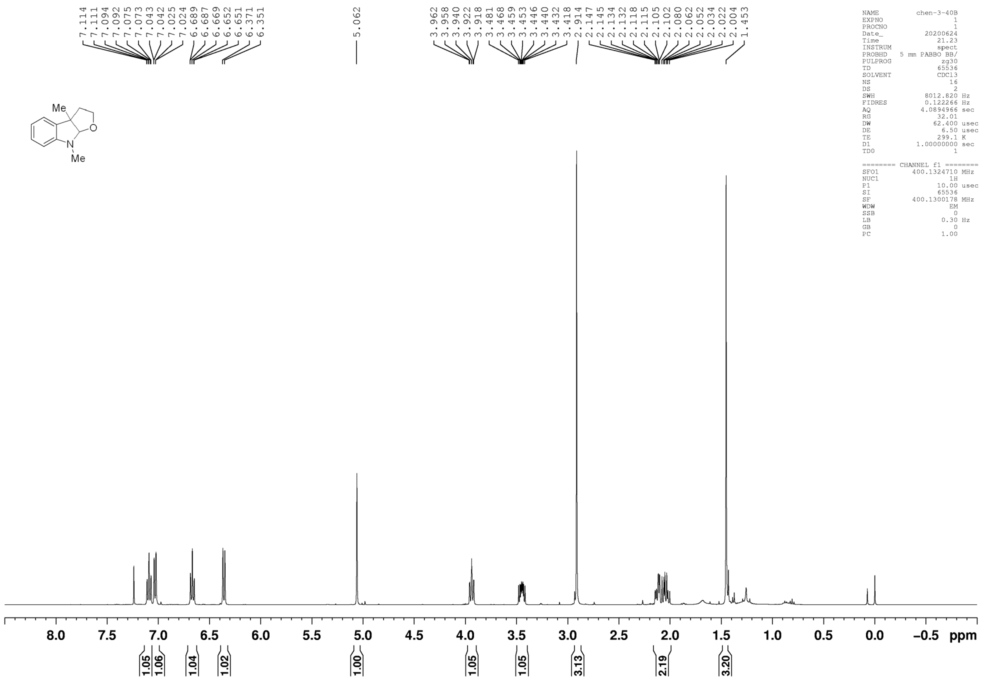

Supplementary Figure 197. ^{1}H NMR spectrum for compound **9a**

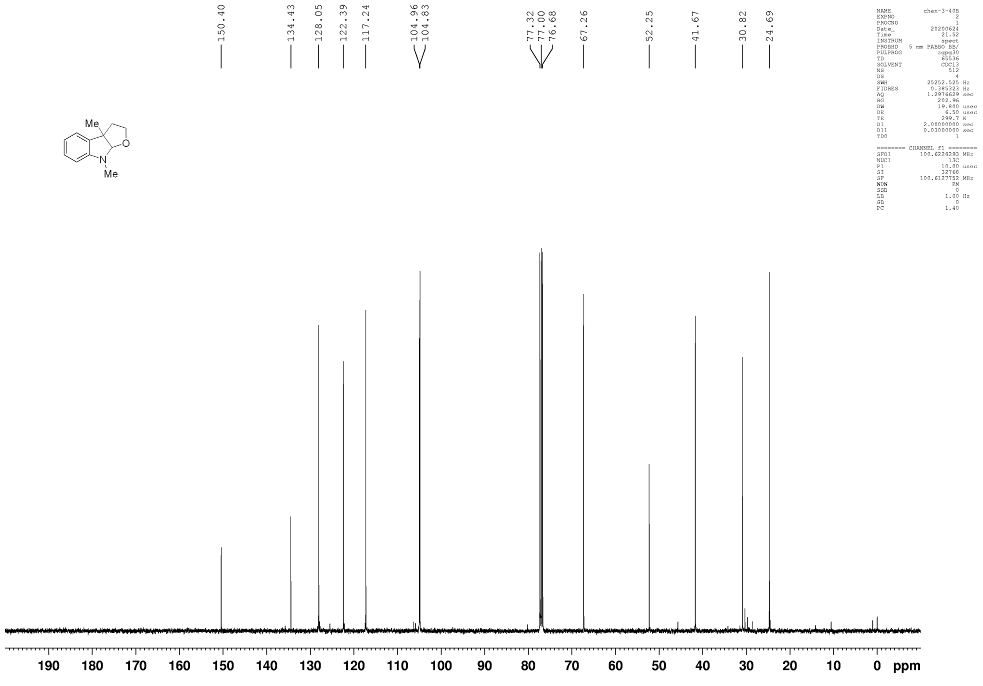

Supplementary Figure 198. ^{13}C NMR spectrum for compound **9a**

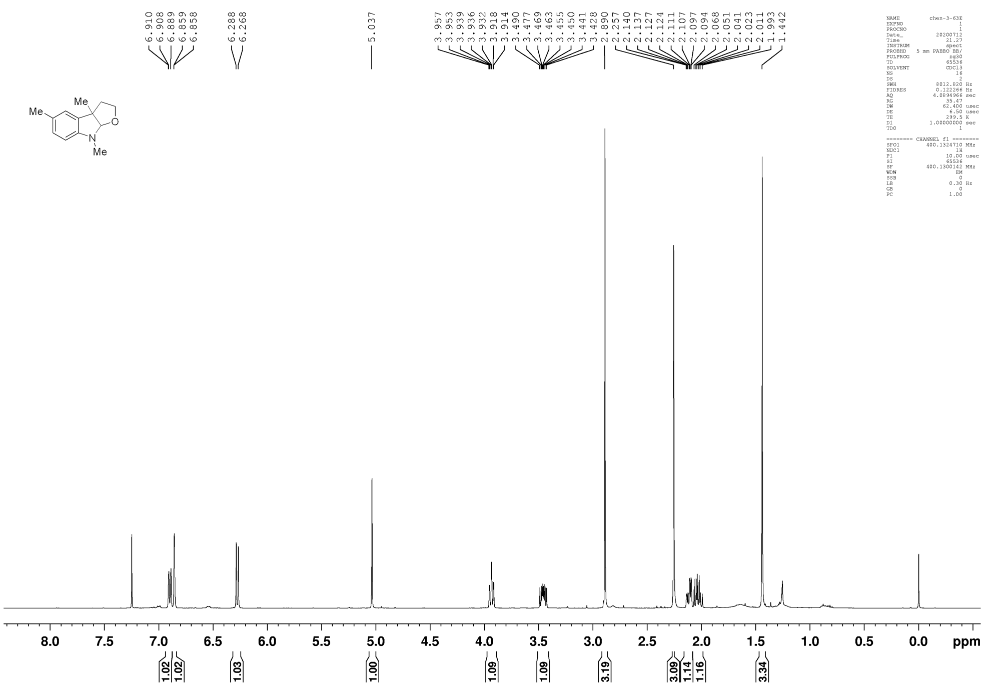

Supplementary Figure 199. ^{1}H NMR spectrum for compound **9b**

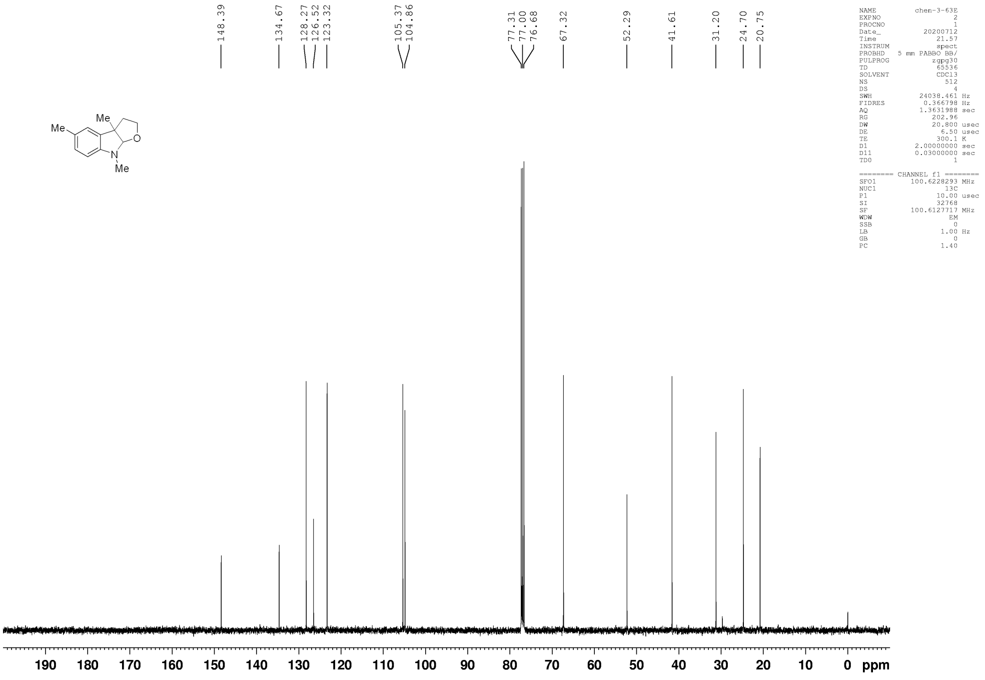

Supplementary Figure 200. ^{13}C NMR spectrum for compound **9b**

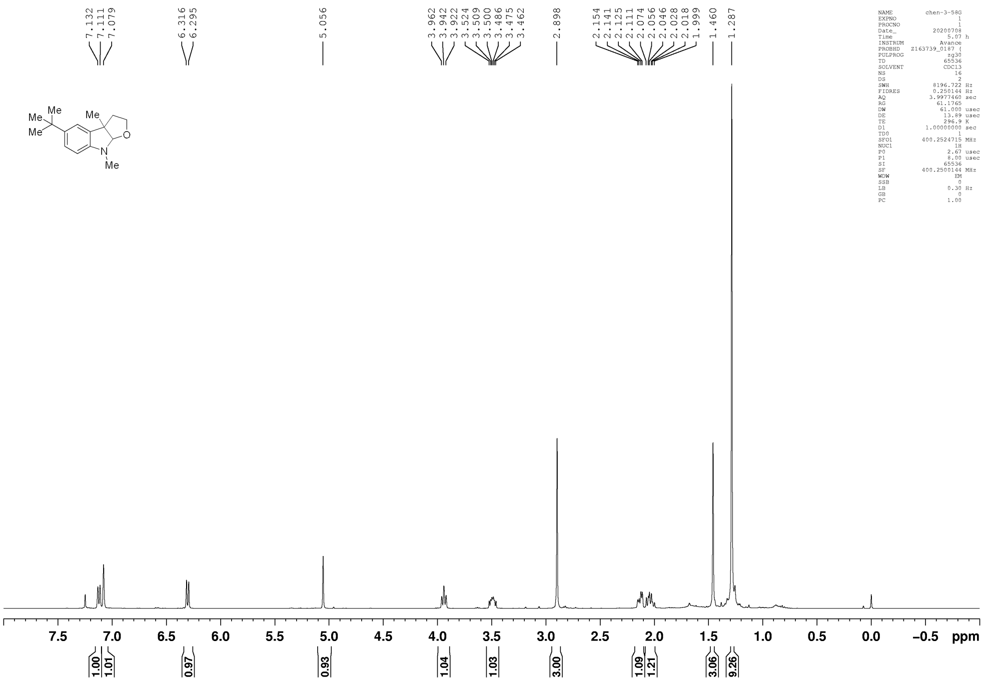

Supplementary Figure 201. ^{1}H NMR spectrum for compound **9c**

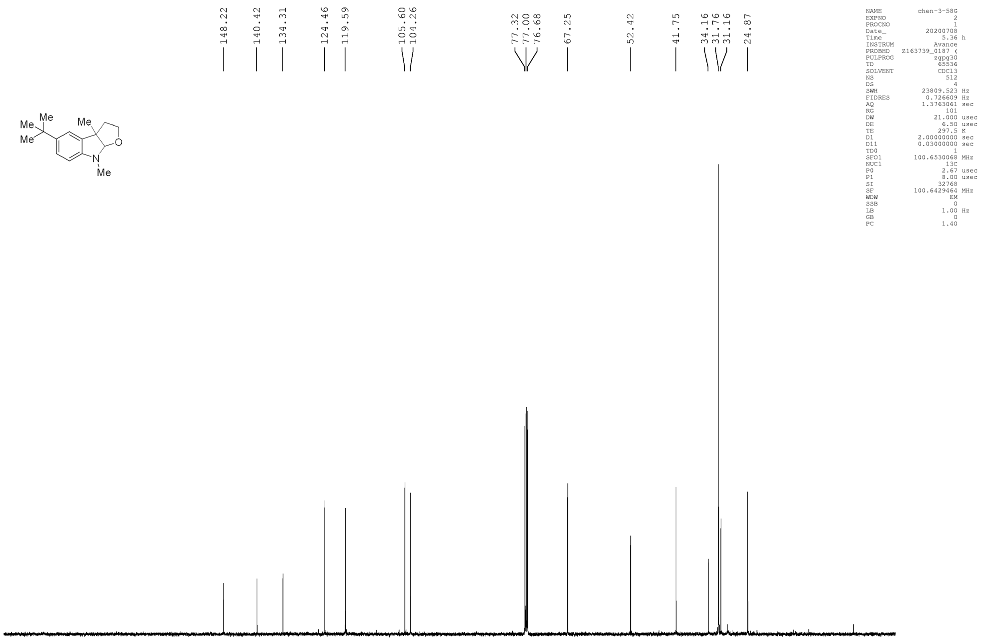

Supplementary Figure 202. ^{13}C NMR spectrum for compound **9c**

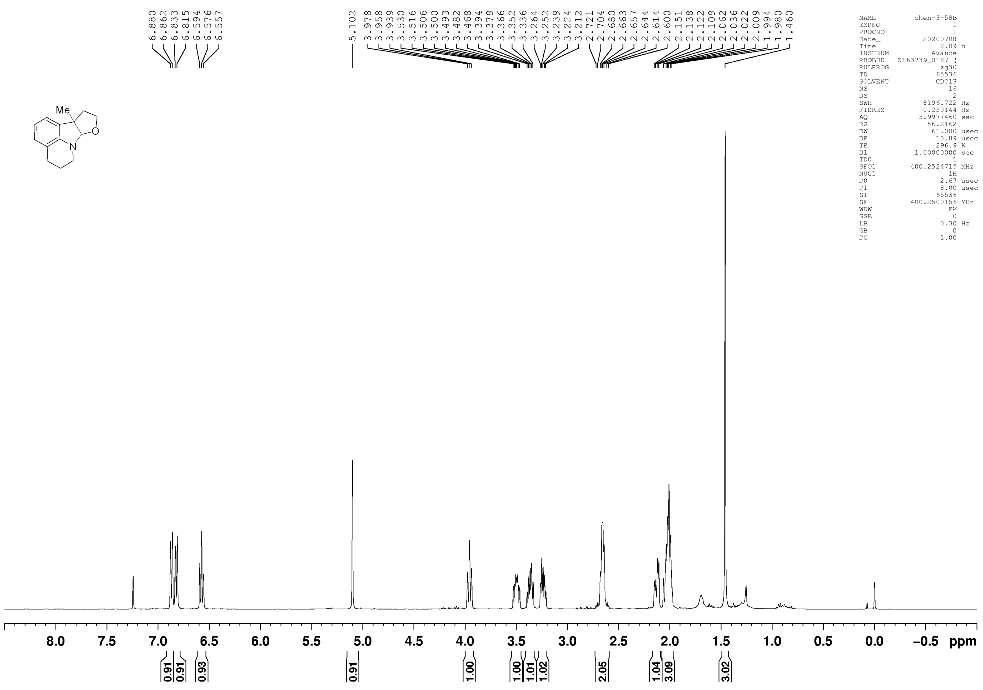

Supplementary Figure 203. ^{1}H NMR spectrum for compound **9d**

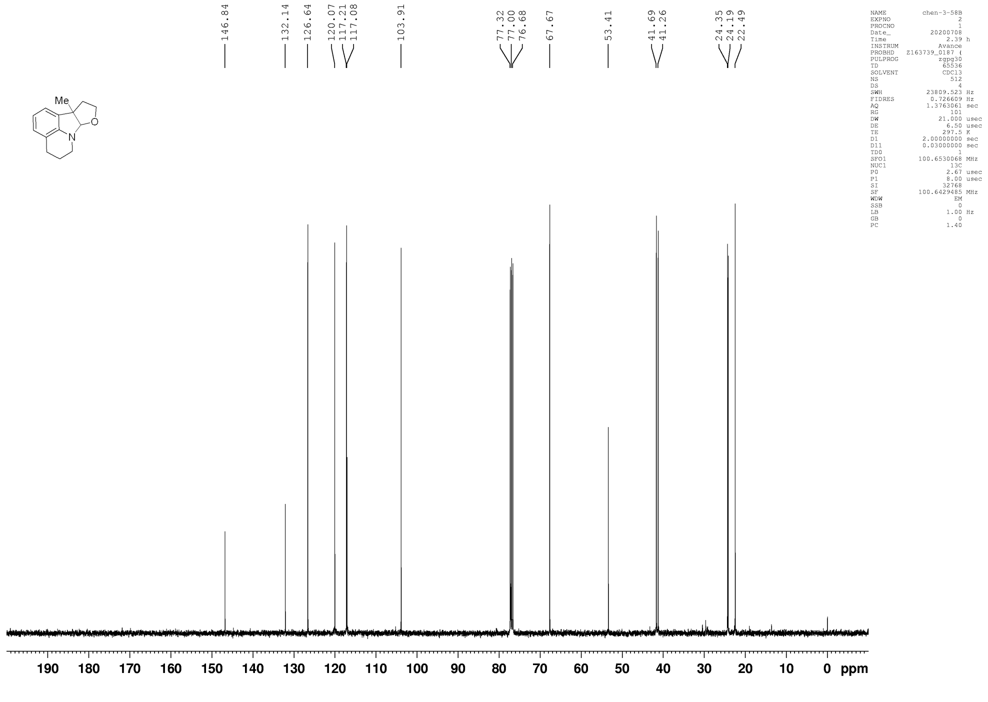

Supplementary Figure 204. ^{13}C NMR spectrum for compound **9d**

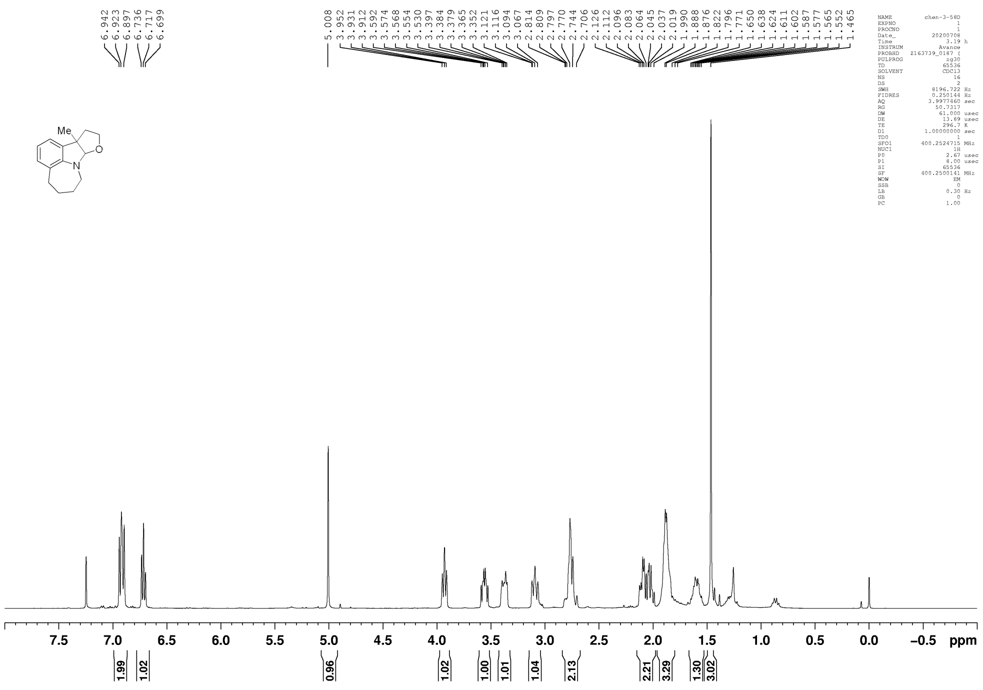

Supplementary Figure 205. ^{1}H NMR spectrum for compound **9e**

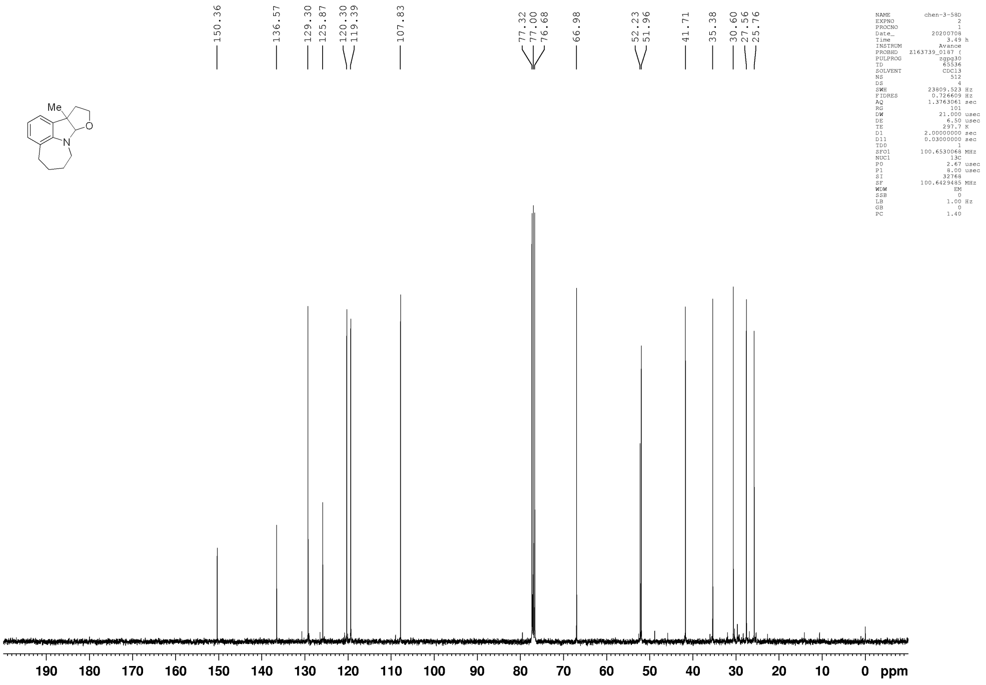

Supplementary Figure 206. ^{13}C NMR spectrum for compound **9e**

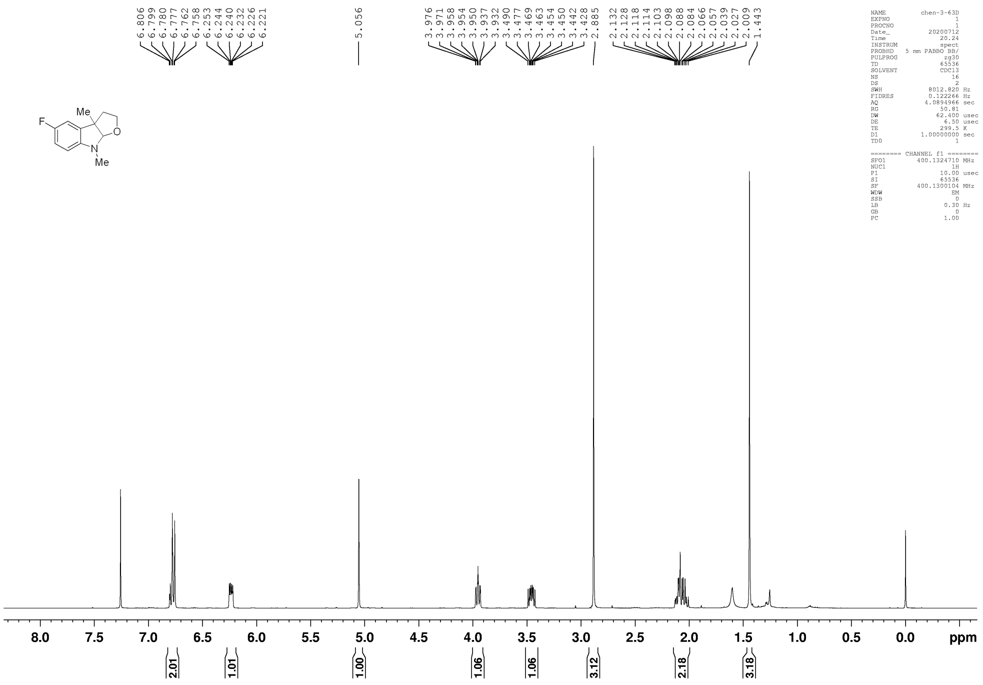

Supplementary Figure 207. ^{1}H NMR spectrum for compound **9f**

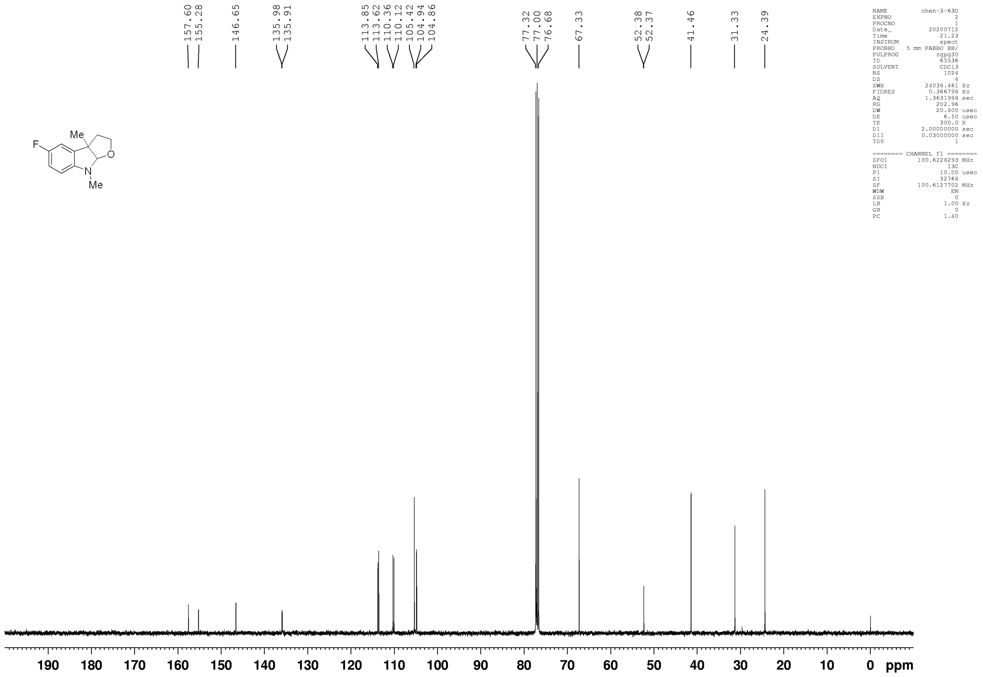

Supplementary Figure 208. ^{13}C NMR spectrum for compound **9f**

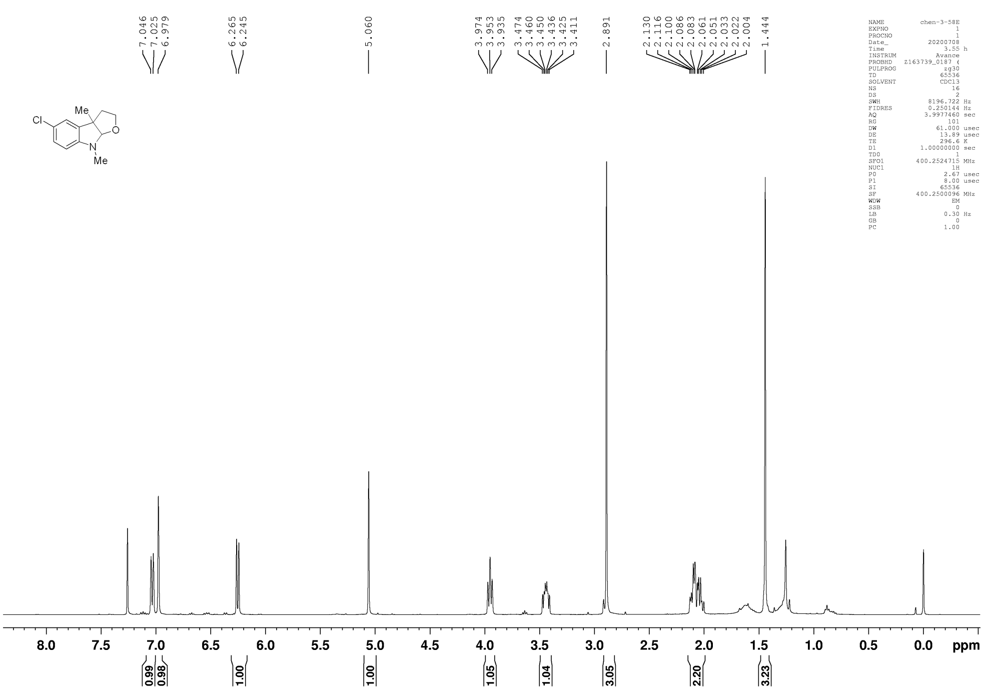

Supplementary Figure 209. ^{1}H NMR spectrum for compound **9g**

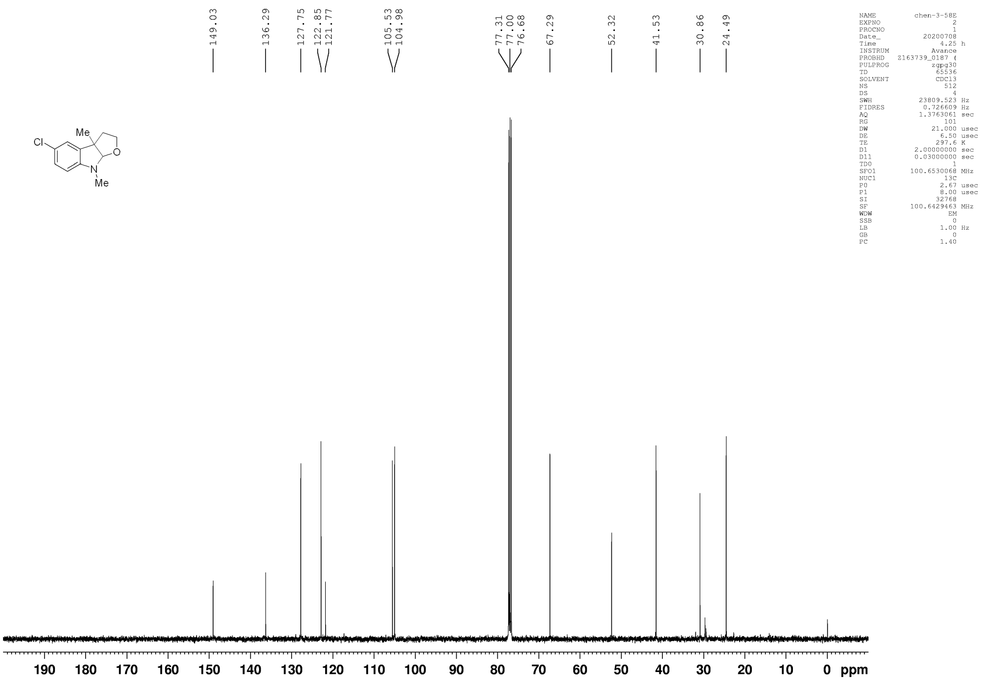

Supplementary Figure 210. ^{13}C NMR spectrum for compound **9g**

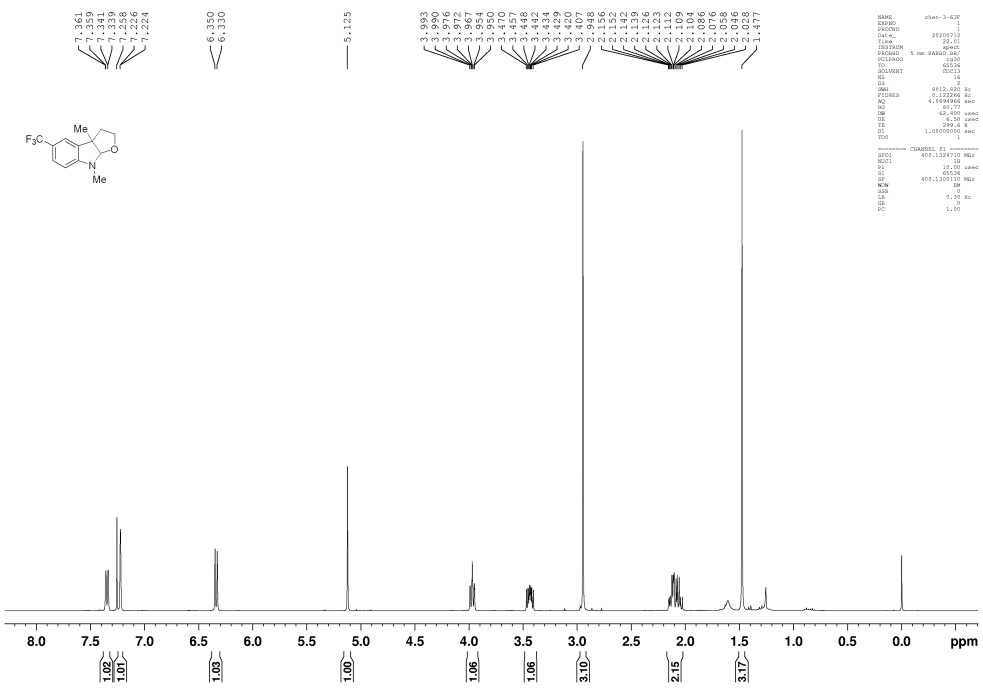

Supplementary Figure 211. ^{1}H NMR spectrum for compound **9h**

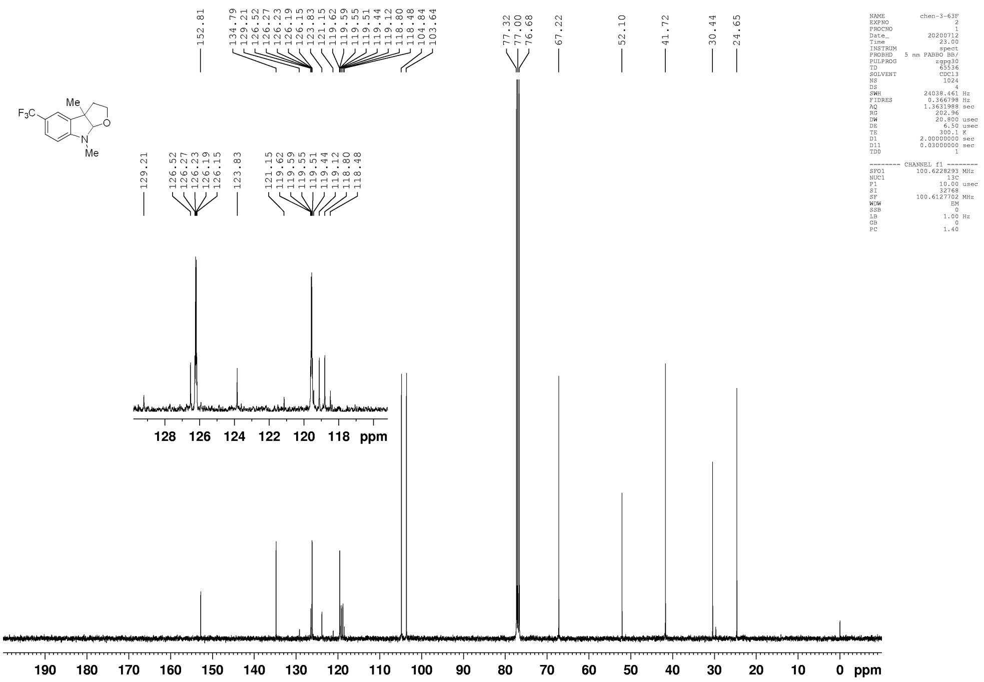

Supplementary Figure 212. ^{13}C NMR spectrum for compound **9h**

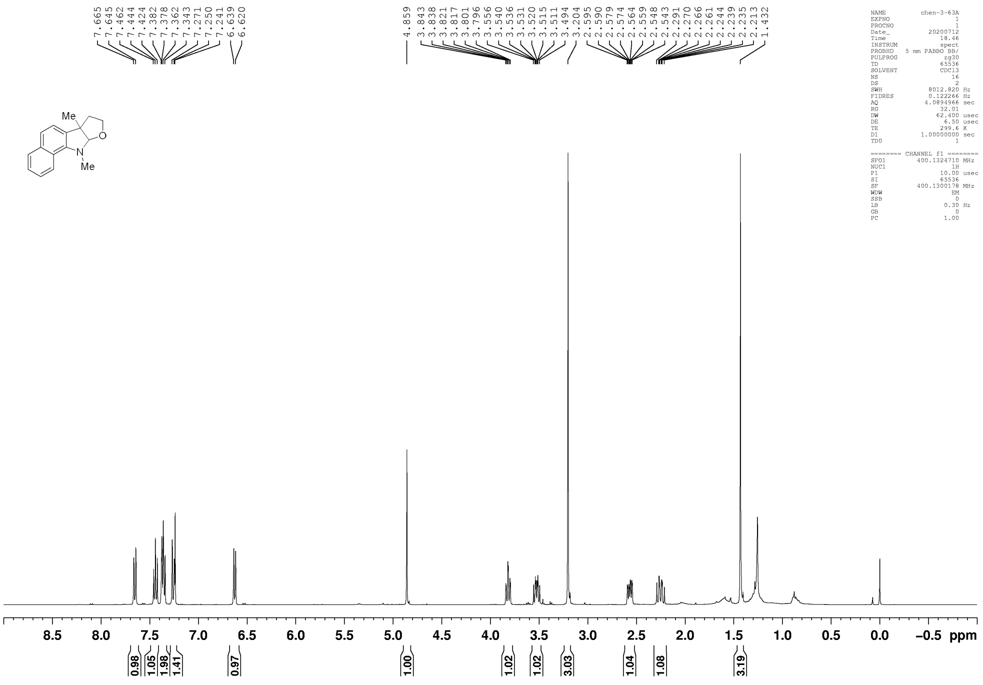

Supplementary Figure 213. ^{1}H NMR spectrum for compound **9i**

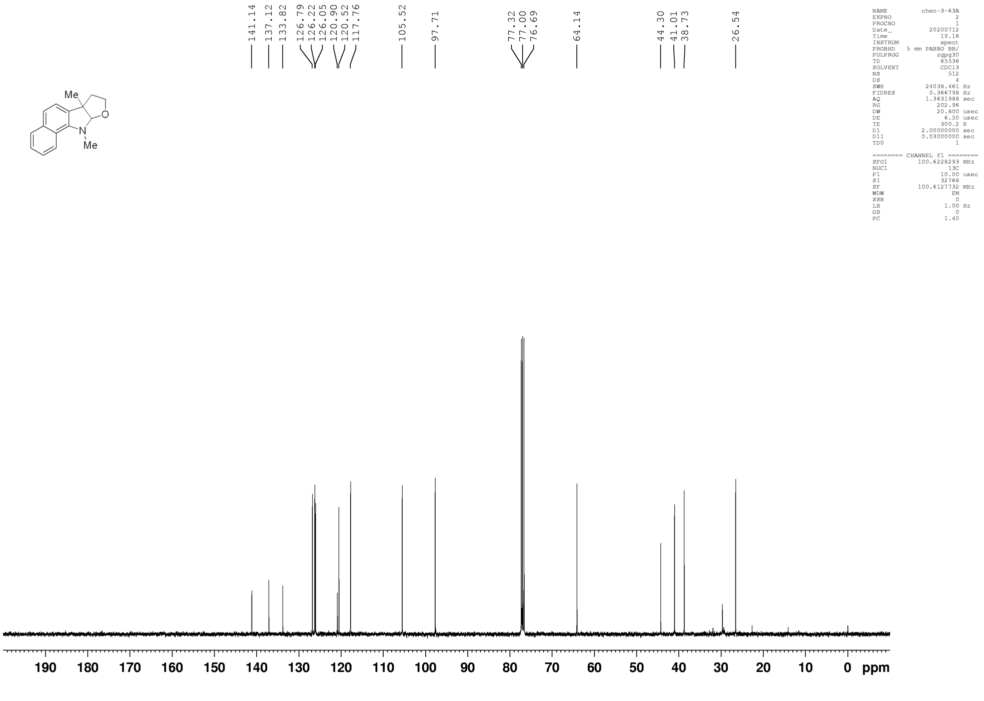

Supplementary Figure 214. ^{13}C NMR spectrum for compound **9i**

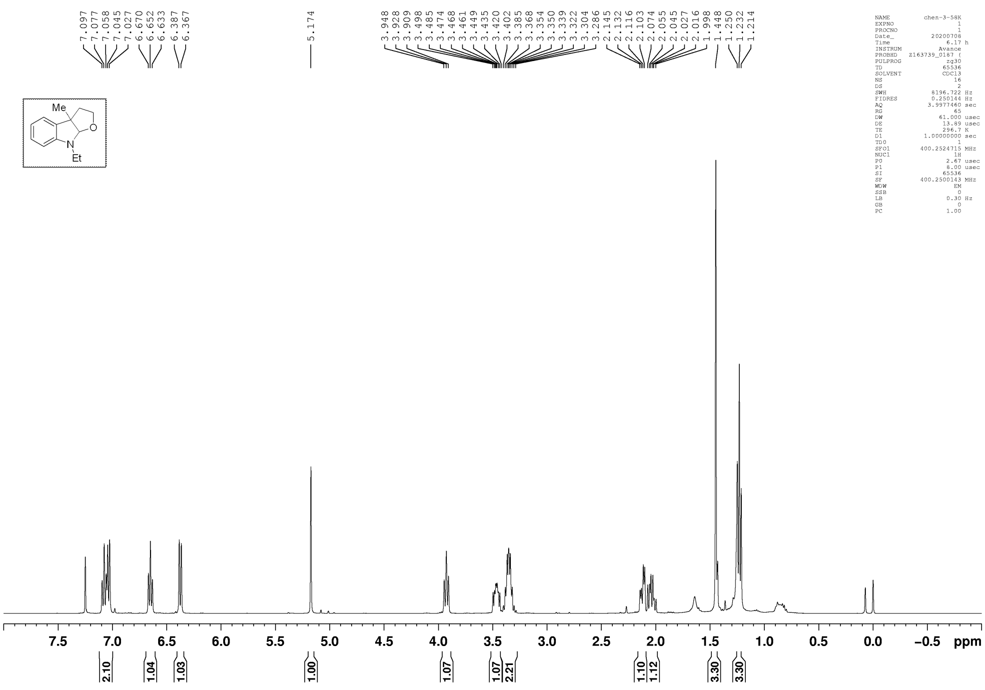

Supplementary Figure 215. ^{1}H NMR spectrum for compound **9j**

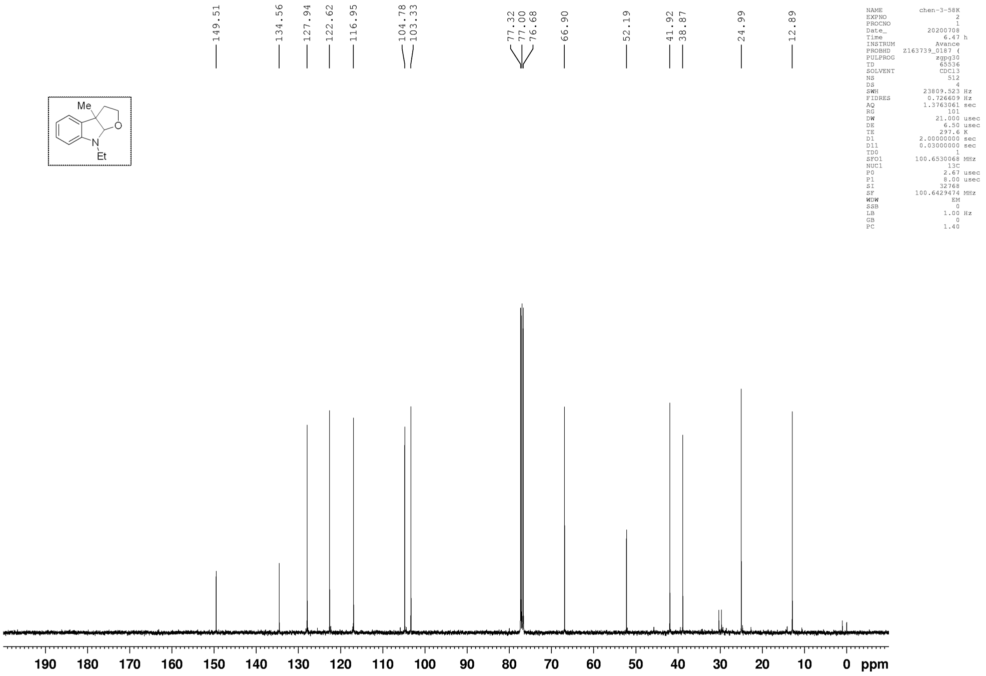

Supplementary Figure 216. ^{13}C NMR spectrum for compound **9j**

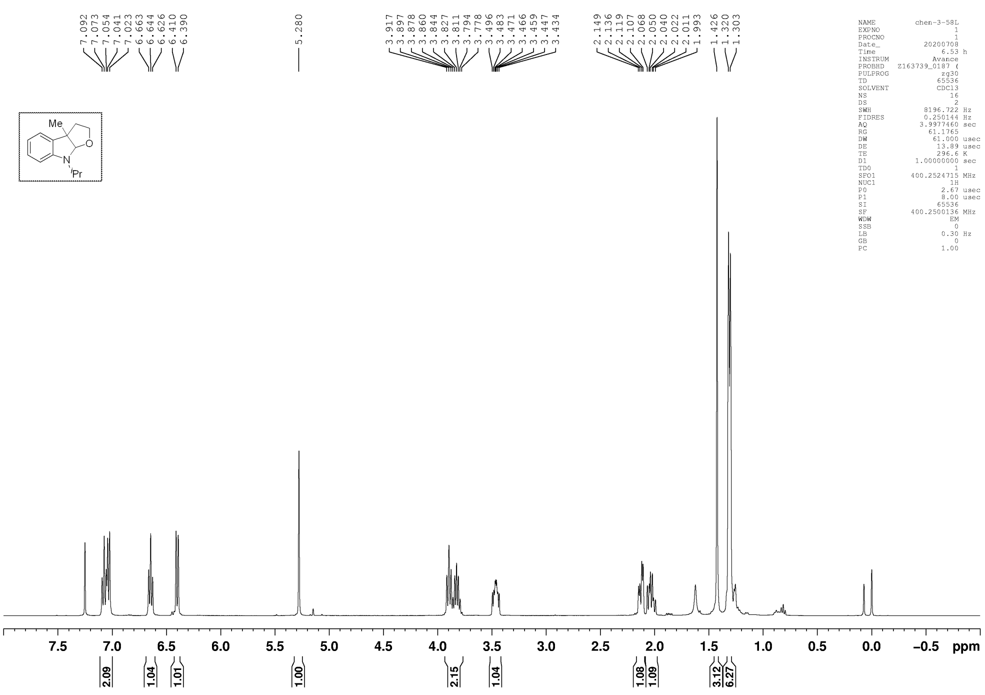

Supplementary Figure 217. ^{1}H NMR spectrum for compound **9k**

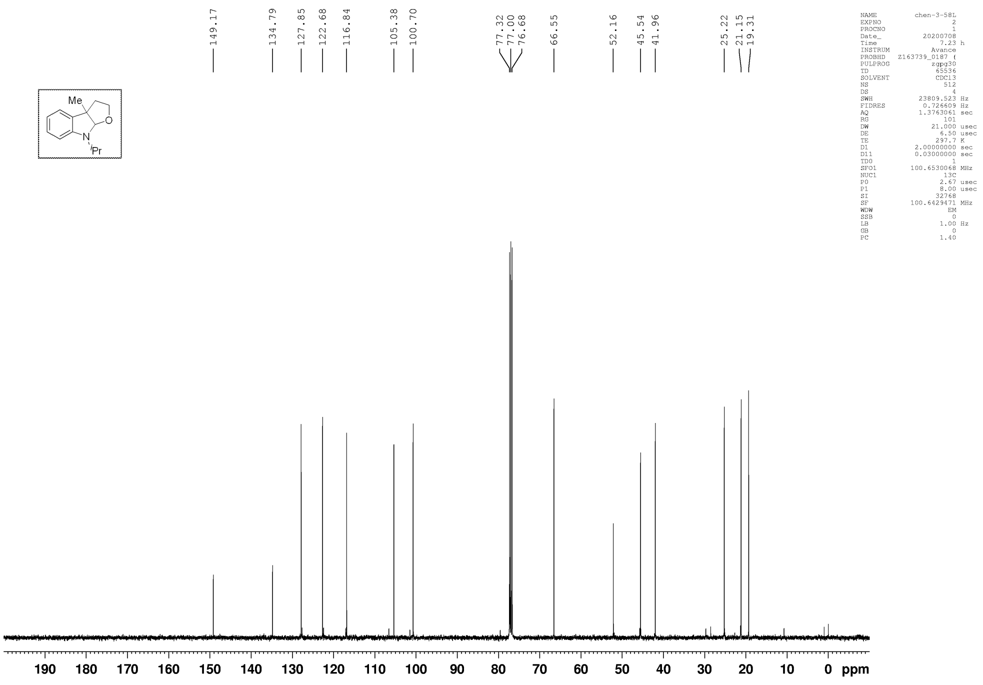

Supplementary Figure 218. ^{13}C NMR spectrum for compound **9k**

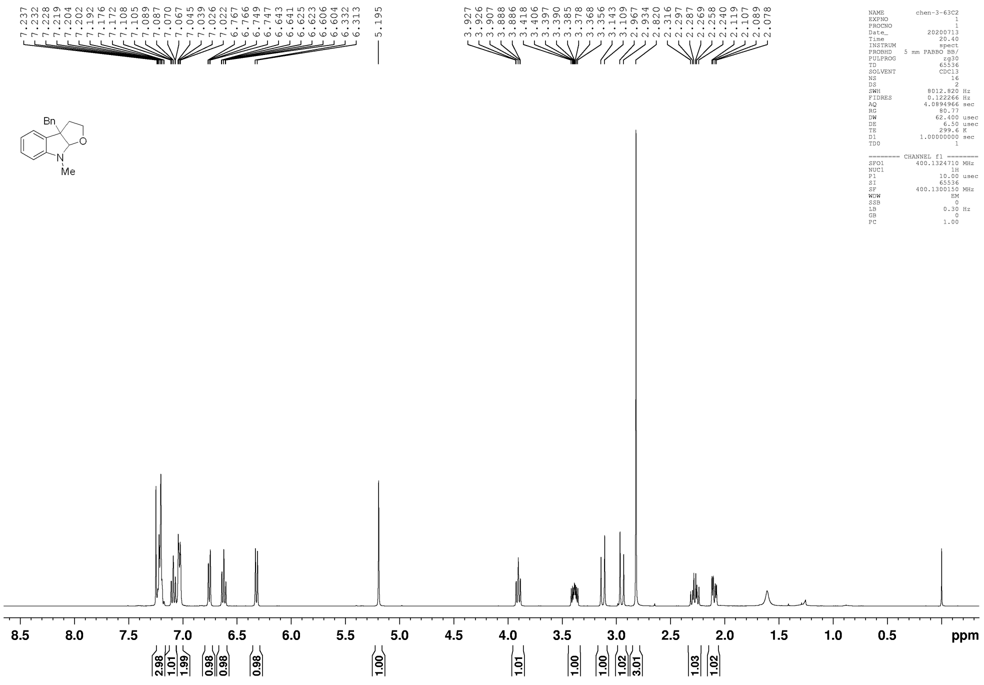

Supplementary Figure 219. ^{1}H NMR spectrum for compound **9l**

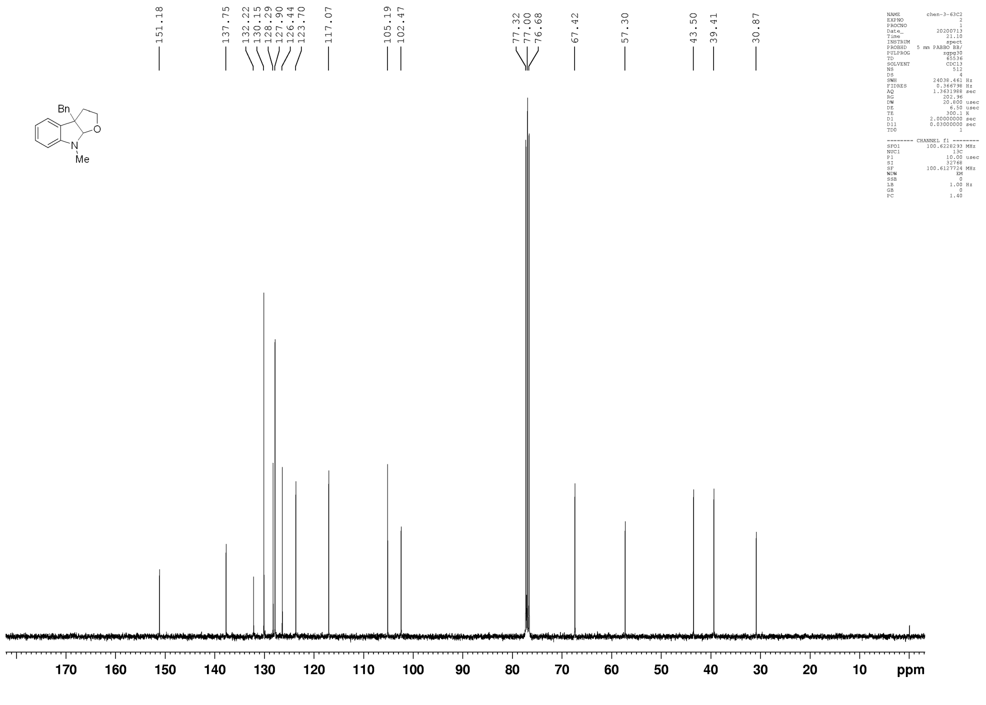

Supplementary Figure 220. ^{13}C NMR spectrum for compound **9l**

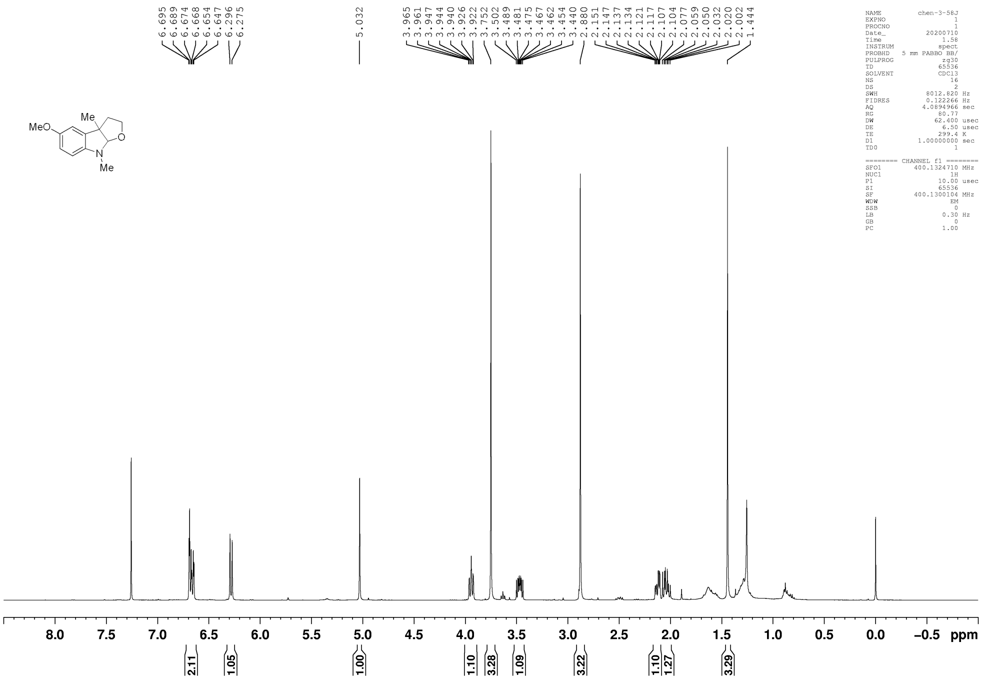

Supplementary Figure 221. ^{1}H NMR spectrum for compound **9m**

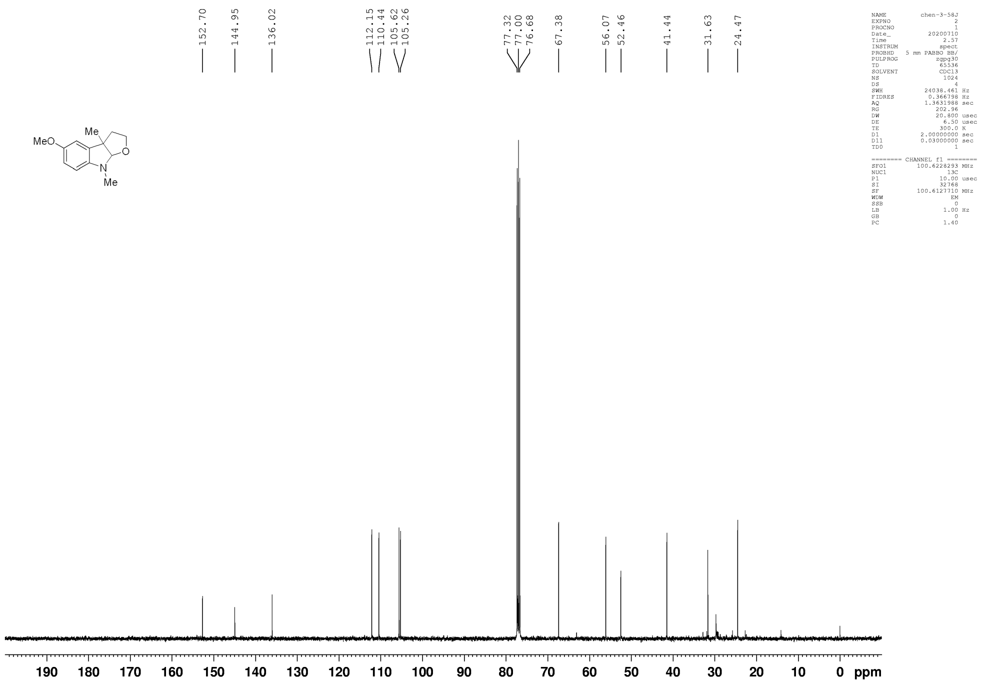

Supplementary Figure 222. ^{13}C NMR spectrum for compound **9m**

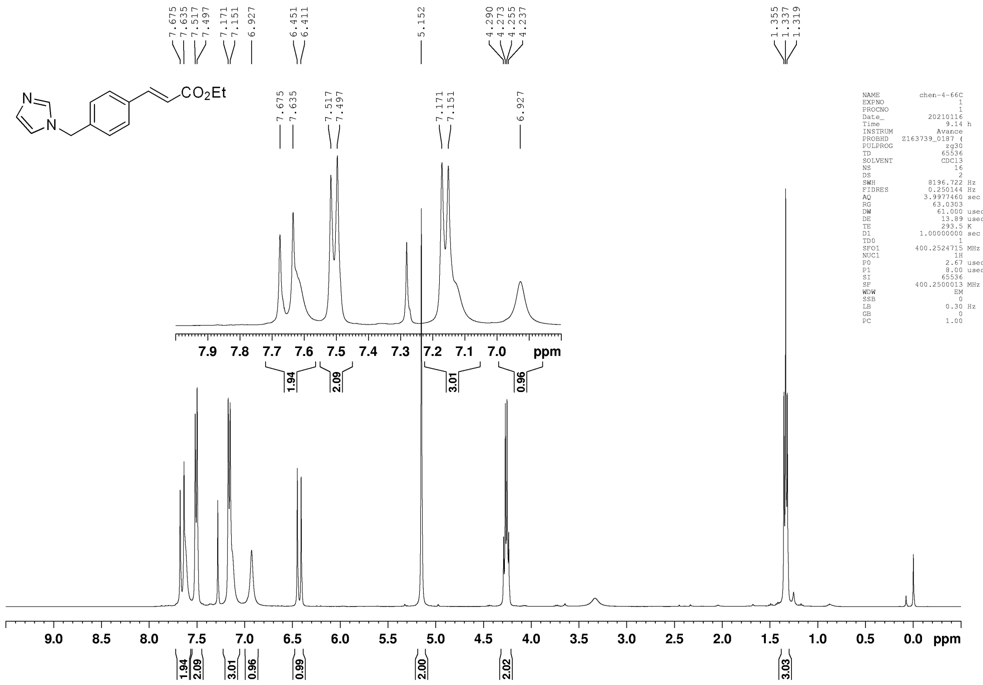

Supplementary Figure 223. ^{1}H NMR spectrum for compound **10i**

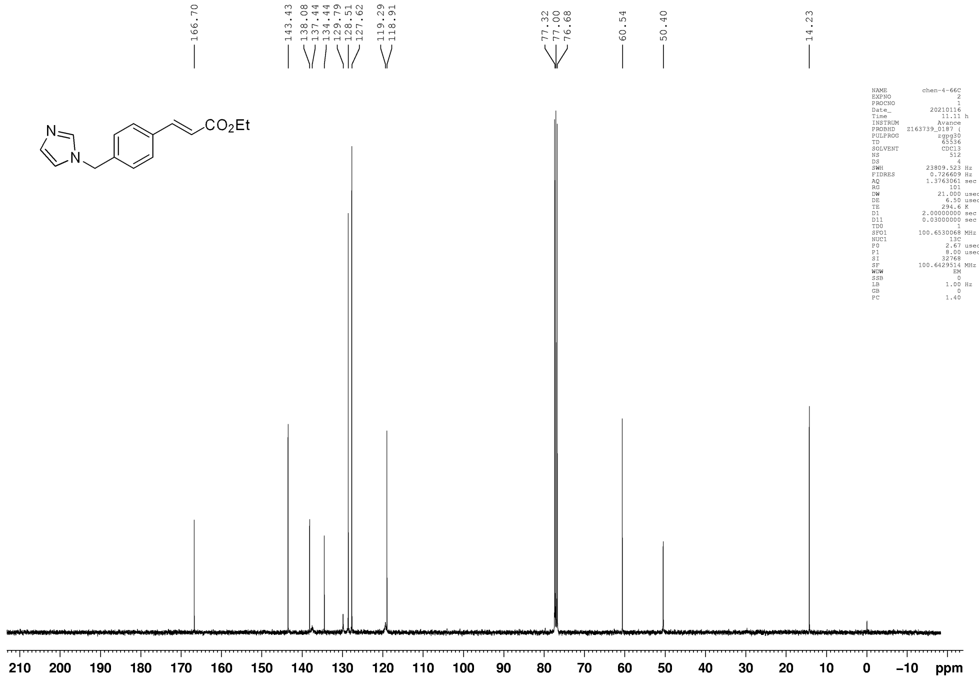

Supplementary Figure 224. ^{13}C NMR spectrum for compound **10i**

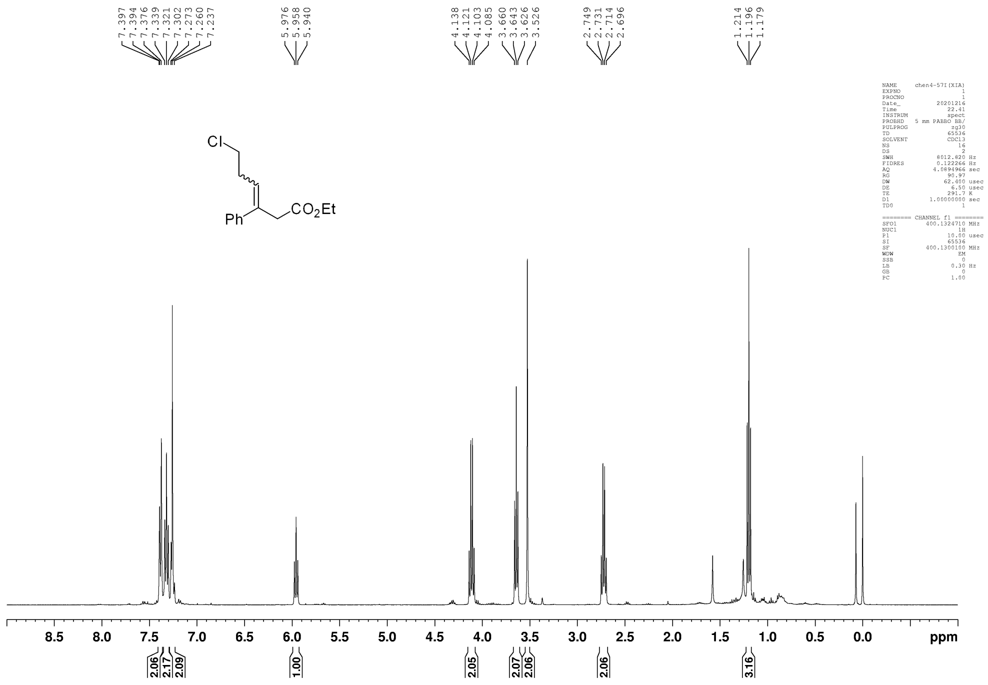

Supplementary Figure 225. ^{1}H NMR spectrum for compound **11b**

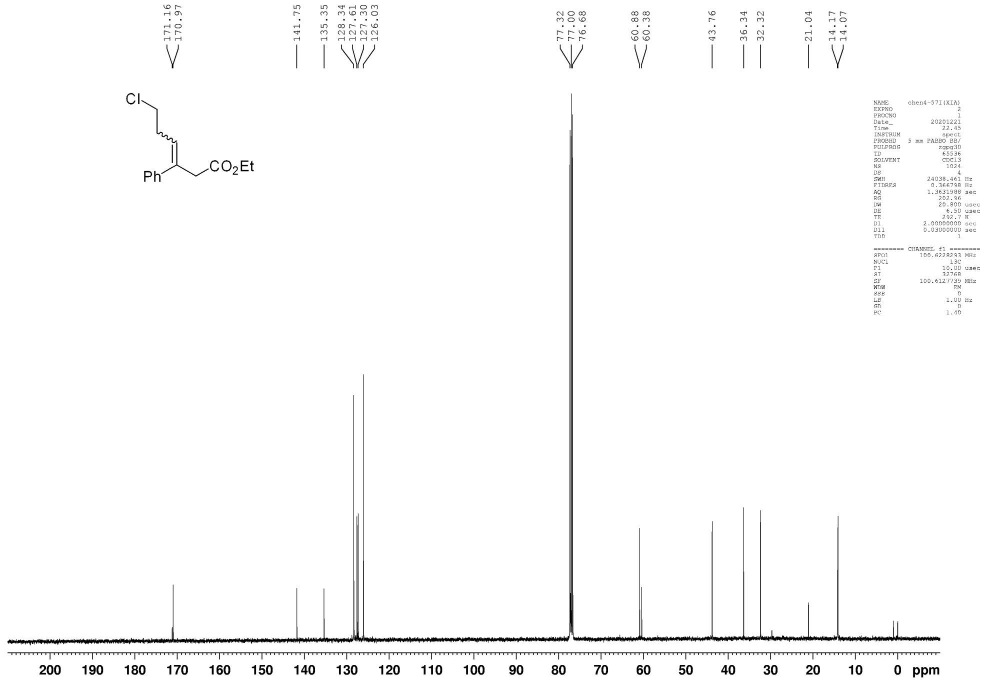

Supplementary Figure 226. ^{13}C NMR spectrum for compound **11b**

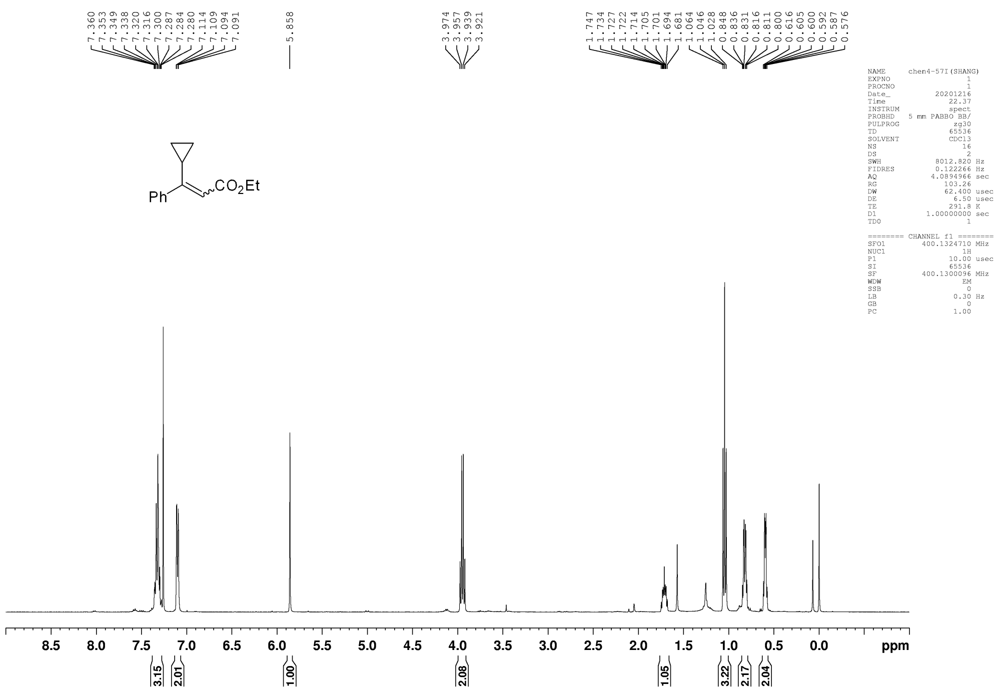

Supplementary Figure 227. ^{1}H NMR spectrum for compound **11c**

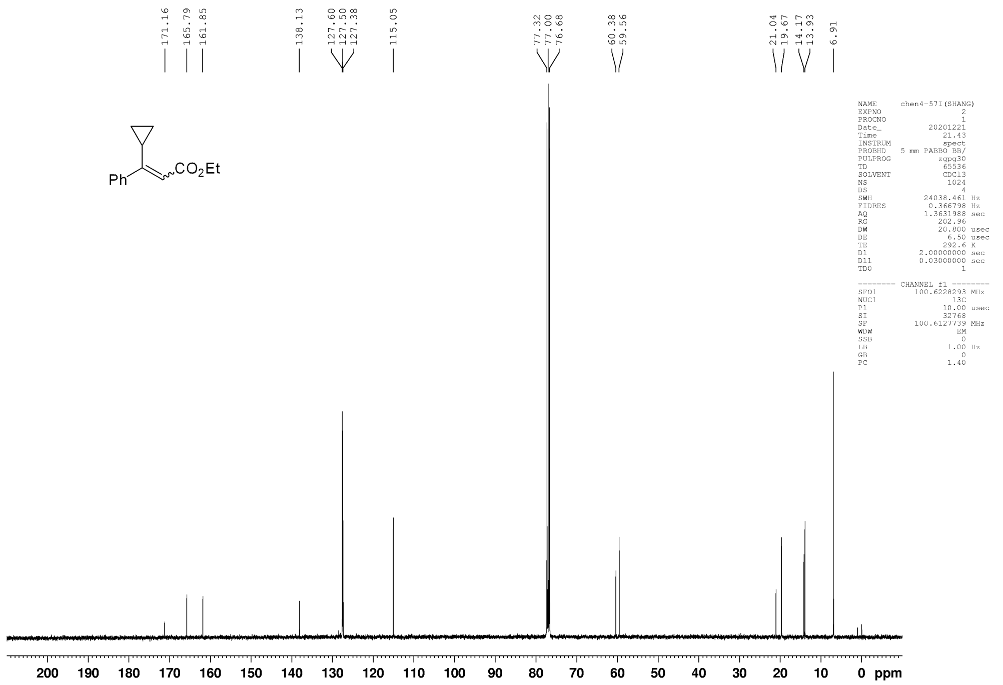

Supplementary Figure 228. ^{13}C NMR spectrum for compound **11c**

Supplementary References

1. Zhao, Y., Wang, G., Li, Y., Wang, S. & Li, Z. Design, synthesis and insecticidal activities of novel *N*-oxalyl derivatives of neonicotinoid compound. *Chin. J. Chem.* **28**, 475–479 (2010).
2. Yi, X., Lei, S., Liu, W., Che, F., Yu, C., Liu, X., Wang, Z., Zhou, X. & Zhang, Y. Copper-Catalyzed Radical *N*-Demethylation of Amides Using N-Fluorobenzenesulfonimide as an Oxidant. *Org. Lett.* **22**, 4583–4587 (2020).
3. Xu, J., Liang, L., Zheng, H., Chi, Y. R. & Tong, R. Green oxidation of indoles using halide catalysis. *Nat. Commun.* **10**, 4754–4764 (2019).
4. Arjomandia, O. K., Kavoosic, M. & Adibi, H. Synthesis and investigation of inhibitory activities of imidazole derivatives against the metallo-β-lactamase IMP-1. *Bioorg. Chem.* **92**, 103277–103285 (2019).
5. Yasuda, M. Yamasaki, S. Onishi, Y. & Baba, A. Indium-catalyzed direct chlorination of alcohols using chlorodimethylsilane−benzil as a selective and mild system. *J. Am. Chem. Soc.* **126**, 7186–7187 (2004).
6. Canestrari, D., Lancianesi, S., Badiola, E., Strinna, C., Ibrahim, H. & Adamo, M. F. A. Desulfurative chlorination of alkyl phenyl sulfides. *Org. Lett.* **19**, 918–921 (2017).
7. Vanos, C. M. & Lambert, T. H. Development of a catalytic platform for nucleophilic substitution: cyclopropenone-catalyzed chlorodehydration of alcohols. *Angew. Chem. Int. Ed.* **50**, 12222–12226 (2011).
8. Cismesiaa, M. A. & Yoon, T. P. Characterizing chain processes in visible light photoredox catalysis. *Chem. Sci.* **6**, 5426–5434 (2015).
9. Becke, A. D. Density - functional thermochemistry. III. The role of exact exchange. *J. Chem. Phys.* **98**, 5648–5652 (1993).
10. Stephens, P. J., Devlin, F. J., Chabalowski, C. F. & Frisch, M. J. Ab initio calculation of vibrational absorption and circular dichroism spectra using density functional force fields. *J. Phys. Chem.* **98**, 11623–11627 (1994).
11. Lee, C., Yang, W. & Parr, R. G. Development of the Colle-Salvetti correlation-

energy formula into a functional of the electron density. *Phys. Rev. B: Condens. Matter Mater. Phys.* **37**, 785–789 (1988).

12. Frisch, M. J., Trucks, G. W., Schlegel, H. B., Scuseria, G. E., Robb, M. A., Cheeseman, J. R., Scalmani, G., Barone, V., Mennucci, B., Petersson, G. A., Nakatsuji, H., Caricato, M., Li, X., Hratchian, H. P., Izmaylov, A. F., Bloino, J., Zheng, G., Sonnenberg, J. L., Hada, M., Ehara, M., Toyota, K., Fukuda, R., Hasegawa, J., Ishida, M., Nakajima, T., Honda, Y., Kitao, O., Nakai, H., Vreven, T., Jr. Montgomery, J. A., Peralta, J. E., Ogliaro, F., Bearpark, M., Heyd, J. J., Brothers, E., Kudin, K. N., Staroverov, V. N., Kobayashi, R., Normand, J., Raghavachari, K., Rendell, A., Burant, J. C., Iyengar, S. S., Tomasi, J., Cossi, M., Rega, N., Millam, N. J., Klene, M., Knox, J. E., Cross, J. B., Bakken, V., Adamo, C., Jaramillo, J., Gomperts, R., Stratmann, R. E., Yazyev, O., Austin, A. J., Cammi, R., Pomelli, C., Ochterski, J. W., Martin, R. L., Morokuma, K., Zakrzewski, V. G., Voth, G. A., Salvador, P., Dannenberg, J. J., Dapprich, S., Daniels, A. D., Farkas, O., Foresman, J. B., Ortiz, J. V., Cioslowski, J. & Fox, D. J. Gaussian 09, Revision D.01; Gaussian, Inc.: Wallingford, CT, 2009.
13. Fuentealba, P., Preuss, H., Stoll, H. & Szentpály, L. V. A proper account of core-polarization with pseudopotentials: single valence-electron alkali compounds. *Chem. Phys. Lett.* **89**, 418–422 (1982).
14. Marenich, A. V., Cramer, C. J. & Truhlar, D. G. Universal solvation model based on solute electron density and on a continuum model of the solvent defined by the bulk dielectric constant and atomic surface tensions. *J. Phys. Chem. B* **113**, 6378–6396 (2009).
15. Bartmess, J. E. Thermodynamics of the electron and the proton. *J. Phys. Chem.* **98**, 6420–6424 (1994).
16. Lei, Z., Banerjee, A., Kusevska, E., Rizzo, E., Liu, P. & Ngai, M.-Y. *β*-Selective aroylation of activated alkenes by photoredox catalysis. *Angew. Chem. Int. Ed.* **58**, 7318–7323 (2019).
